# Anti-cancer stem cell activity of a sesquiterpene lactone isolated from *Ambrosia arborescens* and of a synthetic derivative

**DOI:** 10.1371/journal.pone.0184304

**Published:** 2017-09-01

**Authors:** Wendy Soria Sotillo, Rodrigo Villagomez, Sandra Smiljanic, Xiaoli Huang, Atena Malakpour, Sebastian Kempengren, Gloria Rodrigo, Giovanna Almanza, Olov Sterner, Stina Oredsson

**Affiliations:** 1 Department of Biology, Lund University, Lund, Sweden; 2 Molecular Biology and Biotechnology Institute, University Major of San Andrés, La Paz, Bolivia; 3 Centre for Analysis and Synthesis, Department of Chemistry, Lund University, Lund, Sweden; 4 Chemical Research Institute, University Major of San Andres, La Paz, Bolivia; University of South Alabama Mitchell Cancer Institute, UNITED STATES

## Abstract

New regimens are constantly being pursued in cancer treatment, especially in the context of treatment-resistant cancer stem cells (CSCs) that are assumed to be involved in cancer recurrence. Here, we investigated the anti-cancer activity of sesquiterpene lactones (SLs) isolated from *Ambrosia arborescens* and of synthetic derivatives in breast cancer cell lines, with a specific focus on activity against CSCs. The breast cancer cell lines MCF-7, JIMT-1, and HCC1937 and the normal-like breast epithelial cell line MCF-10A were treated with the SLs damsin and coronopilin, isolated from *A*. *arborescens*, and with ambrosin and dindol-01, synthesized using damsin. Inhibitory concentration 50 (IC_50_) values were obtained from dose-response curves. Based on IC_50_ values, doses in the μM range were used for investigating effects on cell proliferation, cell cycle phase distribution, cell death, micronuclei formation, and cell migration. Western blot analysis was used to investigate proteins involved in cell cycle regulation as well as in the NF-κB pathway since SLs have been shown to inhibit this transcription factor. Specific CSC effects were investigated using three CSC assays. All compounds inhibited cell proliferation; however, damsin and ambrosin were toxic at single-digit micromolar ranges, while higher concentrations were required for coronopilin and dindol-01. Of the four cell lines, the compounds had the least effect on the normal-like MCF-10A cells. The inhibition of cell proliferation can partly be explained by downregulation of cyclin-dependent kinase 2. All compounds inhibited tumour necrosis factor-α-induced translocation of NF-κB from the cytoplasm to the nucleus. Damsin and ambrosin treatment increased the number of micronuclei; moreover, another sign of DNA damage was the increased level of p53. Treatment with damsin and ambrosin decreased the CSC subpopulation and inhibited cell migration. Our results suggest that these compounds should be further investigated to find efficient CSC-inhibiting compounds.

## Introduction

The major cause for cancer mortality is the recurrence of treatment-resistant cancer cells found locally or as metastases at distant organ sites. Conventional treatments such as surgery, chemotherapy, and irradiation apparently do not obliterate all cancer cells. Current research shows that a tumour is an hierarchical organization of cells with different phenotypes and genotypes [1]. At the top of the cancer cell hierarchy is the proposed cancer stem cell (CSC) [2]. The CSC has the main properties of a normal stem cell, *i*.*e*., self-renewal and differentiation [3]. In addition, similar to a normal stem cell, CSCs appear to have an increased survival capacity towards different types of damage caused by chemical and physical insults [4]. The targeting of the small population of CSCs may lie at the centre of identifying a cure for cancer. CSCs were first identified in leukaemia [4] but have henceforth been found in solid tumours [5] and in cancer cell lines derived from different organs [5]. CSCs are identified based on cell surface and intracellular markers [6] and functional capacities, such as tumour formation in immunocompromised animals [7] or colony formation under serum-free conditions *in vitro* [8]. There is a growing interest in finding compounds that lead to the development of new drugs and can be used in the clinic to target CSCs. Our study focused on sesquiterpene lactones (SLs) isolated from *Ambrosia arborescens*, a plant used in traditional medicine in many South American countries [9].

SLs are a group of secondary metabolites found across the plant kingdom [10]. A feature of SLs is the presence of an α-methylene-γ-lactone ring, resulting in a high alkylating ability. Thus, these molecules are thought to act by alkylating nucleophiles in the cell, namely, proteins containing sulfhydryl groups. One specific target suggested for SLs is p65, one of the members of the heterodimeric transcription factor NF-κB, which regulates a broad range of biological activities such as immune responses, development, survival, proliferation, angiogenesis, invasion, and metastasis [11] [12]. We focused on the SLs damsin and coronopilin isolated from *A*. *arborescens* [13]. In addition, we included the two compounds ambrosin and dindol-01, which were synthesized from the isolated damsin. We initially found that all compounds inhibited tumour necrosis factor-α (TNF-α)-induced translocation of NF-κB to the cell nucleus. Dose response assays showed that all compounds were cytotoxic to the breast cancer cell lines (MCF-7, JIMT-1, and HCC1937) as well as to the MCF-10A normal-like breast epithelial cell line; however, the latter cell line was least affected. The most toxic compound was ambrosin, which was also found to reduce the CSC subpopulation of the JIMT-1 cell line.

## Methods

### Compounds and stock solutions

**Fig 1:**
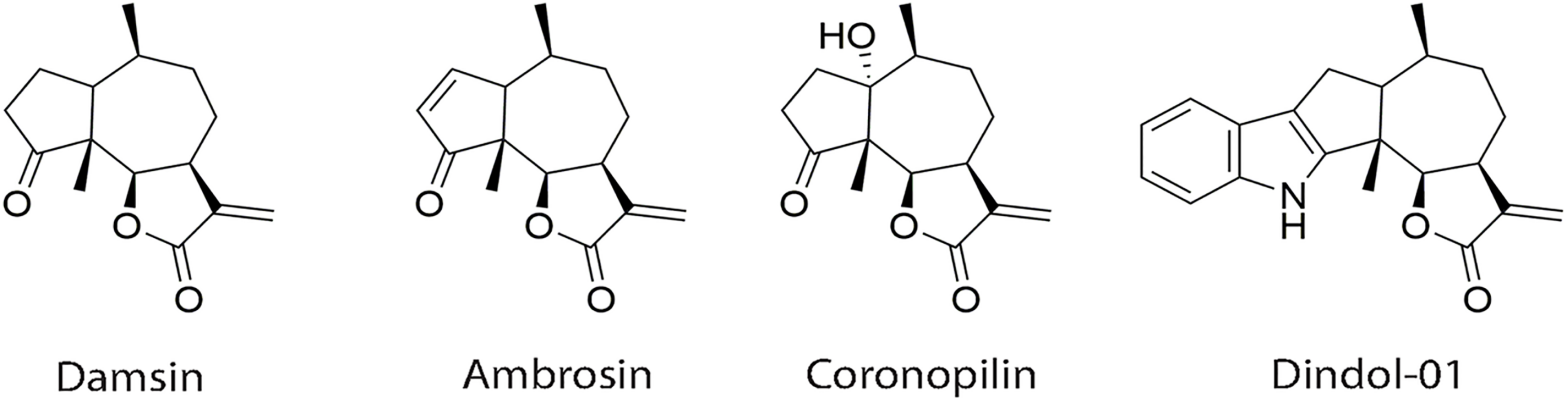
Chemical structures of damsin, ambrosin, coronopolin, and dindol-01.

The natural sesquiterpene lactones used in this study, damsin and coronopilin, were isolated from *A*. *arborescens* [14] (Fig 1). Ambrosin and dindol-01 were semi-synthesized from damsin [14] (Fig 1).

The compounds were dissolved in 100% DMSO as a 100 mM stock solution, which was stored at -20°C. The compounds were then diluted in phosphate-buffered saline (PBS: 8 g/l NaCl, 0.2 g/L KCl, 1.15 g/l Na_2_HPO_4_, 0.2 g/l KH_2_PO_4_, pH 7.3) to prepare the working solutions at the appropriate concentrations. The controls were supplemented with PBS containing DMSO at the same concentrations as the working solutions of the compounds. The final DMSO concentration was equal to or less than 0.1% in all assays.

### Cell lines and culture conditions

The human breast cancer cell lines MCF-7 (HTB-22) and HCC1937 (CRL-2336) as well as the human normal-like breast epithelial cell line MCF-10A (CRL-10317) were purchased from American Type Culture Collection (Manassas, VA, USA). The MCF-7 cells were cultured in RPMI 1640 medium supplemented with 10% heat-inactivated foetal calf serum (FCS) (VWR, Lund, Sweden), 1 mM non-essential amino acids (VWR), 10 μg/ml insulin (Sigma-Aldrich, Stockholm, Sweden), and 100 U/ml penicillin/100 μg/ml streptomycin (VWR). The MCF-10A cells were cultured in RPMI 1640 medium (VWR) supplemented with 10% heat-inactivated FCS (VWR), 1 mM non-essential amino acids (VWR), 10 μg/ml insulin (Sigma-Aldrich), 20 ng/ml epidermal growth factor (Sigma-Aldrich), 50 ng/ml cholera toxin (Sigma-Aldrich), 250 ng/ml hydrocortisol (Sigma-Aldrich), and 100 U/ml penicillin/100 μg/ml streptomycin (VWR). The HCC1937 cells were cultured in RPMI 1640 medium (VWR) supplemented with 10% heat-inactivated FCS (VWR), 1 mM non-essential amino acids (VWR), 10 μg/ml insulin (Sigma-Aldrich), 20 ng/ml epidermal growth factor (Sigma-Aldrich) and 100 U/ml penicillin/100 μg/ml streptomycin (VWR). The human breast carcinoma cell line JIMT-1 (ACC589) was purchased from the German Collection of Microorganisms and Cell Cultures (DSMZ, Braunschweig, Germany) and routinely cultured in DMEM/Ham’s F-12 medium (VWR) supplemented with 10% FCS (VWR), 1 mM non-essential amino acids (VWR), 10 mg/ml insulin (Sigma-Aldrich), and 100 U/ml penicillin/100 μg/ml streptomycin (VWR). All cell lines were kept at 37°C in a humidified incubator with 5% CO_2_. For the experiments, cells were seeded at the following densities: MCF-10A: 10^4^ cells/cm^2^, MCF-7: 2×10^4^ cells/cm^2^, HCC1937: 2×10^4^ cells/cm^2^ and JIMT-1: 1.5×10^4^ cells/cm^2^, in tissue culture vessels of the appropriate size to obtain the desired cell number for the different assays. The volume of medium used was 0.2–0.3 ml per cm^2^. The cells were allowed to attach for 24 hours before the addition of the compounds.

### MTT assay

For the MTT assay, cells were detached by trypsinization and counted in a haemocytometer. Cells were then seeded into 96-well plates at a density described above in 180 μl medium per well. Twenty-four hours after incubation in the CO_2_ incubator, the compounds were added to a final concentration 0.1, 1, 2.5, 5, 10, 25, 50, and 100 μM. At 72 hours following drug treatment, 20 μl of MTT (Sigma-Aldrich) solution (5 mg/ml in PBS) was added to each well, and the 96-well plates were returned to the CO_2_ incubator for 1 hour. The blue formazan product, formed by reduction in live attached cells, was dissolved by adding 100 μl of 100% DMSO per well. The plates were gently swirled at room temperature for 10 minutes to dissolve the precipitate. Absorbance was monitored at 540 nm using a Labsystems iEMS Reader MF (Labsystems Oy, Helsinki, Finland) and the software DeltaSoft II v.4.14 (Biometallics Inc., Princeton, NJ, USA). Dose-response curves and IC_50_ values were obtained using GraphPad Prism software. At least 3 dose-response experiments were performed for each compound, and the mean IC_50_ ± SD was calculated.

### Inhibition of TNF-α-induced translocation of p65/NF-κB to the nucleus

Round glass cover slips (13 mm diameter) were placed in 12-well plates, and JIMT-1 or MCF-7 cells (0.1 × 10^6^) were seeded in the wells in complete medium. The 12-well plates were incubated for 24 hours to allow attachment of the cells. The cover slips were transferred to new 12-well plates, and 800 μl medium supplemented with 0.1% FCS was added to each well. The cells were treated with the compounds at IC_50_ for 60 minutes in the incubator, and then, TNF-α (25 ng/ml) was added to each well and incubation was continued for another 40 minutes. Then, the cells were fixed with 2 ml of 3.7% formaldehyde in PBS for 15 minutes at 4°C. To block nonspecific binding sites and to permeabilize the cells, the samples were incubated with blocking/permeabilization buffer (1% bovine serum albumin and 1% Tween 20 in PBS) at room temperature for 1 hour and then washed twice with PBS. Then, the cells were incubated with rabbit anti-p65/NF-κB (Abcam, Cambridge, MA, USA) (diluted 1:100 in blocking buffer), and incubation was continued for 1 hour at room temperature, followed by washing and incubation with Alexa 488 anti-rabbit-conjugated secondary antibody (Molecular Probes, Inc., Eugene, USA) diluted 1:900 in blocking buffer. Thereafter, the cover slips were mounted onto glass slides in 10 μl Mowiol (Sigma-Aldrich) and kept in the dark. The cells were viewed on an Olympus/Nikon epifluorescence microscope (Olympus Optical Co. Ltd., Japan), and images were taken with a digital camera (Nikon Imaging Japan Inc., Japan).

The localization of p65/NF-κB was also investigated in cells treated for 72 hours with a 5 μM concentration of the compounds. The cells were fixed, labelled, and imaged as described above.

### Growth curves and sampling for flow cytometric analysis of cell cycle phase distribution

Cells were seeded at the density described above and incubated for 24 hours before the addition of the appropriate compound. The compounds were added to the final concentrations of 1, 2.5, or 5 μM. Cells were sampled every 24 hours for 96 hours for cell counting and then pelleted and fixed in 70% ethanol for flow cytometric evaluation of cell cycle phase distribution.

### Analysis of cell cycle phase distribution

The cells fixed in 70% ethanol were washed once with PBS, and after pelleting, the cells were resuspended in propidium iodide (PI)-nuclear isolation medium (PBS containing 100 μg/ml PI (Sigma-Aldrich), 0.6% NP-40, and 100 μg/ml ribonuclease A (Sigma-Aldrich)) for at least 30 minutes at room temperature or overnight at 4°C. The samples were analysed using a BD Accuri C6 flow cytometer (BD Biosciences, San Jose, CA, USA). The data were analysed using MultiCycle software (Phoenix Flow Systems, San Diego, CA, USA).

### Wound-healing assay

The wound-healing assay was performed as described by Huang *et al*. [15]. The compounds were added to the final concentrations of 1 or 5 μM. The maximal DMSO concentration used was 0.001%.

### Western blot analysis

Cells were seeded at the density described above and incubated for 24 hours before the addition of the compounds. The compounds were added to the final concentrations of 1, 2.5, or 5 μM. After 72 hours of treatment, cells were sampled for cell counting and then pelleted; the dry pellets were stored at -80°C before Western blotting. The pellets were diluted in sample buffer (62.5 mM Tris-HCl (pH 6.8), 20% glycerol, 2% sodium dodecyl sulfate, 5% β-mercaptoethanol, 1% NP-40; 100,000 cells/15 μl). The samples were sonicated twice for 20 seconds each, boiled for 7 minutes and stored at -20°C until further application. Pre-cast polyacrylamide gels (4–12% acrylamide Bis-Tris) were loaded with 15 μl of prepared samples per lane. Gel electrophoresis was performed in an XCell Surelock™ Mini-Cell electrophoresis system (Thermo Fisher Scientific, Stockholm, Sweden) at 150 V for 90 minutes. Then, the gels were blotted onto nitrocellulose membranes using an iBlot Dry Blotting System (Thermo Fisher Scientific). The membranes were blocked in 5% bovine serum albumin in PBS and incubated with primary antibodies against p53 (BD Biosciences, Stockholm, Sweden, 554294), p21 (Santa Cruz, Heidelberg, Germany, sc-6246), cyclin-dependent kinase 2 (CDK2) (Santa Cruz), cyclin D1 (BD Biosciences, 554180), total p65 (Abcam, Cambridge, UK, ab76311), total IκBα (Abcam, ab7217), phosphorylated IκBα (pIκBα, Abcam, ab12135), and β-actin (Abcam). After incubation with peroxidase-conjugated secondary antibodies (Dako, Glostrup, Denmark) at room temperature, the membranes were exposed to enhanced chemiluminescent solution (GE Healthcare, Buckinghamshire, UK) to detect the protein bands. Data were collected and analysed using Quantity One software (Bio-Rad, Hercules, California, USA). The intensities of the bands were determined by densitometric scanning.

### Cell surface markers identified by flow cytometry

The identification of cell surface markers was performed as described by Huang *et al*. [15]. The compounds were added to the final concentrations of 1, 2.5, or 5 μM.

### ALDEFLUOR assay

JIMT-1 cells were seeded at the density described above and incubated for 24 hours before the addition of the compounds. The compounds were added to the final concentrations of 1, 2.5, or 5 μM. The ALDEFLUOR kit (Stem Cell Technologies, Grenoble, France) was used according to the manufacturer’s protocol. The cells were harvested by Accutase treatment and kept on ice; after cell counting, two test tubes (200,000 cells in each tube) with assay buffer were prepared for each sample. One of the test tubes was used as a negative control receiving the specific ALDH inhibitor diethylaminobenzaldehyde (DEAB). Then, the ALDH substrate BODIPY-aminoacetaldehyde was added to both tubes, which were incubated for 45 minutes at 37°C. After incubation, the cells were pelleted by centrifugation, and the pellets were resuspended in 500 μl assay buffer before analysis using the BD Accuri C6 flow cytometer. DEAB-treated cells served as a control to set the ALDH^+^ region for each sample. CFlow software was used to evaluate the data.

### Micronuclei as a measure of DNA double-strand breaks

Cells were seeded onto glass cover slips at the densities described above and incubated for 24 hours before the addition of the compounds. The compounds were added to the final concentrations of 2.5 or 5 μM. After fixation with 3.7% paraformaldehyde in PBS for 15 minutes and subsequent washing in PBS, the cells were permeabilized with PBS containing 1% Tween 20 and 1% FCS. Slides were stained with bisbenzimide (1 μg/μl in PBS) for 2 minutes and finally washed with PBS before mounting. The cells were viewed on an Olympus/Nikon epifluorescence microscope (Olympus Optical Co. Ltd., Japan), and images were taken with a digital camera (Nikon Imaging Japan Inc., Japan). Each slide was imaged at randomly chosen areas using 100x and 40x objectives. The fluorescence of all the images was analysed using ImageJ software.

### Colony formation assay in soft agar

JIMT-1 cells were seeded at the density described above and incubated for 24 hours before the addition of the compounds. The compounds were added to the final concentrations of 1, 2.5, or 5 μM. After 72 hours of incubation with the different treatments, the cells were harvested using Accutase for 10 minutes at 37°C and then kept on ice during cell counting. Mammary epithelial basal medium (CC-4136 kit) (Cambrex, Walkersville, Maryland, USA), supplemented with hydrocortisone, insulin, and EGF (all from the CC-4136 kit) as well as with B27 supplement (Thermo Fisher Scientific), basic fibroblast growth factor (20 ng/ml) (R&D Systems, Minneapolis, MN, USA), penicillin (100 U/ml), and streptomycin (100 μg/ml), was heated to 42°C and mixed with agar to a final concentration of 0.4%. Then, the cell suspension was added to a concentration of 1000 cells/ml, followed by immediate addition of 500 μl of this mixture to the inner wells of hydrophobic 48-well plates. To minimize evaporation, the outer wells were filled with 1 ml PBS. The plates were wrapped in saran wrap and incubated in 5% CO_2_ in humidified air at 37°C for 14 days. The colonies were counted manually using an inverted phase-contrast microscope using a 10x objective.

### Statistics

All data are presented as the mean ± standard deviation (SD) or standard error (SE). Data were analysed by one-way ANOVA followed by Holm-Sidak multiple comparison test. The 95% confidence interval in the multiple comparisons was used.

## Results

### Inhibition of TNF-α-induced NF-κB translocation to the nucleus

**Fig 2:**
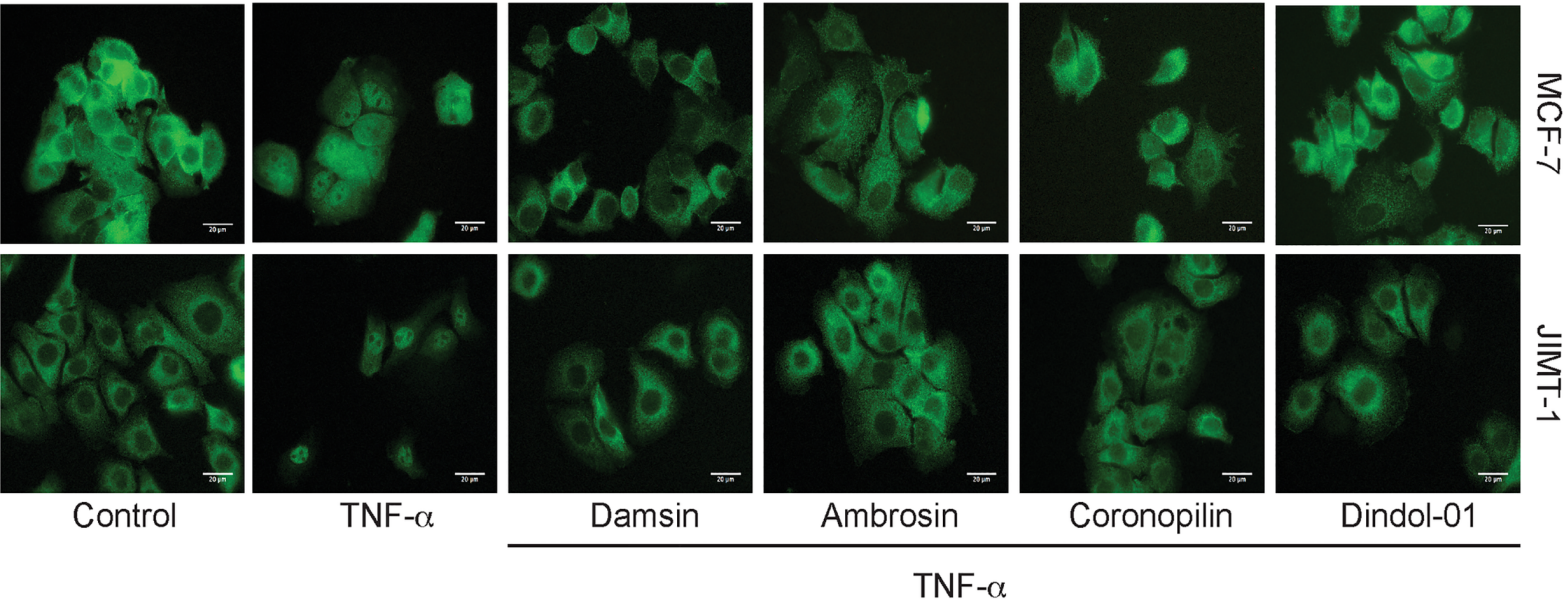
Damsin, ambrosin, coronopilin, and dindol-01 inhibit TNFα-induced p65/NF-κB nuclear translocation. MCF-7 and JIMT-1 cells were treated with 5 μM damsin, ambrosin, coronopilin, or dindol-01 for 60 minutes, and cells were then stimulated with 25 ng/ml TNF-α for 40 minutes. Control-only cells received compound vehicle and TNF-α vehicle. Control-TNF-α cells received compound vehicle for 60 minutes and then TNF-α. The cells were fixed and stained to visualize p65/NF-κB expression (green). Images were taken with a 100x oil immersion objective using an Olympus/Nikon epifluorescence microscope. The scale bars denote 20 μm. Representative images from three independent experiments are shown.

SLs have been shown to exert their effects by interfering with the NF-κB pathway by binding to p65 [14]. Thus, we decided to investigate whether the compounds were indeed active in inhibiting TNF-α-induced NF-κB translocation to the nucleus using immunofluorescence microscopy after labelling with antibodies against p65. Our hypothesis was that if the compounds bound p65, they would inhibit the translocation of the NF-κB complex to the nucleus [16]. In this initial assay to assess activity, we chose the 5 μM concentration based on previous reports of toxicity [17]. TNF-α treatment alone induced the translocation of p65/NF-κB to the nucleus; however, the translocation was inhibited by a 1-hour pretreatment period with a 5 μM concentration of all compounds (Fig 2).

### Dose-response assays

After confirming the compounds were active, we performed dose-response testing using an MTT assay to obtain an overall view of toxicity of the compounds in the breast-derived cell lines. Table 1 shows the IC_50_ values obtained after treating the cells lines for 72 hours with the different compounds (dose-response curves in S1 Fig). Of the four compounds, damsin and ambrosin were the most toxic, with the latter being somewhat more toxic than the former (Table 1). The IC_50_ values of coronopilin and dindol-01 were approximately two times or more as high as those of damsin and ambrosin.

**Table 1:** IC_50_ values for damsin, ambrosin, coronopilin, and dindol-01 in MCF-10A, MCF-7, JIMT-1, and HCC1937 cells.^a^

	MCF-10A	MCF-7	JIMT-1	HCC1937
**Damsin**	8.1 ± 0.4	3.7 ± 0.4	3.3 ± 0.6	6.8 ± 0.4
**Ambrosin**	2.1 ± 0.1	1.7 ± 0.1	1.4 ± 0.1	4.1 ± 0.3
**Coronopilin**	15 ± 0.9	16 ± 6.0	5.5 ± 0.8	16 ± 2.6
**Dindol-01**	37 ± 9.8	16 ± 1.5	16 ± 3.9	15 ± 8.1

^a^ The IC_50_ values were deduced from MTT-based dose-response curves. The data are presented as the mean ± SD from 2–4 experiments with n = 6 in each.

### Inhibition of cell proliferation

**Fig 3:**
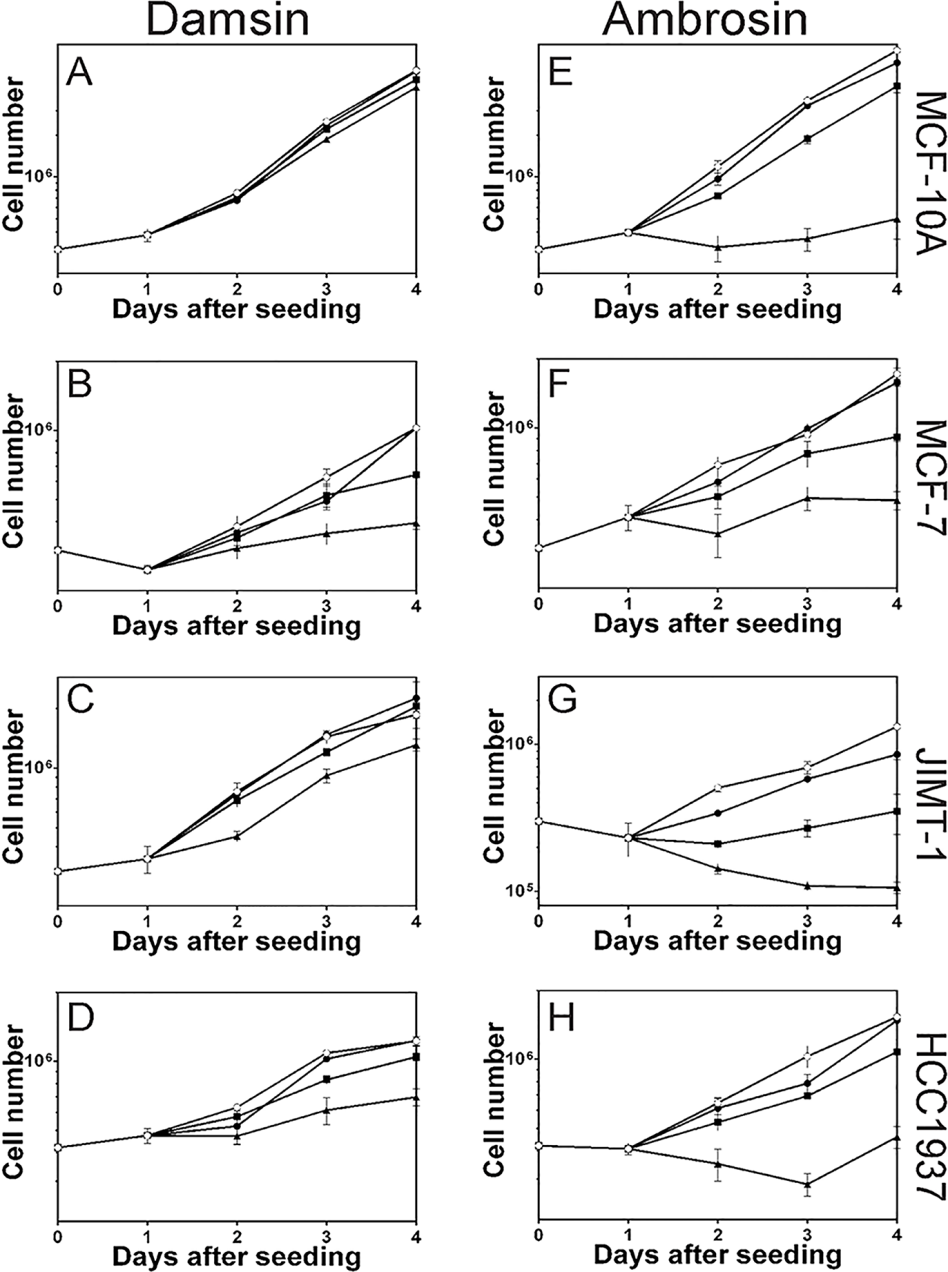
Damsin and ambrosin inhibit cell proliferation of MCF-10A normal-like breast epithelial cells and of MCF-7, JIMT-1, and HCC1937 breast cancer cells in a time- and dose-dependent manner. Cells were seeded day 0, and the compounds were added 1 day after seeding. The cell number was determined by counting in a haemocytometer after cell detachment. ○: control. ●: 1 μM. ■: 2.5 μM. ▲: 5 μM. Data are presented as the mean of 3 experiments, and bars indicate SD.

Based on the dose-response curves, we performed a kinetic investigation of the inhibition of cell proliferation after treatment with the most toxic compounds, damsin and ambrosin, in the four cell lines (Fig 3). Cells were seeded, and 24 hours later, the compounds were added to the final concentrations of 1, 2.5, or 5 μM to cover a range of inhibitory responses in all cell lines; the cell number was determined every 24 hours for a total of 72 hours of treatment. The data were used to construct growth curves, which showed dose- and time-response effects. Ambrosin was more toxic in all cell lines compared to damsin (Fig 3). Treatment with a 5 μM concentration of ambrosin resulted in complete inhibition of cell proliferation already 24 hours after addition of the compound in all cell lines.

### Altered cell cycle phase distribution and induced cell death

**Fig 4:**
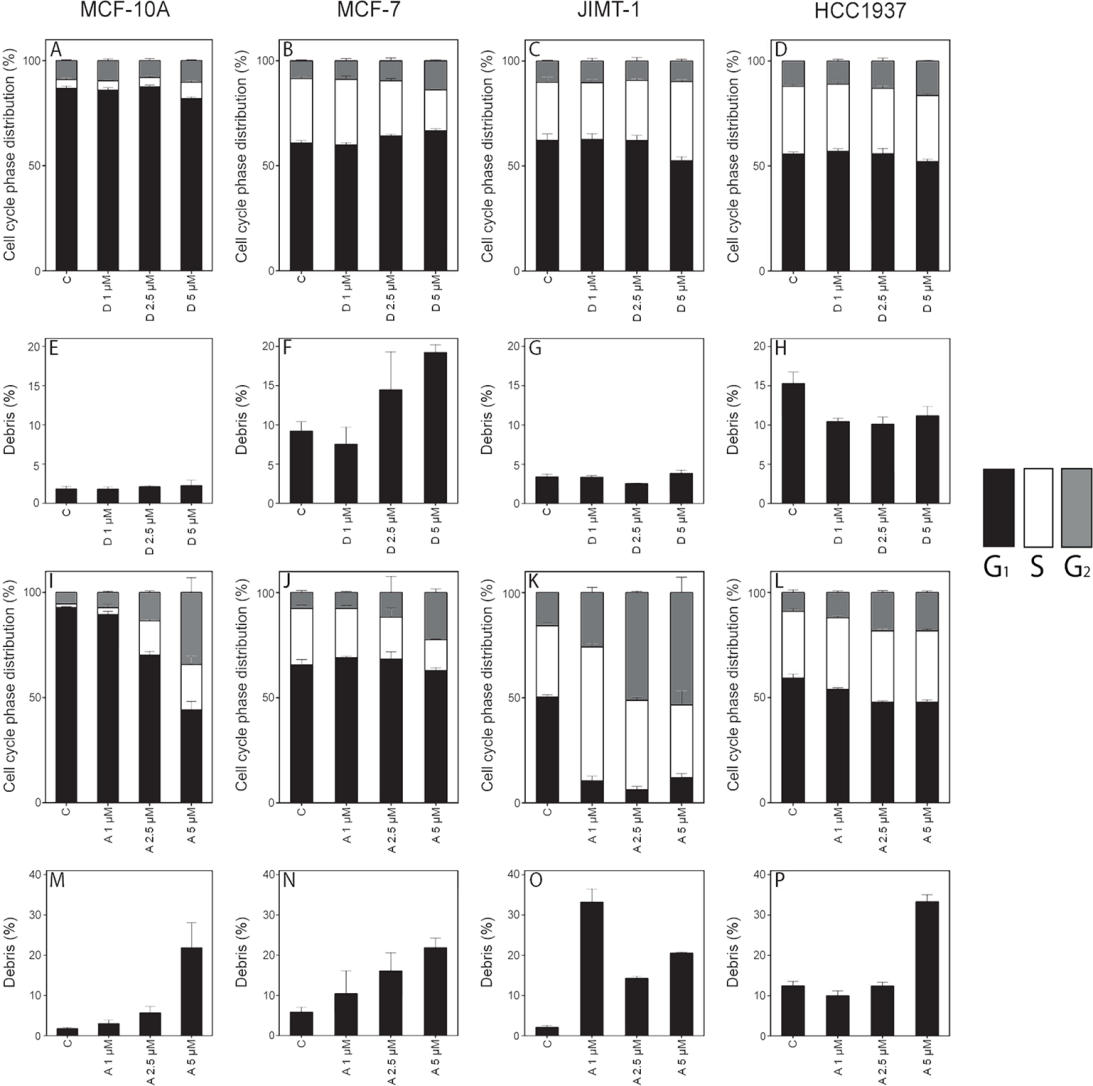
Damsin and ambrosin treatment results in altered cell cycle phase distribution, and ambrosin treatment also induces cell death. The cell cycle phase distribution (panels A-D and I-L) and the % debris (sub-G_1_ region) (panels E-H and M-P) related to cell death was deduced from DNA histograms obtained by flow cytometry of cells treated with damsin or ambrosin for 72 hours. C: control. D: damsin. A: ambrosin. The numbers 1, 2.5, and 5 denote micromolar concentration. Column definition for cell cycle phase distribution: Black, G_1_ phase; White, S phase; Grey, G_2_ phase. Data are presented as the mean ± SD for n = 3.

DNA histograms were used to investigate the effects on the cell cycle phase distribution and if cell death was induced. Thus, after cell counting, the cells were fixed in 70% ethanol and stained with PI, and the cell cycle phase distribution was evaluated by flow cytometry (Fig 4). Treatment with 1 μM and 2.5 μM damsin had no clear effect on the cell cycle phase distribution (Fig 4A–4D), while treatment with 5 μM resulted in increased S and/or G_2_ phases. Ambrosin treatment resulted in an apparent increase in the S and G_2_ phases and at a lower concentration compared to damsin (Fig 4I–4L). The cell cycle phase distribution was more affected in the JIMT-1 cell line than in the other cell lines, especially by ambrosin treatment.

When cells death occurs by either apoptosis or necrosis, the DNA content is reduced by various mechanisms, and the nuclei/parts of nuclei appeared in the sub-G_1_ region (% debris). The different cell lines had different degrees of spontaneous cell death (see the control in Fig 3E–3H and Fig 4M–4P). Damsin treatment did not result in any major difference in the sub-G_1_ region compared to the control (Fig 4E–4H), except in MCF-7 cells at a 5 μM concentration. However, ambrosin treatment increased cell death in all cell lines, especially at a 5 μM concentration (Fig 4M–4P).

### Effect on proteins involved in cell cycle regulation

**Fig 5:**
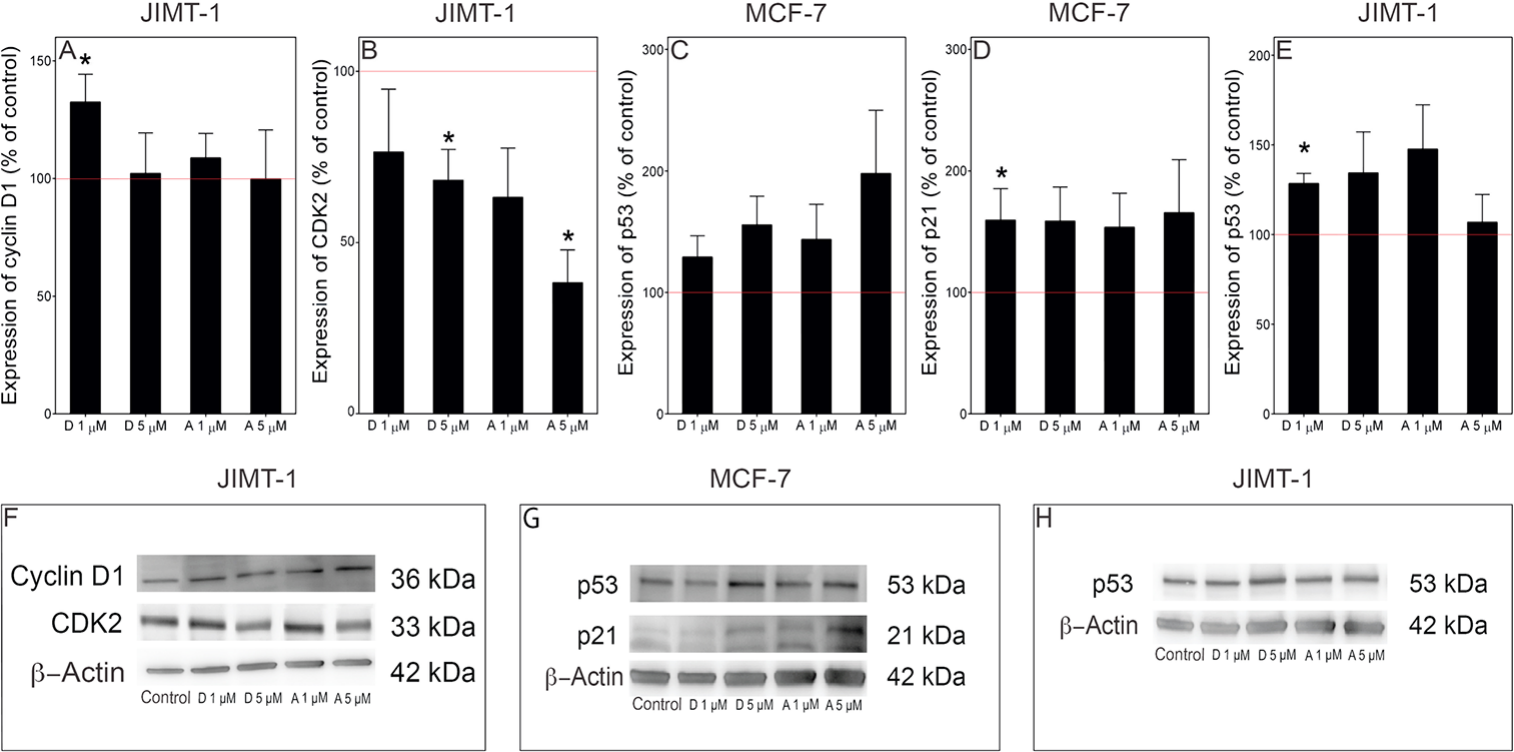
Damsin and ambrosin treatment affect cell cycle regulatory proteins. Panels A-E show the results of densitometric scanning, and panels F-H are representative Western blots used for the scanning. A. Cyclin D1 expression in JIMT-1 cells. B. CDK2 expression in JIMT-1 cells. C. p53 expression in MCF-7 cells. D. p21 expression in MCF-7 cells. E. p53 expression in JIMT-1 cells. Representative blots used for densitometric scanning. F. Cyclin D1 and CDK2 expression in JIMT-1 cells. G. p53 and p21 expression in MCF-7 cells. H. p53 expression in JIMT-1 cells. The cells were treated with 1 (D 1 and A 1) or 5 (D 5 and A 5) μM concentrations of damsin (D) or ambrosin (A) for 72 hours. The data in A-E are expressed in % of control and presented as the mean ± SE for n = 3. D and E. *, p < 0.05 compared to control.

To obtain an understanding of the possible mechanisms involved in the inhibition of cell proliferation, we investigated proteins involved in cell cycle regulation. Cyclin D1 and CDK2, which are important for G_1_ phase progression and S phase transition, respectively, were investigated in JIMT-1 cells. Fig 5 shows that the level of cyclin D1 increased in JIMT-1 cells treated for 72 hours with 1 μM damsin; however, treatment with 5 μM damsin or 1 and 5 μM ambrosin had no effect. On the other hand, CDK2 was downregulated by treatment with both damsin and ambrosin.

The tumour suppressor p53 has a regulatory role in the transition from G_1_ to S phase by upregulating the cyclin-dependent kinase inhibitor p21 in response to DNA damage and oxidative stress [18] and in the regulation of G_2_/M transition [19]. There was a trend towards increased expression of p53 in MCF-7 and JIMT-1 cells treated with damsin and ambrosin (Fig 5A and 5C). In MCF-7 cells containing wild-type p53, there was a trend towards increased p21 (Fig 5B), while p21 was not detected in JIMT-1 cells. This finding is in agreement with the p53 protein in JIMT-1 cells containing a point mutation and thus preventing its action as a transcription factor for p21 [20].

### Formation of micronuclei by treatment with damsin and ambrosin

**Fig 6:**
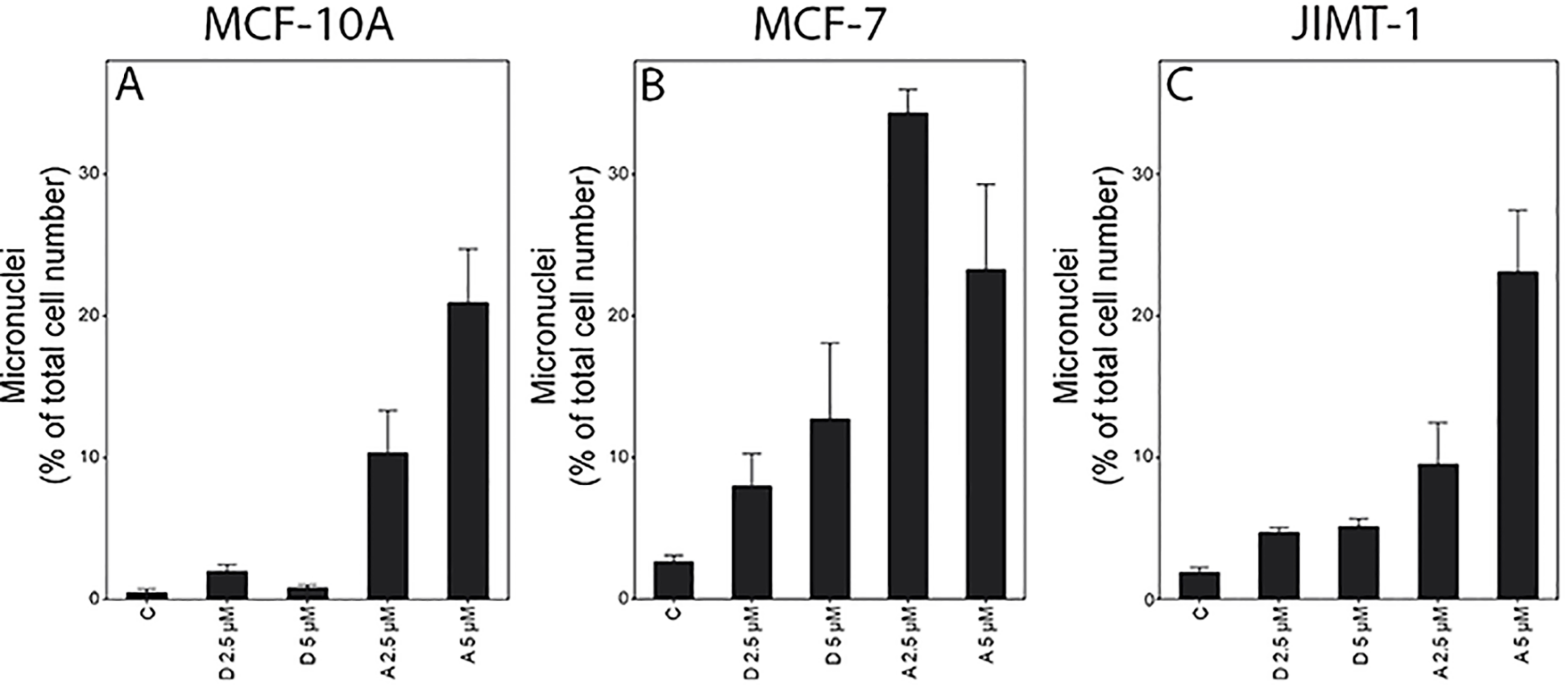
Treatment of MCF-10A (A), MCF-7 (B), and JIMT-1 (C) cells with damsin (D) or ambrosin (A) increases the number of micronuclei. The cells were treated with 1 (D 1 and A 1) or 5 (D 5 and A 5) μM concentrations for 72 hours. The cells were then fixed, and the nuclei were stained with bisbenzimide. Micronuclei were counted in the fluorescence microscopy images. Data are presented as the mean ± SE for n = 6 experiments.

Since there was a trend towards increased p53 expression, and p53 has been connected to DNA damage, we analysed micronuclei in cells after treatment with damsin and ambrosin (Fig 6). In MCF-10A cells, only ambrosin treatment resulted in increased formation of micronuclei (Fig 6A). Both compounds increased the micronuclei in MCF-7 and JIMT-1 cells. However, ambrosin treatment resulted in the formation of more nuclei than damsin treatment (Fig 6). The formation of micronuclei largely reflects the sub-G_1_ region (compare Figs 4 and 6).

### Effects on the protein levels of NF-κB and IκBα

**Fig 7:**
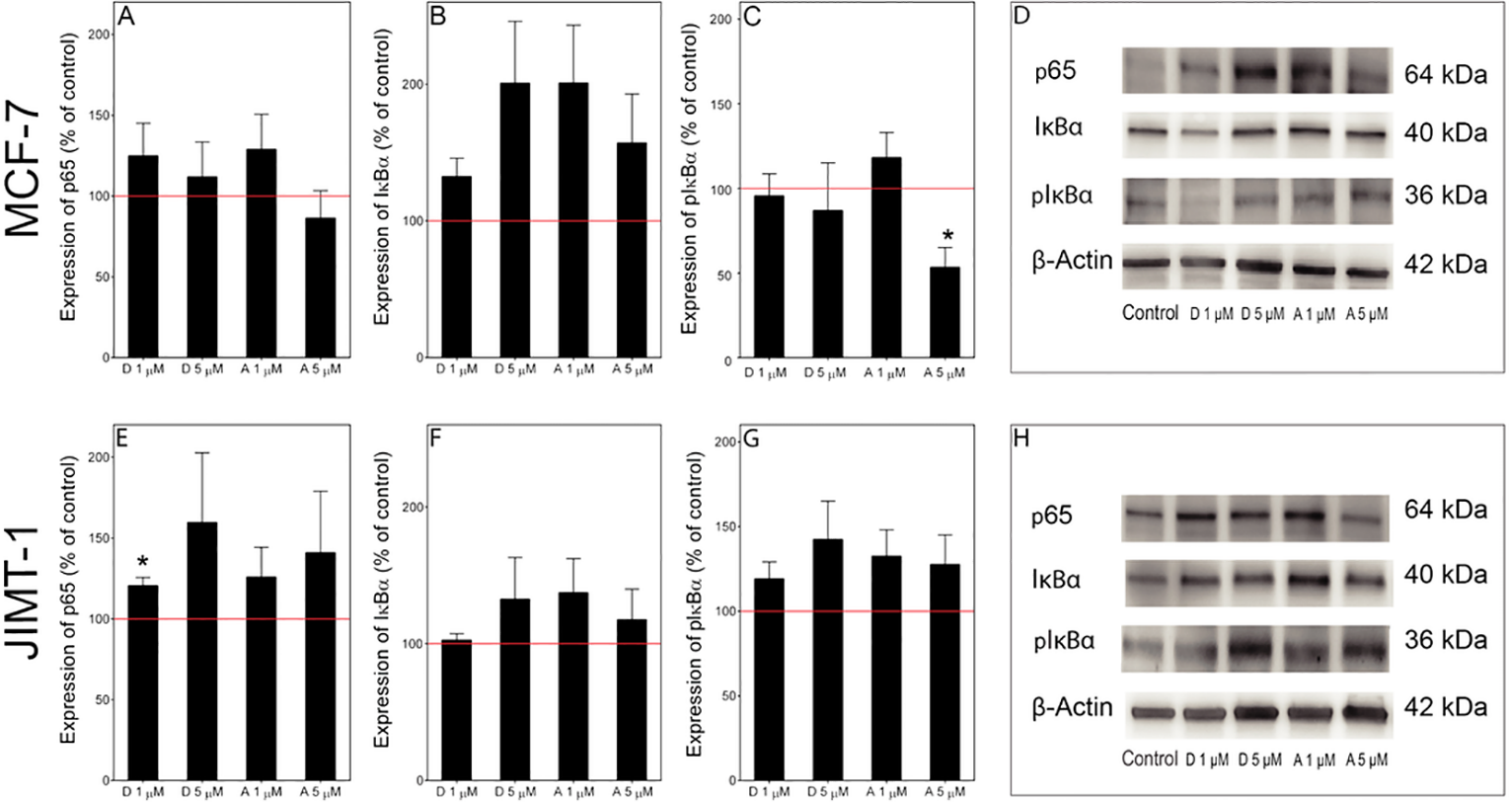
Effect of damsin and ambrosin treatment on the expression of proteins in the NF-κB pathway. Panels A-D. MCF-7 cells. Panels E-H. JIMT-1 cells. Panels D and H show the representative Western blots used to derive the data. D: damsin. A: ambrosin. The numbers 1 and 5 denote micromolar concentration for 72 hours of treatment. The data are expressed in % of control and presented as the mean ± SE for n = 3. *, p < 0.05 compared to control.

After 72 hours of treatment with a 5 μM concentration of the compounds, we could not see any difference in the localization of NF-κB compared to control cells, as evaluated using immunofluorescence microscopy, and NF-κB was found mainly in the cytoplasm (not shown). To obtain a better estimate of the levels of inactive (non-phosphorylated p65/NF-κB) and active (phosphorylated p65/NF-κB), we performed a Western blot analysis of the proteins in MCF-7 and JIMT-1 cells (Fig 7). We also investigated the level of the NF-κB inhibiting protein IκBα (total level) as well as of its phosphorylated form, *i*.*e*., pIκBα.

There was a trend towards increased total level of p65 in MCF7 and JIMT-1 cells after treatment with damsin or ambrosin (Fig 7A and 7E). The phosphorylated form of p65 was not detected in any Western blots. Regarding the NF-κB inhibitor IκBα and its phosphorylated form, pIκBα, there was a trend towards increased total levels in JIMT-1 cells (Fig 7F and 7G). In MCF-7 cells, there was a trend towards increased IκBα in treated cells (Fig 6B), while the level of the phosphorylated form of the protein was unchanged apart from treatment with 5 μM ambrosin, which resulted in a significant decrease (Fig 7C). Together, these data imply that there was a decrease in the proportion of phosphorylated IκBα in cells treated with ambrosin and damsin, implying that the NF-κB heterodimer was partly inhibited from entering the nucleus.

### Effects on cancer stem cells

**Fig 8:**
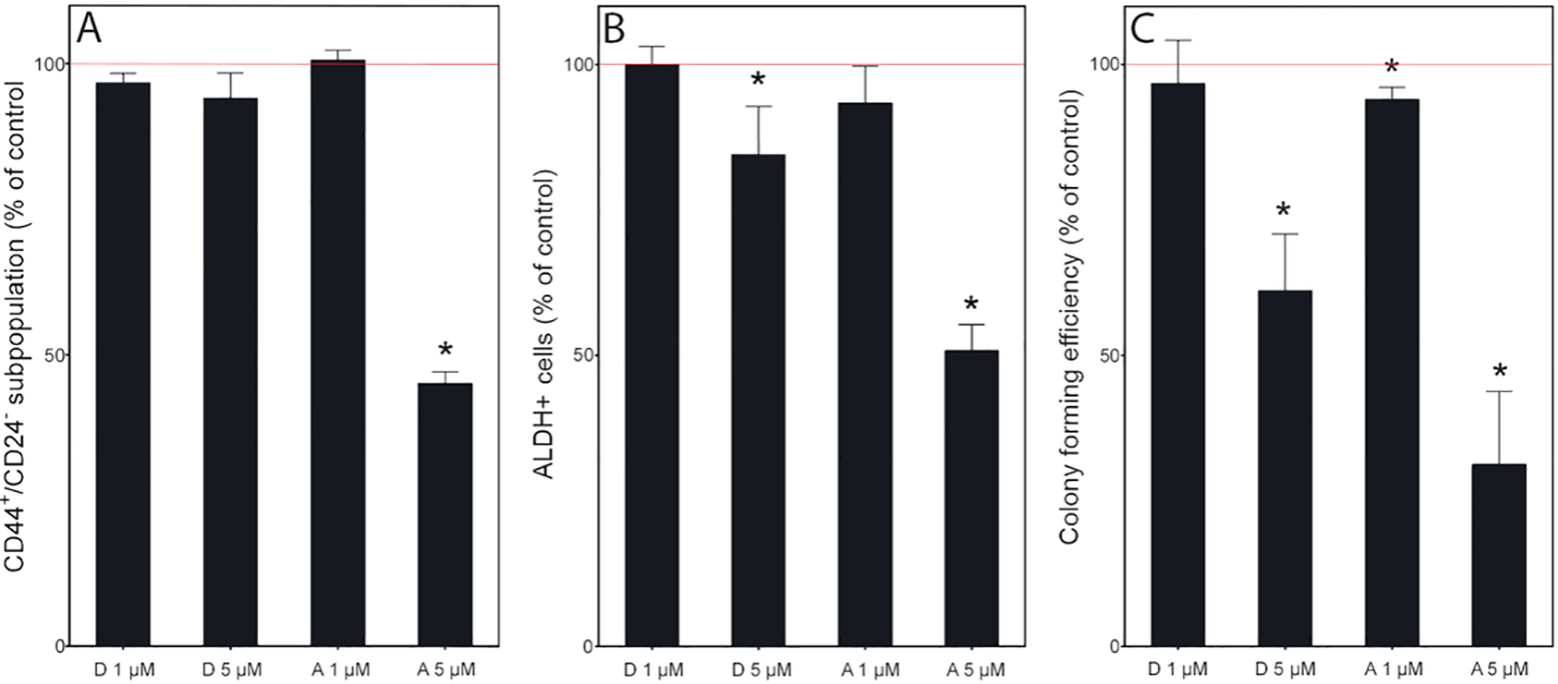
Damsin (D) and ambrosin (A) treatment decreases the CSC population. The cells were treated with 1 (D 1 and A 1) or 5 (D 5 and A 5) μM concentrations for 72 hours. A. The CD44^+^/CD24^-^ population and B. the ALDH^+^ populations were evaluated by flow cytometry. C. Colony forming efficiency was evaluated using a serum-free soft agar assay. JIMT-1 cells were treated for 72 hours and then reseeded at cloning density. The colonies were counted after two weeks of incubation. The numbers denote treatment concentrations (μM). Data are presented as the mean ± SE for n = 3–6. Significantly different compared to control: *: p < 0.05.

CSCs are thought to be at the top of a hierarchical cancer cell organization, and thus, eliminating these cells may result in a higher probability of curing cancer [21]. To investigate CSC specific effects, we used three assays that are presumed to reflect changes in this subpopulation (Fig 8). A high expression of CD44 paired with absent/low expression of CD24 on the cell surface has been used as a CSC marker in breast tumours [22] (Fig 8A). Another marker of breast CSCs is increased ALDH activity [23]. Colony forming efficiency in serum free soft agar is a functional assay, which has been used to investigate the survival of cells with stem cell properties [24] (Fig 8C). Treatment with 5 μM ambrosin decreased the CSC population by more than 50% in all assays (Fig 8), while treatment with 5 μM damsin had a lower effect.

### Inhibition of cell migration

**Fig 9:**
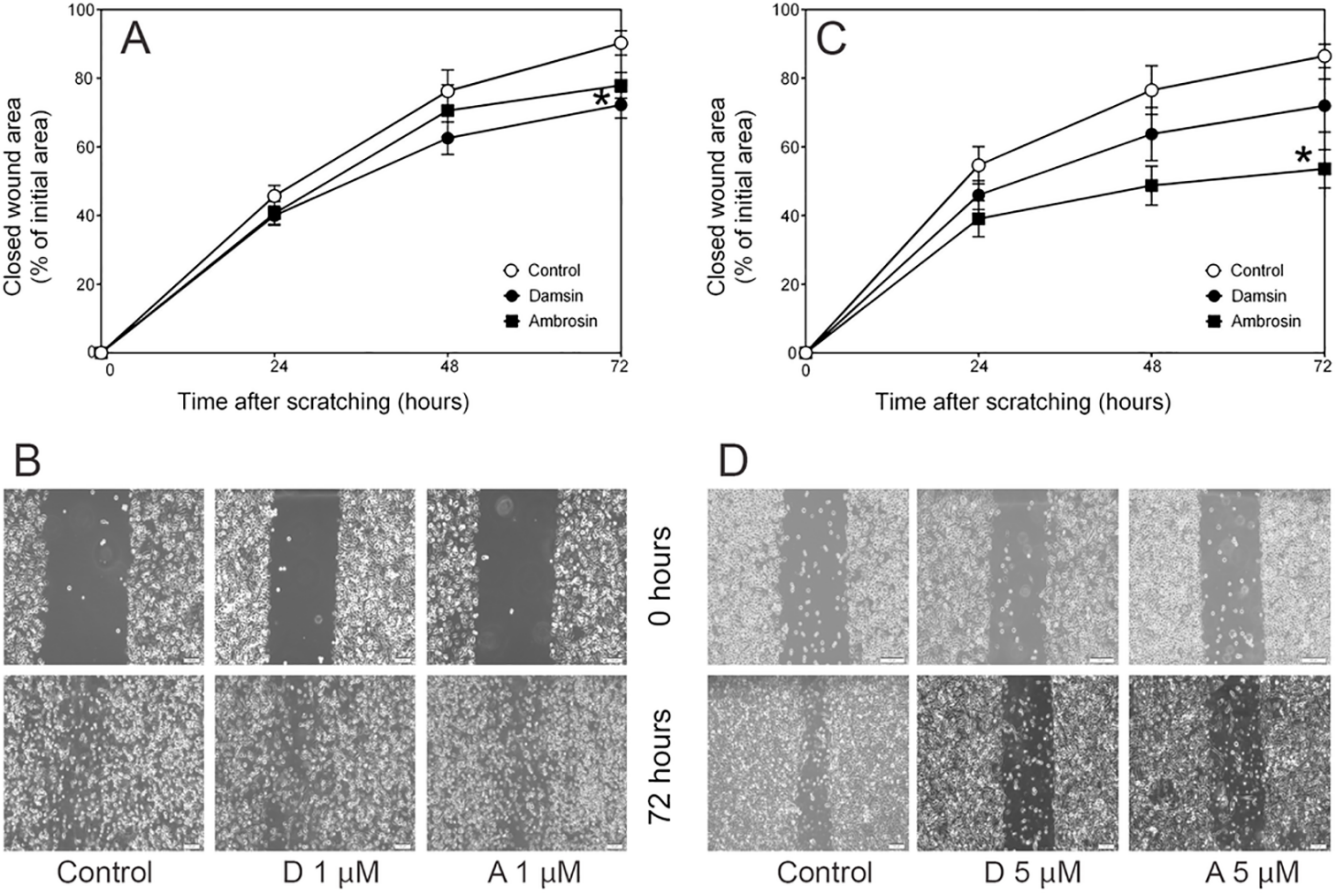
Damsin (D) and ambrosin (A) treatment inhibits wound healing evaluated with a wound-healing assay using JIMT-1 cells. Panels A and B: Treatment with a 1 μM concentration. Panels C and D: Treatment with a 5 μM concentration. Data in A and C are presented as the mean ± SE for n = 6. Panels B and D: Representative phase-contrast images 72 hours after wounding. The bars denote 100 μm. The numbers in B and D denote treatment concentrations (μM). Panel A. Significantly different compared to control: A: *: p < 0.05. Panel C. Ambrosin is significantly different compared to control and damsin: *: p < 0.05.

Cancer cells, specifically CSCs, are endowed with the property of migration, which gives rise to distant metastases [25]. Thus, drugs that can inhibit this process are desirable. The JIMT-1 cell line was used to investigate the effect on cell migration of damsin and ambrosin using a wound-healing assay (Fig 9). Cell migration was inhibited by treatment with either compound.

## Discussion

SLs have been considered in cancer treatment for quite a while because of their chemical properties and biological activities [26]. SLs are, in fact, a large group of compounds that can be categorized into several smaller groups based on the carbocyclic skeleton. The four compounds used in this study belong to the pseudoguanolides group. The toxicities of damsin, ambrosin, and coronopilin have been investigated previously, while dindol-01 is a new SL in biological studies [14]. In our study, we found that damsin and ambrosin were more toxic than coronopilin and dindol-01, and thus, we used these two former compounds for more extensive investigation. However, in a study with CaCo-2 colon cancer cells, coronopilin was shown to exhibit somewhat higher toxicity than damsin, but the concentrations used (25–100 μM) were much higher than in the present study [27]. The toxicity of ambrosin and damsin was investigated in a number of drug-sensitive and drug-resistant cancer cell lines [28]. The IC_50_ values for ambrosin and damsin were similar in the previous study, and in general, higher IC_50_ values were obtained compared to those in the present study. There are many causes for differences in IC_50_ values of the same compounds between different studies, and one obvious difference is the cell lines used and culture conditions.

To the best of our knowledge, no extensive studies of the effects of these compounds on cell proliferation kinetics have been reported. Here, we show the kinetics of inhibition of cell proliferation in growth curves. As expected, increasing the concentrations of a compound showed increasing effects on cell proliferation kinetics, and this trend was clear for the doses chosen for ambrosin in all cell lines. However, regarding damsin, this trend was clear for the breast cancer cell lines, while it was less obvious for the MCF-10A cells. Of course, it is beneficial to find that normal cells are not affected at a dose where cancer cells are affected; however, general conclusions regarding differences between cancer cells and normal cells cannot be drawn on the basis of data from one normal-like cell line. In addition, ambrosin did not display this difference in inhibition of cell proliferation between MCF-10A and the breast cancer cell lines.

When cell proliferation is inhibited by treatment with toxic compounds, it may result in changes in cell cycle phase distribution as a reflection of which cell cycle phases are affected [29]. Damsin and ambrosin treatment resulted in an increased fraction of cells in S and G_2_ phases, which suggests the induction of DNA damage. That DNA damage was indeed induced as was demonstrated by an increased number of micronuclei and a tendency to increased p53, a DNA damage response protein. A study using the SLs costunolide and dehydrocostus lactone also showed the induction of DNA damage, which arrested the cells at the G_2_/M interface [30]. That study corroborated the block with decreased expression of CDK2, which we also found. In the study mentioned above comparing the toxicity of damsin and ambrosin in drug-sensitive and drug-resistant cell lines, the cells were found to express mutated p53 and were sensitive to damsin but not to ambrosin [19]. This finding is in contrast to our study where we found that all cell lines were more sensitive to ambrosin independent of p53 status. MCF-10A and MCF-7 cells have wild-type p53, while JIMT-1 and HCC1937 have mutated p53.

SLs have been shown to inhibit the NF-κB pathway. NF-κB is a transcription factor that is regulated by its localization [31]. When inactive, NF-κB is sequestered in the cytoplasm by association with IκB proteins; however, when activated by various signals, NF-κB is released from the inhibitor and translocates to the nucleus to activate gene transcription. TNF-α is an activator of NF-κB, and we used this cytokine to show that all four SLs were indeed active at a 5 μM concentration in acute experiments. Western blot analysis of proteins involved in this pathway showed only slight changes after 72 hours of treatment. However, NF-κB has been shown to be important for CSCs [32], and we found a reduction of the CSC subpopulation of the JIMT-1 cell line using three different assays. In addition, we found an inhibition of cell migration, which can also be a result of a reduced CSC population, as CSCs potentially possess mesenchymal traits and contribute to cancer metastases [33]. Thus, the most important information obtained from this study is the effect on the CSC population and on cell migration. CSCs have been identified in almost all solid tumours and have been suggested to be responsible for cancer treatment failure, as CSCs display resistance to surgery, radiation, and chemotherapy. Thus, CSCs are an obvious target in the search for more efficient anti-cancer drugs that alone or in various combinations can eradicate all cancer cells of a tumour. Our data suggest that SLs should be further exploited regarding anti-CSC activity through rational design.

## Supporting information

S1 Fig Dose response curves of damsin, ambrosin, coronopolin, and dindol-01 obtained when treating the MCF-10A normal-like breast epithelial cell line and the MCF-7, JIMT-1, and HCC1937 breast cancer cell lines. (TIF)

S2 Fig Original data for Fig 3. (PDF)

S3 Fig Original data for Fig 4. (PDF)

S4 Fig Original data for Fig 5. (PDF)

S5 Fig Original data for Fig 6. (PDF)

S6 Fig Original data for Fig 7. (PDF)

S7 Fig Original data for Fig 8. (PDF)

S8 Fig Original data for Fig 9. (PDF)
