# Supplementary figures and images for: Anti-cancer stem cell activity of a sesquiterpene lactone isolated from Ambrosia arborescens and of a synthetic derivative
Source: PLoS One. 2017 Sep 1;12(9):e0184304. doi: 10.1371/journal.pone.0184304 (PMC5581169; doi:10.1371/journal.pone.0184304)

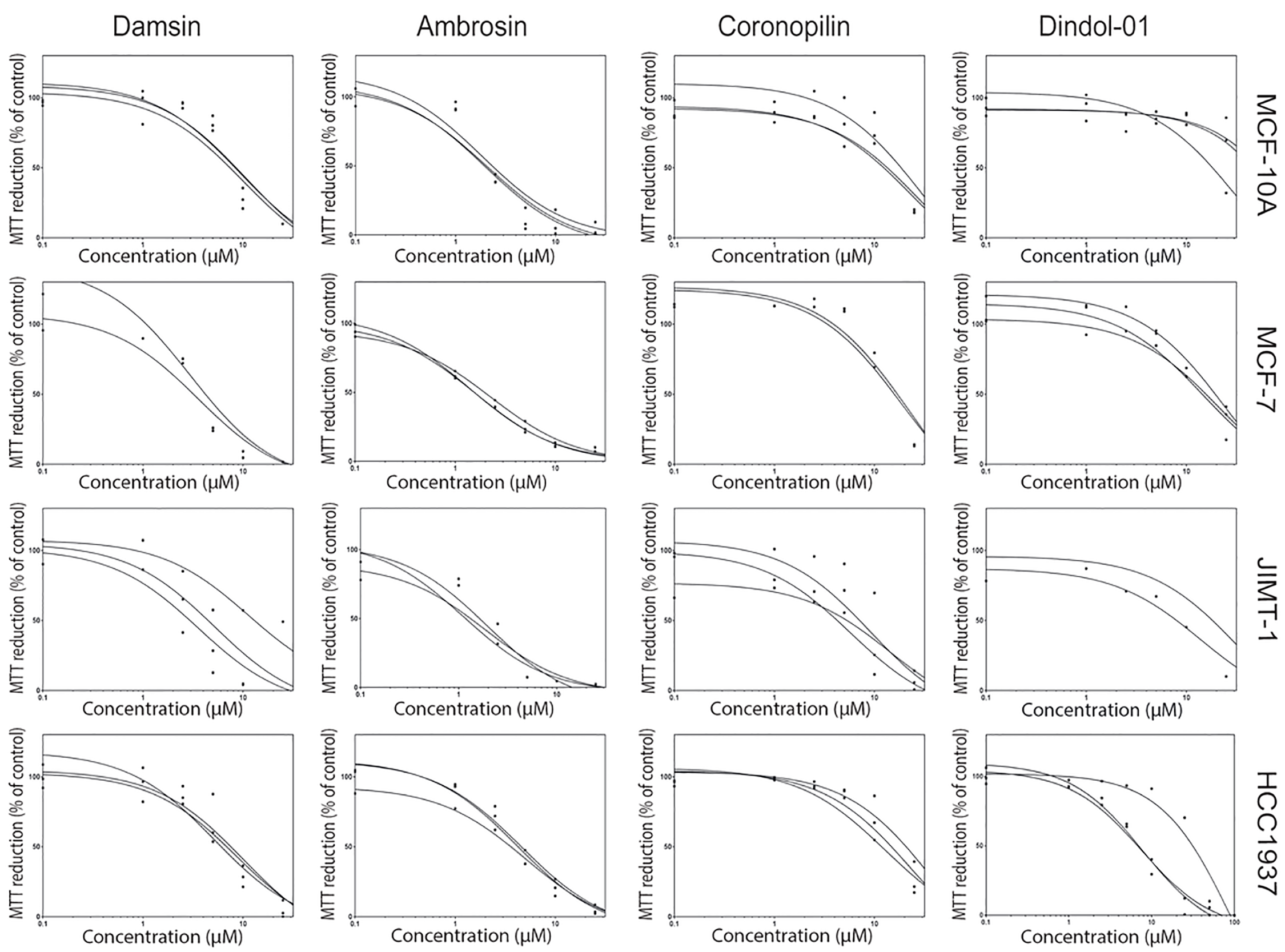

Supplement: S1 Fig — (TIF) [file pone.0184304.s001.tif]
