# Supplementary material for: Anti-cancer stem cell activity of a sesquiterpene lactone isolated from Ambrosia arborescens and of a synthetic derivative
Source: PLoS One. 2017 Sep 1;12(9):e0184304. doi: 10.1371/journal.pone.0184304 (PMC5581169; doi:10.1371/journal.pone.0184304)

MCF-10A Damsin

Sample #	Treatment	Day	Cells/ml	Cells/Petri dish	Mean value
Seeding	Ctrl	-1		210000	
Seeding	Ctrl	-1		210000	
Seeding	Ctrl	-1		210000	
0 hrs	Ctrl	0	51100	357700	
0 hrs	Ctrl	0	51100	357700	381033
0 hrs	Ctrl	0	61100	427700	
24 hrs	Ctrl	1	106700	746900	
24 hrs	Ctrl	1	113300	793100	767433
24 hrs	Ctrl	1	108900	762300	
24 hrs	1 µM	1	94400	660800	
24 hrs	1 µM	1	98900	692300	676667
24 hrs	1 µM	1	96700	676900	
24 hrs	2.5 µM	1	106700	746900	
24 hrs	2.5 µM	1	101100	707700	707933
24 hrs	2.5 µM	1	95600	669200	
24 hrs	5 µM	1	98900	692300	
24 hrs	5 µM	1	104400	730800	697433
24 hrs	5 µM	1	95600	669200	
48 hrs	Ctrl	2	364400	2550800	
48 hrs	Ctrl	2	361100	2527700	2501800
48 hrs	Ctrl	2	346700	2426900	
48 hrs	1 µM	2	341100	2387700	
48 hrs	1 µM	2	330000	2310000	2346167
48 hrs	1 µM	2	334400	2340800	
48 hrs	2.5 µM	2	307800	2154600	
48 hrs	2.5 µM	2	311100	2177700	2211533
48 hrs	2.5 µM	2	328900	2302300	
48 hrs	5 µM	2	273300	1913100	
48 hrs	5 µM	2	263300	1843100	1866433
48 hrs	5 µM	2	263300	1843100	
72 hrs	Ctrl	3	810000	5670000	
72 hrs	Ctrl	3	813300	5693100	5856667
72 hrs	Ctrl	3	886700	6206900	
72 hrs	1 µM	3	783300	5483100	
72 hrs	1 µM	3	820000	5740000	5778733
72 hrs	1 µM	3	873300	6113100	
72 hrs	2.5 µM	3	716700	5016900	
72 hrs	2.5 µM	3	713300	4993100	5032300
72 hrs	2.5 µM	3	726700	5086900	
72 hrs	5 µM	3	612500	4287500	
72 hrs	5 µM	3	635000	4445000	4421667
72 hrs	5 µM	3	647500	4532500	

Day	# of cells for			
	Ctrl	1 µM	2.5 µm	5 µM
0	300000	300000	300000	300000
1	381033	381033	381033	381033
2	767433	676667	707933	697433
3	2501800	2346167	2211533	1866433
4	5856667	5778733	5032300	4421667

SD				
Day	Ctrl	1 µM	2.5 µm	5 µM
0	0	0	0	0
1	40415	40415	40415	40415
2	23524	15751	38851	31119
3	65886	39127	79450	40415
4	303531	316781	48759	124155

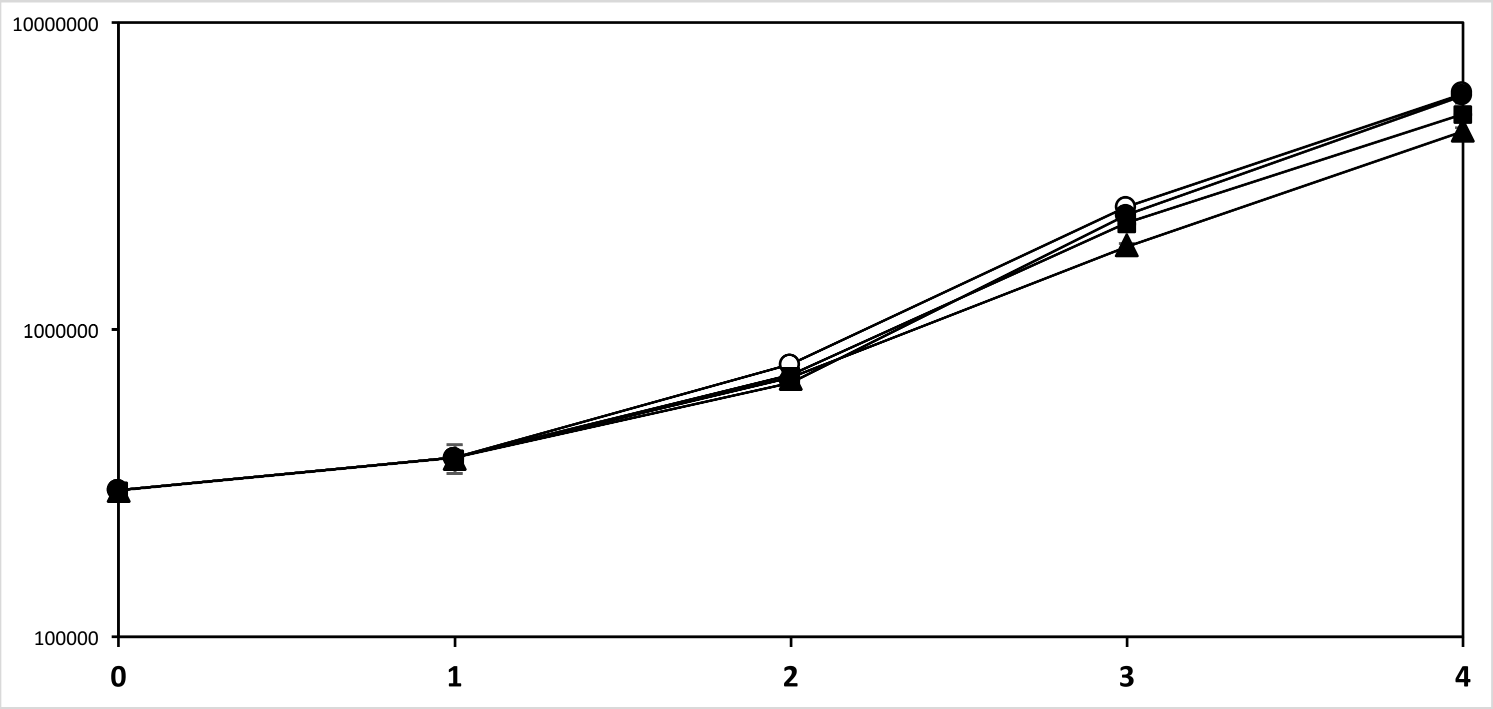

MCF-10A Ambrosin

Sample #	Treatment	Day	Cells/ml	Cells/Petri dish	Mean value
Seeding	Ctrl	-1		300000	
Seeding	Ctrl	-1		300000	
Seeding	Ctrl	-1		300000	
0 hrs	Ctrl	0	54400	380800	
0 hrs	Ctrl	0	57800	404600	396667
0 hrs	Ctrl	0	57800	404600	
24 hrs	Ctrl	1	157000	1099000	
24 hrs	Ctrl	1	161000	1127000	1185333
24 hrs	Ctrl	1	190000	1330000	
24 hrs	1 µM	1	151000	1057000	
24 hrs	1 µM	1	124000	868000	968333
24 hrs	1 µM	1	140000	980000	
24 hrs	2.5 µM	1	104000	728000	
24 hrs	2.5 µM	1	107000	749000	730333
24 hrs	2.5 µM	1	102000	714000	
24 hrs	5 µM	1	43300	303100	
24 hrs	5 µM	1	54400	380800	311033
24 hrs	5 µM	1	35600	249200	
48 hrs	Ctrl	2	466000	3262000	
48 hrs	Ctrl	2	517500	3622500	3554833
48 hrs	Ctrl	2	540000	3780000	
48 hrs	1 µM	2	469000	3283000	
48 hrs	1 µM	2	468000	3276000	3259667
48 hrs	1 µM	2	460000	3220000	
48 hrs	2.5 µM	2	289000	2023000	
48 hrs	2.5 µM	2	246000	1722000	1883000
48 hrs	2.5 µM	2	272000	1904000	
48 hrs	5 µM	2	52200	365400	
48 hrs	5 µM	2	60000	420000	357700
48 hrs	5 µM	2	41100	287700	
72 hrs	Ctrl	3	1180000	8260000	
72 hrs	Ctrl	3	1086700	7606900	8165967
72 hrs	Ctrl	3	1233000	8631000	
72 hrs	1 µM	3	933000	6531000	
72 hrs	1 µM	3	920000	6440000	6640667
72 hrs	1 µM	3	993000	6951000	
72 hrs	2.5 µM	3	710000	4970000	
72 hrs	2.5 µM	3	653000	4571000	4516167
72 hrs	2.5 µM	3	572500	4007500	
72 hrs	5 µM	3	66700	466900	
72 hrs	5 µM	3	53300	373100	497700
72 hrs	5 µM	3	93300	653100	

Day	# of cells for			
	Ctrl	1 µM	2.5 µm	5 µM
0	300000	300000	300000	300000
1	396667	396667	396667	396667
2	1185333	968333	730333	311033
3	3554833	3259667	1883000	357700
4	8165967	6640667	4516167	497700

SD				
Day	Ctrl	1 µM	2.5 µm	5 µM
0	0	0	0	0
1	13741	13741	13741	13741
2	126065	95039	17616	66158
3	265547	34530	151595	66485
4	518485	272581	483587	142518

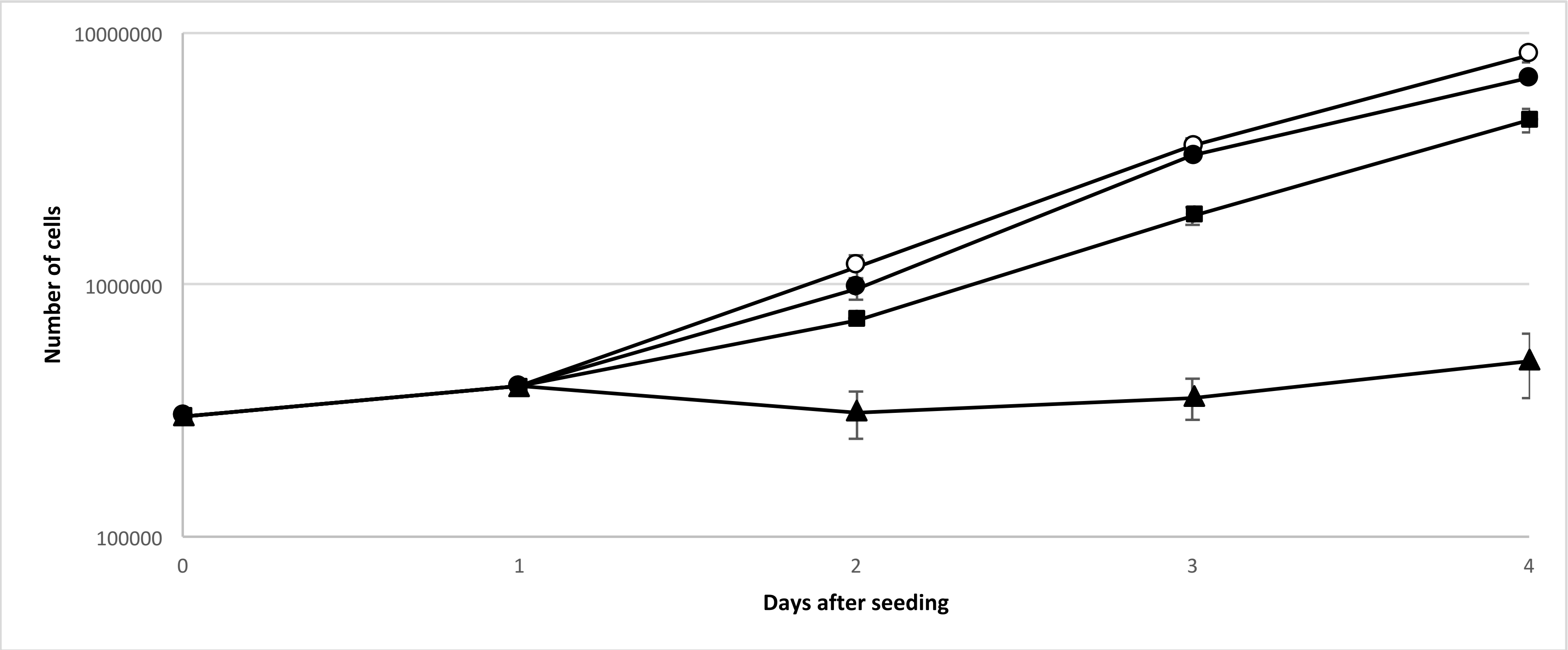

MCF-7 Damsin

Sample #	Treatment	Day	Cells/ml	Cells/Petri dish	Mean value
Seeding	Ctrl	-1		450000	
Seeding	Ctrl	-1		450000	
Seeding	Ctrl	-1		450000	
0 hrs	Ctrl	0	33300	233100	
0 hrs	Ctrl	0	36700	256900	246307
0 hrs	Ctrl	0	35560	248920	
24 hrs	Ctrl	1	61100	427700	
24 hrs	Ctrl	1	48890	342230	381080
24 hrs	Ctrl	1	53330	373310	
24 hrs	1 µM	1	53330	373310	
24 hrs	1 µM	1	50000	350000	357770
24 hrs	1 µM	1	50000	350000	
24 hrs	2.5 µM	1	50000	350000	
24 hrs	2.5 µM	1	44400	310800	339523
24 hrs	2.5 µM	1	51110	357770	
24 hrs	5 µM	1	41100	287700	
24 hrs	5 µM	1	41100	287700	305877
24 hrs	5 µM	1	48890	342230	
48 hrs	Ctrl	2	97800	684600	
48 hrs	Ctrl	2	86700	606900	624867
48 hrs	Ctrl	2	83300	583100	
48 hrs	1 µM	2	76700	536900	
48 hrs	1 µM	2	65600	459200	490233
48 hrs	1 µM	2	67800	474600	
48 hrs	2.5 µM	2	70000	490000	
48 hrs	2.5 µM	2	84400	590800	521033
48 hrs	2.5 µM	2	68900	482300	
48 hrs	5 µM	2	44400	310800	
48 hrs	5 µM	2	53300	373100	354900
48 hrs	5 µM	2	54400	380800	
72 hrs	Ctrl	3	148900	1042300	
72 hrs	Ctrl	3	146700	1026900	1024100
72 hrs	Ctrl	3	143300	1003100	
72 hrs	1 µM	3	146700	1026900	
72 hrs	1 µM	3	144400	1010800	1024100
72 hrs	1 µM	3	147800	1034600	
72 hrs	2.5 µM	3	93300	653100	
72 hrs	2.5 µM	3	91110	637770	640290
72 hrs	2.5 µM	3	90000	630000	
72 hrs	5 µM	3	55600	389200	
72 hrs	5 µM	3	60000	420000	394100
72 hrs	5 µM	3	53300	373100	

Day	# of cells for			
	Ctrl	1 µM	2.5 µm	5 µM
0	300000	300000	300000	300000
1	246307	246307	246307	246307
2	381080	357770	339523	305877
3	624867	490233	521033	354900
4	1024100	1024100	640290	394100

SD				
Day	Ctrl	1 µM	2.5 µm	5 µM
0	0	0	0	0
1	12113	12113	12113	12113
2	43262	13458	25177	31483
3	53082	41142	60542	38385
4	19749	12145	11754	23831

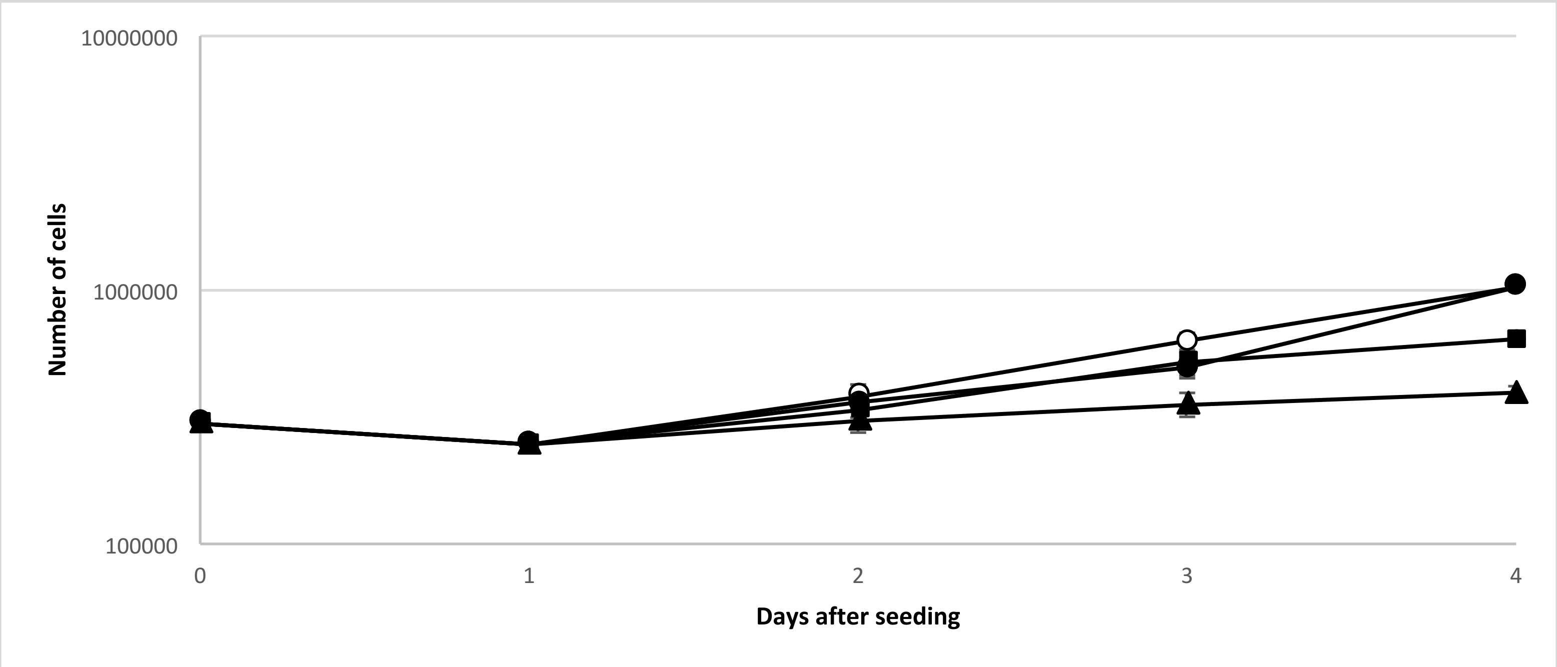

MCF-7 Ambrosin

Sample #	Treatment	Day	Cells/ml	Cells/Petri dish	Mean value
Seeding	Ctrl	-1		450000	
Seeding	Ctrl	-1		450000	
Seeding	Ctrl	-1		450000	
0 hrs	Ctrl	0	124400	870800	
0 hrs	Ctrl	0	115600	809200	816667
0 hrs	Ctrl	0	110000	770000	
24 hrs	Ctrl	1	188900	1322300	
24 hrs	Ctrl	1	205600	1439200	1379467
24 hrs	Ctrl	1	196700	1376900	
24 hrs	1 µM	1	172200	1205400	
24 hrs	1 µM	1	170000	1190000	1164100
24 hrs	1 µM	1	156700	1096900	
24 hrs	2.5 µM	1	136700	956900	
24 hrs	2.5 µM	1	141100	987700	1003333
24 hrs	2.5 µM	1	152200	1065400	
24 hrs	5 µM	1	92200	645400	
24 hrs	5 µM	1	111000	777000	691833
24 hrs	5 µM	1	93300	653100	
48 hrs	Ctrl	2	263300	1843100	
48 hrs	Ctrl	2	267800	1874600	1874367
48 hrs	Ctrl	2	272200	1905400	
48 hrs	1 µM	2	288900	2022300	
48 hrs	1 µM	2	287800	2014600	1988700
48 hrs	1 µM	2	275600	1929200	
48 hrs	2.5 µM	2	235600	1649200	
48 hrs	2.5 µM	2	206700	1446900	1547933
48 hrs	2.5 µM	2	221100	1547700	
48 hrs	5 µM	2	141100	987700	
48 hrs	5 µM	2	150000	1050000	990267
48 hrs	5 µM	2	133300	933100	
72 hrs	Ctrl	3	476000	3332000	
72 hrs	Ctrl	3	494000	3458000	3447500
72 hrs	Ctrl	3	507500	3552500	
72 hrs	1 µM	3	458000	3206000	
72 hrs	1 µM	3	442000	3094000	3154667
72 hrs	1 µM	3	452000	3164000	
72 hrs	2.5 µM	3	255600	1789200	
72 hrs	2.5 µM	3	267800	1874600	1830500
72 hrs	2.5 µM	3	261100	1827700	
72 hrs	5 µM	3	143300	1003100	
72 hrs	5 µM	3	140000	980000	966933
72 hrs	5 µM	3	131100	917700	

Day	# of cells for			
	Ctrl	1 µM	2.5 µm	5 µM
0	450000	450000	450000	450000
1	816667	816667	816667	816667
2	1379467	1164100	1003333	691833
3	1874367	1988700	1547933	990267
4	3447500	3154667	1547933	966933

SD				
Day	Ctrl	1 µM	2.5 µm	5 µM
0	0	0	0	0
1	50813	50813	50813	50813
2	58492	58704	55914	73857
3	31151	51672	101150	58492
4	110624	56580	42769	44174

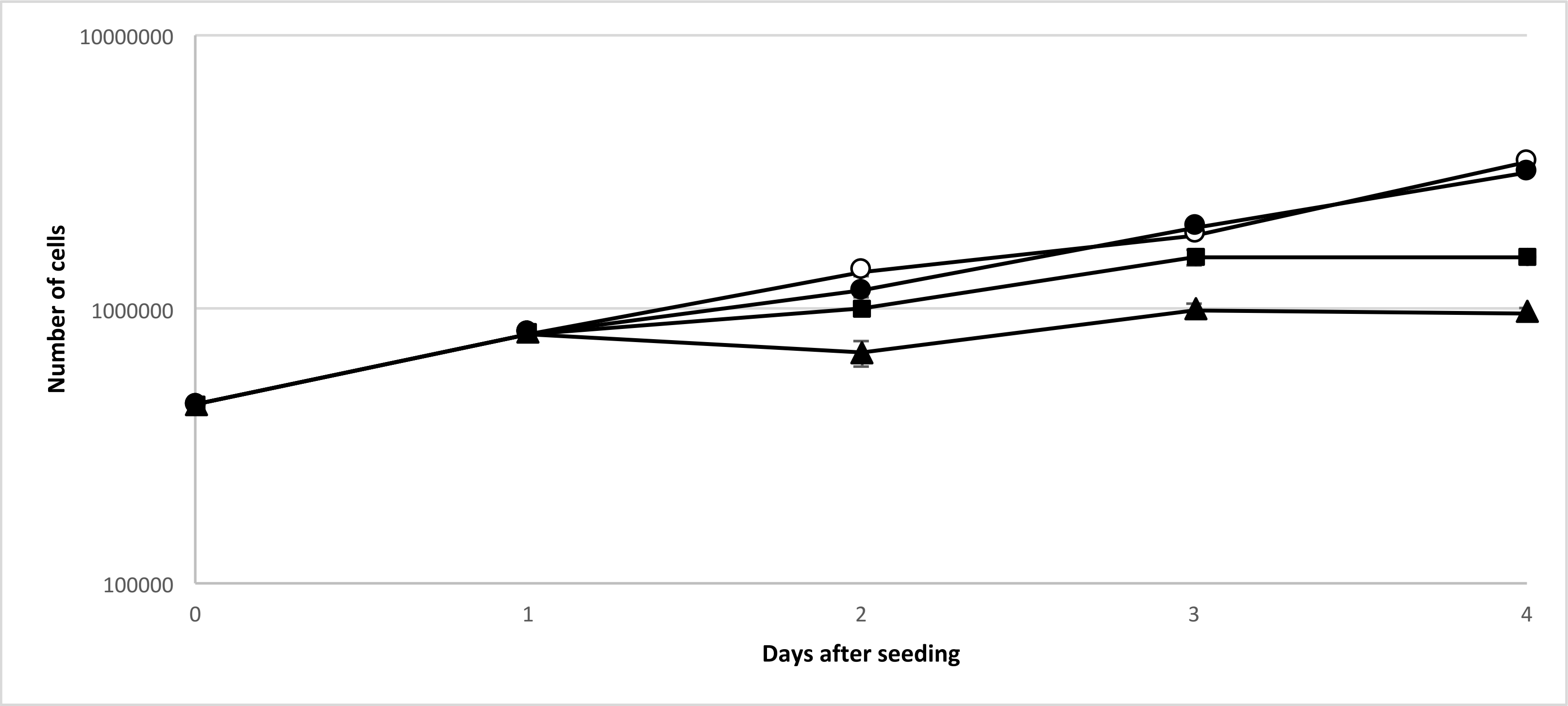

JIMT-1 Damsin

Sample #	Treatment	Day	Cells/ml	Cells/Petri dish	Mean value
Seeding	Ctrl	-1		300000	
Seeding	Ctrl	-1		300000	
Seeding	Ctrl	-1		300000	
0 hrs	Ctrl	0	42200	295400	
0 hrs	Ctrl	0	48900	342300	347433,33
0 hrs	Ctrl	0	57800	404600	
24 hrs	Ctrl	1	54400	761600	
24 hrs	Ctrl	1	60000	840000	762020
24 hrs	Ctrl	1	97780	684460	
24 hrs	1 µM	1	108000	756000	
24 hrs	1 µM	1	107000	749000	739667
24 hrs	1 µM	1	102000	714000	
24 hrs	2.5 µM	1	87800	702400	
24 hrs	2.5 µM	1	104000	728000	686800
24 hrs	2.5 µM	1	90000	630000	
24 hrs	5 µM	1	68900	482300	
24 hrs	5 µM	1	62200	435400	451033
24 hrs	5 µM	1	62200	435400	
48 hrs	Ctrl	2	210000	1470000	
48 hrs	Ctrl	2	189000	1323000	1446667
48 hrs	Ctrl	2	221000	1547000	
48 hrs	1 µM	2	210000	1470000	
48 hrs	1 µM	2	221000	1547000	1479333
48 hrs	1 µM	2	203000	1421000	
48 hrs	2.5 µM	2	143000	1144000	
48 hrs	2.5 µM	2	178000	1246000	1207333
48 hrs	2.5 µM	2	176000	1232000	
48 hrs	5 µM	2	123000	861000	
48 hrs	5 µM	2	127000	889000	917000
48 hrs	5 µM	2	143000	1001000	
72 hrs	Ctrl	3	311000	2177000	
72 hrs	Ctrl	3	246000	1722000	1864333
72 hrs	Ctrl	3	242000	1694000	
72 hrs	1 µM	3	401000	2807000	
72 hrs	1 µM	3	291000	2037000	2263333
72 hrs	1 µM	3	278000	1946000	
72 hrs	2.5 µM	3	282000	1974000	
72 hrs	2.5 µM	3	250000	2000000	2052667
72 hrs	2.5 µM	3	273000	2184000	
72 hrs	5 µM	3	174000	1218000	
72 hrs	5 µM	3	200000	1400000	1311333
72 hrs	5 µM	3	188000	1316000	

Day	# of cells for			
	Ctrl	1 µM	2.5 µm	5 µM
0	300000	300000	300000	300000
1	347433	347433	347433	347433
2	762020	739667	686800	451033
3	1446667	1479333	1207333	917000
4	1864333	2263333	2052667	1311333

SD				
Day	Ctrl	1 µM	2.5 µm	5 µM
0	0	0	0	0
1	54781	54781	54781	54781
2	77771	22502	50828	27078
3	113808	63516	55293	74081
4	271139	473023	114479	91090

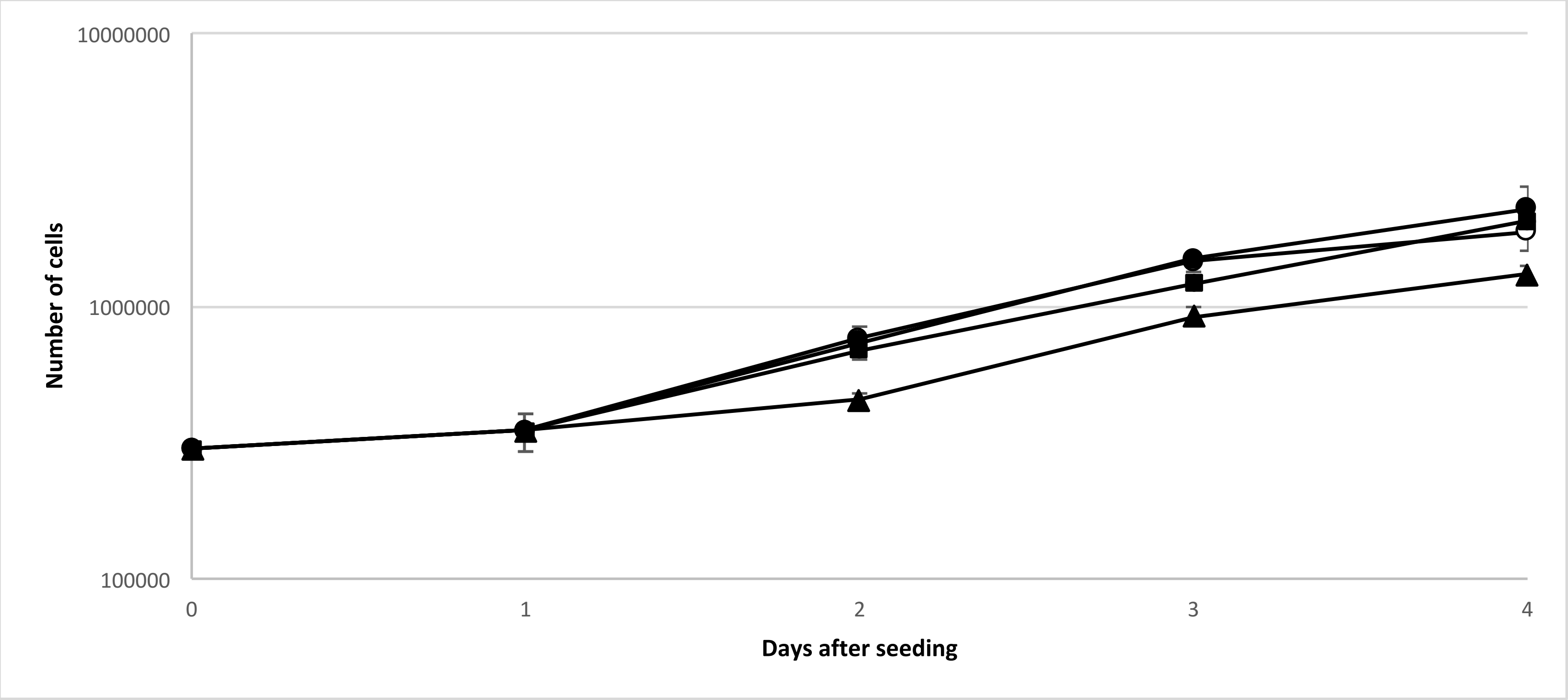

JIMT-1 Ambrosin

Sample #	Treatment	Day	Cells/ml	Cells/Petri dish	Mean value
Seeding	Ctrl	-1		300000	
Seeding	Ctrl	-1		300000	
Seeding	Ctrl	-1		300000	
0 hrs	Ctrl	0	36700	256900	
0 hrs	Ctrl	0	38900	272300	230767
0 hrs	Ctrl	0	23300	163100	
24 hrs	Ctrl	1	72200	505400	
24 hrs	Ctrl	1	67800	474600	505400
24 hrs	Ctrl	1	76600	536200	
24 hrs	1 µM	1	47800	334600	
24 hrs	1 µM	1	51100	357700	339733
24 hrs	1 µM	1	46700	326900	
24 hrs	2.5 µM	1	28900	202300	
24 hrs	2.5 µM	1	32200	225400	210000
24 hrs	2.5 µM	1	28900	202300	
24 hrs	5 µM	1	18900	132300	
24 hrs	5 µM	1	22200	155400	142567
24 hrs	5 µM	1	20000	140000	
48 hrs	Ctrl	2	96700	676900	
48 hrs	Ctrl	2	91100	637700	694867
48 hrs	Ctrl	2	110000	770000	
48 hrs	1 µM	2	85600	599200	
48 hrs	1 µM	2	84400	590800	580767
48 hrs	1 µM	2	78900	552300	
48 hrs	2.5 µM	2	38900	272300	
48 hrs	2.5 µM	2	33300	233100	269500
48 hrs	2.5 µM	2	43300	303100	
48 hrs	5 µM	2	16700	116900	
48 hrs	5 µM	2	14400	100800	108967
48 hrs	5 µM	2	15600	109200	
72 hrs	Ctrl	3	189000	1323000	
72 hrs	Ctrl	3	190000	1330000	1320667
72 hrs	Ctrl	3	187000	1309000	
72 hrs	1 µM	3	111000	777000	
72 hrs	1 µM	3	124000	868000	854000
72 hrs	1 µM	3	131000	917000	
72 hrs	2.5 µM	3	66700	466900	
72 hrs	2.5 µM	3	46700	326900	350233
72 hrs	2.5 µM	3	36700	256900	
72 hrs	5 µM	3	14400	100800	
72 hrs	5 µM	3	14400	100800	106167
72 hrs	5 µM	3	16700	116900	

Day	# of cells for			
	Ctrl	1 µM	2.5 µm	5 µM
0	300000	300000	300000	300000
1	230767	230767	230767	230767
2	505400	339733	210000	142567
3	694867	580767	269500	108967
4	1320667	854000	350233	106167

SD				
Day	Ctrl	1 µM	2.5 µm	5 µM
0	0	0	0	0
1	59105	59105	59105	59105
2	30800	16029	13337	11762
3	67955	25008	35084	8053
4	10693	71042	106927	9295

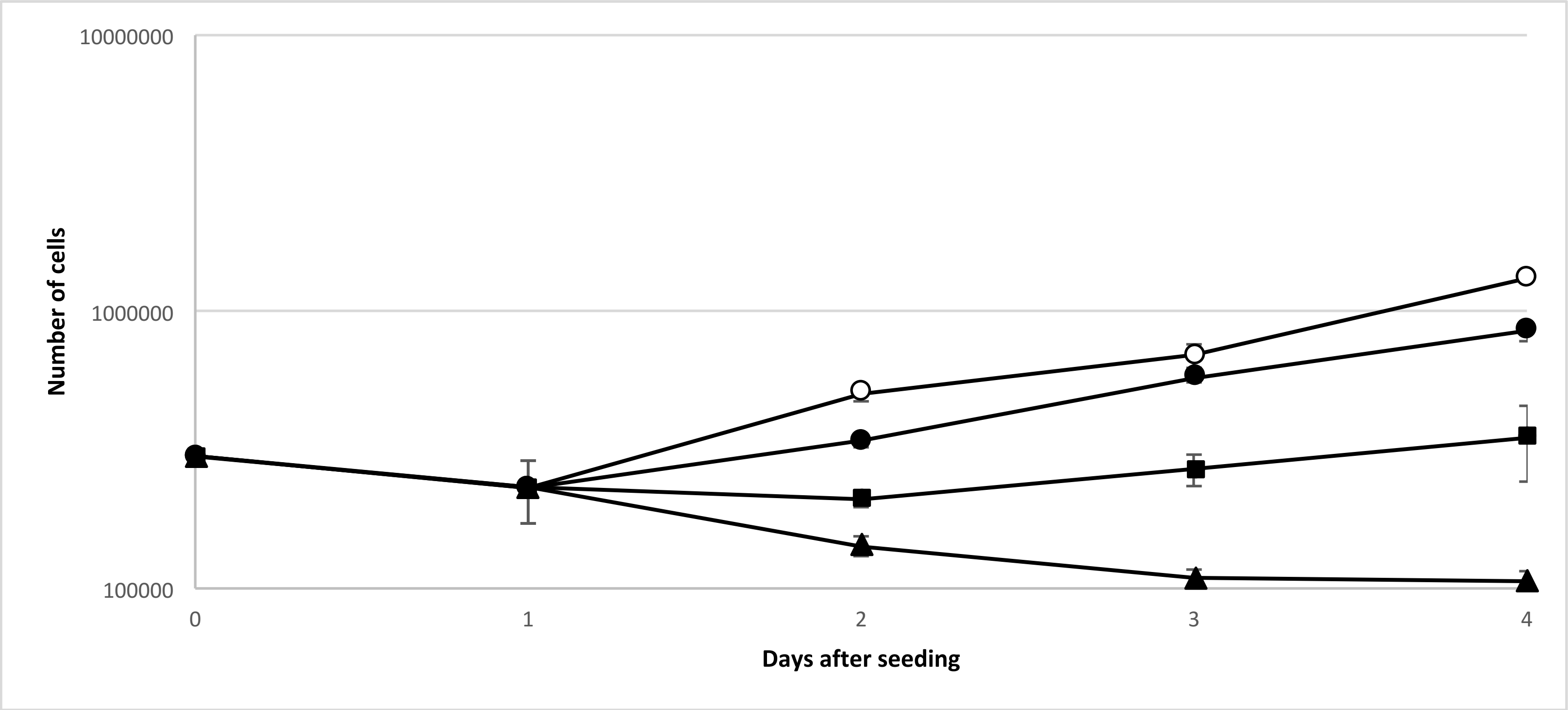

HCC1937 Damsin

Sample #	Treatment	Day	Cells/ml	Cells/Petri dish	Mean value
Seeding	Ctrl	-1		420000	
Seeding	Ctrl	-1		420000	
Seeding	Ctrl	-1		420000	
0 hrs	Ctrl	0	66700	466900	
0 hrs	Ctrl	0	73300	513100	474367
0 hrs	Ctrl	0	63300	443100	
24 hrs	Ctrl	1	90000	630000	
24 hrs	Ctrl	1	87800	614600	630000
24 hrs	Ctrl	1	92200	645400	
24 hrs	1 µM	1	87800	614600	
24 hrs	1 µM	1	72200	505400	521033
24 hrs	1 µM	1	63300	443100	
24 hrs	2.5 µM	1	74400	520800	
24 hrs	2.5 µM	1	85600	599200	573067
24 hrs	2.5 µM	1	85600	599200	
24 hrs	5 µM	1	73300	513100	
24 hrs	5 µM	1	62200	435400	471800
24 hrs	5 µM	1	66700	466900	
48 hrs	Ctrl	2	158000	1106000	
48 hrs	Ctrl	2	158000	1106000	1087333
48 hrs	Ctrl	2	150000	1050000	
48 hrs	1 µM	2	142000	994000	
48 hrs	1 µM	2	147000	1029000	1024333
48 hrs	1 µM	2	150000	1050000	
48 hrs	2.5 µM	2	120000	840000	
48 hrs	2.5 µM	2	113000	791000	830667
48 hrs	2.5 µM	2	123000	861000	
48 hrs	5 µM	2	100000	700000	
48 hrs	5 µM	2	76700	536900	612033
48 hrs	5 µM	2	85600	599200	
72 hrs	Ctrl	3	184000	1288000	
72 hrs	Ctrl	3	172000	1204000	1225000
72 hrs	Ctrl	3	169000	1183000	
72 hrs	1 µM	3	173000	1211000	
72 hrs	1 µM	3	187000	1309000	1232000
72 hrs	1 µM	3	168000	1176000	
72 hrs	2.5 µM	3	151000	1057000	
72 hrs	2.5 µM	3	143000	1001000	1045333
72 hrs	2.5 µM	3	154000	1078000	
72 hrs	5 µM	3	108000	756000	
72 hrs	5 µM	3	100000	700000	697900
72 hrs	5 µM	3	91100	637700	

Day	# of cells for			
	Ctrl	1 µM	2.5 µm	5 µM
0	420000	420000	420000	420000
1	474367	474367	474367	474367
2	630000	521033	573067	471800
3	1087333	1024333	830667	612033
4	1225000	1232000	1045333	697900

SD				
Day	Ctrl	1 µM	2.5 µm	5 µM
0	0	0	0	0
1	35592	35592	35592	35592
2	15400	86812	45264	39081
3	32332	28290	35921	82304
4	55561	68942	39804	59178

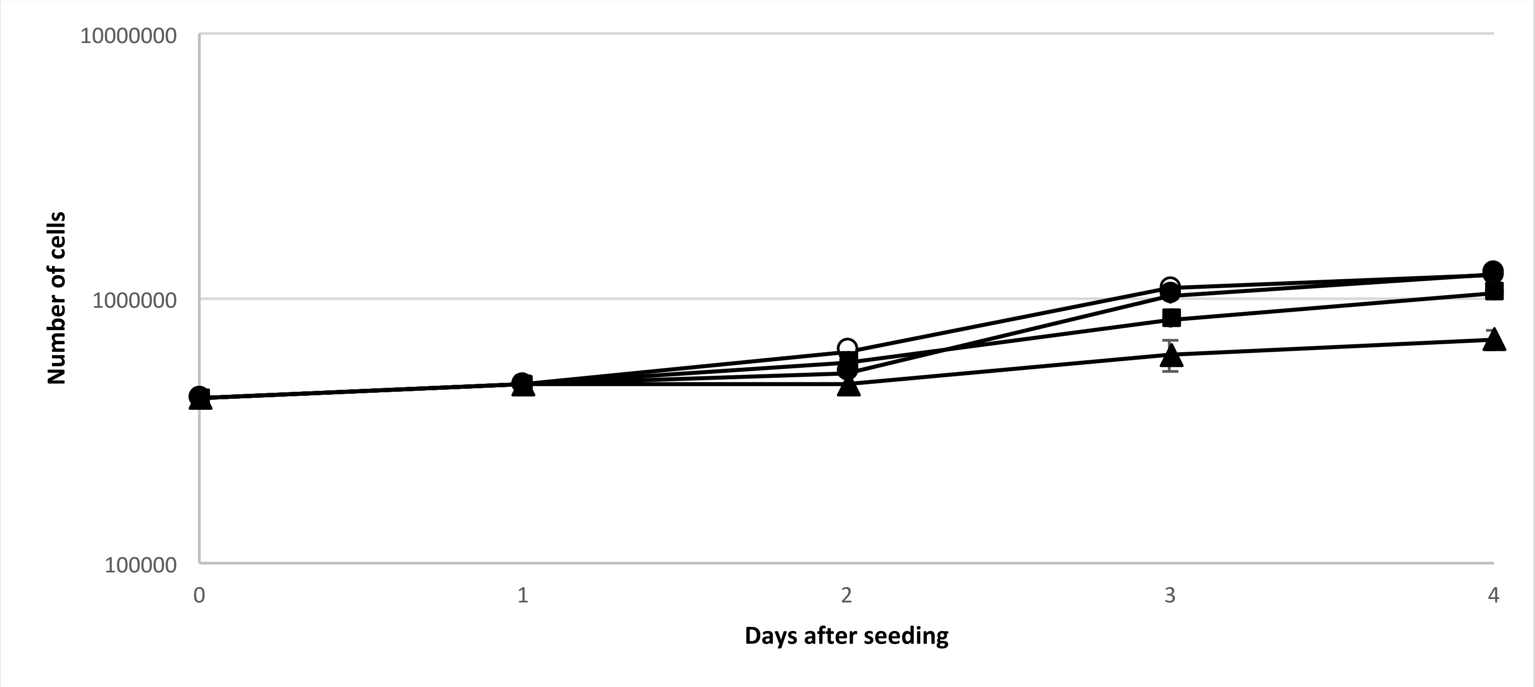

HCC1937 Ambrosin

Sample #	Treatment	Day	Cells/ml	Cells/Petri dish	Mean value
Seeding	Ctrl	-1		420000	
Seeding	Ctrl	-1		420000	
Seeding	Ctrl	-1		420000	
0 hrs	Ctrl	0	54400	380800	
0 hrs	Ctrl	0	58900	412300	406933
0 hrs	Ctrl	0	61100	427700	
24 hrs	Ctrl	1	87800	614600	
24 hrs	Ctrl	1	97800	684600	645633
24 hrs	Ctrl	1	91100	637700	
24 hrs	1 µM	1	83300	583100	
24 hrs	1 µM	1	81100	567700	611800
24 hrs	1 µM	1	97800	684600	
24 hrs	2.5 µM	1	78900	552300	
24 hrs	2.5 µM	1	68900	482300	531533
24 hrs	2.5 µM	1	80000	560000	
24 hrs	5 µM	1	58900	412300	
24 hrs	5 µM	1	43300	303100	350000
24 hrs	5 µM	1	47800	334600	
48 hrs	Ctrl	2	139000	973000	
48 hrs	Ctrl	2	137000	959000	1031333
48 hrs	Ctrl	2	166000	1162000	
48 hrs	1 µM	2	102000	714000	
48 hrs	1 µM	2	123000	861000	786333
48 hrs	1 µM	2	112000	784000	
48 hrs	2.5 µM	2	98900	692300	
48 hrs	2.5 µM	2	101000	707000	692067
48 hrs	2.5 µM	2	96700	676900	
48 hrs	5 µM	2	44400	310800	
48 hrs	5 µM	2	35600	249200	285133
48 hrs	5 µM	2	42200	295400	
72 hrs	Ctrl	3	212000	1484000	
72 hrs	Ctrl	3	223000	1561000	1533000
72 hrs	Ctrl	3	222000	1554000	
72 hrs	1 µM	3	218000	1526000	
72 hrs	1 µM	3	204000	1428000	1479333
72 hrs	1 µM	3	212000	1484000	
72 hrs	2.5 µM	3	161000	1127000	
72 hrs	2.5 µM	3	148000	1036000	1080333
72 hrs	2.5 µM	3	154000	1078000	
72 hrs	5 µM	3	57800	404600	
72 hrs	5 µM	3	72200	505400	458967
72 hrs	5 µM	3	66700	466900	

Day	# of cells for			
	Ctrl	1 µM	2.5 µm	5 µM
0	420000	420000	420000	420000
1	406933	406933	406933	406933
2	645633	611800	531533	350000
3	1031333	786333	692067	285133
4	1533000	1479333	1080333	458967

SD				
Day	Ctrl	1 µM	2.5 µm	5 µM
0	0	0	0	0
1	23906	23906	23906	23906
2	35668	63515	42811	56205
3	113377	73528	15051	32058
4	42579	49166	45545	50866

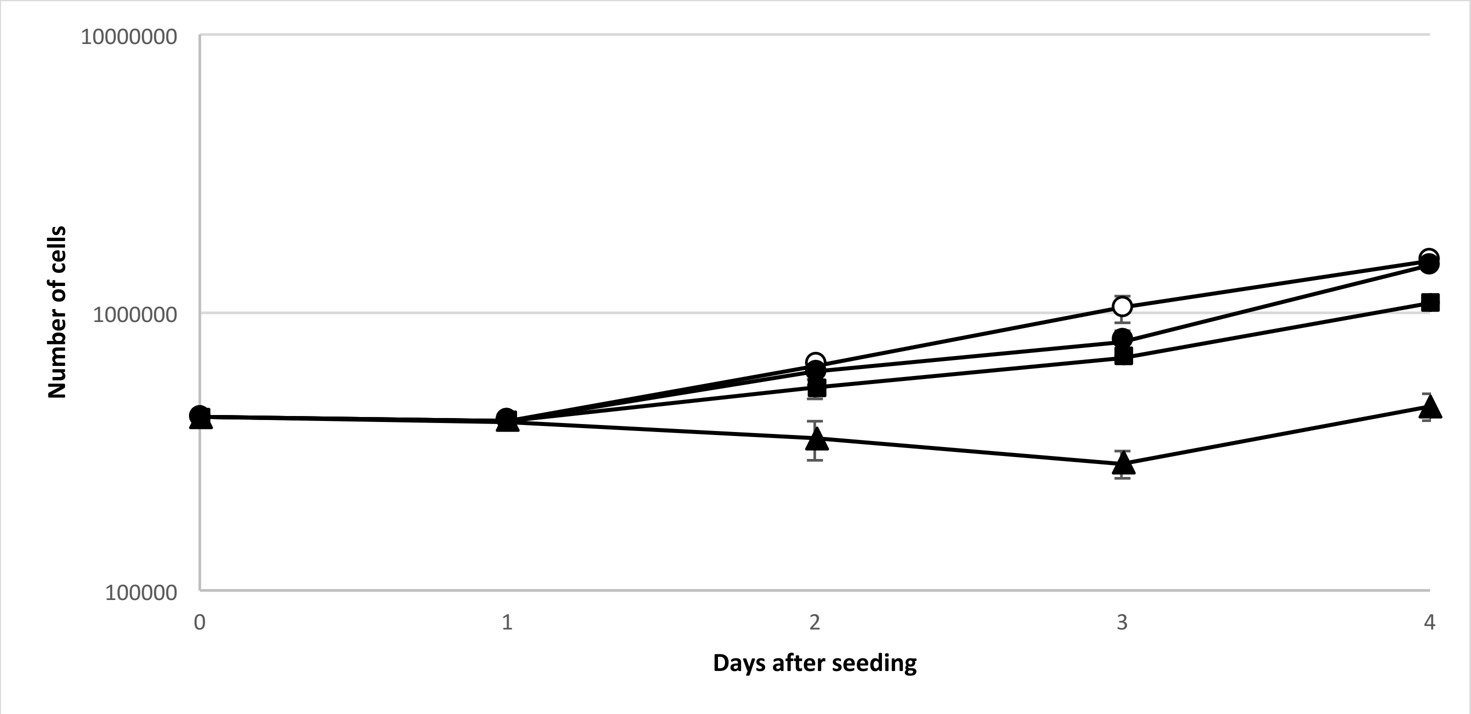

Supplement: S2 Fig — (PDF) [file pone.0184304.s002.pdf]
