# Supplementary material for: Anti-cancer stem cell activity of a sesquiterpene lactone isolated from Ambrosia arborescens and of a synthetic derivative
Source: PLoS One. 2017 Sep 1;12(9):e0184304. doi: 10.1371/journal.pone.0184304 (PMC5581169; doi:10.1371/journal.pone.0184304)
Supplement: S3 Fig — (PDF) [file pone.0184304.s003.pdf]

Original data for Figure 4

Ungated original data acquired using an Accuri C6 flow cytometer exported to excel in the signal acquisition program BD CSampler Software. For analysis of cell cycle phase distribution, the data was exported as FCS files and analysed using MultiCycle software (Phoenix Flow Systems, San Diego, CA, USA).

MCF-10A Damsin Day 4

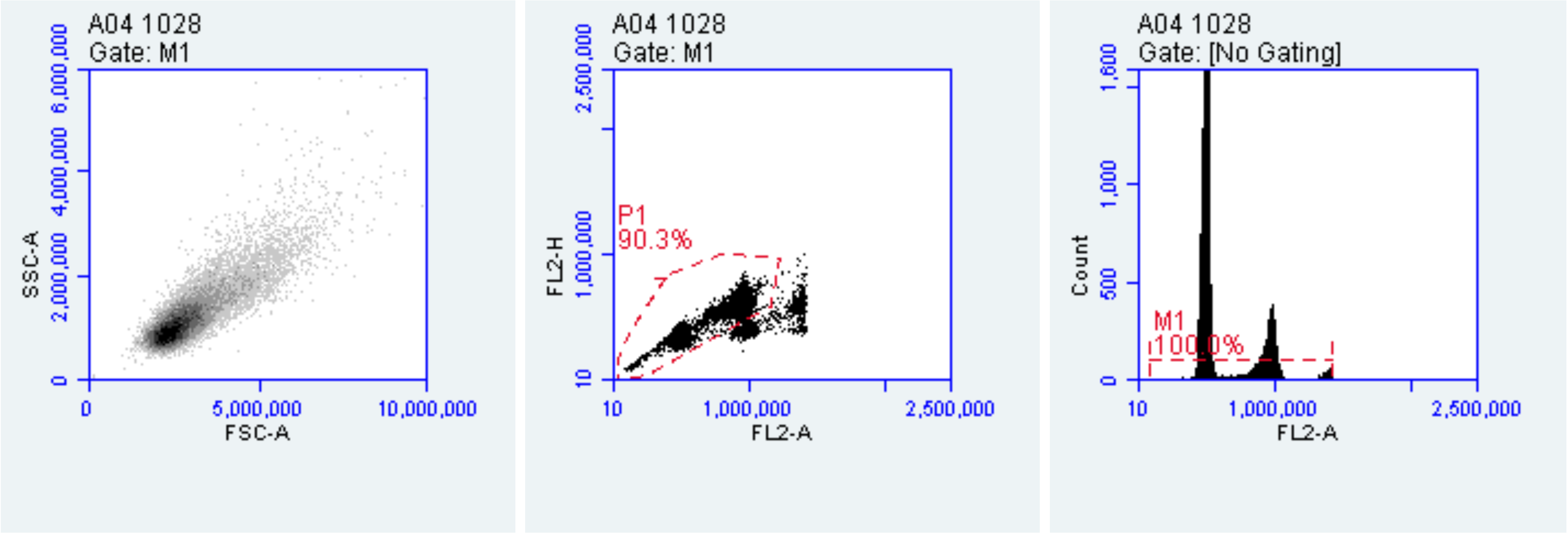

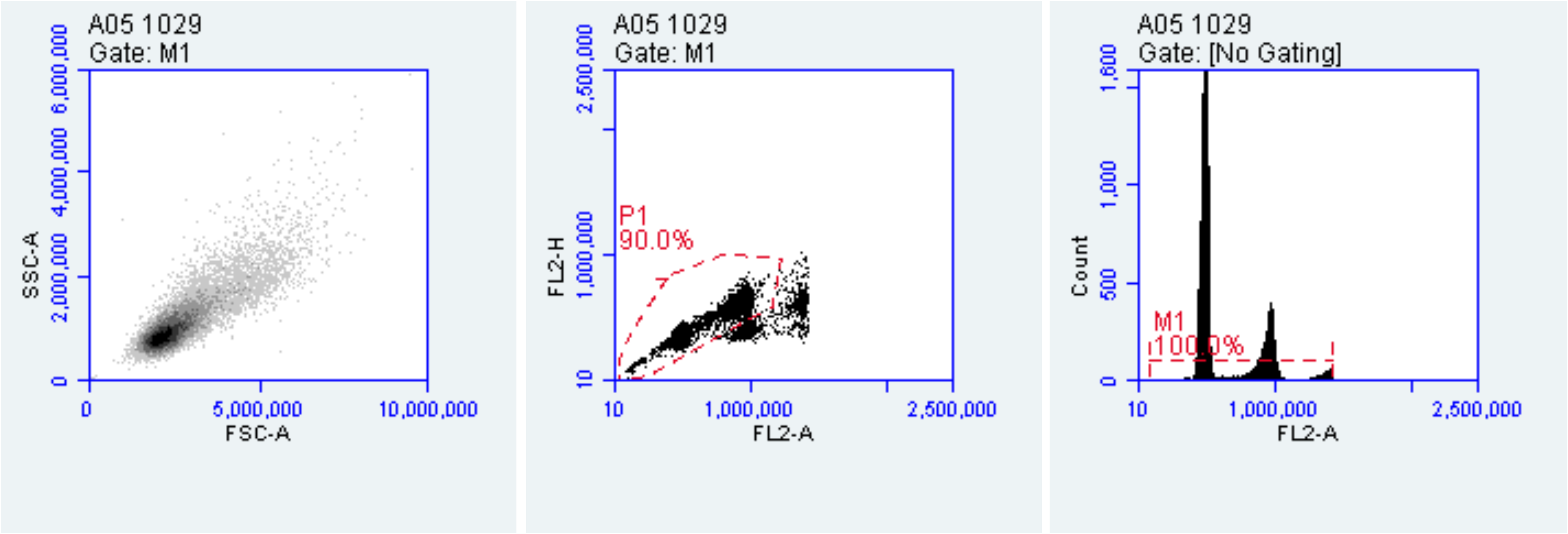
A05 1029
Gate: M1
SSC-A
0
2,000,000
4,000,000
6,000,000
0
5,000,000
10,000,000
FSC-A
A05 1029
Gate: M1
FL2-H
10
1,000,000
2,500,000
P1
90.0%
10
1,000,000
2,500,000
FL2-A
A05 1029
Gate: [No Gating]
Count
0
500
1,000
1,500
M1
100.0%
10
1,000,000
2,500,000
FL2-A

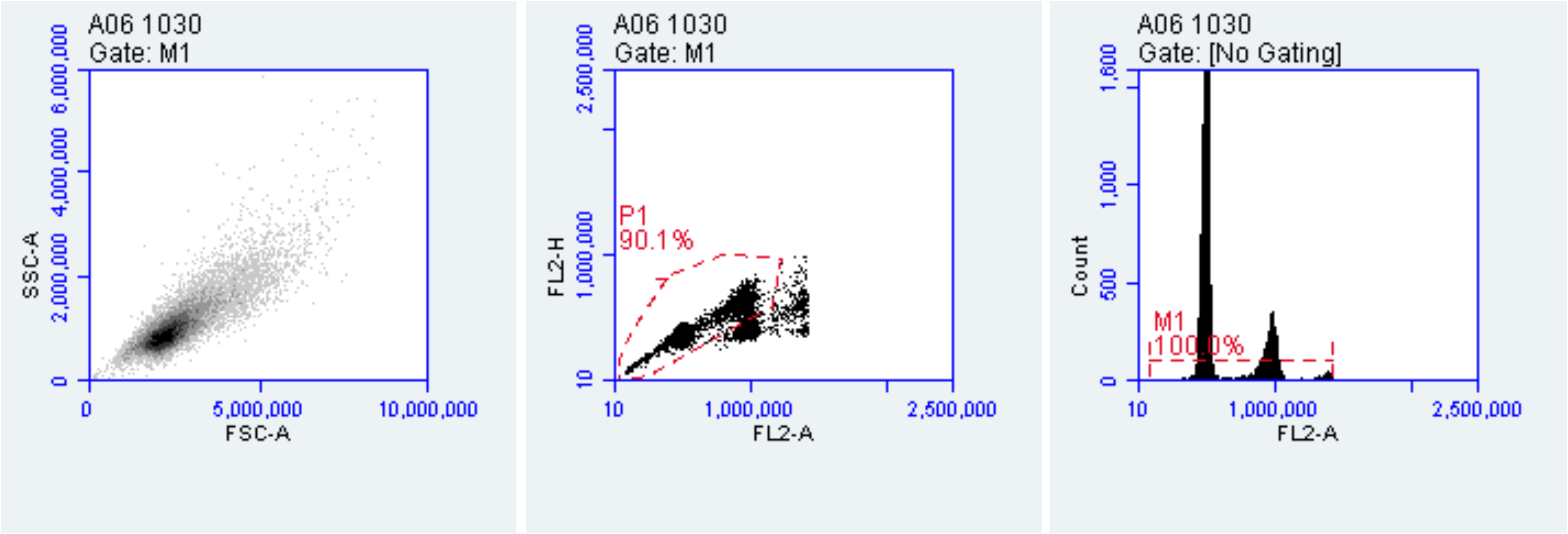

A06 1030
Gate: M1
SSC-A
FSC-A
0
2,000,000
4,000,000
6,000,000
5,000,000
10,000,000
A06 1030
Gate: M1
FL2-H
FL2-A
P1
90.1%
10
1,000,000
2,500,000
A06 1030
Gate: [No Gating]
Count
M1
100.0%
0
500
1,000
1,600
10
1,000,000
2,500,000

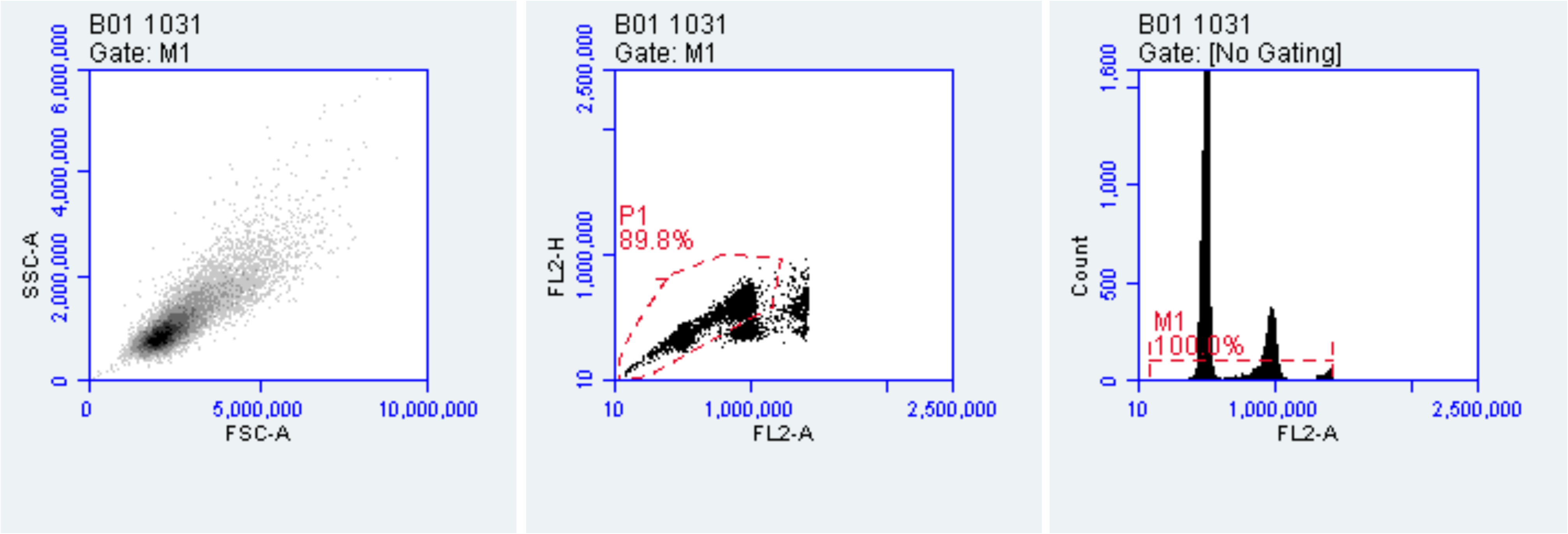
B01 1031
Gate: M1
SSC-A
FSC-A
0
2,000,000
4,000,000
6,000,000
5,000,000
10,000,000
B01 1031
Gate: M1
P1
89.8%
FL2-H
FL2-A
10
1,000,000
2,500,000
B01 1031
Gate: [No Gating]
M1
100.0%
Count
FL2-A
0
500
1,000
1,500
10
1,000,000
2,500,000

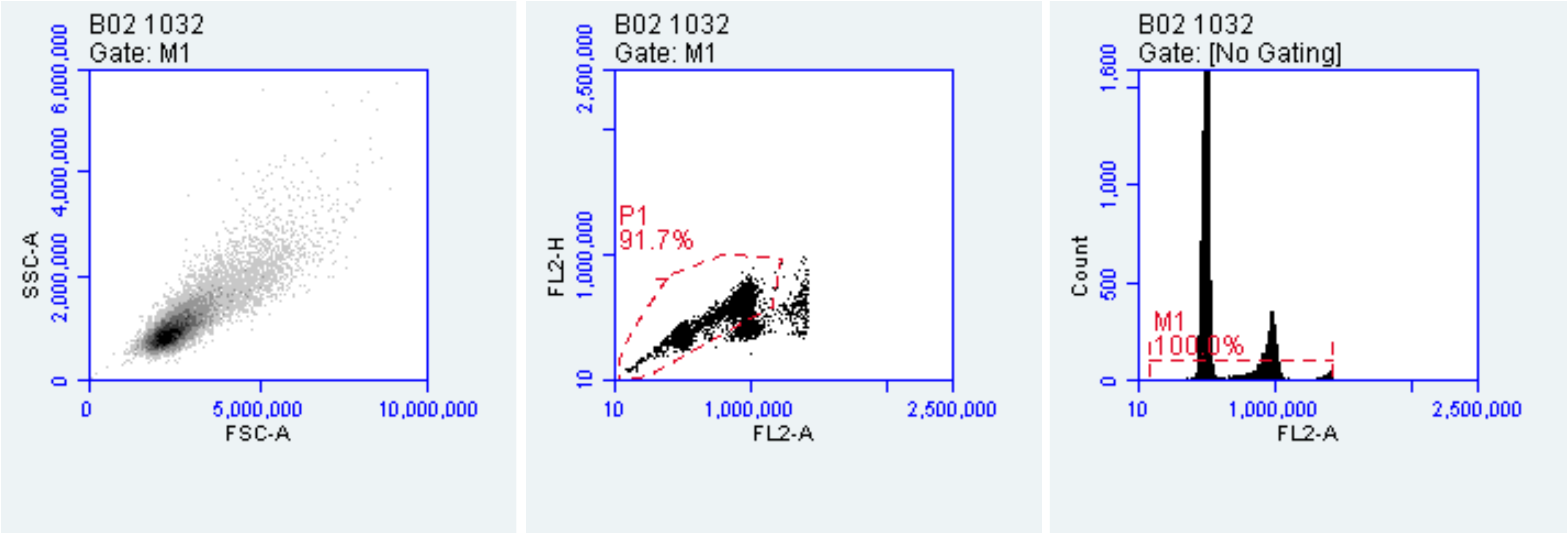
B02 1032
Gate: M1
SSC-A
0
2,000,000
4,000,000
6,000,000
0
5,000,000
10,000,000
FSC-A
B02 1032
Gate: M1
FL2-H
10
1,000,000
2,500,000
P1
91.7%
10
1,000,000
2,500,000
FL2-A
B02 1032
Gate: [No Gating]
Count
0
500
1,000
1,500
M1
100.0%
10
1,000,000
2,500,000
FL2-A

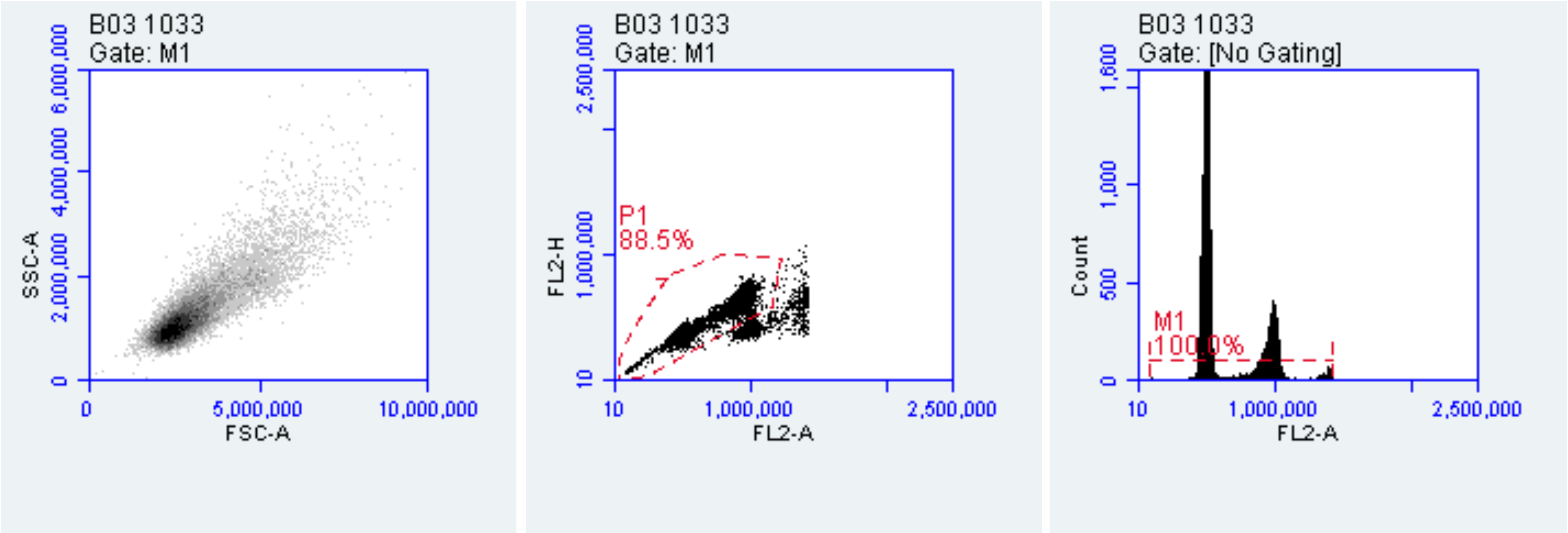
B03 1033
Gate: M1
SSC-A
0
2,000,000
4,000,000
6,000,000
FSC-A
0
5,000,000
10,000,000
B03 1033
Gate: M1
FL2-H
10
1,000,000
2,500,000
P1
88.5%
FL2-A
10
1,000,000
2,500,000
B03 1033
Gate: [No Gating]
Count
0
500
1,000
1,500
M1
100.0%
FL2-A
10
1,000,000
2,500,000

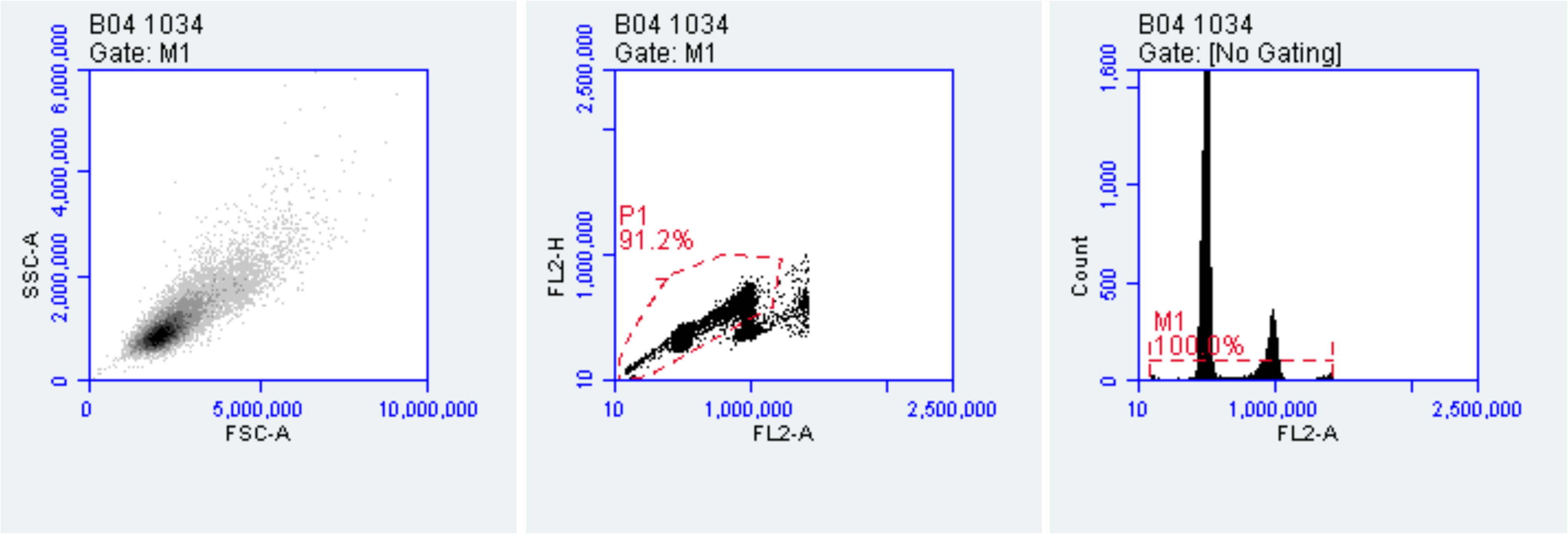
B04 1034
Gate: M1
SSC-A
FSC-A
0
2,000,000
4,000,000
6,000,000
5,000,000
10,000,000
B04 1034
Gate: M1
FL2-H
FL2-A
P1
91.2%
10
1,000,000
2,500,000
B04 1034
Gate: [No Gating]
Count
M1
100.0%
0
500
1,000
1,500

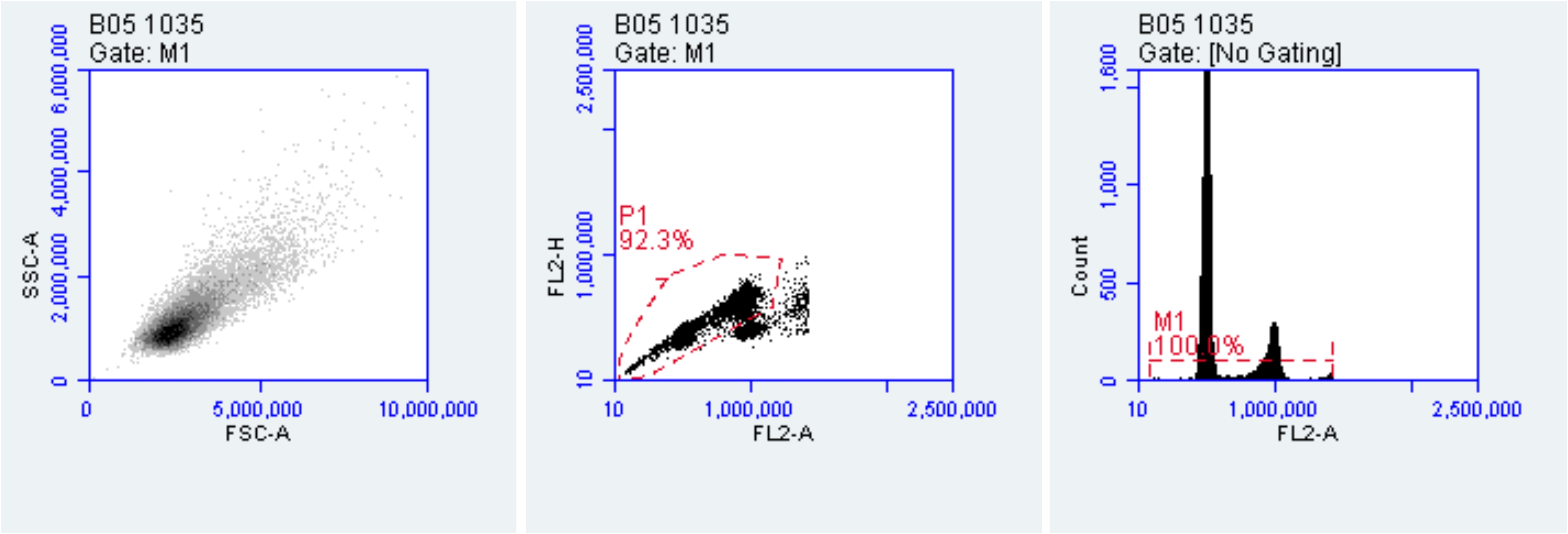
B05 1035
Gate: M1
SSC-A
FSC-A
0
2,000,000
4,000,000
6,000,000
5,000,000
10,000,000
B05 1035
Gate: M1
FL2-H
FL2-A
P1
92.3%
10
1,000,000
2,500,000
B05 1035
Gate: [No Gating]
Count
FL2-A
M1
100.0%
500
1,000
1,500

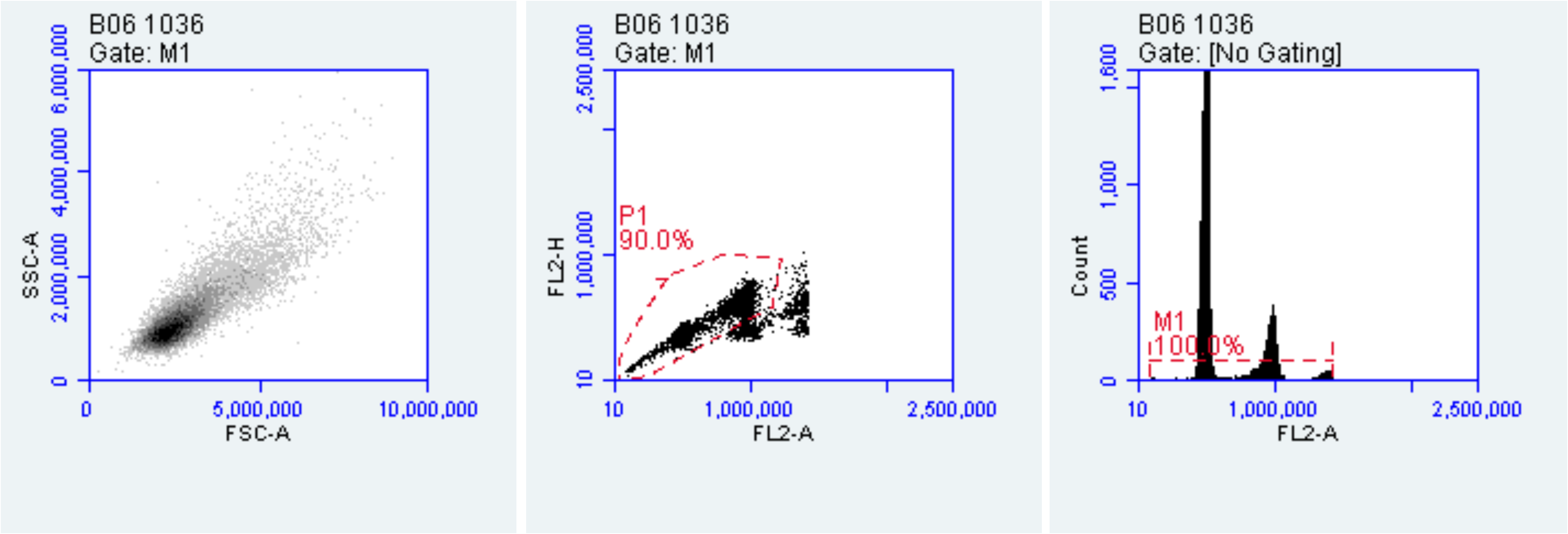

B06 1036
Gate: M1
SSC-A
FSC-A
0
2,000,000
4,000,000
6,000,000
5,000,000
10,000,000
B06 1036
Gate: M1
FL2-H
FL2-A
P1
90.0%
10
1,000,000
2,500,000
B06 1036
Gate: [No Gating]
Count
FL2-A
M1
100.0%
0
500
1,000
1,500
10
1,000,000
2,500,000

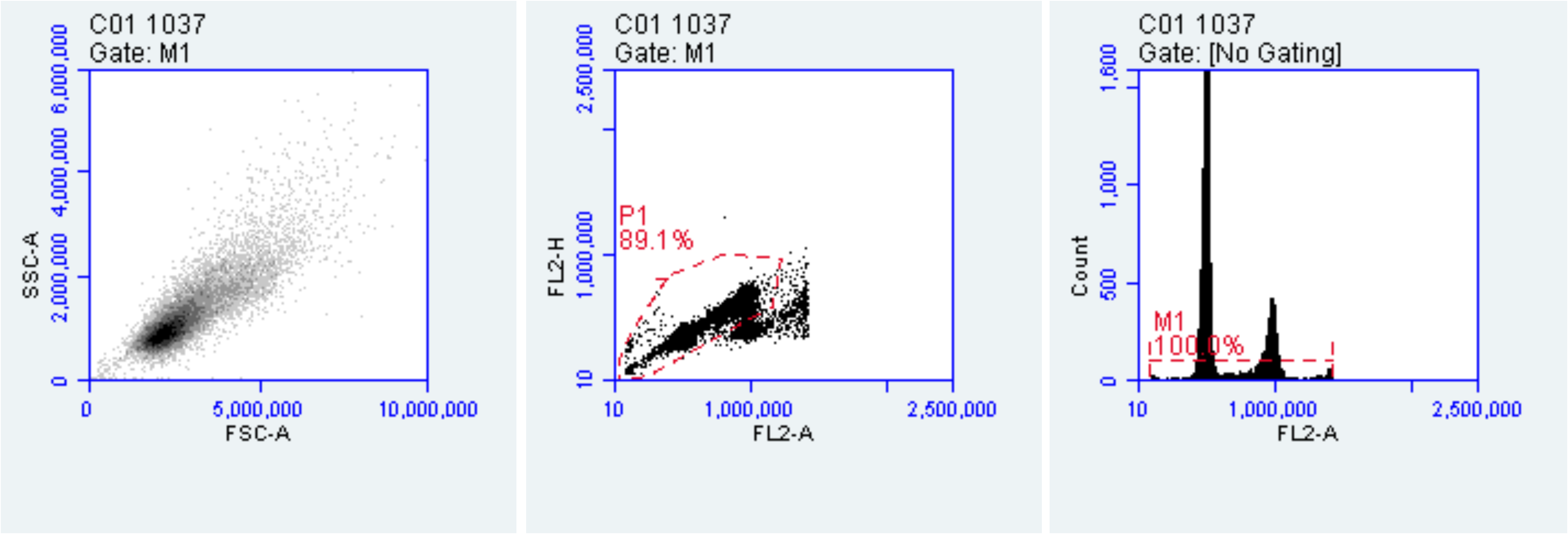
C01 1037
Gate: M1
SSC-A
FSC-A
0
2,000,000
4,000,000
6,000,000
0
5,000,000
10,000,000
C01 1037
Gate: M1
P1
89.1%
FL2-H
FL2-A
10
1,000,000
2,500,000
10
1,000,000
2,500,000
C01 1037
Gate: [No Gating]
M1
100.0%
Count
FL2-A
0
500
1,000
1,600
10
1,000,000
2,500,000

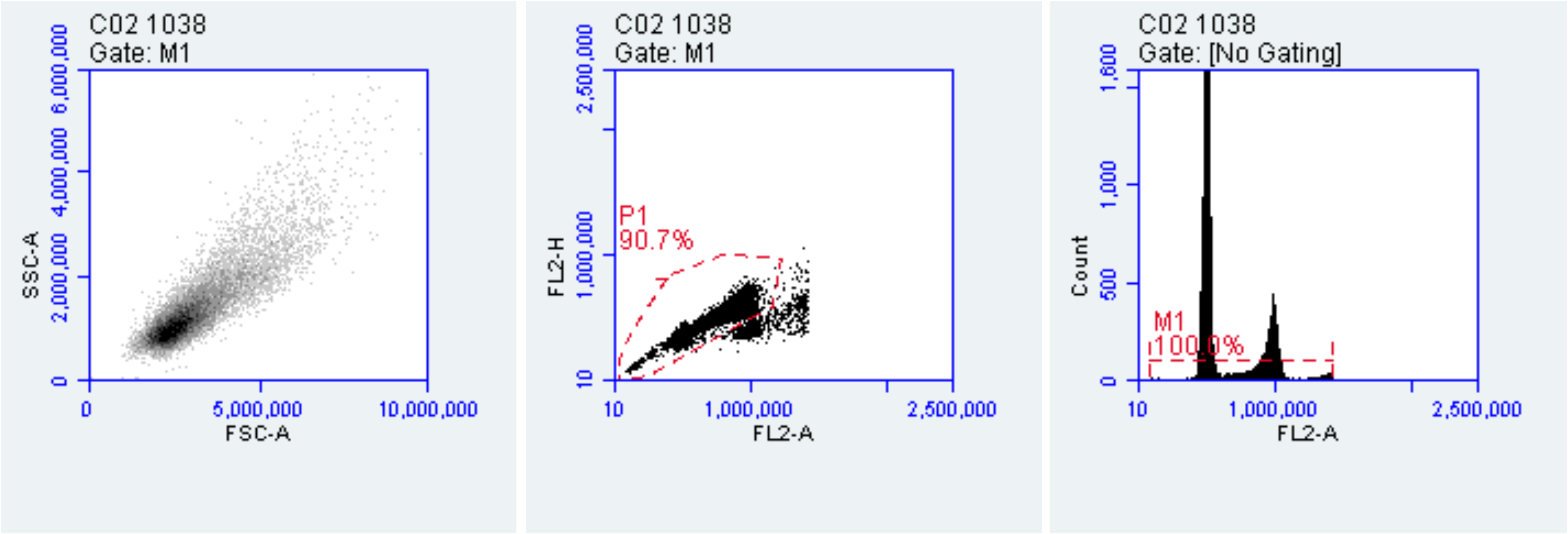

C02 1038
Gate: M1
SSC-A
FSC-A
0
2,000,000
4,000,000
6,000,000
5,000,000
10,000,000
C02 1038
Gate: M1
FL2-H
FL2-A
10
1,000,000
2,500,000
P1
90.7%
C02 1038
Gate: [No Gating]
Count
0
500
1,000
1,500
10
1,000,000
2,500,000
M1
100.0%

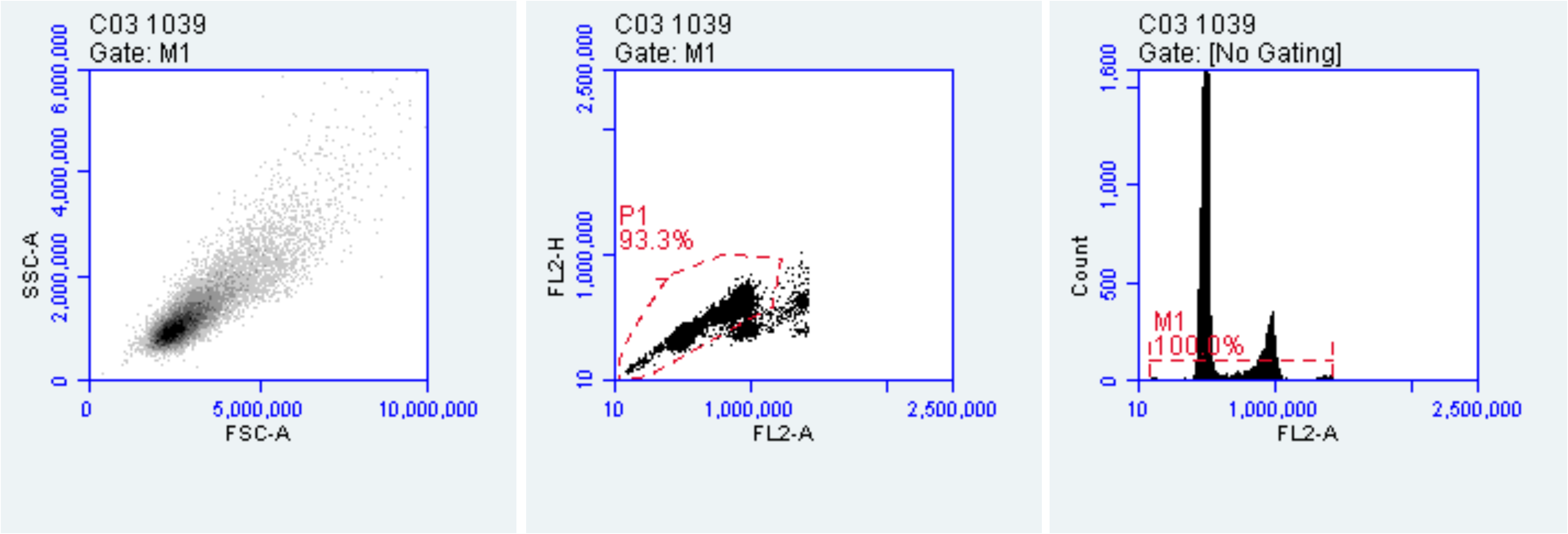
C03 1039
Gate: M1
SSC-A
FSC-A
0
2,000,000
4,000,000
6,000,000
5,000,000
10,000,000
C03 1039
Gate: M1
FL2-H
FL2-A
10
1,000,000
2,500,000
P1
93.3%
C03 1039
Gate: [No Gating]
Count
FL2-A
0
500
1,000
1,600
10
1,000,000
2,500,000
M1
100.0%

MCF-7 Damsin Day 4

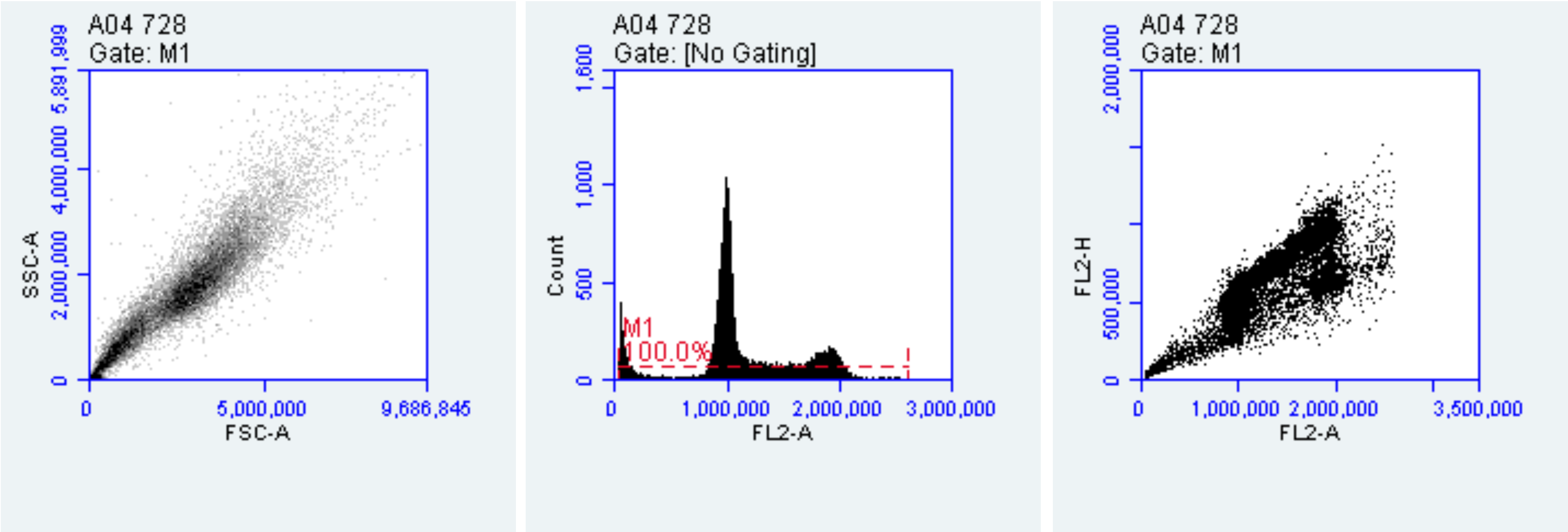

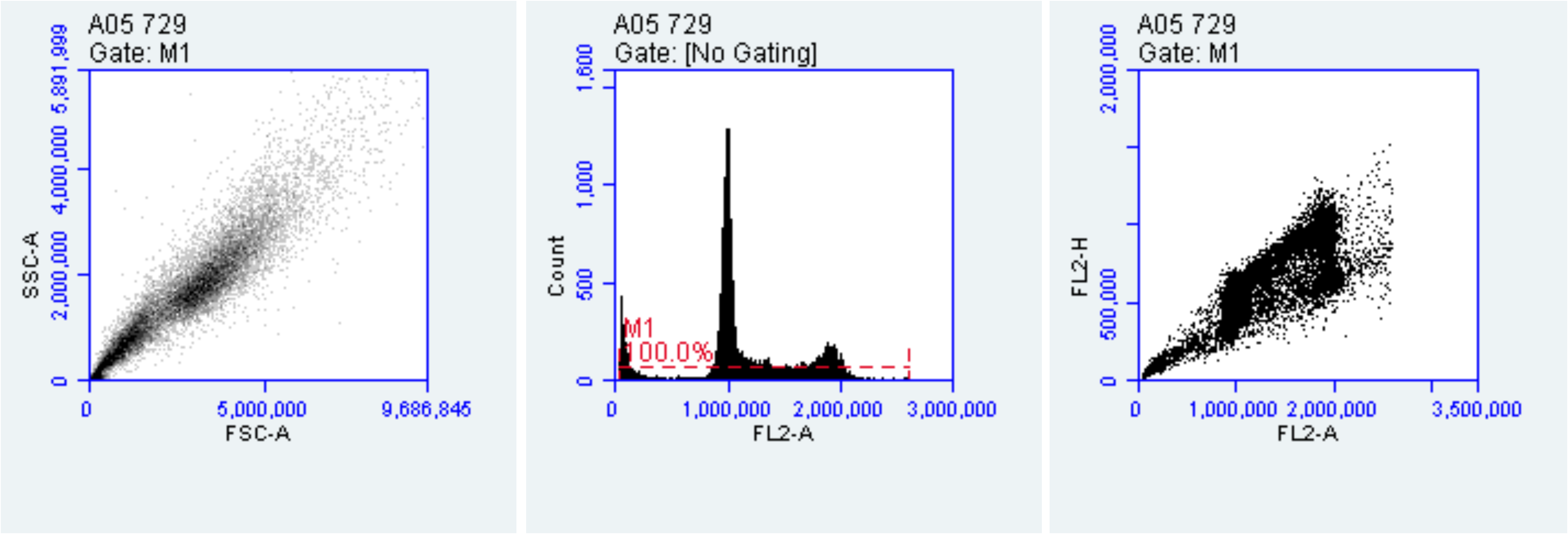

A05 729
Gate: M1
SSC-A
0
2,000,000
4,000,000
5,891,999
0
5,000,000
9,686,845
FSC-A
A05 729
Gate: [No Gating]
Count
0
500
1,000
1,600
M1
100.0%
0
1,000,000
2,000,000
3,000,000
FL2-A
A05 729
Gate: M1
FL2-H
0
500,000
2,000,000
0
1,000,000
2,000,000
3,500,000
FL2-A

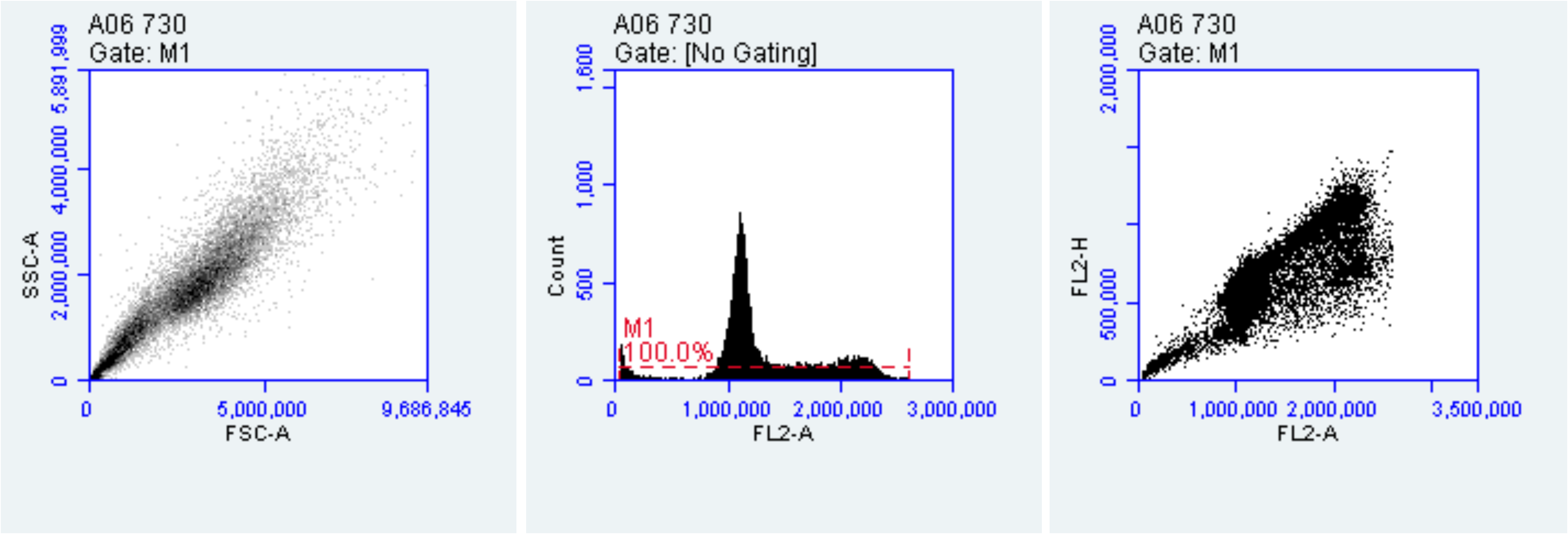

A06 730
Gate: M1
SSC-A
0
2,000,000
4,000,000
5,891,999
5,000,000
9,686,845
FSC-A
A06 730
Gate: [No Gating]
Count
0
500
1,000
1,600
M1
100.0%
1,000,000
2,000,000
3,000,000
FL2-A
A06 730
Gate: M1
FL2-H
500,000
2,000,000
3,500,000
FL2-A

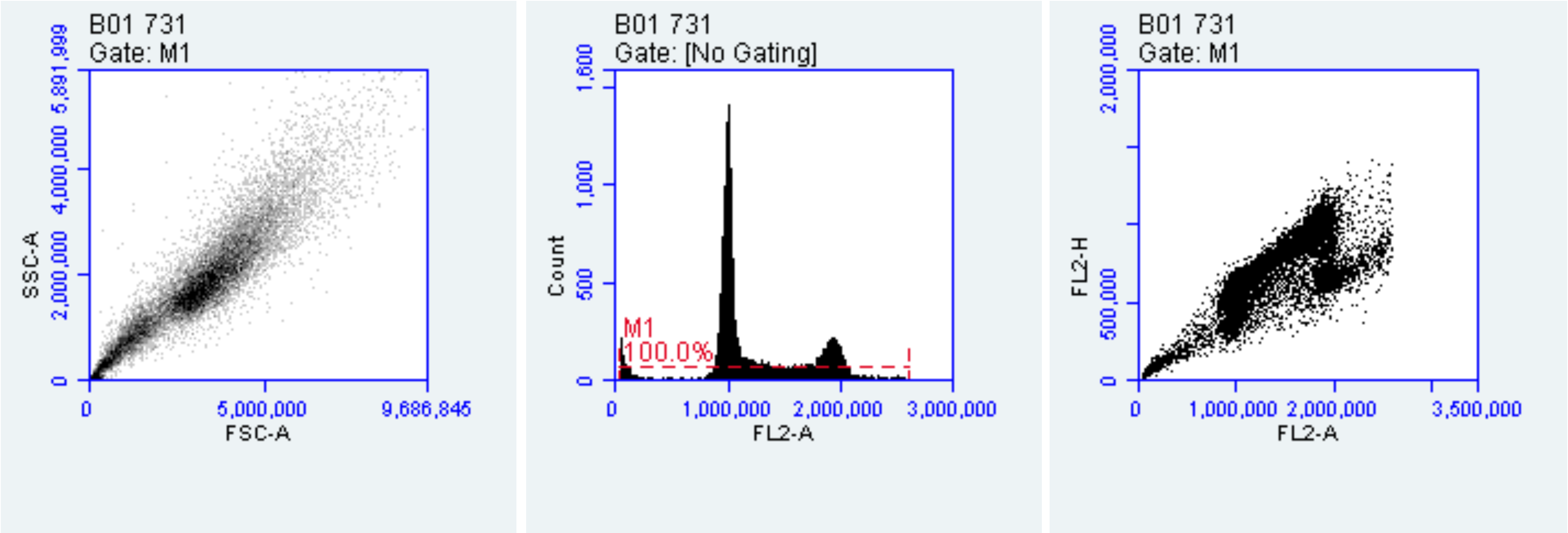
B01 731
Gate: M1
SSC-A
0
2,000,000
4,000,000
5,891,999
0
5,000,000
9,686,845
FSC-A
B01 731
Gate: [No Gating]
Count
0
500
1,000
1,600
M1
100.0%
0
1,000,000
2,000,000
3,000,000
FL2-A
B01 731
Gate: M1
FL2-H
0
500,000
2,000,000
0
1,000,000
2,000,000
3,500,000
FL2-A

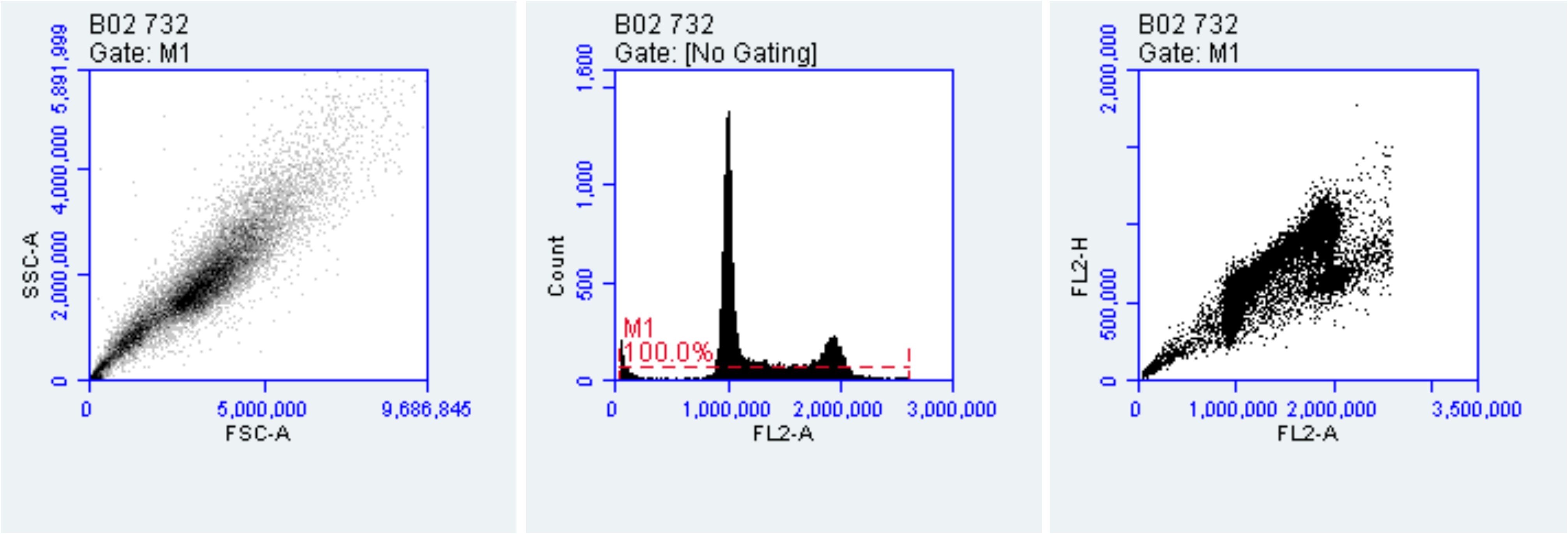

B02 732
Gate: M1
SSC-A
5,891,999
4,000,000
2,000,000
0
0
5,000,000
9,686,845
FSC-A
B02 732
Gate: [No Gating]
Count
1,600
1,000
500
0
M1
100.0%
0
1,000,000
2,000,000
3,000,000
FL2-A
B02 732
Gate: M1
FL2-H
2,000,000
500,000
0
0
1,000,000
2,000,000
3,500,000
FL2-A

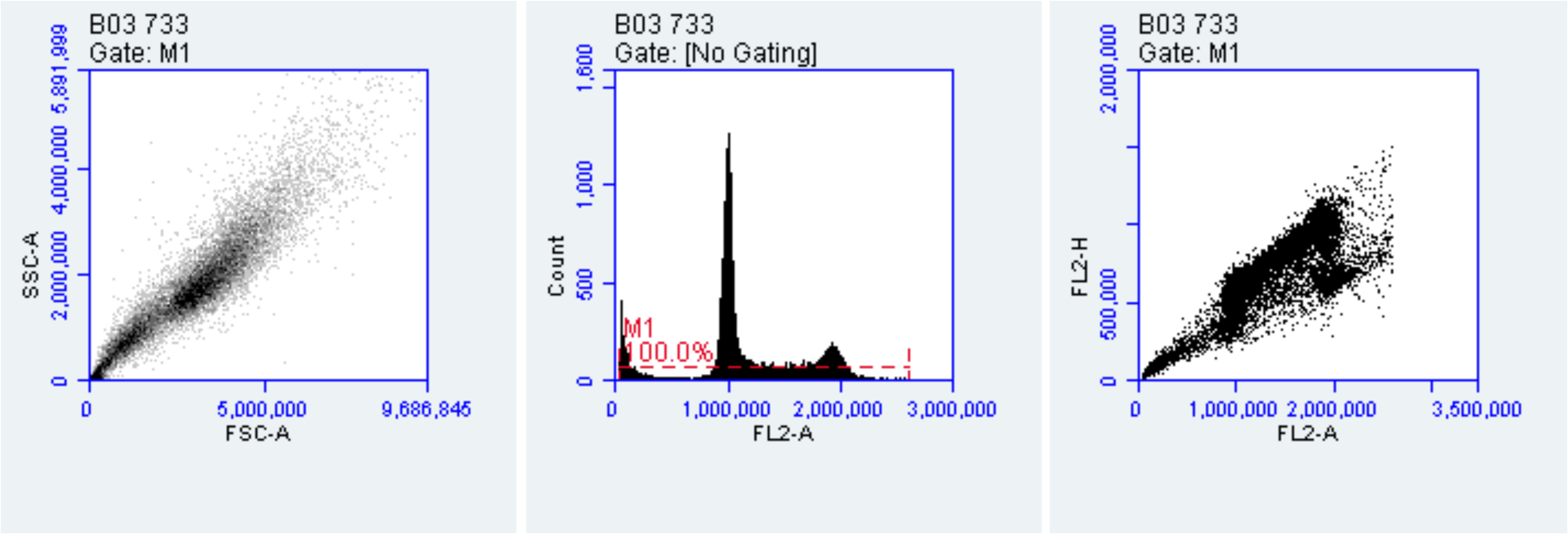

B03 733
Gate: M1
SSC-A
FSC-A
0
2,000,000
4,000,000
5,891,999
5,000,000
9,686,845
B03 733
Gate: [No Gating]
Count
FL2-A
0
500
1,000
1,600
1,000,000
2,000,000
3,000,000
M1
100.0%
B03 733
Gate: M1
FL2-H
FL2-A
0
500,000
2,000,000
1,000,000
2,000,000
3,500,000

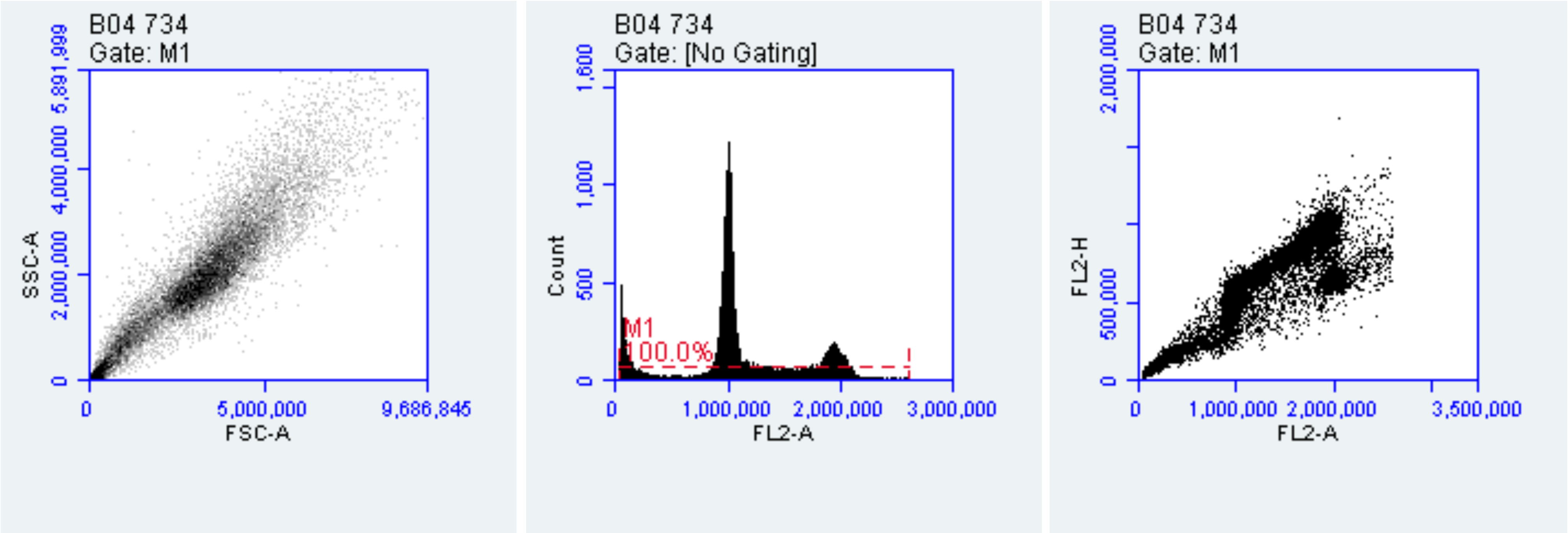
B04 734
Gate: M1
SSC-A
FSC-A
0
2,000,000
4,000,000
5,891,999
5,000,000
9,686,845
B04 734
Gate: [No Gating]
Count
FL2-A
M1
100.0%
0
500
1,000
1,600
1,000,000
2,000,000
3,000,000
B04 734
Gate: M1
FL2-H
FL2-A
0
500,000
2,000,000
1,000,000
2,000,000
3,500,000

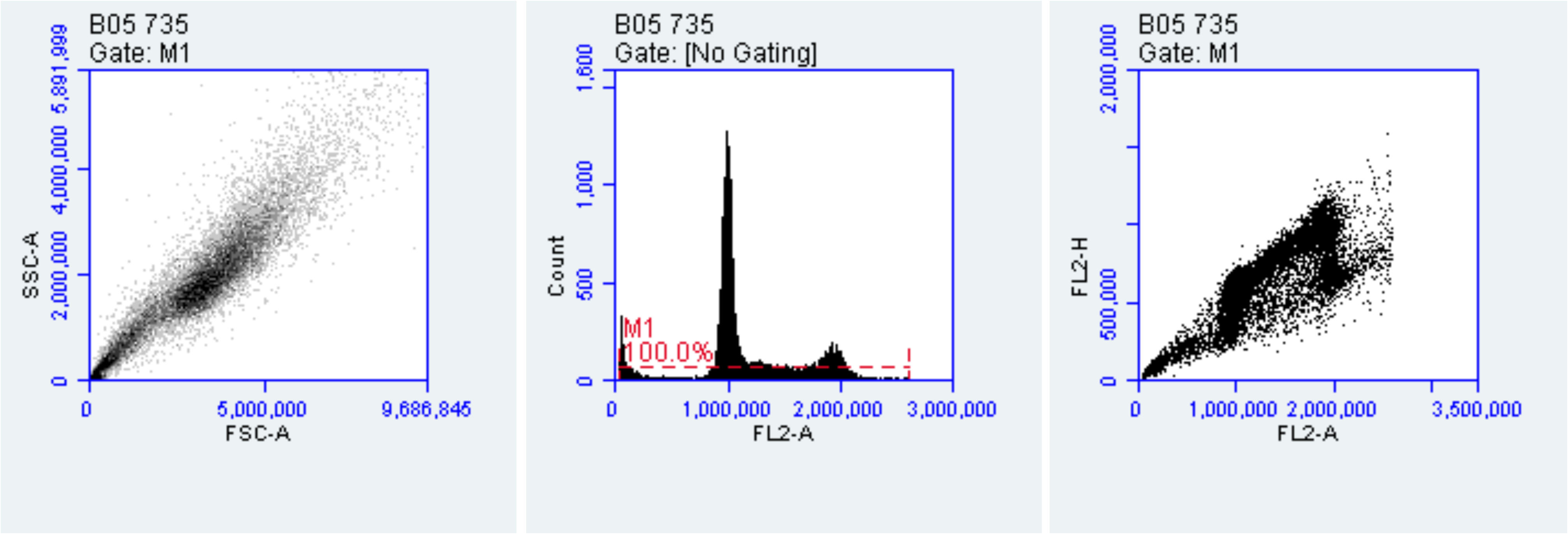

B05 735
Gate: M1
SSC-A
FSC-A
0
2,000,000
4,000,000
5,891,999
5,000,000
9,686,845
B05 735
Gate: [No Gating]
Count
FL2-A
0
500
1,000
1,600
1,000,000
2,000,000
3,000,000
M1
100.0%
B05 735
Gate: M1
FL2-H
FL2-A
0
500,000
2,000,000
1,000,000
2,000,000
3,500,000

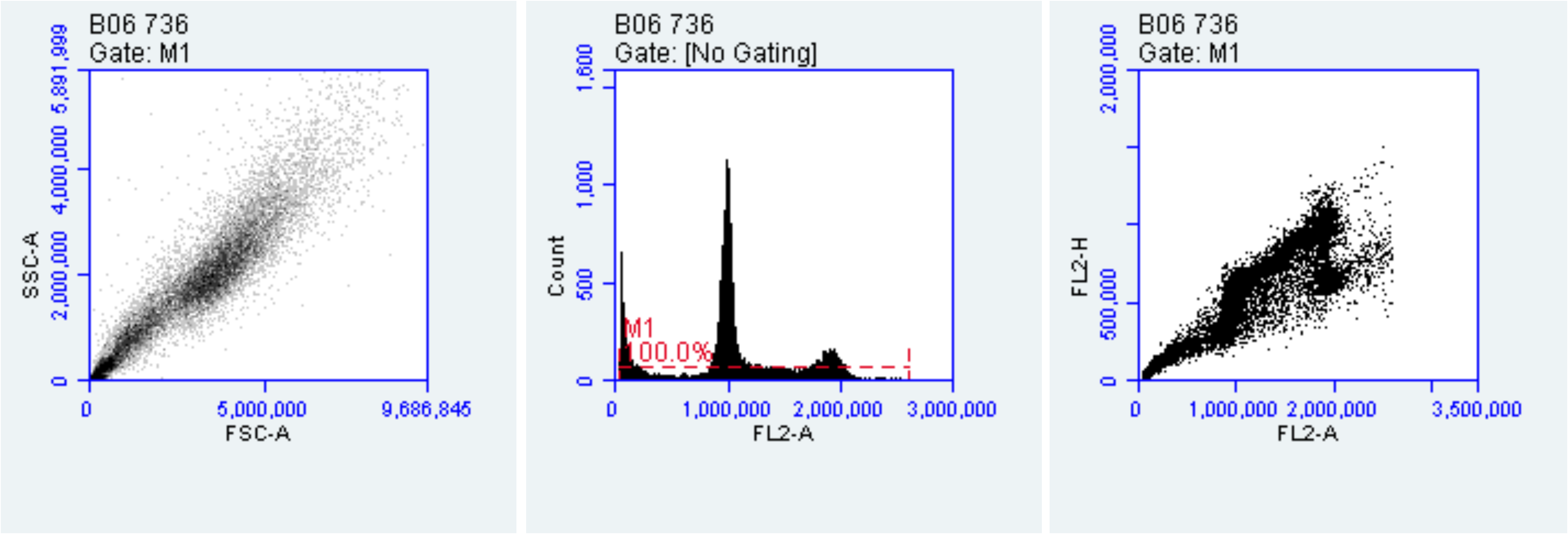
B06 736
Gate: M1
SSC-A
0
2,000,000
4,000,000
5,891,999
0
5,000,000
9,686,845
FSC-A
B06 736
Gate: [No Gating]
Count
0
500
1,000
1,600
M1
100.0%
0
1,000,000
2,000,000
3,000,000
FL2-A
B06 736
Gate: M1
FL2-H
0
500,000
2,000,000
0
1,000,000
2,000,000
3,500,000
FL2-A

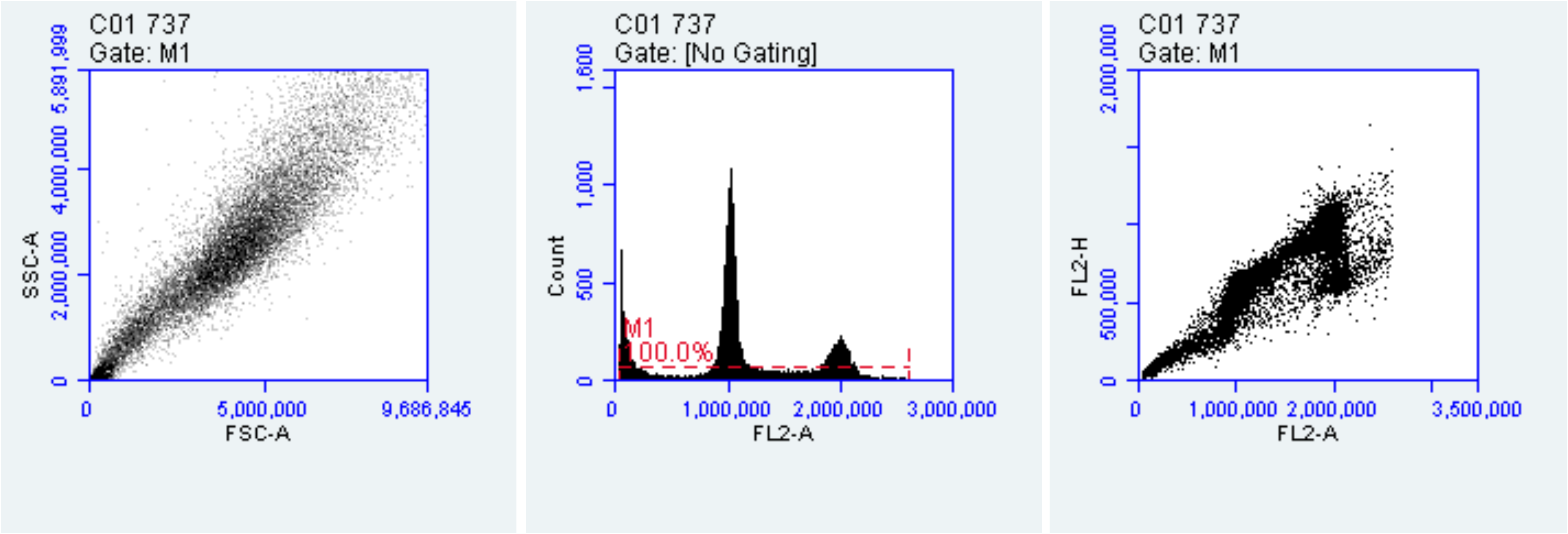
C01 737
Gate: M1
SSC-A
0
2,000,000
4,000,000
5,891,999
0
5,000,000
9,686,845
FSC-A
C01 737
Gate: [No Gating]
Count
0
500
1,000
1,600
M1
100.0%
0
1,000,000
2,000,000
3,000,000
FL2-A
C01 737
Gate: M1
FL2-H
0
500,000
2,000,000
0
1,000,000
2,000,000
3,500,000
FL2-A

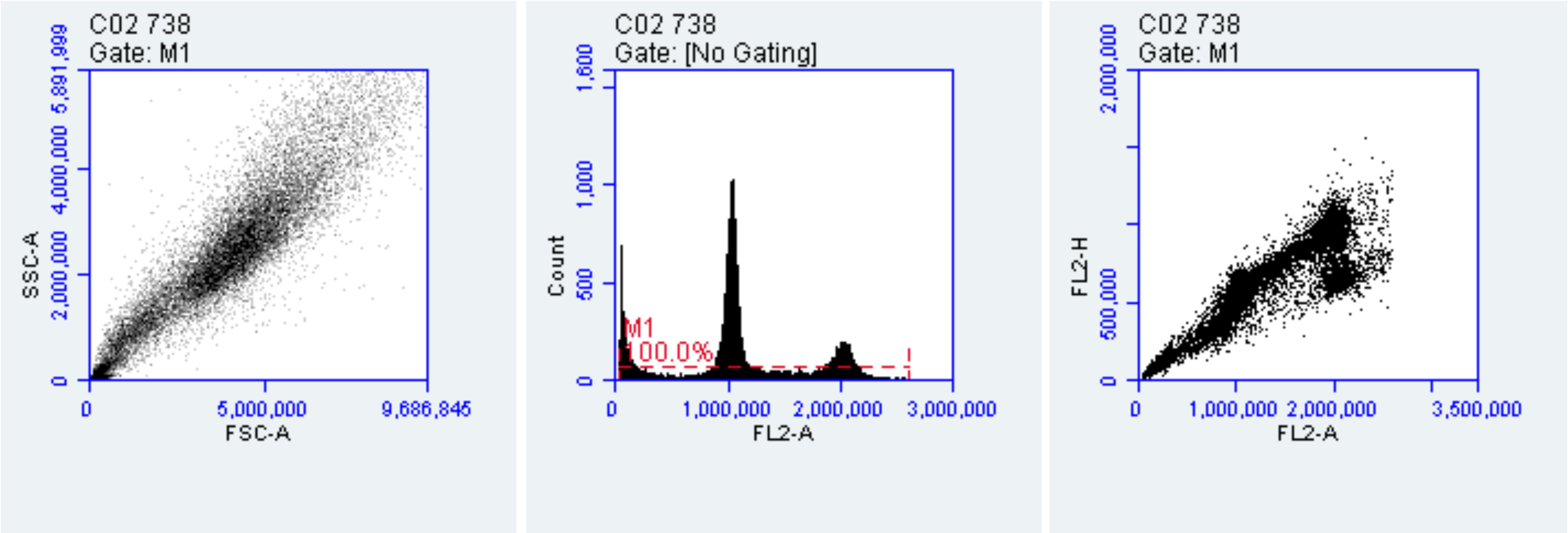
C02 738
Gate: M1
SSC-A
FSC-A
0
2,000,000
4,000,000
5,891,999
5,000,000
9,686,845
C02 738
Gate: [No Gating]
Count
FL2-A
M1
100.0%
0
500
1,000
1,600
1,000,000
2,000,000
3,000,000
C02 738
Gate: M1
FL2-H
FL2-A
0
500,000
2,000,000
1,000,000
2,000,000
3,500,000

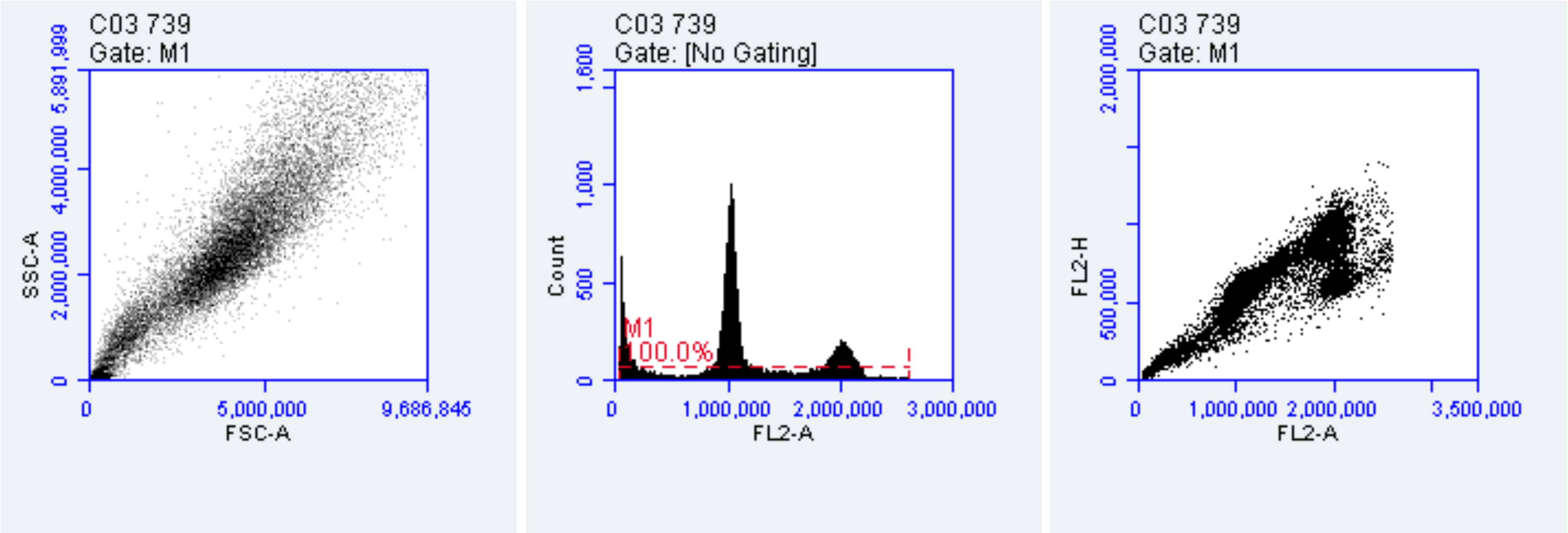
C03 739
Gate: M1
SSC-A
FSC-A
0
2,000,000
4,000,000
5,891,999
5,000,000
9,686,845
C03 739
Gate: [No Gating]
Count
FL2-A
0
500
1,000
1,600
1,000,000
2,000,000
3,000,000
M1
100.0%
C03 739
Gate: M1
FL2-H
FL2-A
0
500,000
2,000,000
1,000,000
2,000,000
3,500,000

JIMT-1 Damsin Day 4

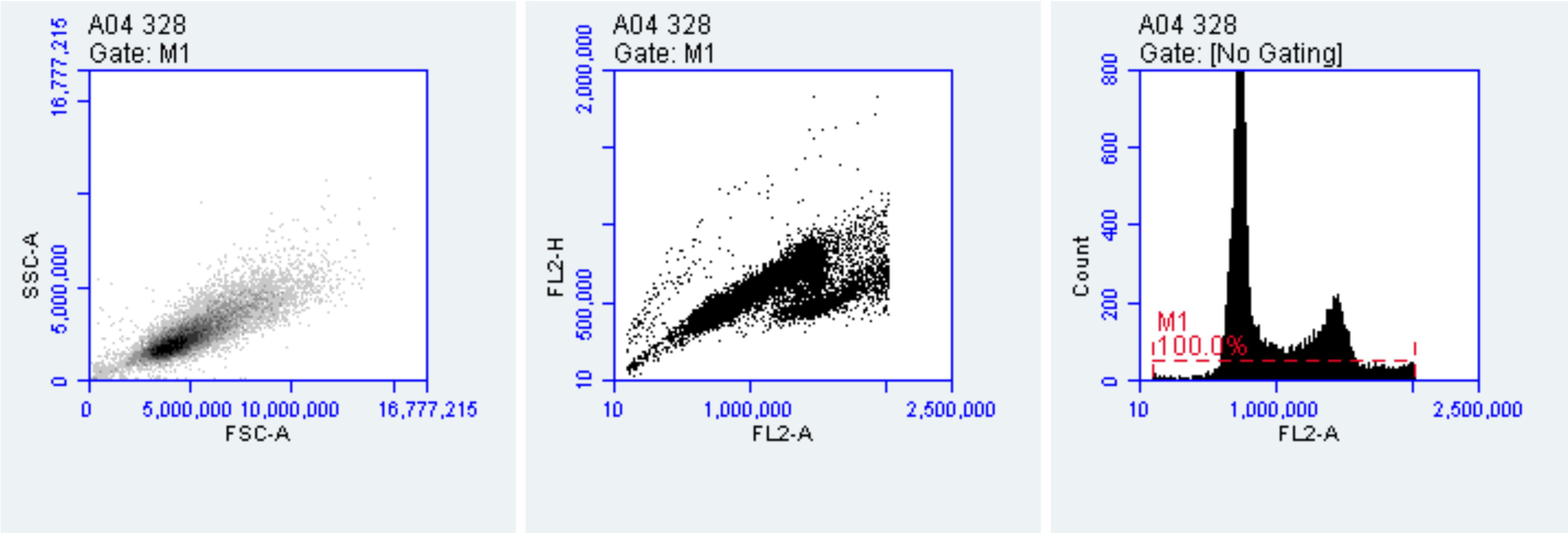

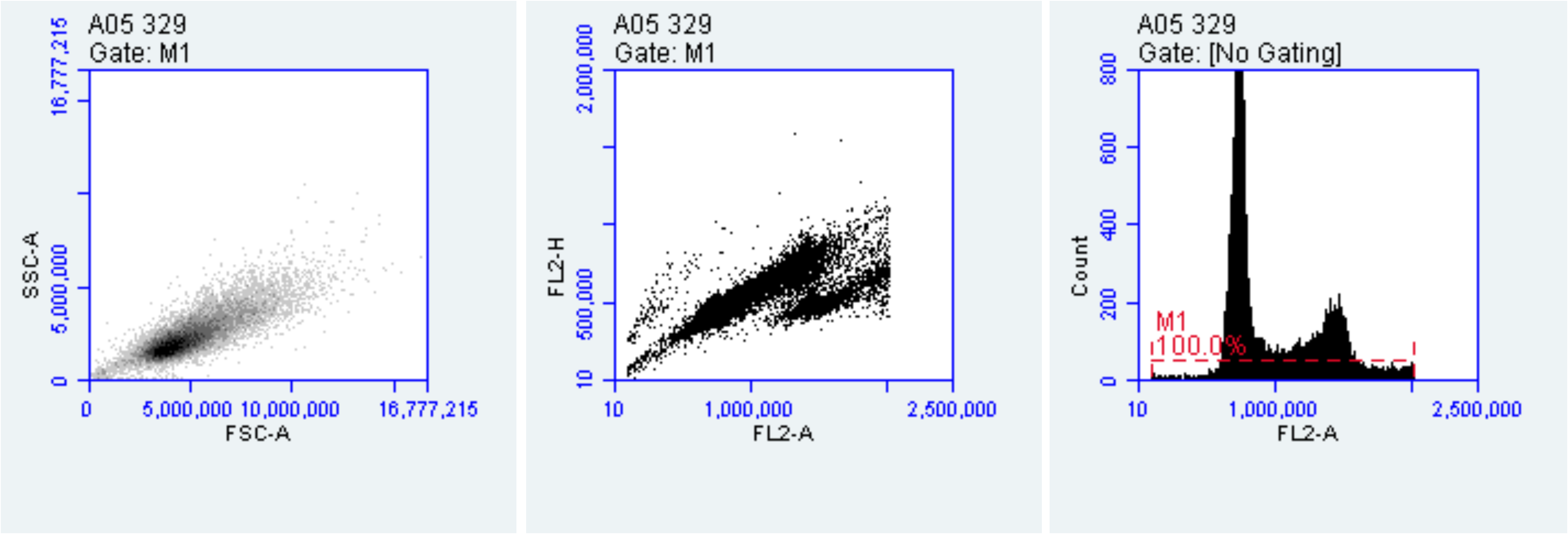

A05 329
Gate: M1
16,777,215
5,000,000
0
SSC-A
0
5,000,000
10,000,000
16,777,215
FSC-A
A05 329
Gate: M1
2,000,000
500,000
10
FL2-H
10
1,000,000
2,500,000
FL2-A
A05 329
Gate: [No Gating]
800
600
400
200
0
Count
M1
100.0%
10
1,000,000
2,500,000
FL2-A

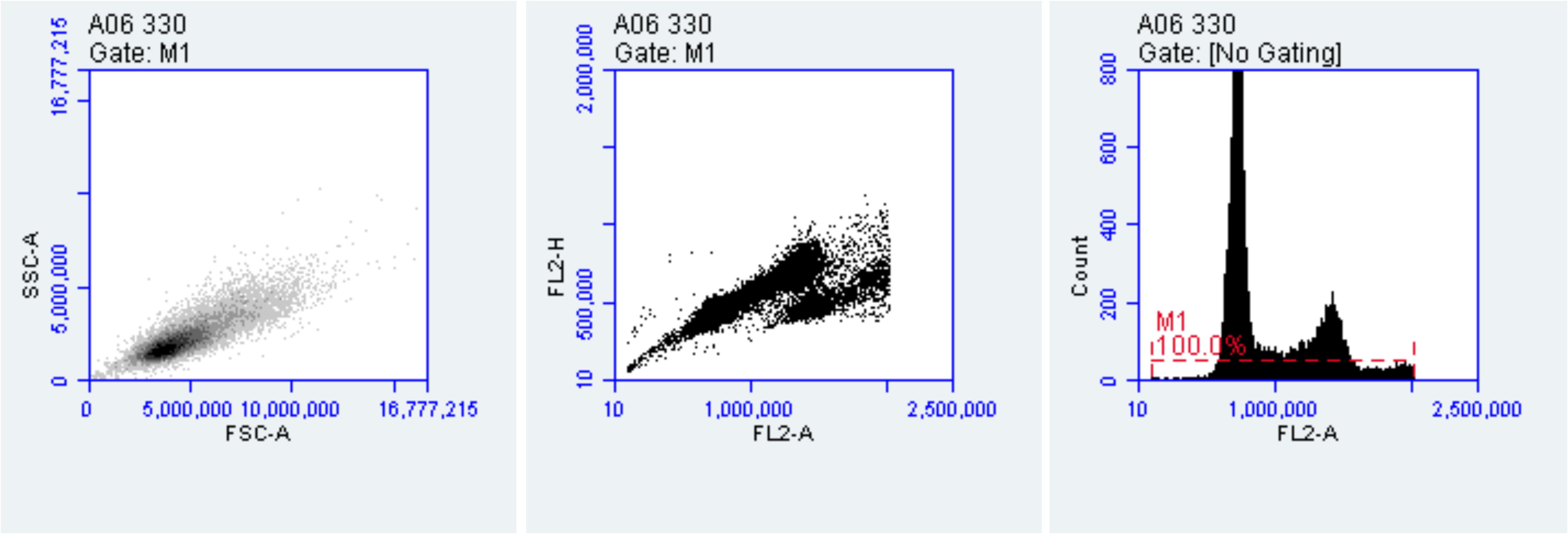
A06 330
Gate: M1
SSC-A
FSC-A
16,777,215
5,000,000
0
0
5,000,000
10,000,000
16,777,215
A06 330
Gate: M1
FL2-H
FL2-A
2,000,000
500,000
10
10
1,000,000
2,500,000
A06 330
Gate: [No Gating]
Count
FL2-A
800
600
400
200
0
10
1,000,000
2,500,000
M1
100.0%

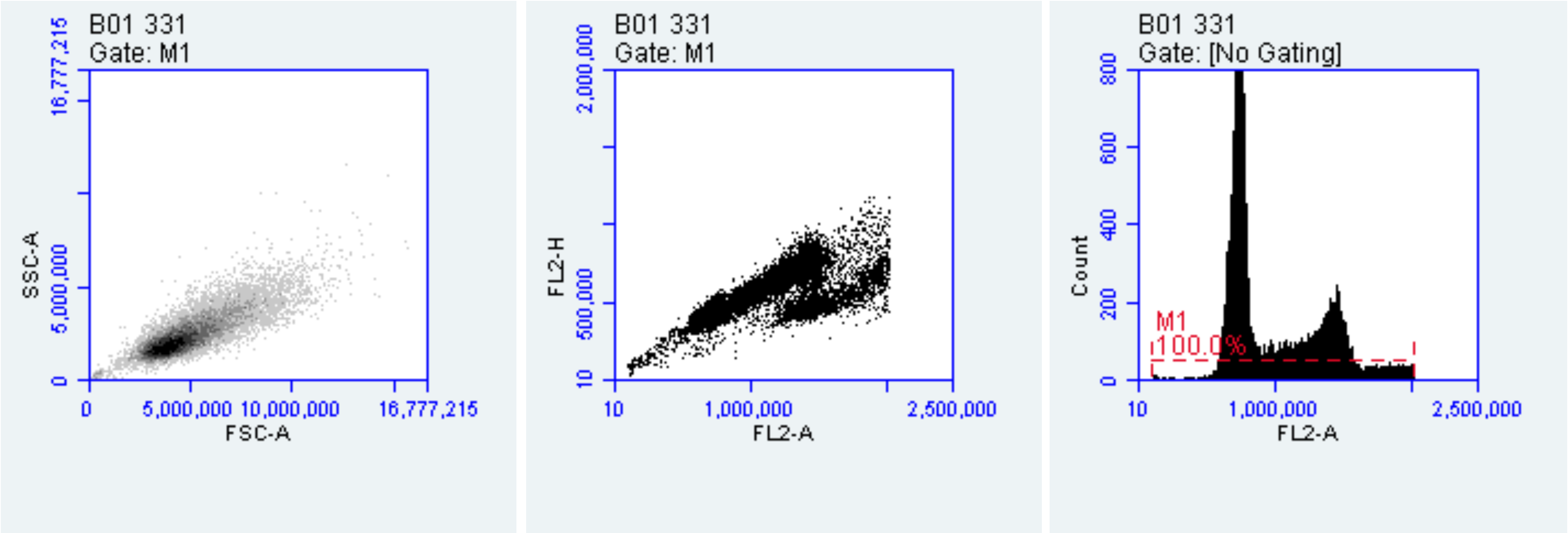
B01 331
Gate: M1
SSC-A
FSC-A
0
5,000,000
10,000,000
16,777,215
B01 331
Gate: M1
FL2-H
FL2-A
10
500,000
1,000,000
2,000,000
2,500,000
B01 331
Gate: [No Gating]
Count
FL2-A
0
200
400
600
800
10
1,000,000
2,500,000
M1
100.0%

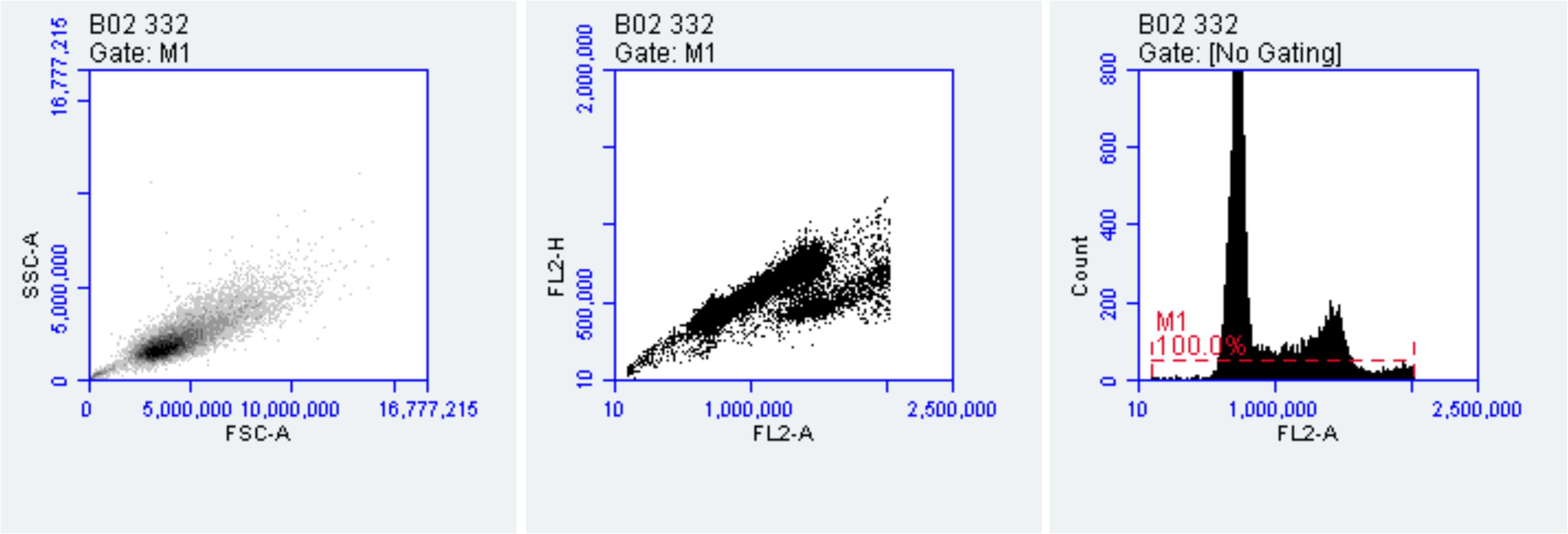
B02 332
Gate: M1
16,777,215
5,000,000
0
SSC-A
0
5,000,000
10,000,000
16,777,215
FSC-A
B02 332
Gate: M1
2,000,000
500,000
10
FL2-H
10
1,000,000
2,500,000
FL2-A
B02 332
Gate: [No Gating]
800
600
400
200
0
Count
M1
100.0%
10
1,000,000
2,500,000
FL2-A

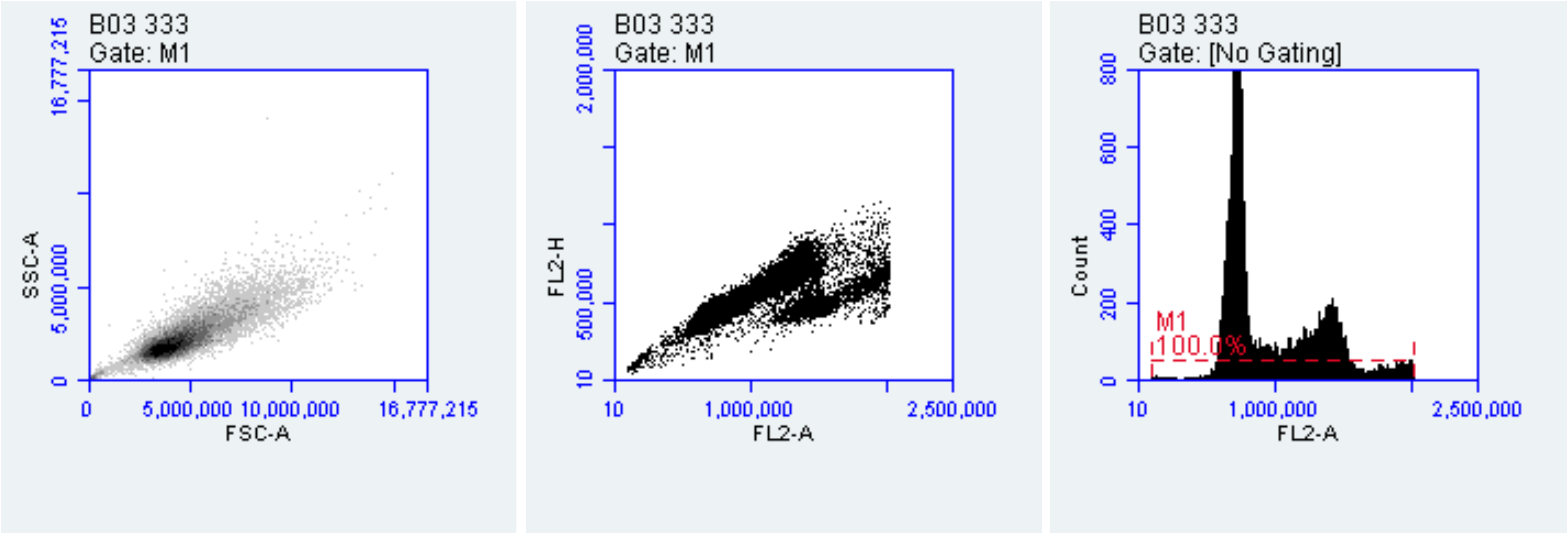
B03 333
Gate: M1
16,777,215
5,000,000
0
SSC-A
0
5,000,000
10,000,000
16,777,215
FSC-A
B03 333
Gate: M1
2,000,000
500,000
10
FL2-H
10
1,000,000
2,500,000
FL2-A
B03 333
Gate: [No Gating]
800
600
400
200
0
Count
M1
100.0%
10
1,000,000
2,500,000
FL2-A

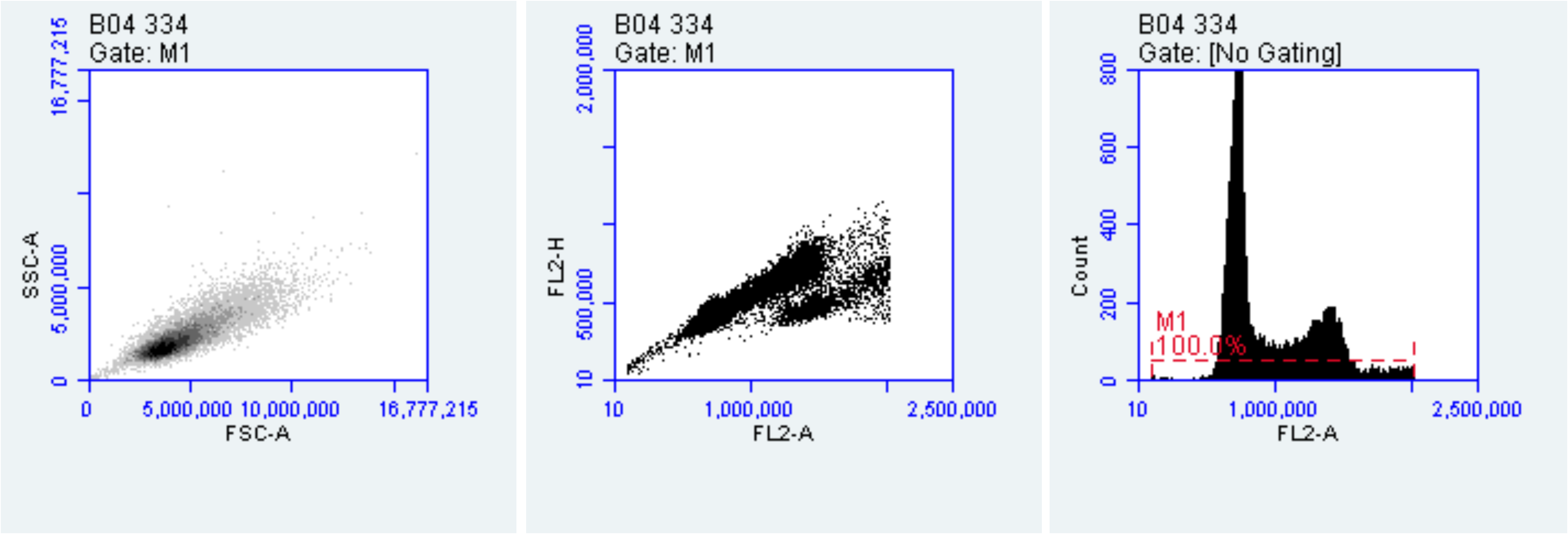
B04 334
Gate: M1
SSC-A
FSC-A
0
5,000,000
10,000,000
16,777,215
B04 334
Gate: M1
FL2-H
FL2-A
10
500,000
2,000,000
1,000,000
2,500,000
B04 334
Gate: [No Gating]
Count
0
200
400
600
800
M1
100.0%
10
1,000,000
2,500,000
FL2-A

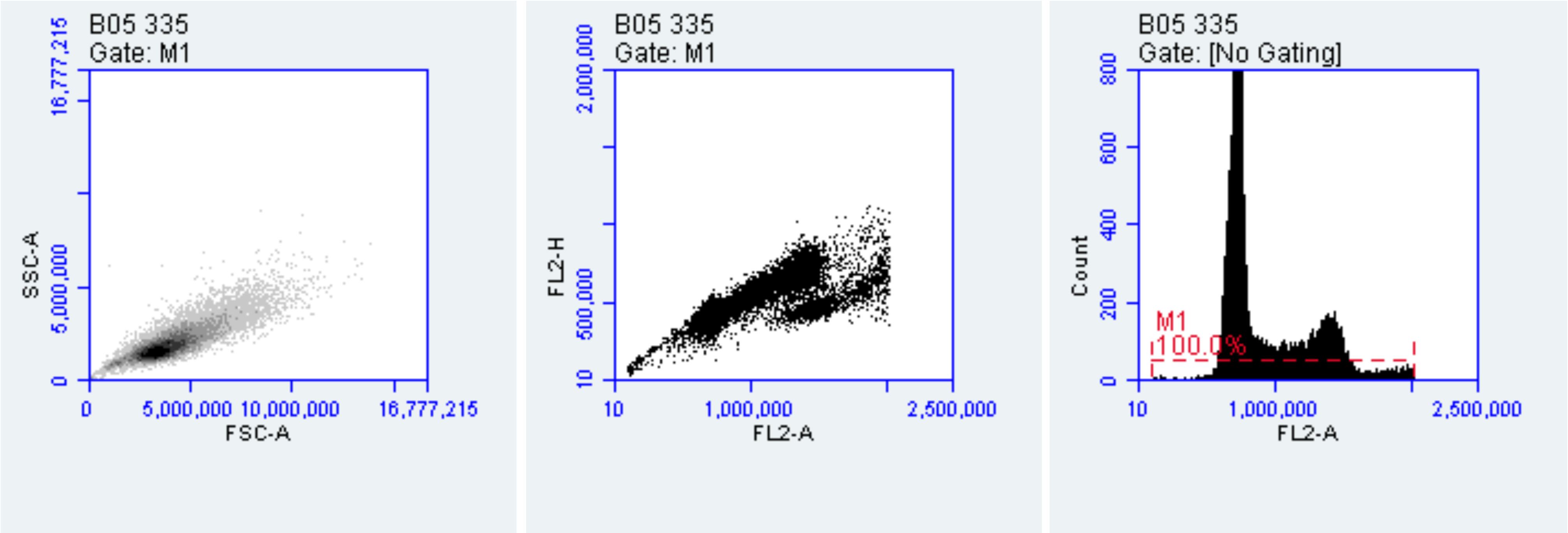
B05 335
Gate: M1
SSC-A
FSC-A
0
5,000,000
10,000,000
16,777,215
B05 335
Gate: M1
FL2-H
FL2-A
10
500,000
1,000,000
2,000,000
2,500,000
B05 335
Gate: [No Gating]
Count
FL2-A
0
200
400
600
800
10
1,000,000
2,500,000
M1
100.0%

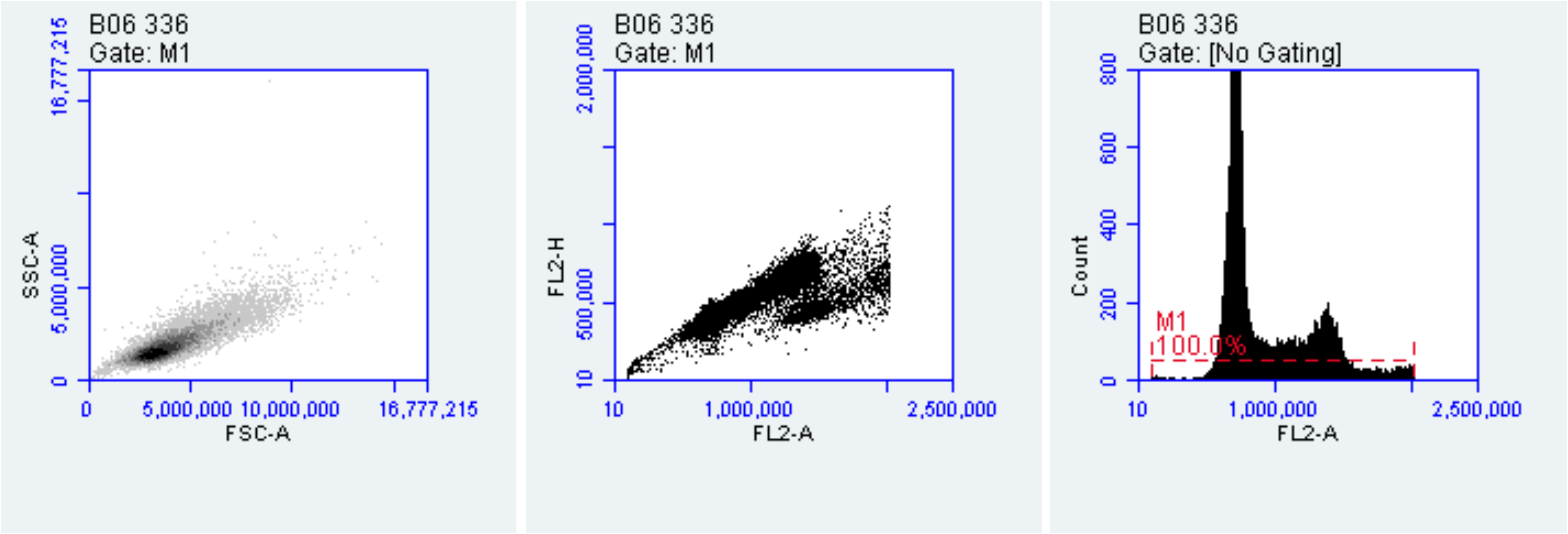

B06 336
Gate: M1
SSC-A
FSC-A
0
5,000,000
10,000,000
16,777,215
B06 336
Gate: M1
FL2-H
FL2-A
10
500,000
2,000,000
1,000,000
2,500,000
B06 336
Gate: [No Gating]
Count
0
200
400
600
800
M1
100.0%
10
1,000,000
2,500,000
FL2-A

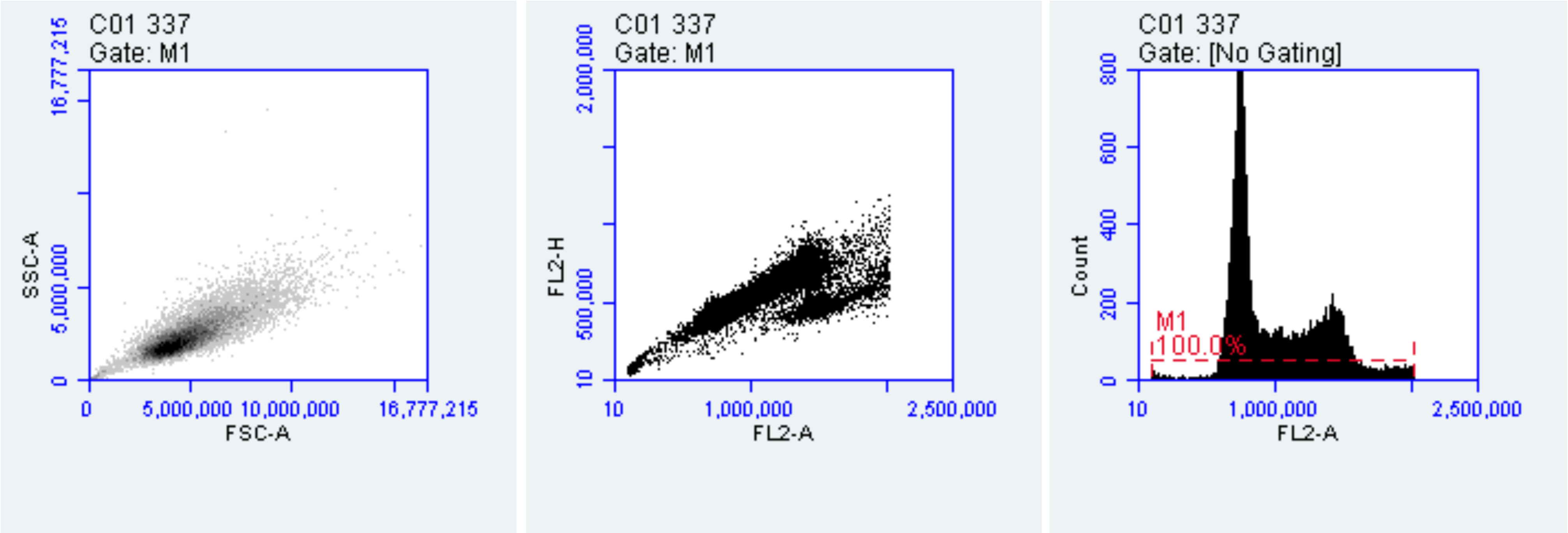
C01 337
Gate: M1
SSC-A
FSC-A
0
5,000,000
10,000,000
16,777,215
C01 337
Gate: M1
FL2-H
FL2-A
10
500,000
2,000,000
1,000,000
2,500,000
C01 337
Gate: [No Gating]
Count
FL2-A
0
200
400
600
800
M1
100.0%

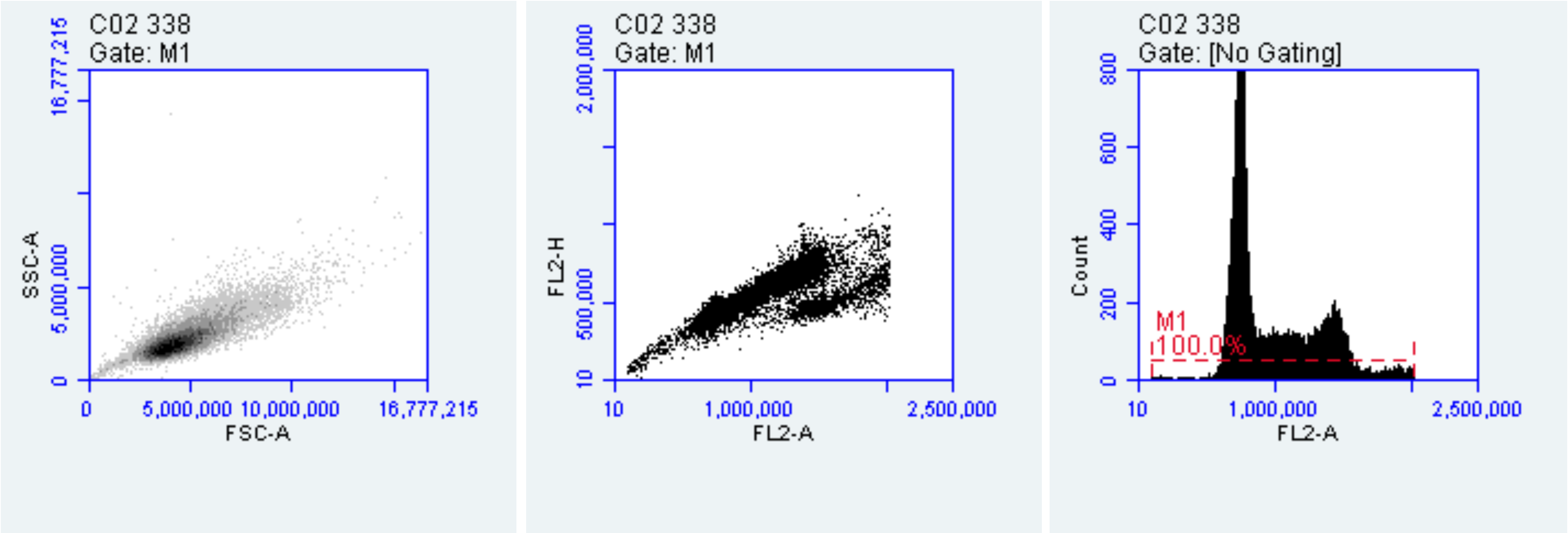

C02 338
Gate: M1
16,777,215
SSC-A
5,000,000
0
0
5,000,000
10,000,000
16,777,215
FSC-A
C02 338
Gate: M1
2,000,000
FL2-H
500,000
10
10
1,000,000
2,500,000
FL2-A
C02 338
Gate: [No Gating]
800
600
400
200
0
Count
M1
100.0%
10
1,000,000
2,500,000
FL2-A

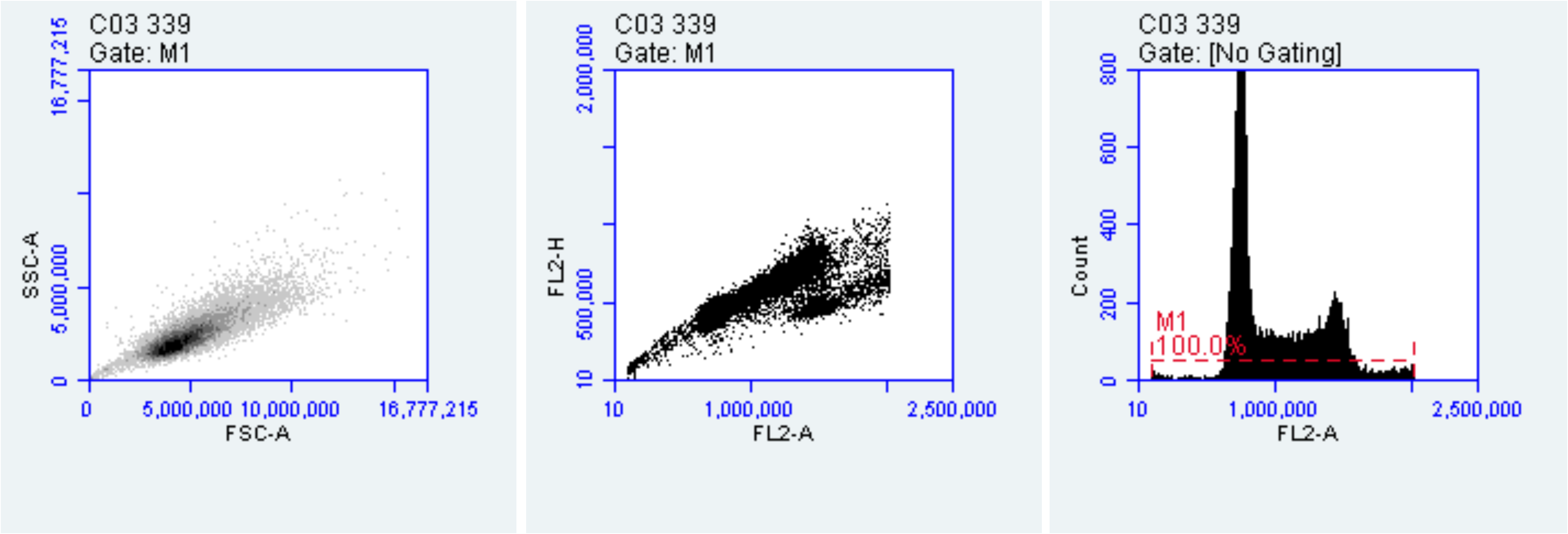
C03 339
Gate: M1
SSC-A
16,777,215
5,000,000
0
FSC-A
0
5,000,000
10,000,000
16,777,215
C03 339
Gate: M1
FL2-H
2,000,000
500,000
10
FL2-A
10
1,000,000
2,500,000
C03 339
Gate: [No Gating]
Count
800
600
400
200
0
M1
100.0%
FL2-A
10
1,000,000
2,500,000

HCC1937 Damsin Day 4

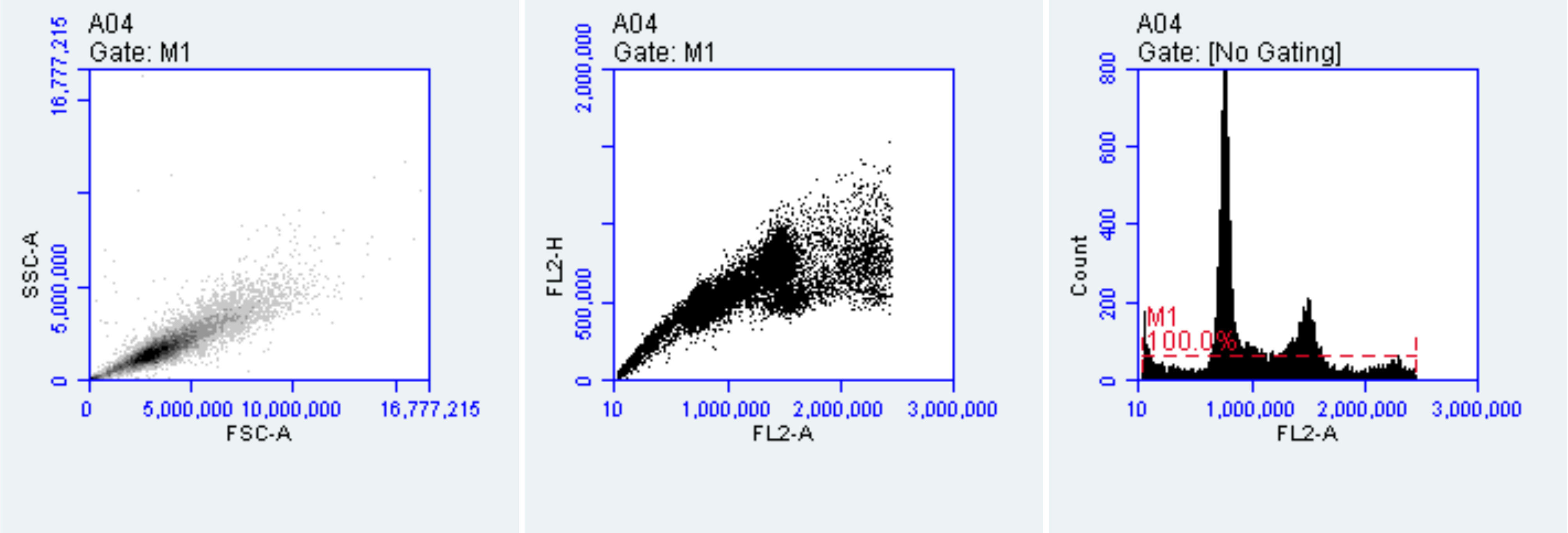

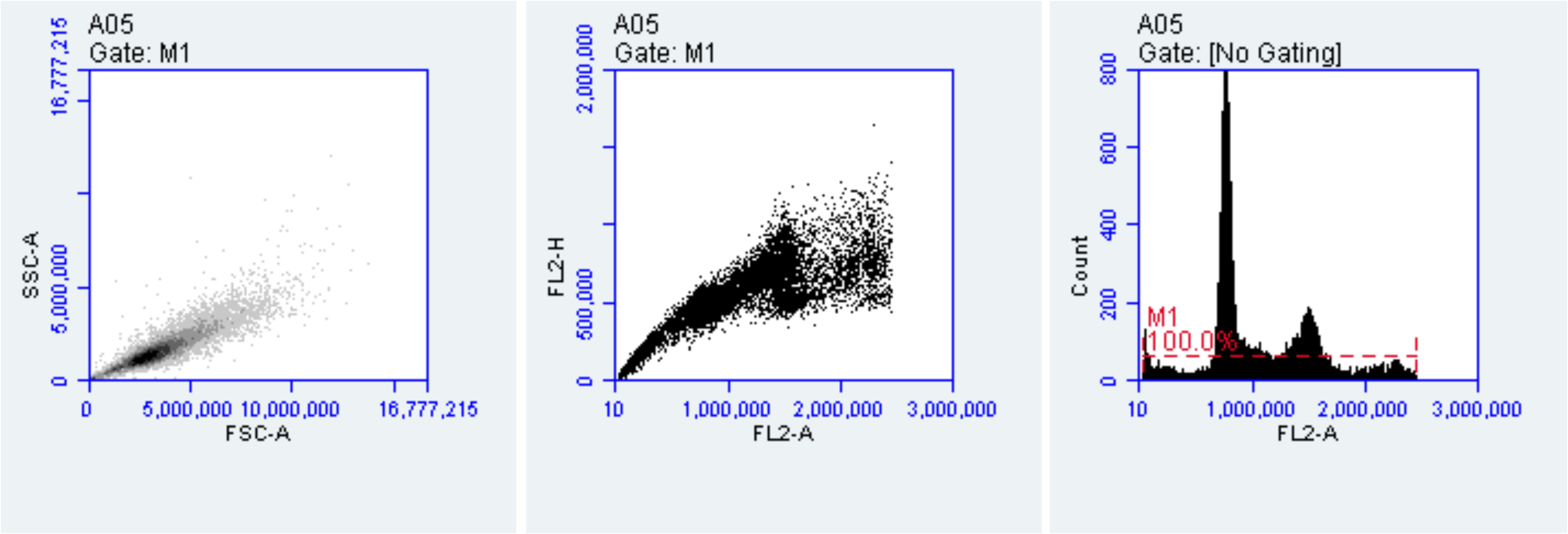
A05
Gate: M1
SSC-A
FSC-A
0
5,000,000
10,000,000
16,777,215
A05
Gate: M1
FL2-H
FL2-A
0
500,000
2,000,000
10
1,000,000
2,000,000
3,000,000
A05
Gate: [No Gating]
Count
FL2-A
0
200
400
600
800
M1
100.0%
10
1,000,000
2,000,000
3,000,000

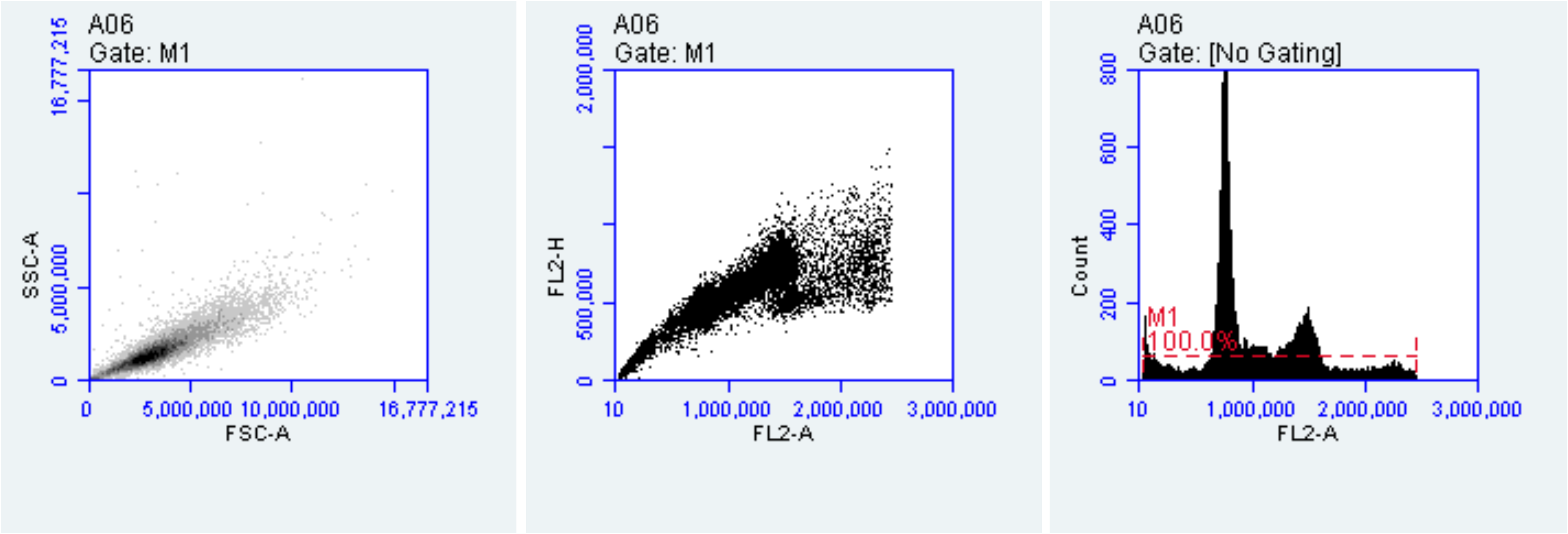
A06
Gate: M1
SSC-A
FSC-A
0
5,000,000
10,000,000
16,777,215
A06
Gate: M1
FL2-H
FL2-A
0
500,000
2,000,000
10
1,000,000
2,000,000
3,000,000
A06
Gate: [No Gating]
Count
FL2-A
0
200
400
600
800
M1
100.0%
10
1,000,000
2,000,000
3,000,000

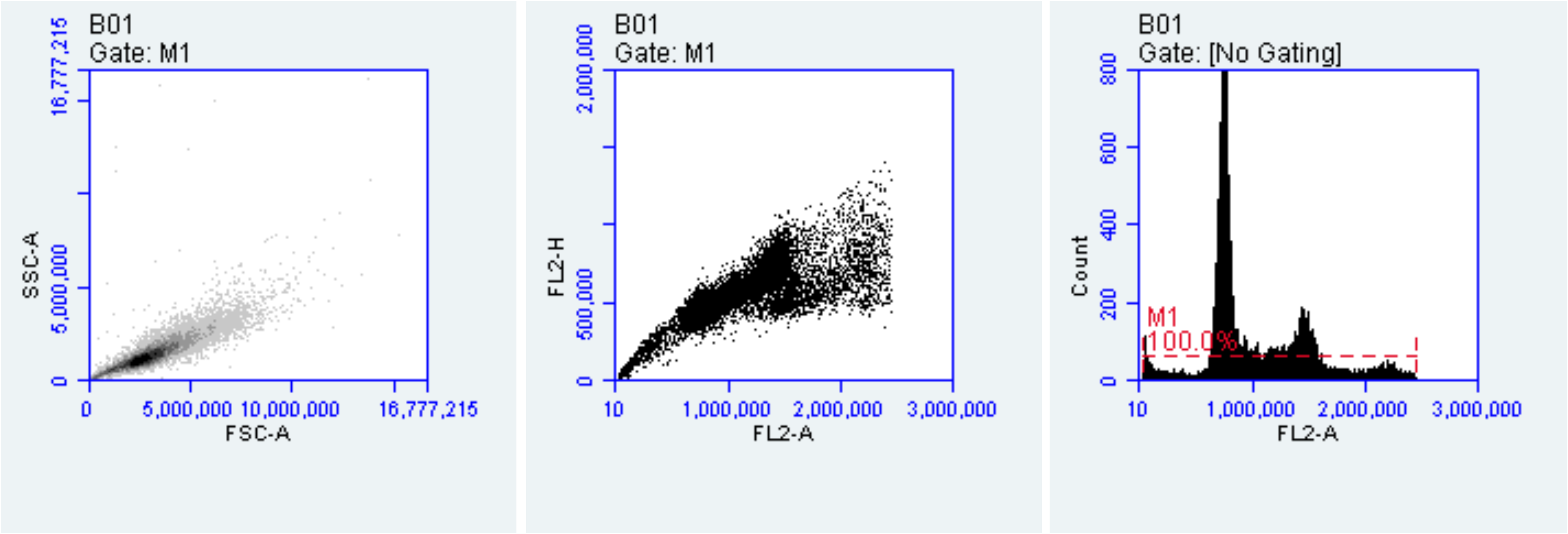
B01
Gate: M1
SSC-A
FSC-A
0
5,000,000
10,000,000
16,777,215
B01
Gate: M1
FL2-H
FL2-A
0
500,000
2,000,000
10
1,000,000
2,000,000
3,000,000
B01
Gate: [No Gating]
Count
FL2-A
0
200
400
600
800
M1
100.0%

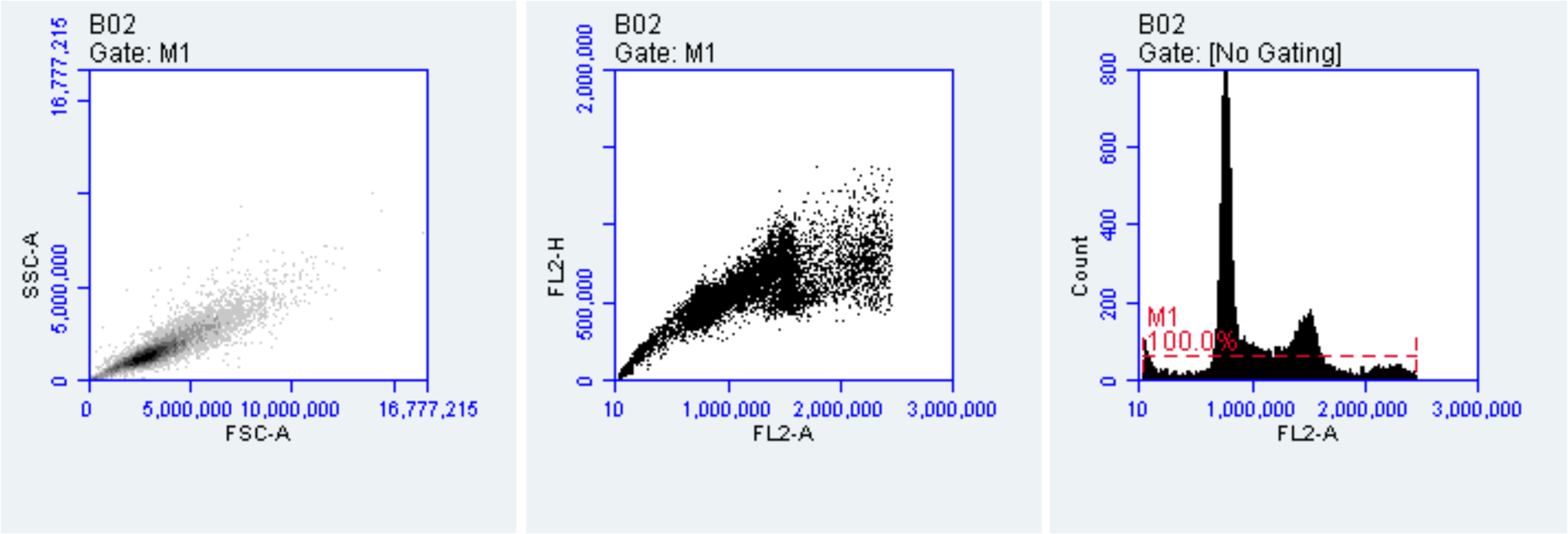

B02
Gate: M1
SSC-A
FSC-A
0
5,000,000
10,000,000
16,777,215
B02
Gate: M1
FL2-H
FL2-A
0
500,000
2,000,000
10
1,000,000
2,000,000
3,000,000
B02
Gate: [No Gating]
Count
0
200
400
600
800
M1
100.0%
10
1,000,000
2,000,000
3,000,000

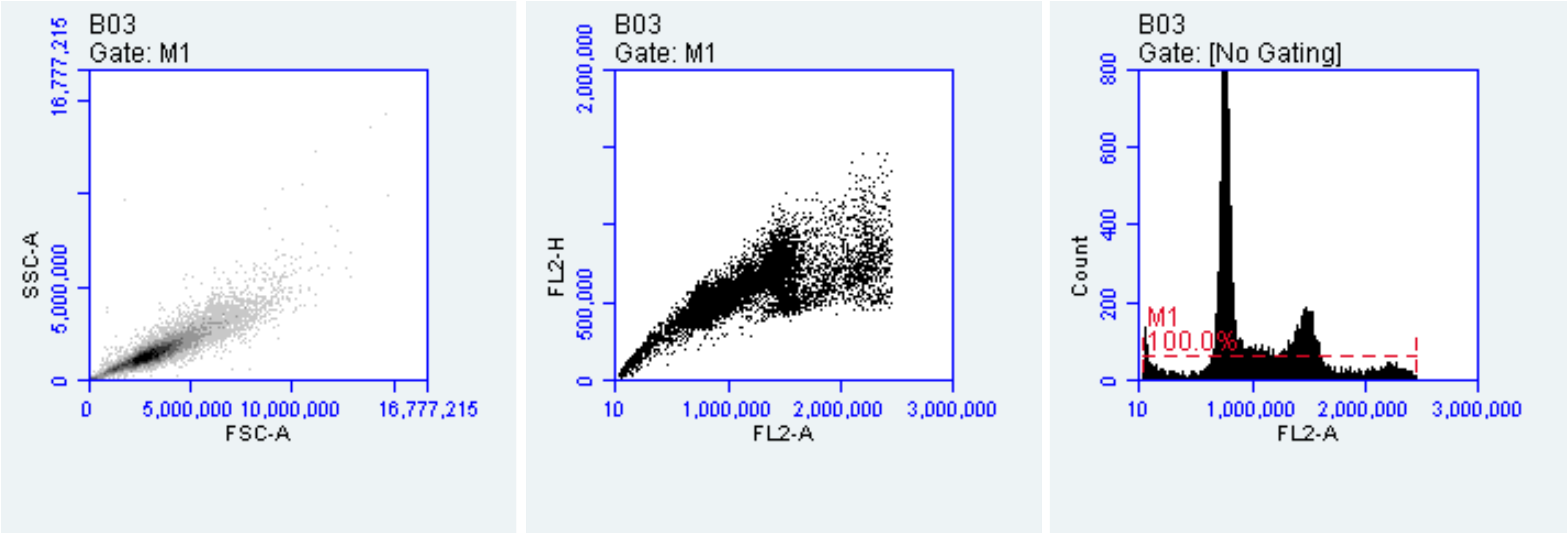
B03
Gate: M1
SSC-A
FSC-A
0
5,000,000
10,000,000
16,777,215
B03
Gate: M1
FL2-H
FL2-A
0
500,000
2,000,000
10
1,000,000
2,000,000
3,000,000
B03
Gate: [No Gating]
Count
FL2-A
0
200
400
600
800
M1
100.0%
10
1,000,000
2,000,000
3,000,000

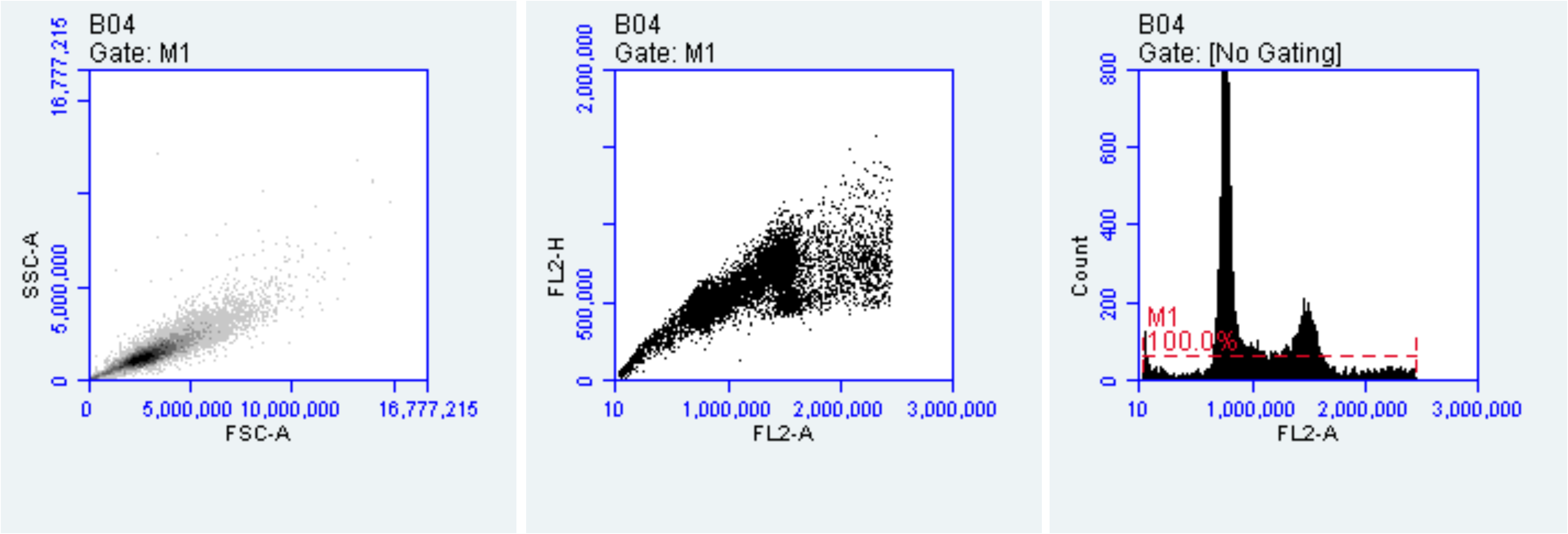

B04
Gate: M1
SSC-A
FSC-A
0
5,000,000
10,000,000
16,777,215
B04
Gate: M1
FL2-H
FL2-A
0
500,000
2,000,000
10
1,000,000
2,000,000
3,000,000
B04
Gate: [No Gating]
Count
0
200
400
600
800
M1
100.0%
FL2-A
10
1,000,000
2,000,000
3,000,000

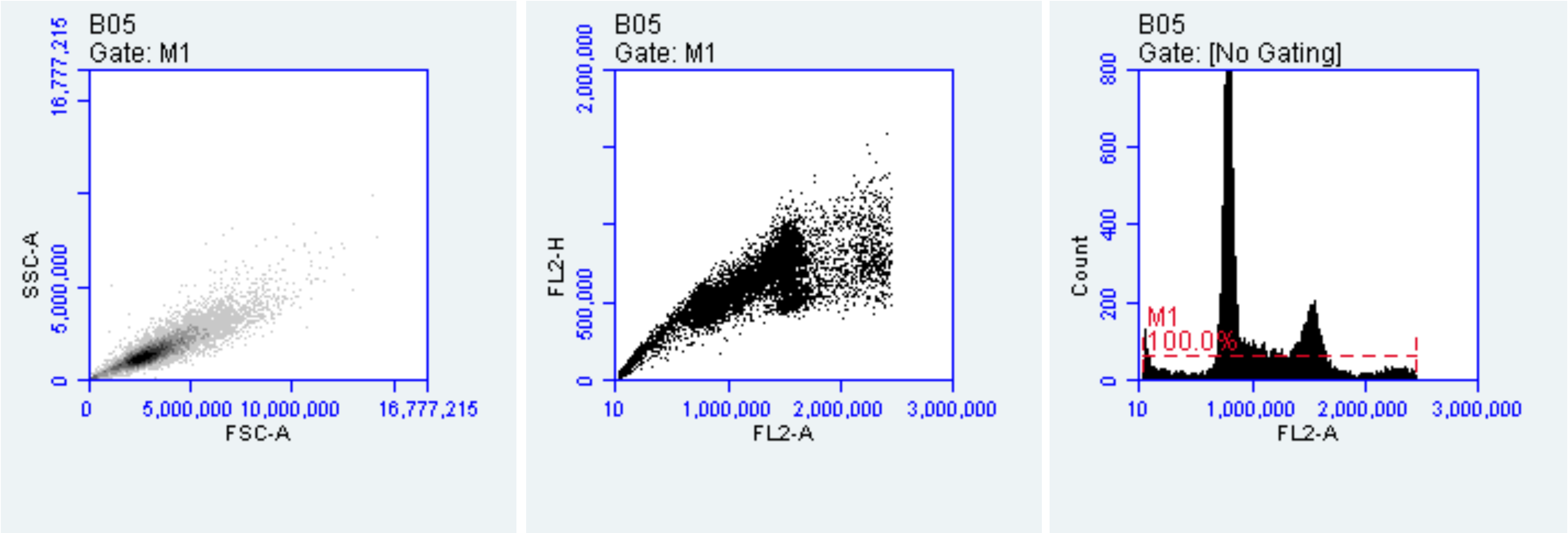
B05
Gate: M1
SSC-A
FSC-A
0
5,000,000
10,000,000
16,777,215
B05
Gate: M1
FL2-H
FL2-A
0
500,000
2,000,000
10
1,000,000
2,000,000
3,000,000
B05
Gate: [No Gating]
Count
FL2-A
M1
100.0%
0
200
400
600
800
10
1,000,000
2,000,000
3,000,000

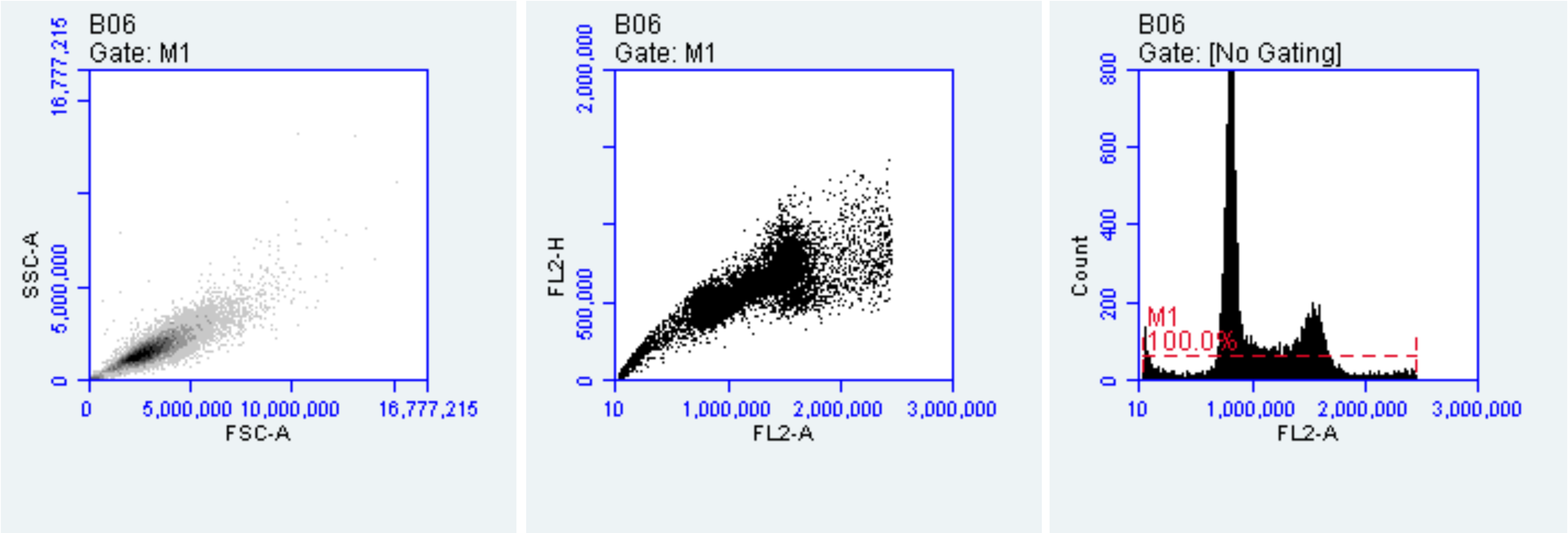
B06
Gate: M1
16,777,215
5,000,000
0
SSC-A
0
5,000,000
10,000,000
16,777,215
FSC-A
B06
Gate: M1
2,000,000
500,000
0
FL2-H
10
1,000,000
2,000,000
3,000,000
FL2-A
B06
Gate: [No Gating]
800
600
400
200
0
Count
M1
100.0%
10
1,000,000
2,000,000
3,000,000
FL2-A

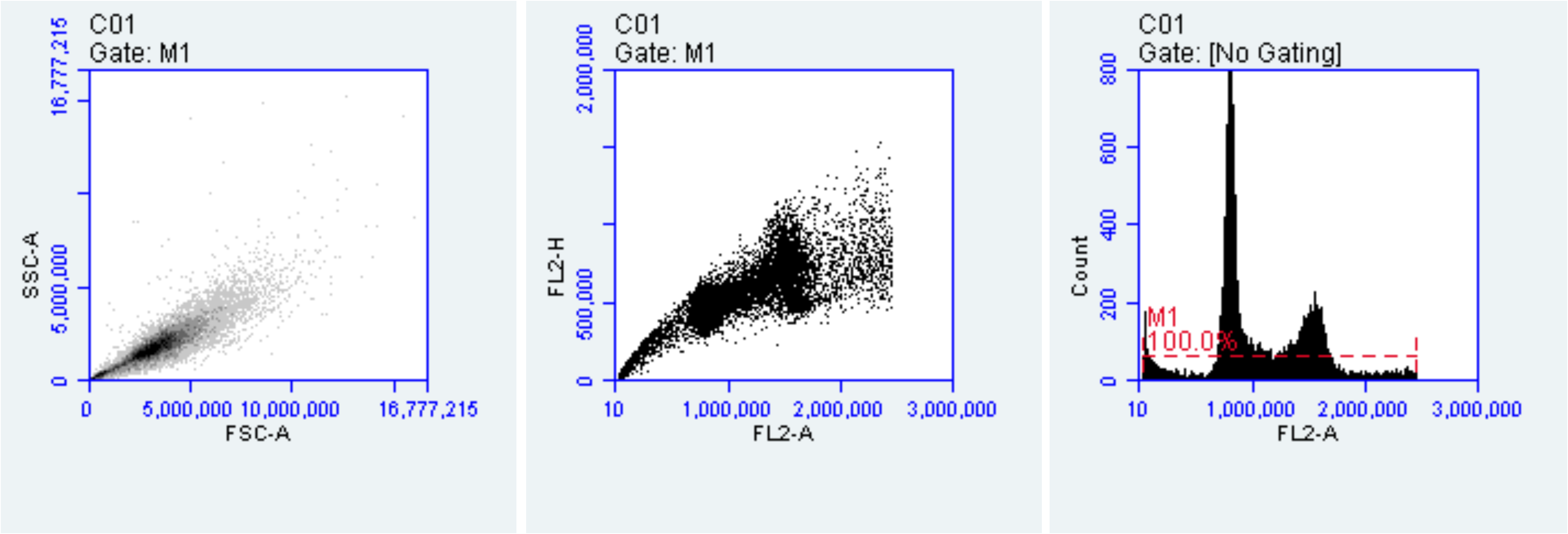
C01
Gate: M1
SSC-A
FSC-A
0
5,000,000
10,000,000
16,777,215
C01
Gate: M1
FL2-H
FL2-A
0
500,000
2,000,000
10
1,000,000
2,000,000
3,000,000
C01
Gate: [No Gating]
Count
0
200
400
600
800
M1
100.0%
10
1,000,000
2,000,000
3,000,000
FL2-A

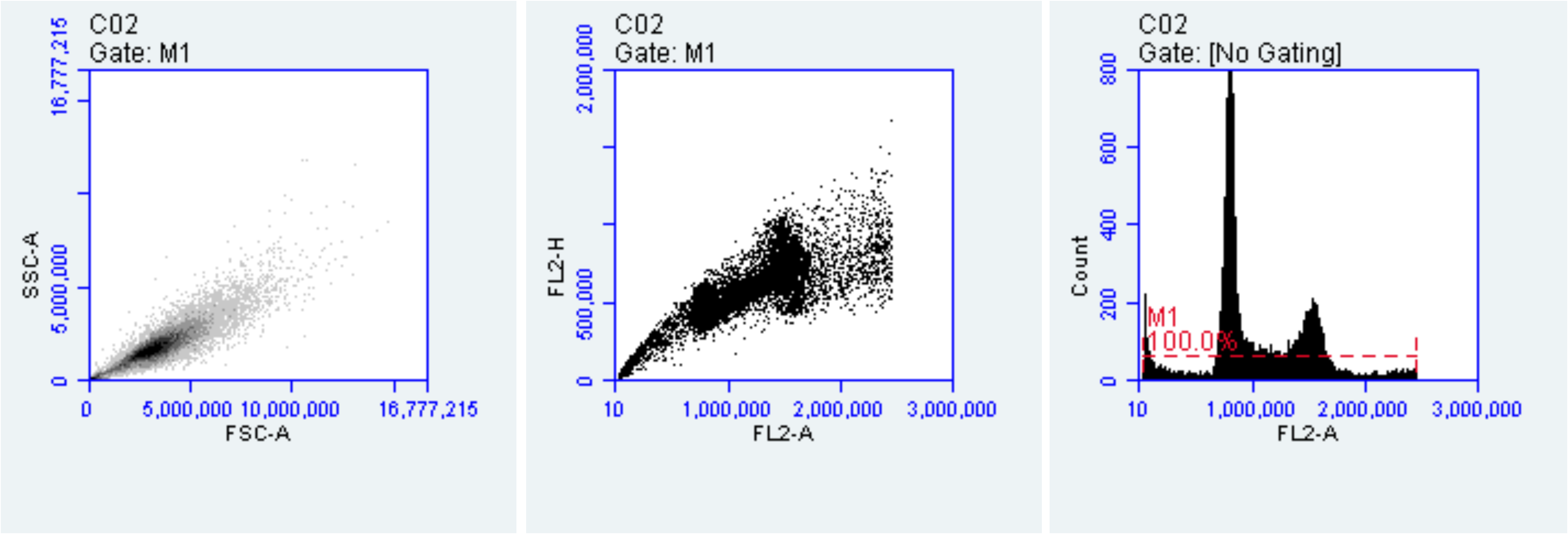
C02
Gate: M1
SSC-A
FSC-A
0
5,000,000
16,777,215
10,000,000
C02
Gate: M1
FL2-H
FL2-A
0
500,000
2,000,000
10
1,000,000
2,000,000
3,000,000
C02
Gate: [No Gating]
Count
FL2-A
M1
100.0%
0
200
400
600
800
10
1,000,000
2,000,000
3,000,000

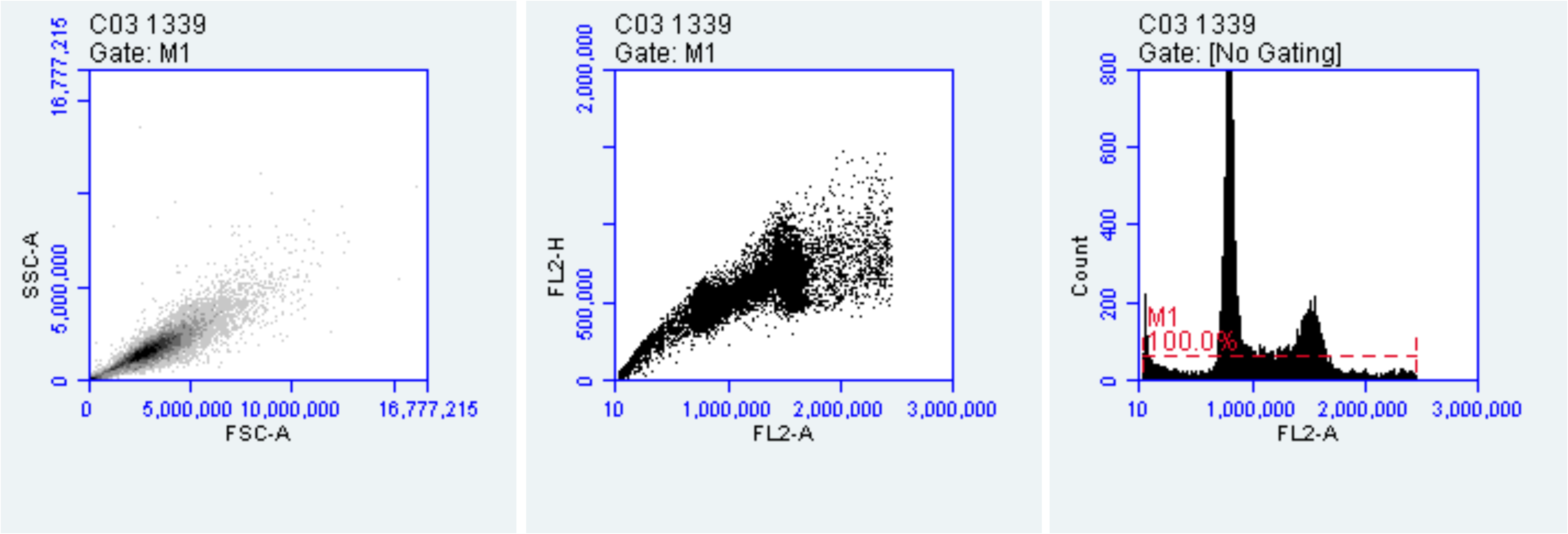
C03 1339
Gate: M1
SSC-A
FSC-A
0
5,000,000
10,000,000
16,777,215
C03 1339
Gate: M1
FL2-H
FL2-A
0
500,000
2,000,000
10
1,000,000
2,000,000
3,000,000
C03 1339
Gate: [No Gating]
Count
FL2-A
0
200
400
600
800
10
1,000,000
2,000,000
3,000,000
M1
100.0%

MCF-10A Ambrosin Day 4

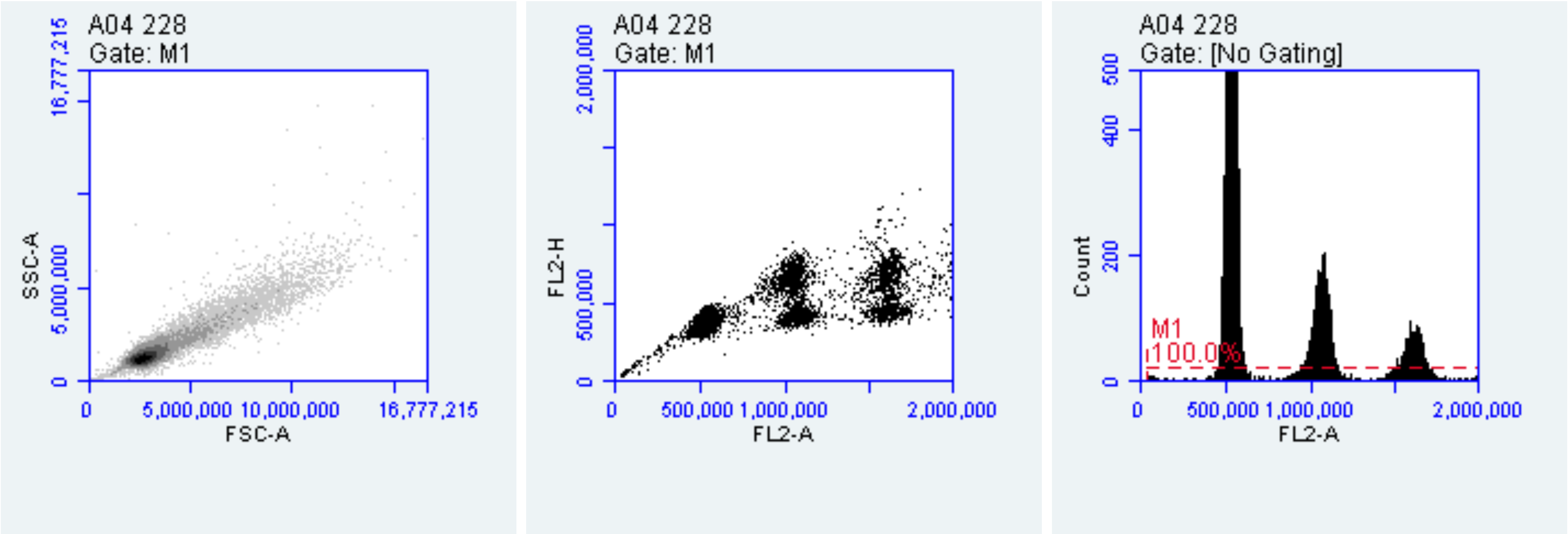

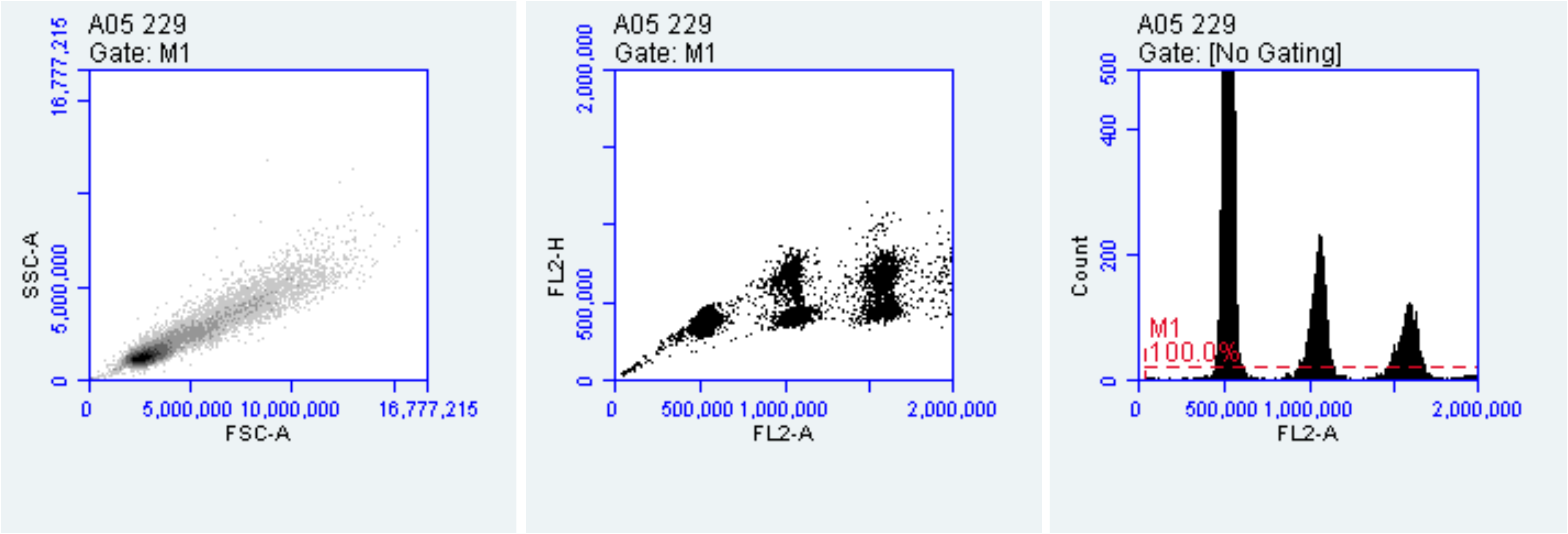
A05 229
Gate: M1
SSC-A
FSC-A
0
5,000,000
10,000,000
16,777,215
A05 229
Gate: M1
FL2-H
FL2-A
0
500,000
2,000,000
500,000
1,000,000
2,000,000
A05 229
Gate: [No Gating]
Count
0
200
400
500
M1
100.0%
FL2-A
0
500,000
1,000,000
2,000,000

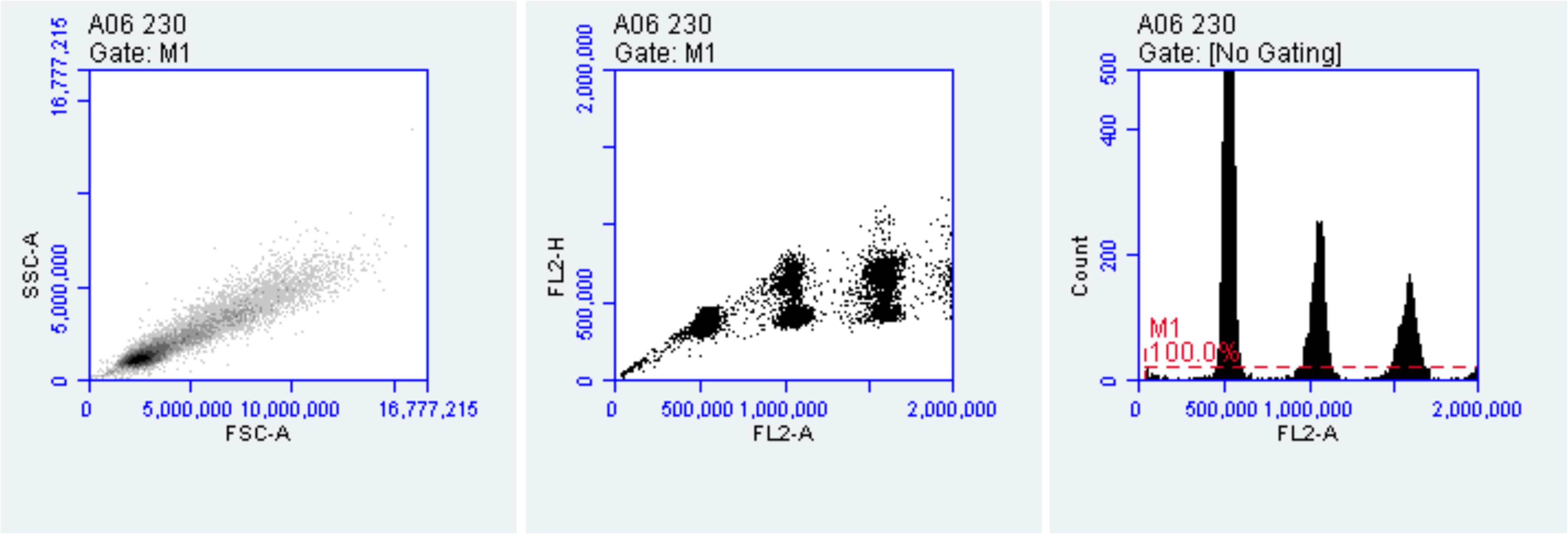
A06 230
Gate: M1
SSC-A
FSC-A
0
5,000,000
10,000,000
16,777,215
A06 230
Gate: M1
FL2-H
FL2-A
0
500,000
1,000,000
2,000,000
A06 230
Gate: [No Gating]
Count
FL2-A
M1
100.0%
0
200
400
500
500,000
1,000,000
2,000,000

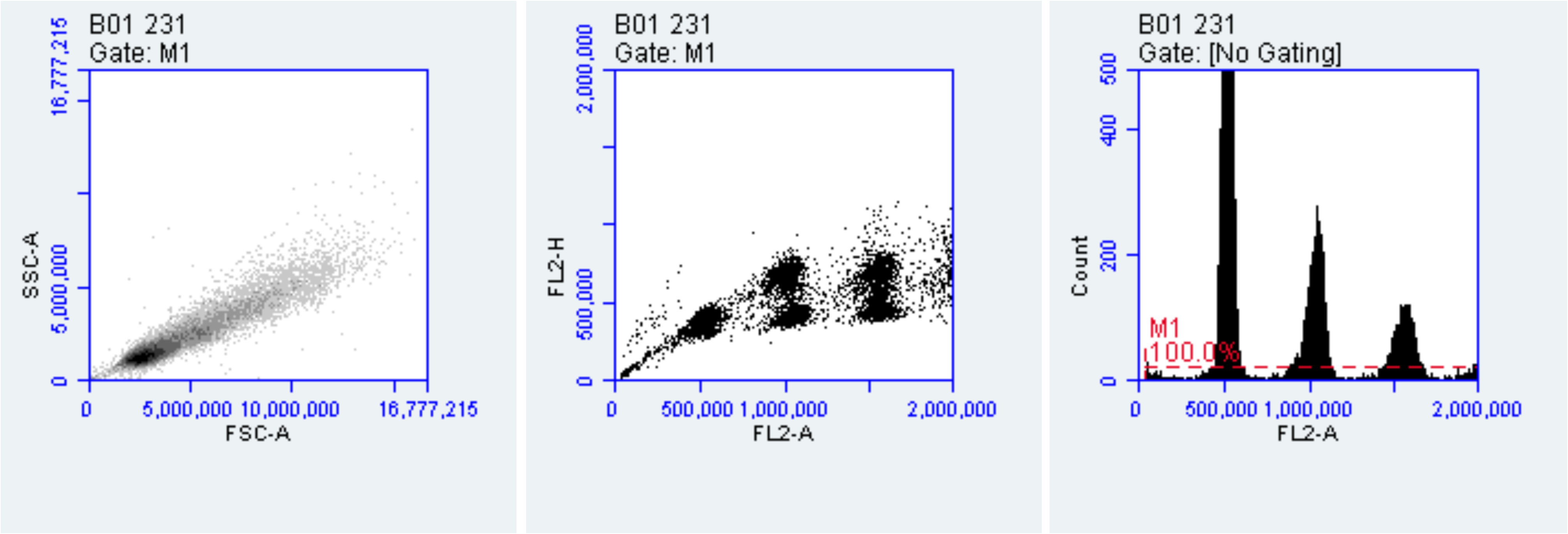

B01 231
Gate: M1
SSC-A
FSC-A
0
5,000,000
10,000,000
16,777,215
B01 231
Gate: M1
FL2-H
FL2-A
0
500,000
1,000,000
2,000,000
B01 231
Gate: [No Gating]
Count
FL2-A
M1
100.0%
0
200
400
500

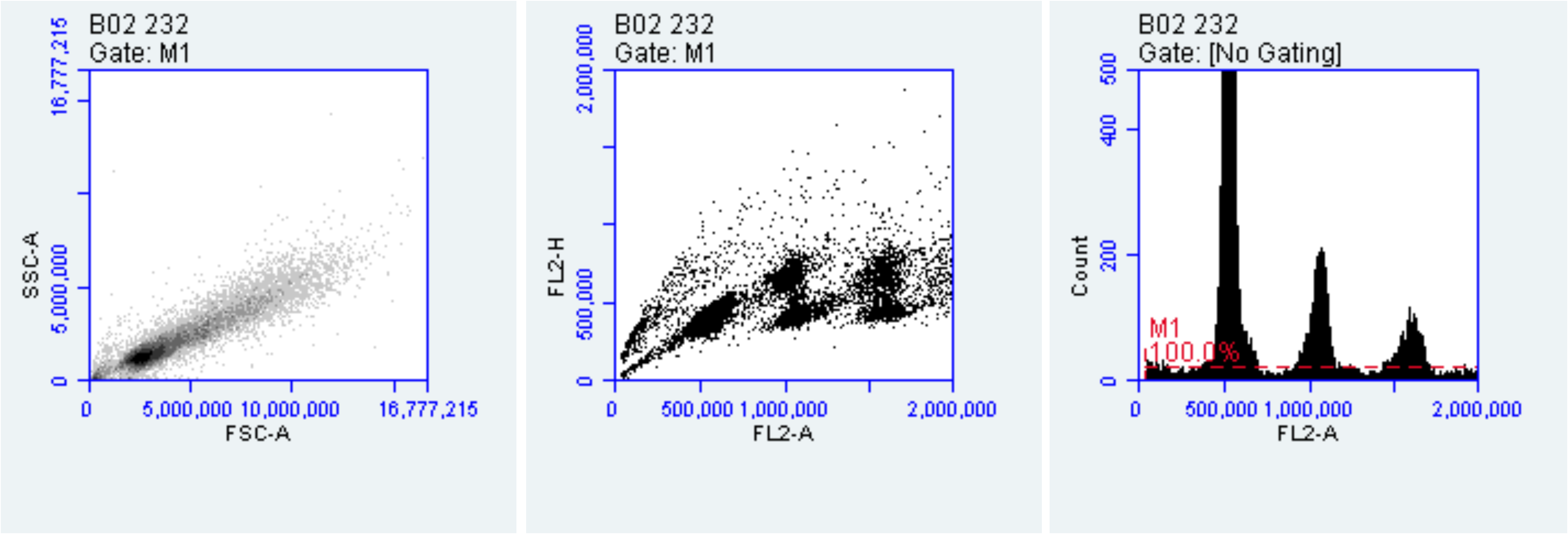
B02 232
Gate: M1
SSC-A
FSC-A
0
5,000,000
10,000,000
16,777,215
B02 232
Gate: M1
FL2-H
FL2-A
0
500,000
1,000,000
2,000,000
B02 232
Gate: [No Gating]
Count
FL2-A
0
200
400
500
500,000
1,000,000
2,000,000
M1
100.0%

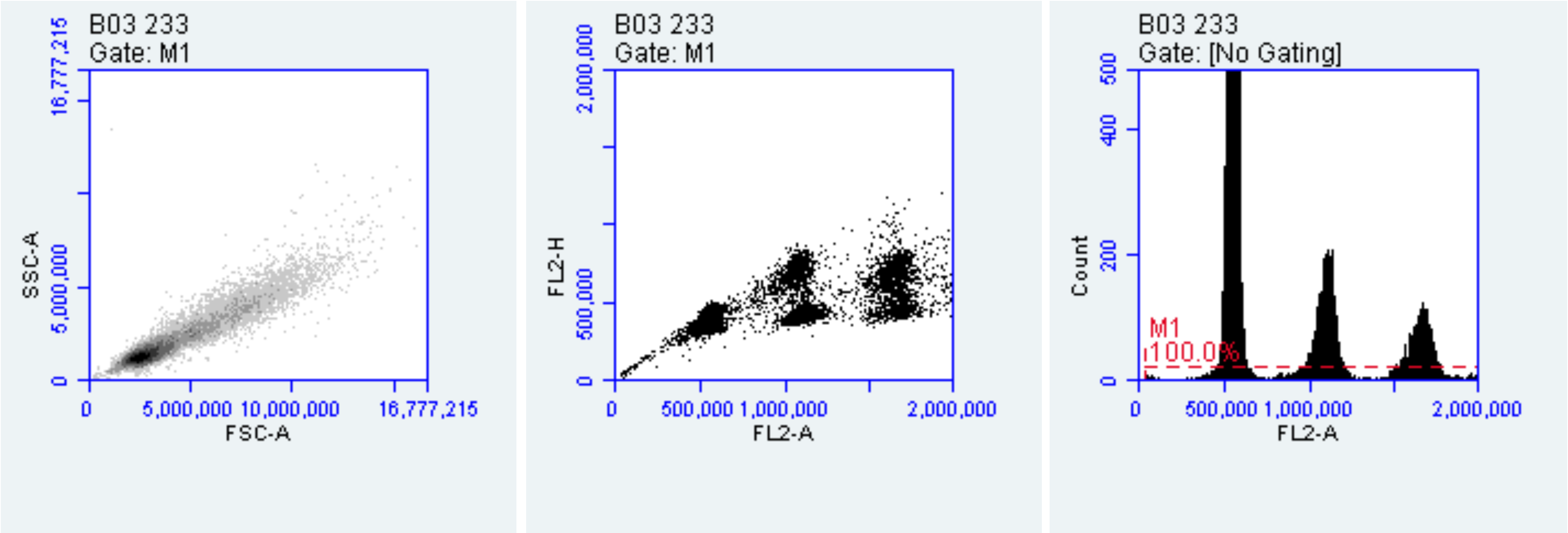
B03 233
Gate: M1
SSC-A
FSC-A
0
5,000,000
10,000,000
16,777,215
B03 233
Gate: M1
FL2-H
FL2-A
0
500,000
1,000,000
2,000,000
B03 233
Gate: [No Gating]
Count
FL2-A
M1
100.0%
0
200
400
500
0
500,000
1,000,000
2,000,000

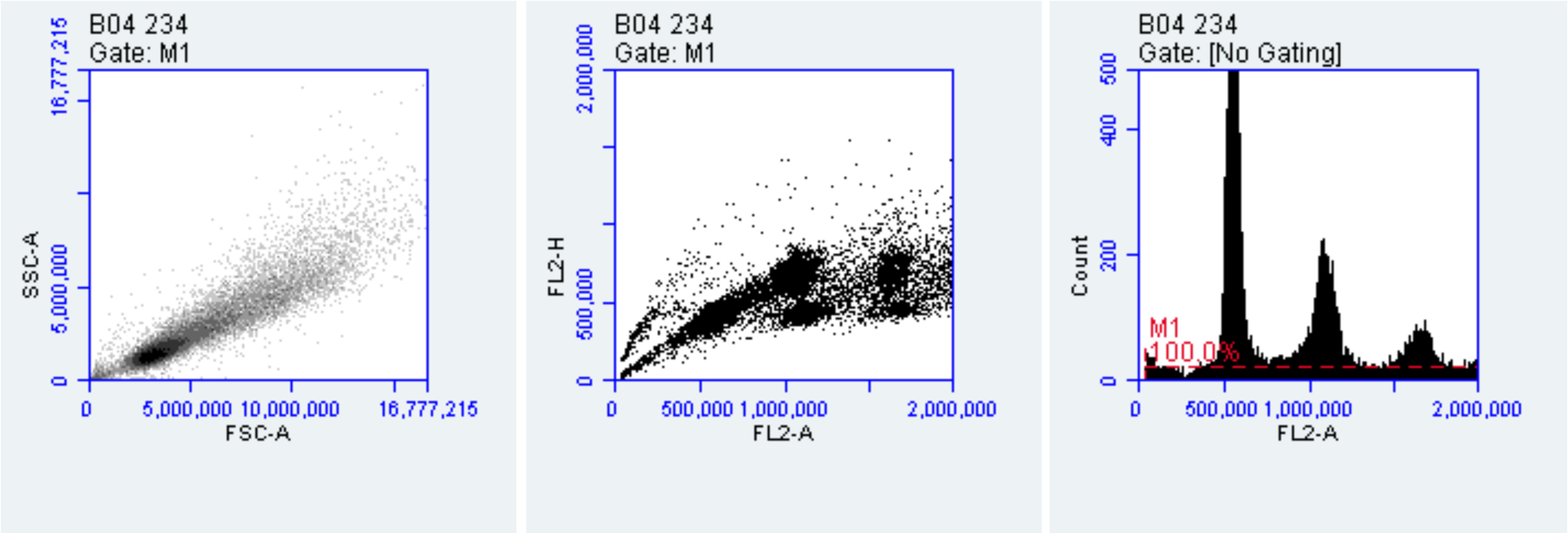
B04 234
Gate: M1
SSC-A
16,777,215
5,000,000
0
FSC-A
0
5,000,000
10,000,000
16,777,215
B04 234
Gate: M1
FL2-H
2,000,000
500,000
0
FL2-A
0
500,000
1,000,000
2,000,000
B04 234
Gate: [No Gating]
Count
500
400
200
0
M1
100.0%
FL2-A
0
500,000
1,000,000
2,000,000

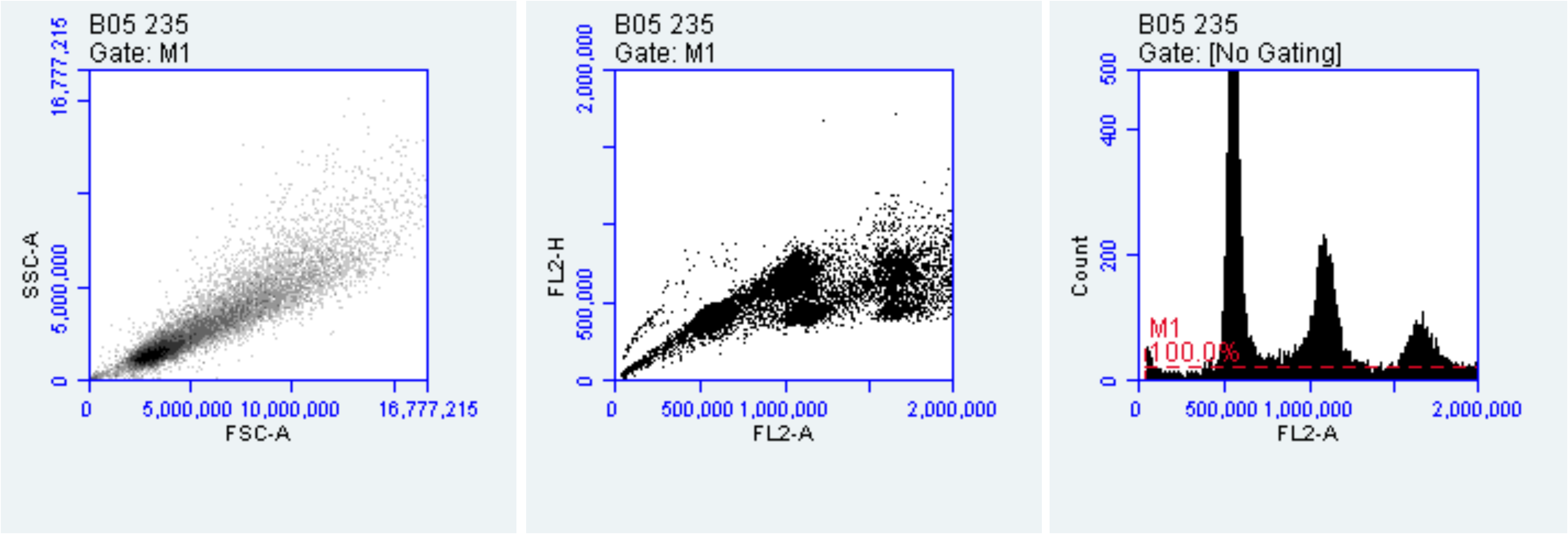
B05 235
Gate: M1
SSC-A
FSC-A
0
5,000,000
10,000,000
16,777,215
B05 235
Gate: M1
FL2-H
FL2-A
0
500,000
1,000,000
2,000,000
B05 235
Gate: [No Gating]
Count
FL2-A
M1
100.0%
0
200
400
500
500,000
1,000,000
2,000,000

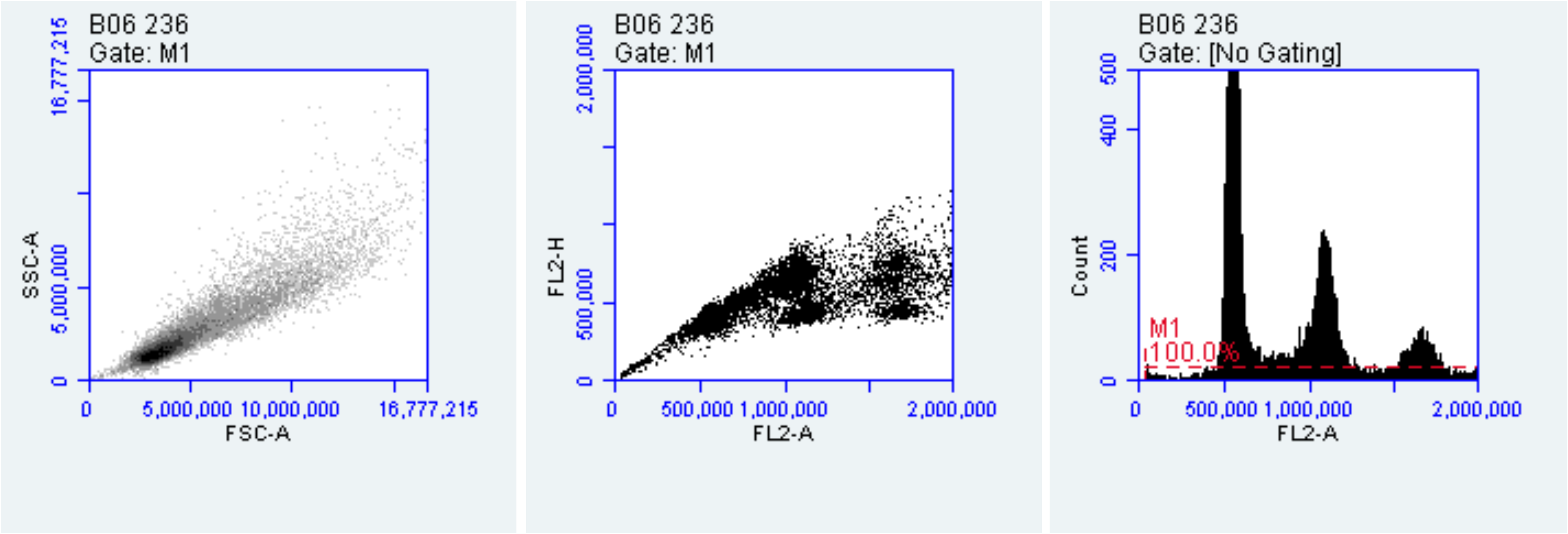
B06 236
Gate: M1
SSC-A
FSC-A
0
5,000,000
10,000,000
16,777,215
B06 236
Gate: M1
FL2-H
FL2-A
0
500,000
1,000,000
2,000,000
B06 236
Gate: [No Gating]
Count
FL2-A
0
200
400
500
M1
100.0%
500,000
1,000,000
2,000,000

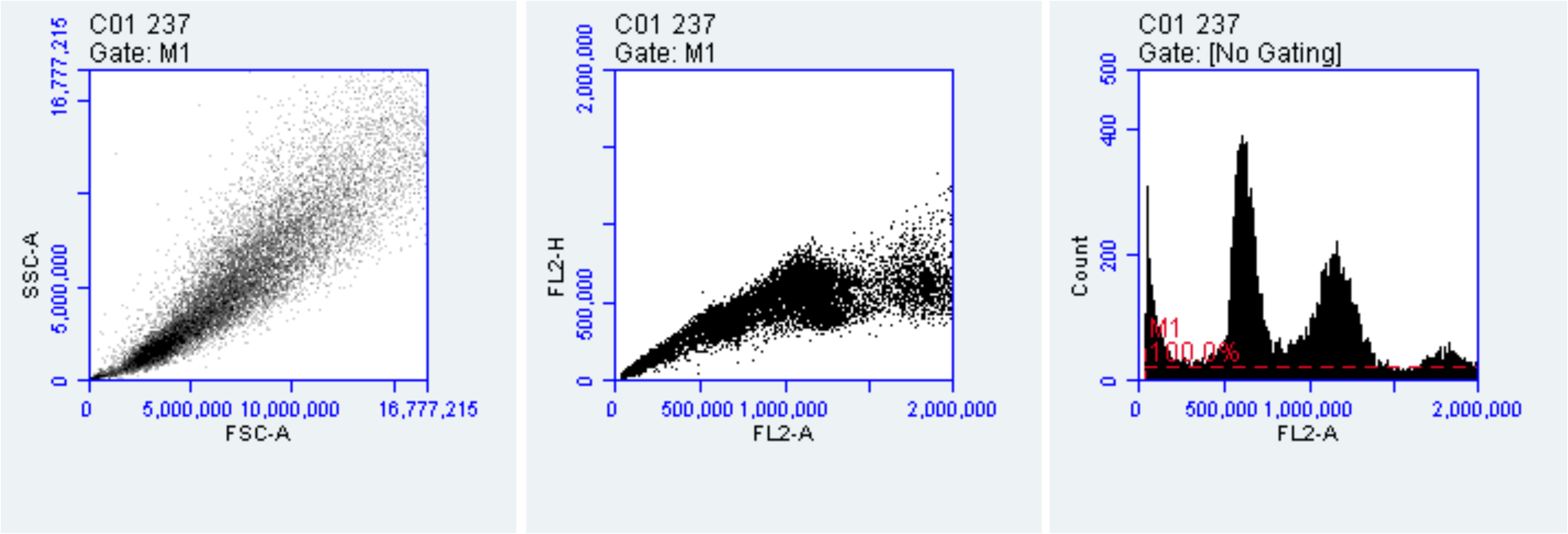
C01 237
Gate: M1
SSC-A
FSC-A
0
5,000,000
10,000,000
16,777,215
C01 237
Gate: M1
FL2-H
FL2-A
0
500,000
1,000,000
2,000,000
C01 237
Gate: [No Gating]
Count
FL2-A
0
200
400
500
500,000
1,000,000
2,000,000
M1
100.0%

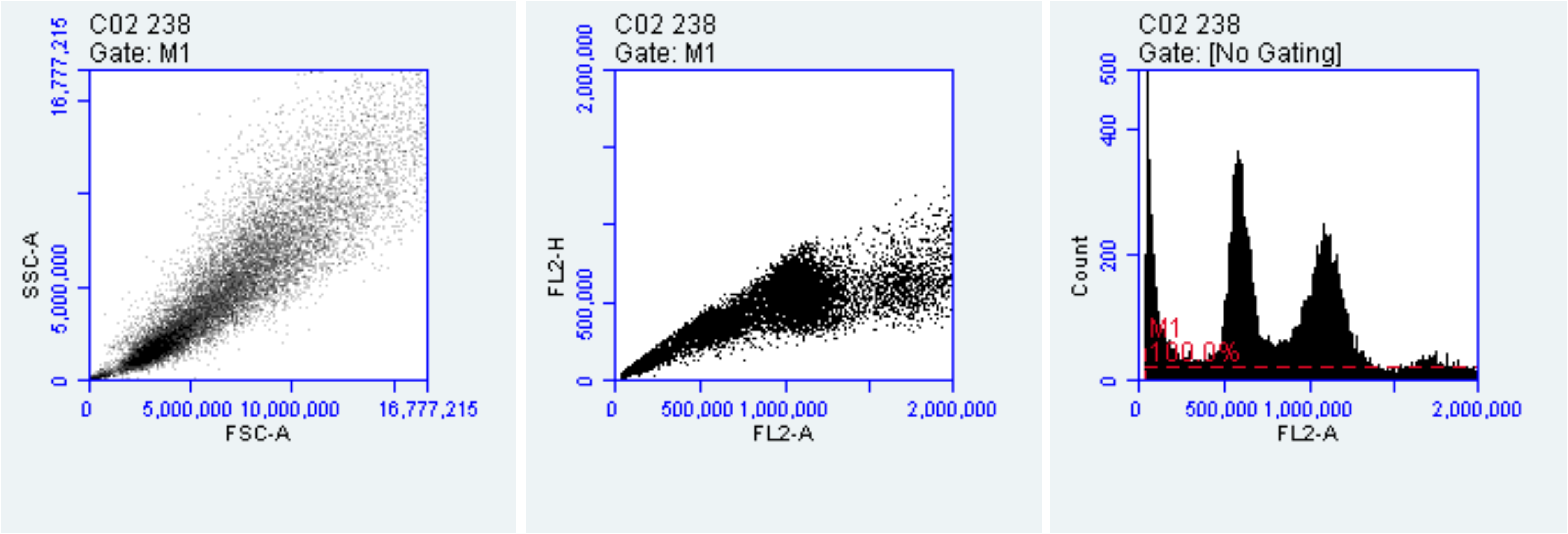
C02 238
Gate: M1
SSC-A
FSC-A
0
5,000,000
16,777,215
0
5,000,000
10,000,000
16,777,215
C02 238
Gate: M1
FL2-H
FL2-A
0
500,000
2,000,000
0
500,000
1,000,000
2,000,000
C02 238
Gate: [No Gating]
Count
FL2-A
0
200
400
500
0
500,000
1,000,000
2,000,000
M1
100.0%

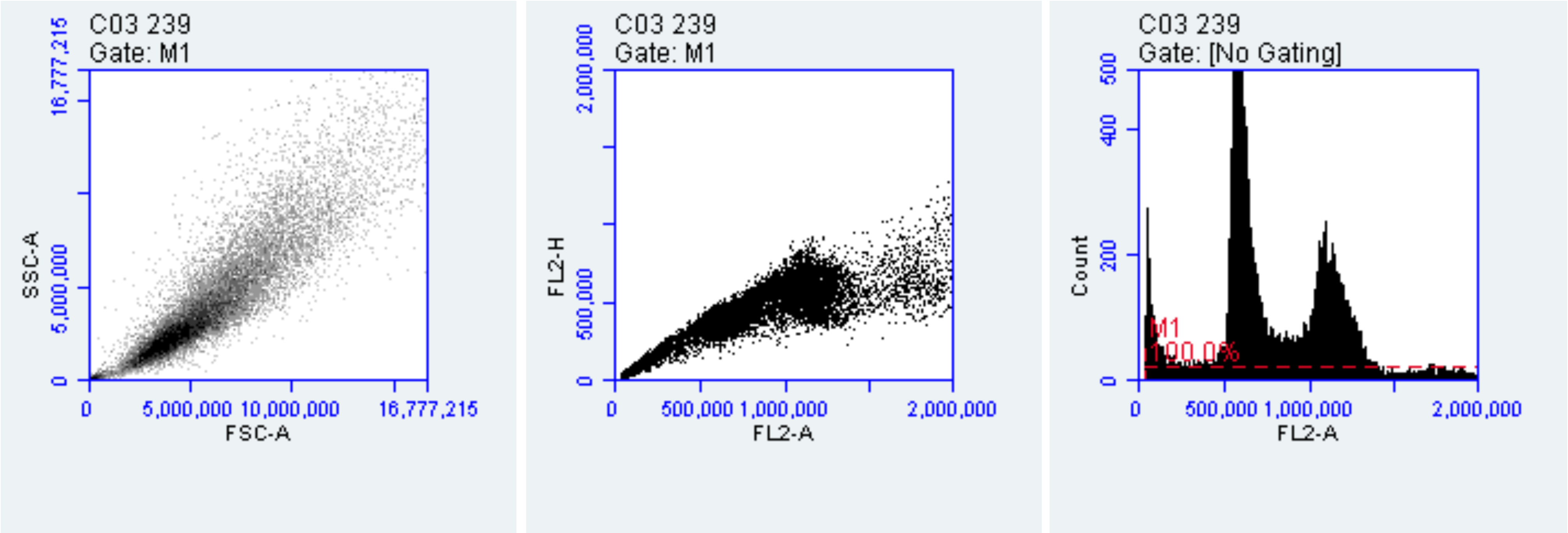

C03 239
Gate: M1
SSC-A
FSC-A
0
5,000,000
10,000,000
16,777,215
C03 239
Gate: M1
FL2-H
FL2-A
0
500,000
1,000,000
2,000,000
C03 239
Gate: [No Gating]
Count
FL2-A
0
200
400
500
500,000
1,000,000
2,000,000
M1
100.0%

MCF-7 Ambrosin Day 4

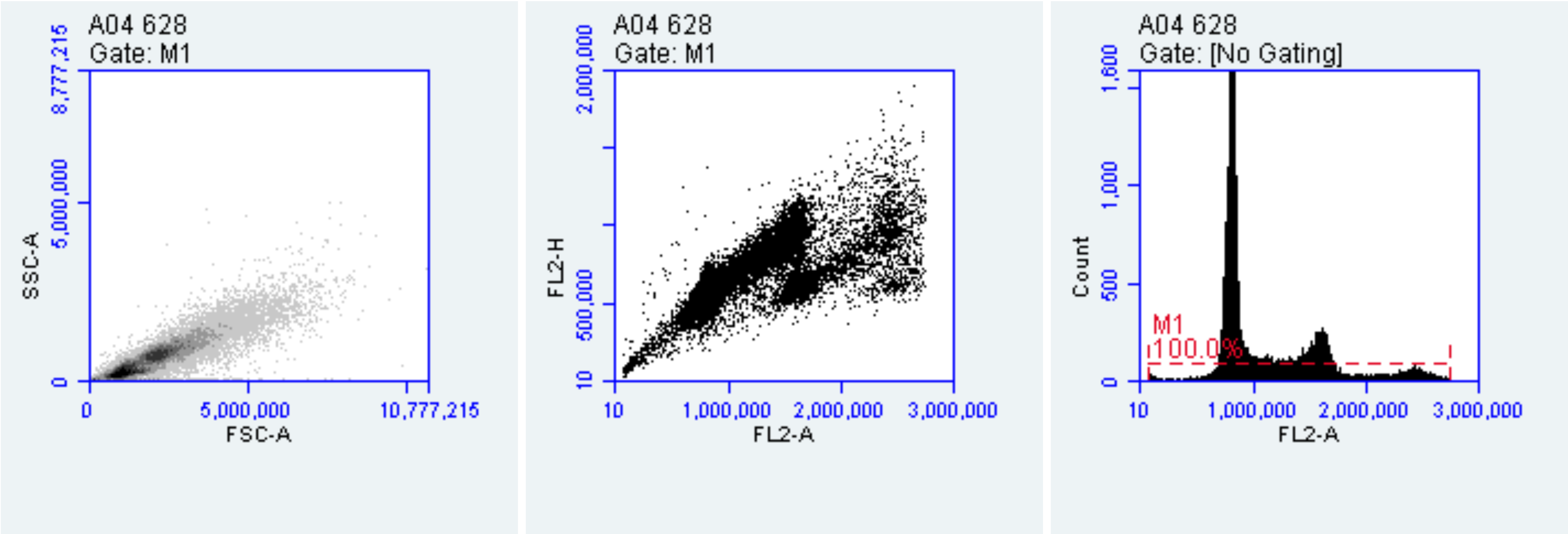

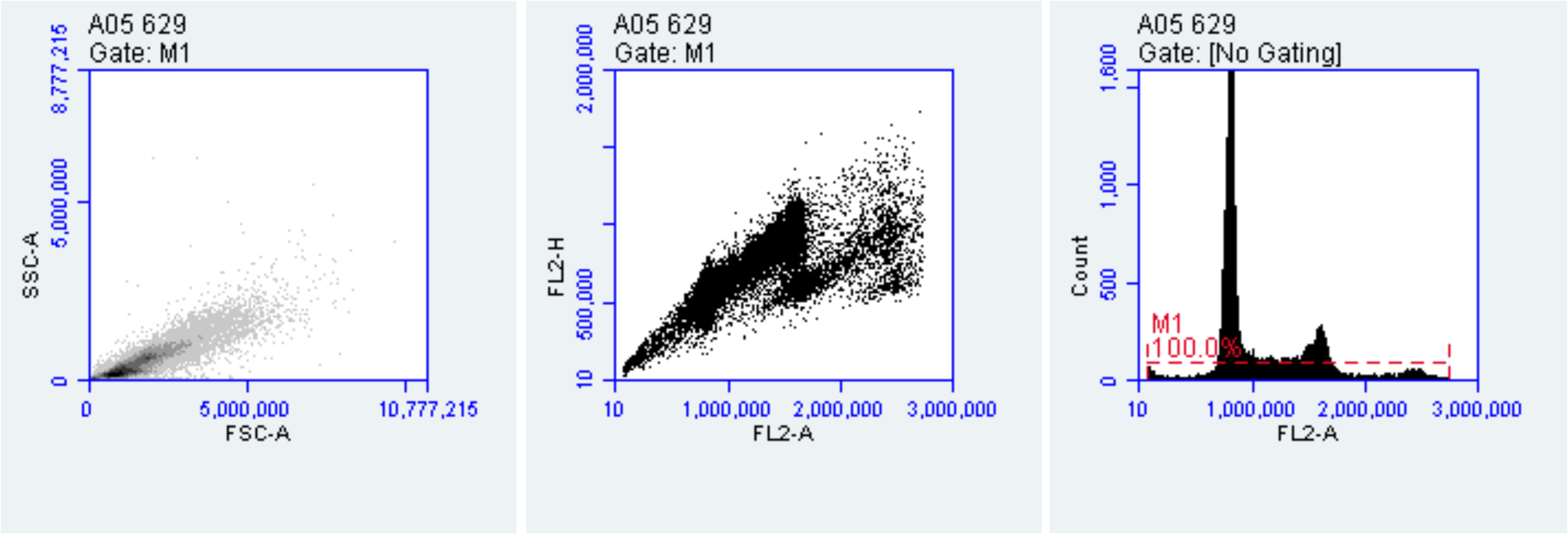
A05 629
Gate: M1
SSC-A
FSC-A
0
5,000,000
8,777,215
10,777,215
A05 629
Gate: M1
FL2-H
FL2-A
10
500,000
2,000,000
1,000,000
3,000,000
A05 629
Gate: [No Gating]
Count
0
500
1,000
1,600
M1
100.0%
10
1,000,000
2,000,000
3,000,000

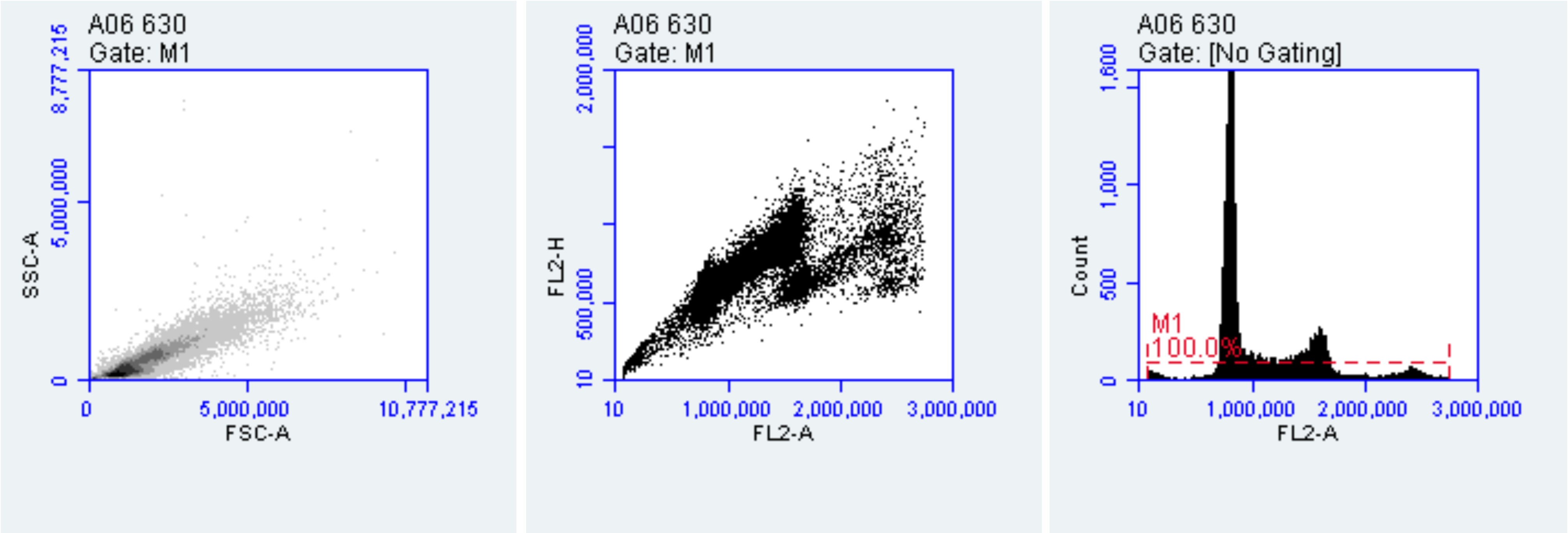
A06 630
Gate: M1
SSC-A
0
5,000,000
8,777,215
FSC-A
0
5,000,000
10,777,215
A06 630
Gate: M1
FL2-H
10
500,000
2,000,000
FL2-A
10
1,000,000
2,000,000
3,000,000
A06 630
Gate: [No Gating]
Count
0
500
1,000
1,600
M1
100.0%
FL2-A
10
1,000,000
2,000,000
3,000,000

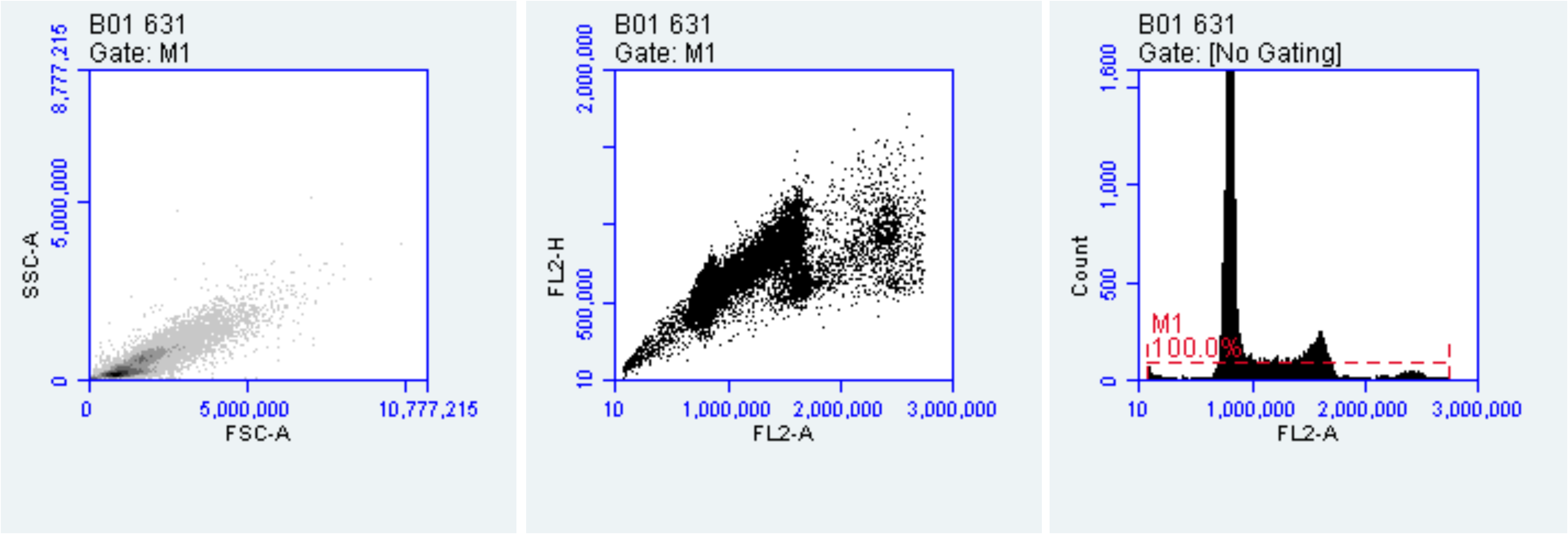

B01 631
Gate: M1
SSC-A
FSC-A
0
5,000,000
8,777,215
5,000,000
10,777,215
B01 631
Gate: M1
FL2-H
FL2-A
10
500,000
2,000,000
1,000,000
2,000,000
3,000,000
B01 631
Gate: [No Gating]
Count
0
500
1,000
1,600
M1
100.0%
10
1,000,000
2,000,000
3,000,000

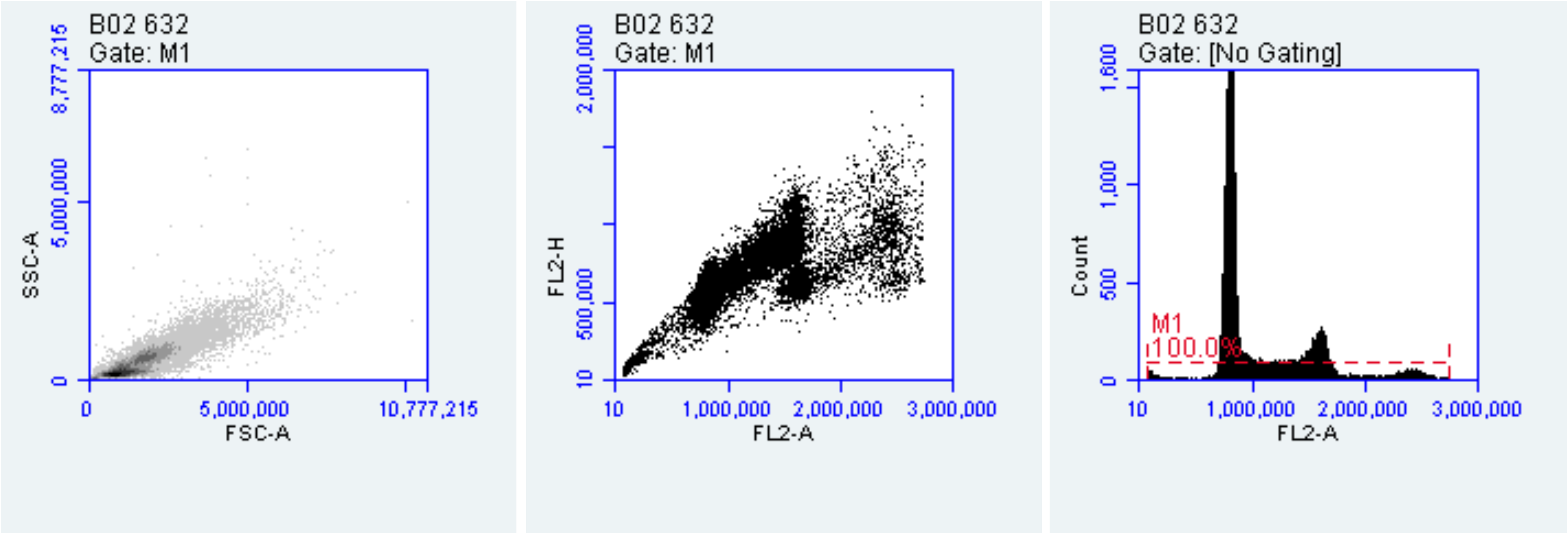
B02 632
Gate: M1
SSC-A
FSC-A
0
5,000,000
8,777,215
10,777,215
B02 632
Gate: M1
FL2-H
FL2-A
10
500,000
2,000,000
1,000,000
3,000,000
B02 632
Gate: [No Gating]
Count
M1
100.0%
0
500
1,000
1,600
10
1,000,000
2,000,000
3,000,000

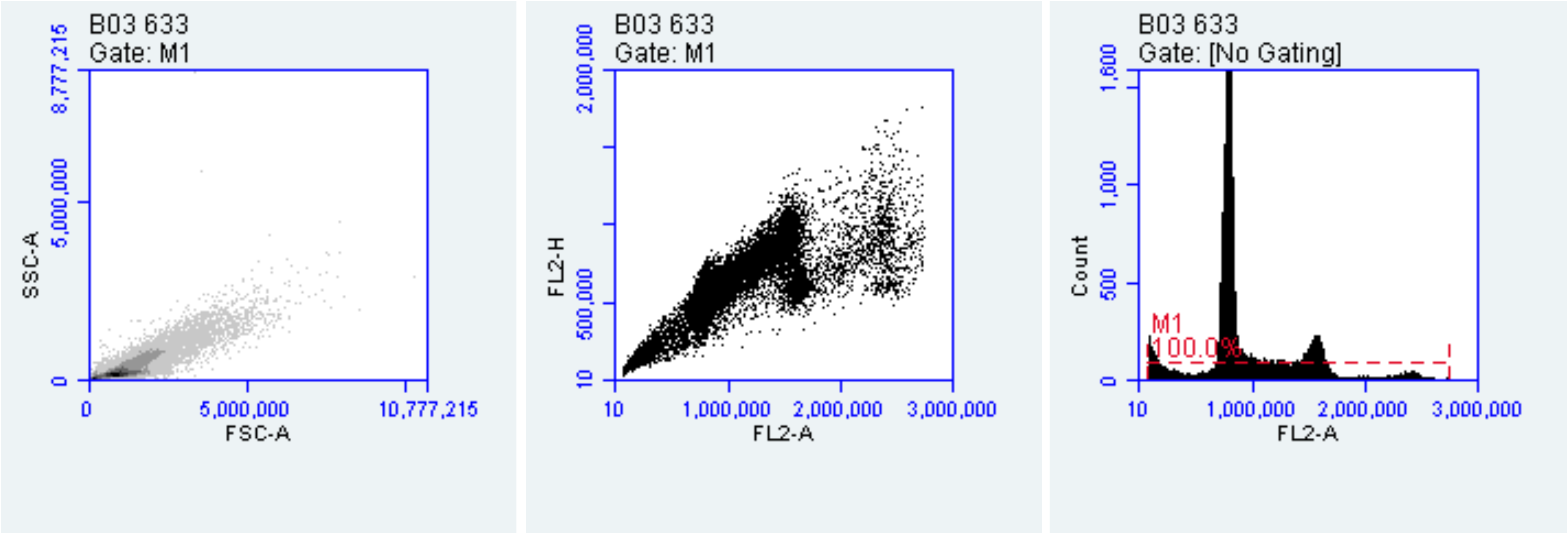
B03 633
Gate: M1
SSC-A
FSC-A
0
5,000,000
10,777,215
8,777,215
B03 633
Gate: M1
FL2-H
FL2-A
10
500,000
2,000,000
1,000,000
3,000,000
B03 633
Gate: [No Gating]
Count
FL2-A
0
500
1,000
1,600
10
1,000,000
2,000,000
3,000,000
M1
100.0%

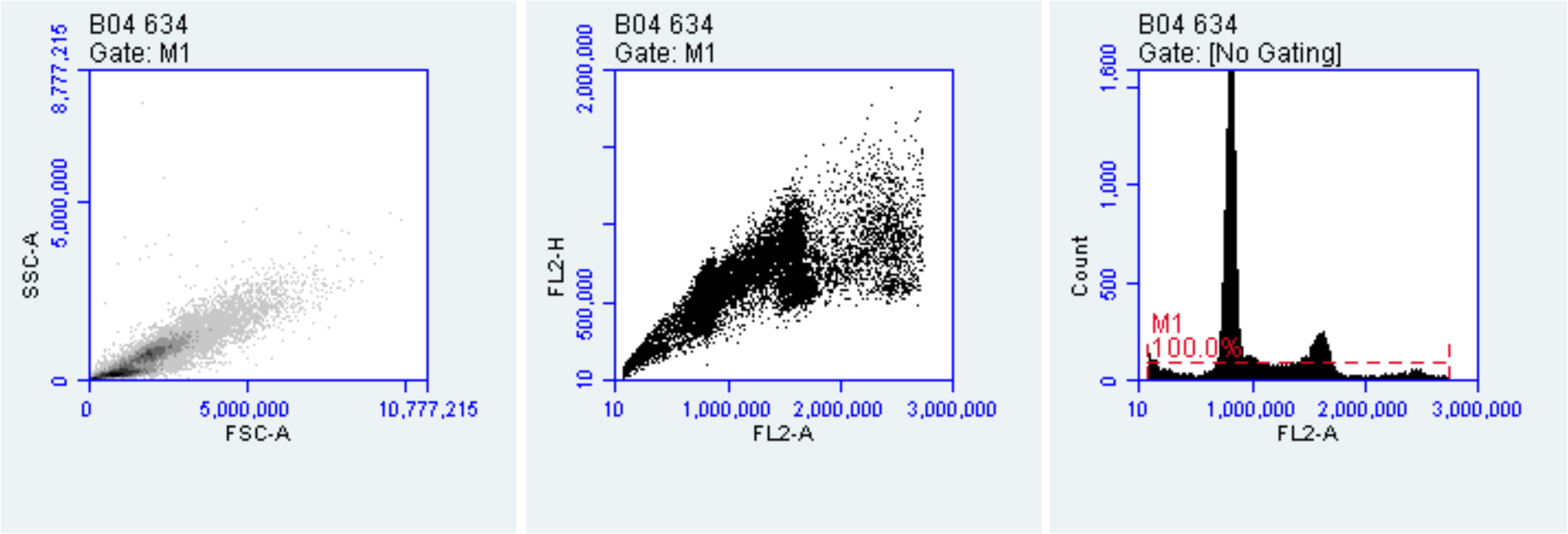
B04 634
Gate: M1
SSC-A
FSC-A
0
5,000,000
8,777,215
10,777,215
B04 634
Gate: M1
FL2-H
FL2-A
10
500,000
2,000,000
1,000,000
3,000,000
B04 634
Gate: [No Gating]
Count
0
500
1,000
1,600
M1
100.0%

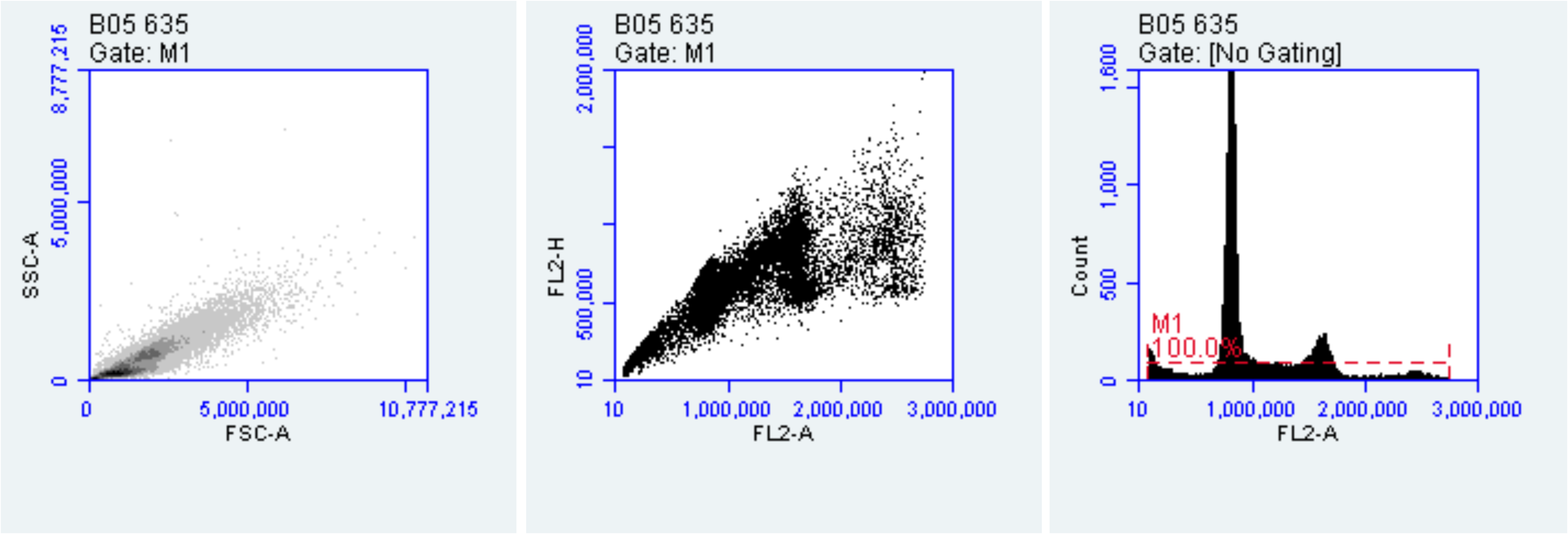
B05 635
Gate: M1
SSC-A
FSC-A
0
5,000,000
8,777,215
10,777,215
B05 635
Gate: M1
FL2-H
FL2-A
10
500,000
2,000,000
1,000,000
3,000,000
B05 635
Gate: [No Gating]
Count
FL2-A
0
500
1,000
1,600
M1
100.0%

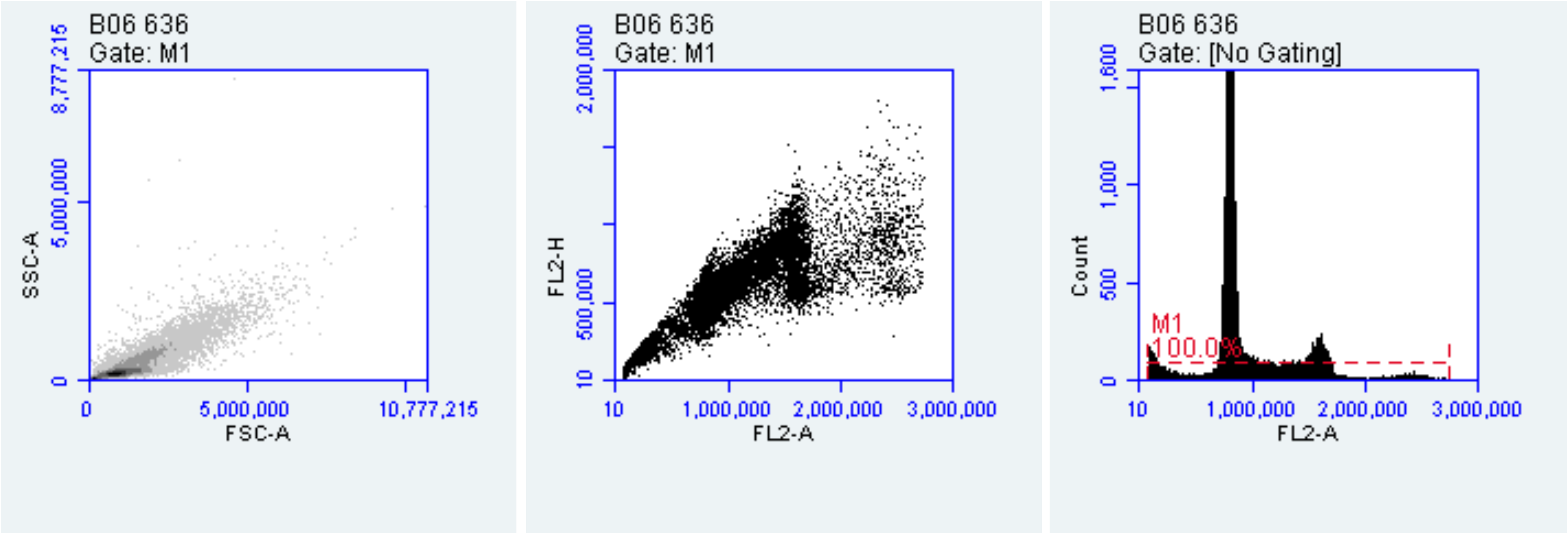

B06 636
Gate: M1
SSC-A
FSC-A
8,777,215
5,000,000
0
5,000,000
10,777,215
B06 636
Gate: M1
FL2-H
FL2-A
2,000,000
500,000
10
1,000,000
2,000,000
3,000,000
B06 636
Gate: [No Gating]
Count
FL2-A
1,600
1,000
500
0
10
1,000,000
2,000,000
3,000,000
M1
100.0%

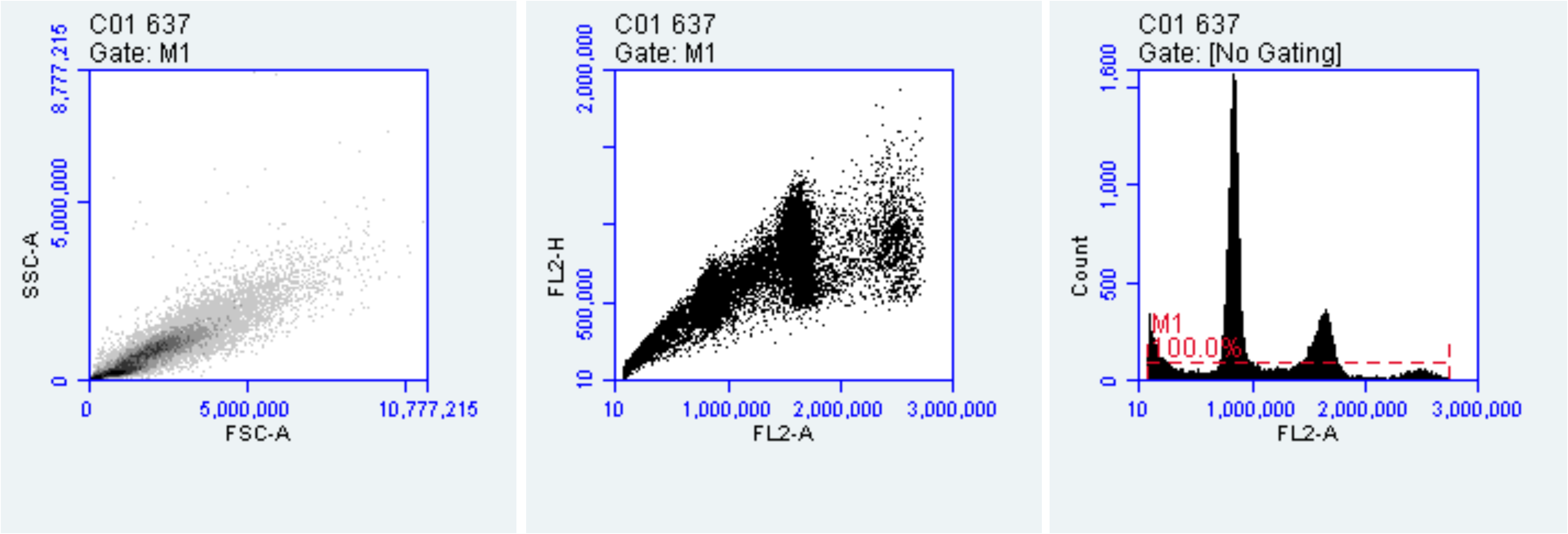
C01 637
Gate: M1
SSC-A
8,777,215
5,000,000
0
FSC-A
5,000,000
10,777,215
C01 637
Gate: M1
FL2-H
2,000,000
500,000
10
FL2-A
10
1,000,000
2,000,000
3,000,000
C01 637
Gate: [No Gating]
Count
1,600
1,000
500
0
M1
100.0%
FL2-A
10
1,000,000
2,000,000
3,000,000

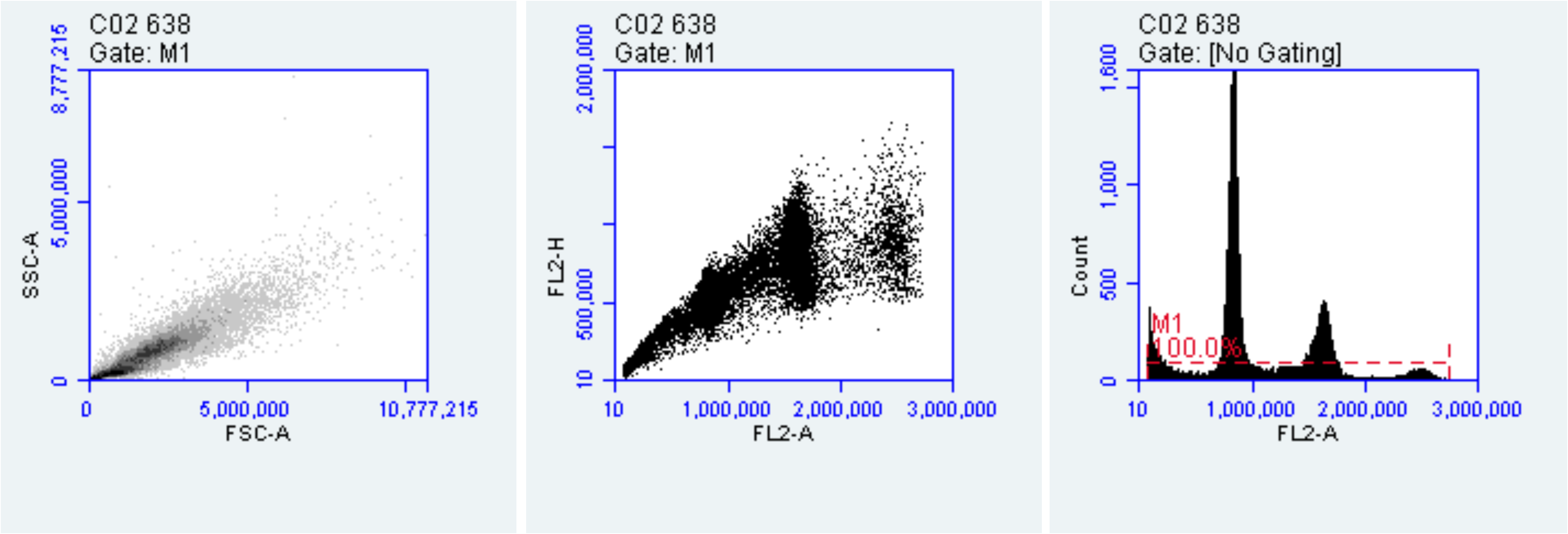
C02 638
Gate: M1
SSC-A
FSC-A
0
5,000,000
8,777,215
10,777,215
C02 638
Gate: M1
FL2-H
FL2-A
10
500,000
2,000,000
1,000,000
3,000,000
C02 638
Gate: [No Gating]
Count
FL2-A
M1
100.0%
0
500
1,000
1,600
10
1,000,000
2,000,000
3,000,000

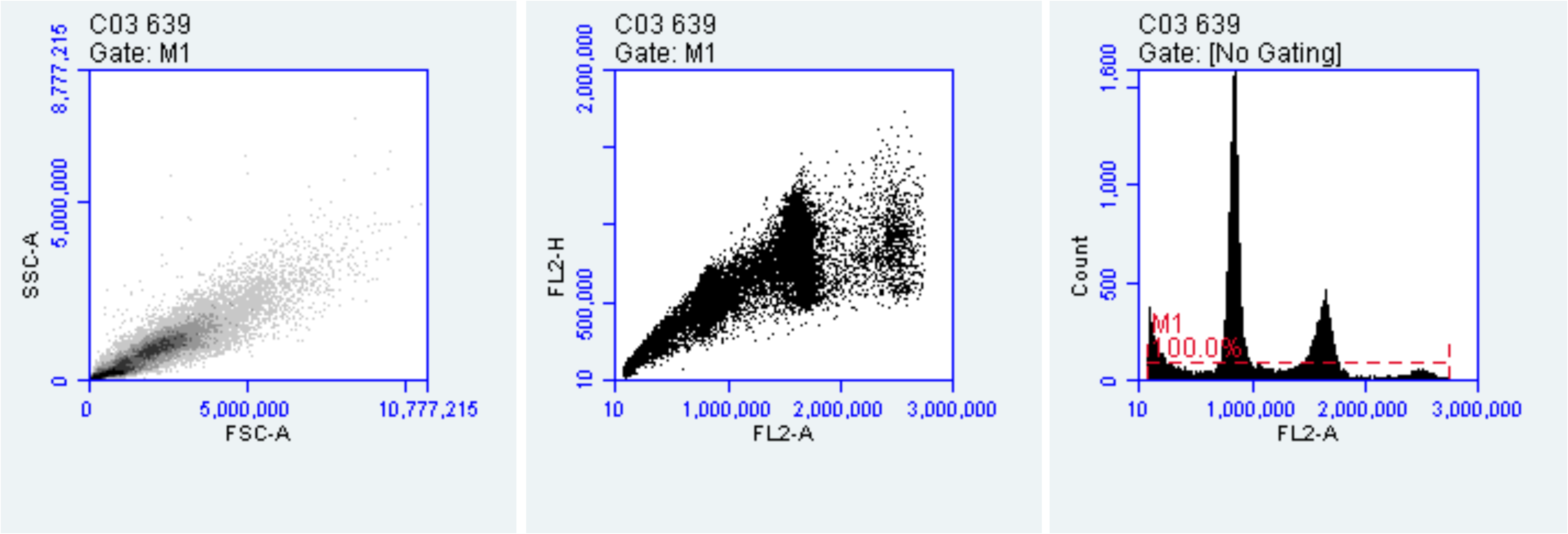
C03 639
Gate: M1
SSC-A
0
5,000,000
8,777,215
FSC-A
0
5,000,000
10,777,215
C03 639
Gate: M1
FL2-H
10
500,000
2,000,000
FL2-A
10
1,000,000
2,000,000
3,000,000
C03 639
Gate: [No Gating]
Count
0
500
1,000
1,600
M1
100.0%
FL2-A
10
1,000,000
2,000,000
3,000,000

JIMT-1 Ambrosin Day 4

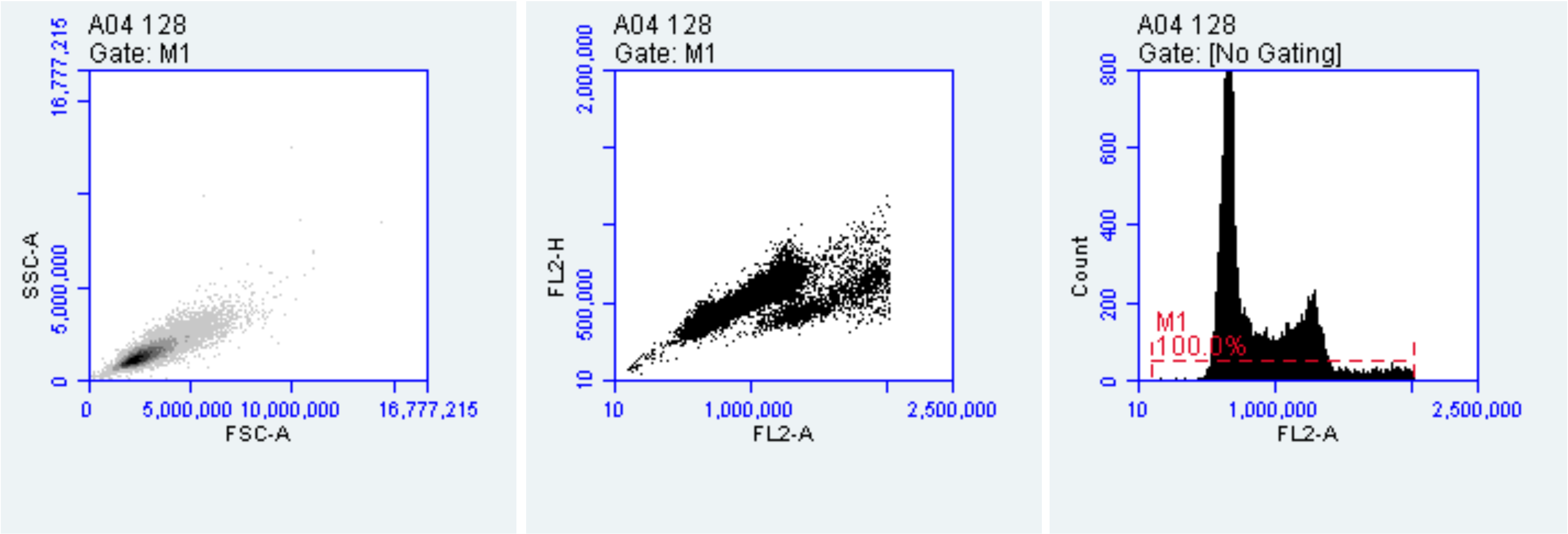

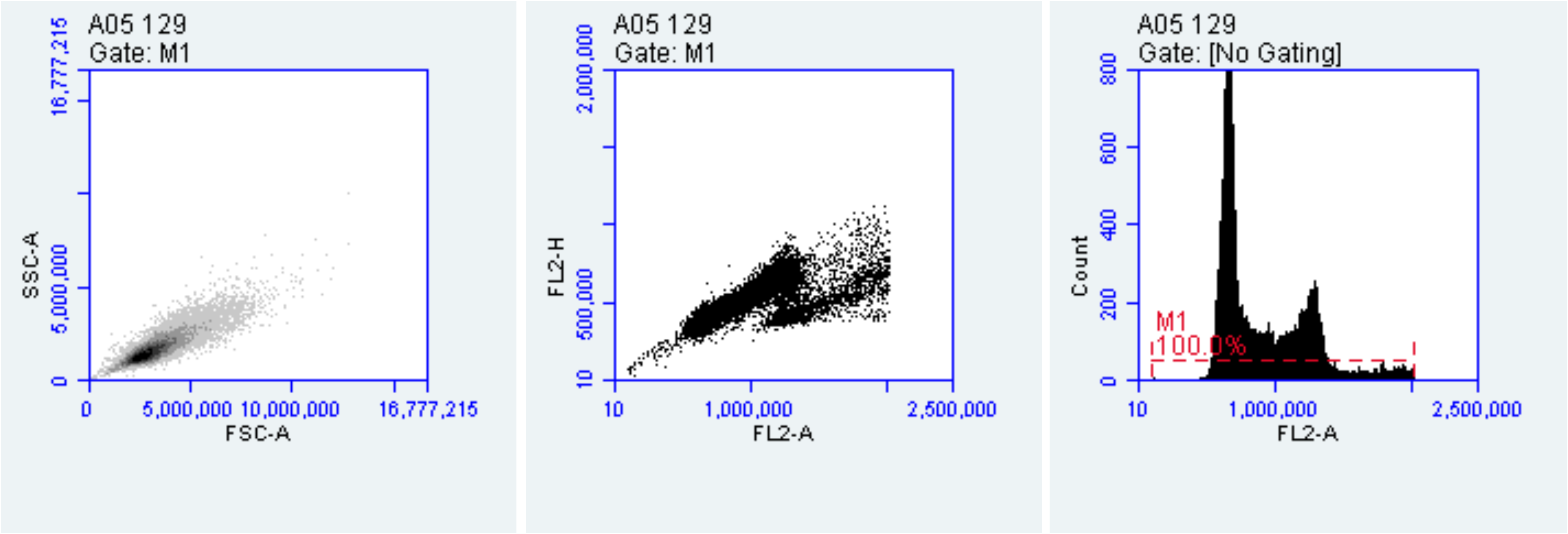
A05 129
Gate: M1
16,777,215
SSC-A
5,000,000
0
0
5,000,000
10,000,000
16,777,215
FSC-A
A05 129
Gate: M1
2,000,000
FL2-H
500,000
10
10
1,000,000
2,500,000
FL2-A
A05 129
Gate: [No Gating]
800
600
400
200
0
Count
M1
100.0%
10
1,000,000
2,500,000
FL2-A

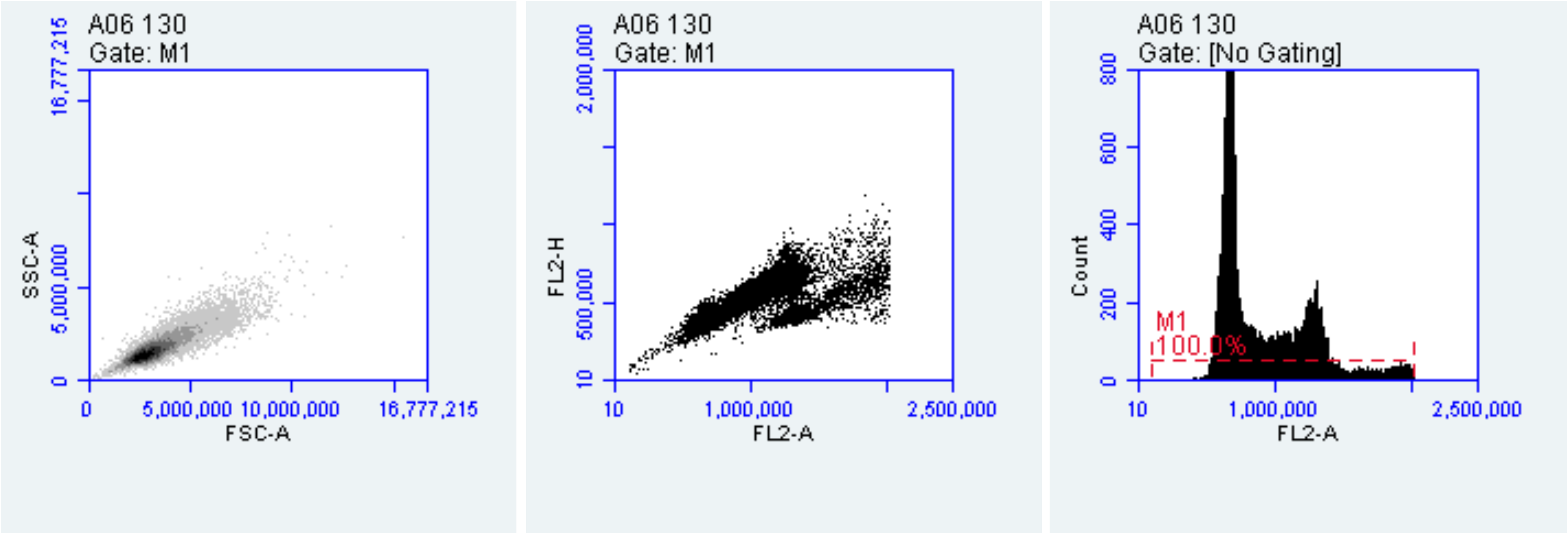
A06 130
Gate: M1
SSC-A
FSC-A
0
5,000,000
10,000,000
16,777,215
A06 130
Gate: M1
FL2-H
FL2-A
10
500,000
2,000,000
1,000,000
2,500,000
A06 130
Gate: [No Gating]
Count
0
200
400
600
800
M1
100.0%
FL2-A
10
1,000,000
2,500,000

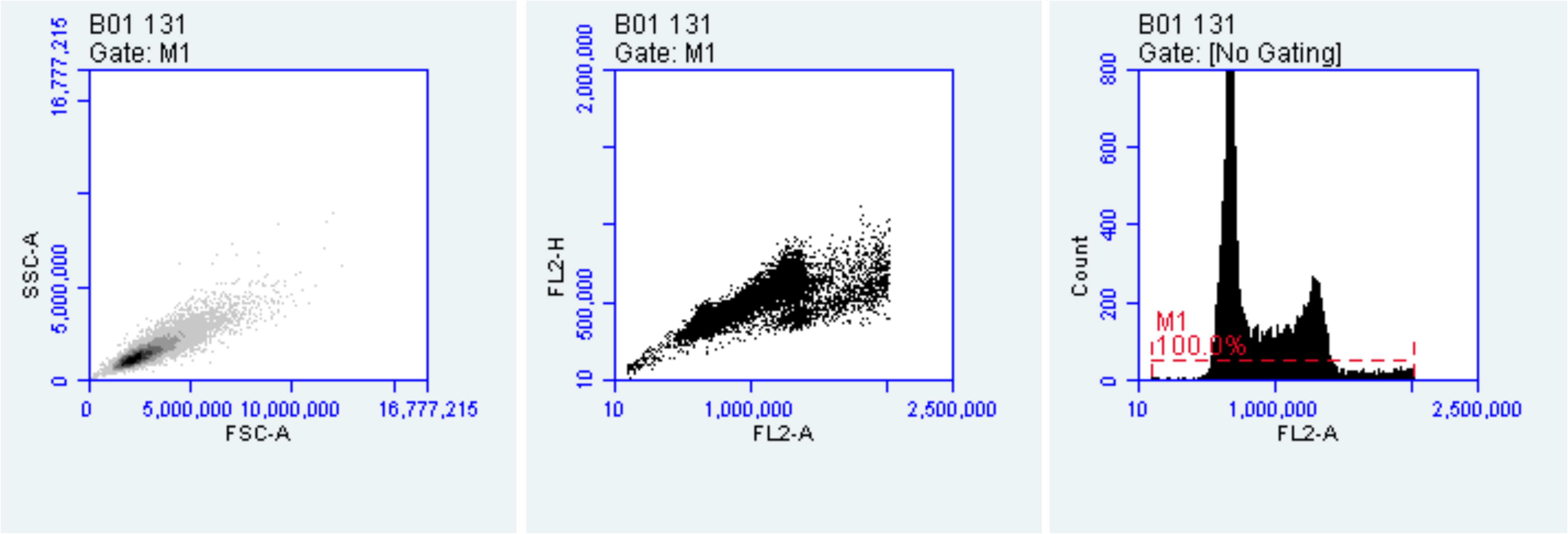
B01 131
Gate: M1
SSC-A
FSC-A
0
5,000,000
16,777,215
10,000,000
B01 131
Gate: M1
FL2-H
FL2-A
10
500,000
2,000,000
1,000,000
2,500,000
B01 131
Gate: [No Gating]
Count
0
200
400
600
800
M1
100.0%

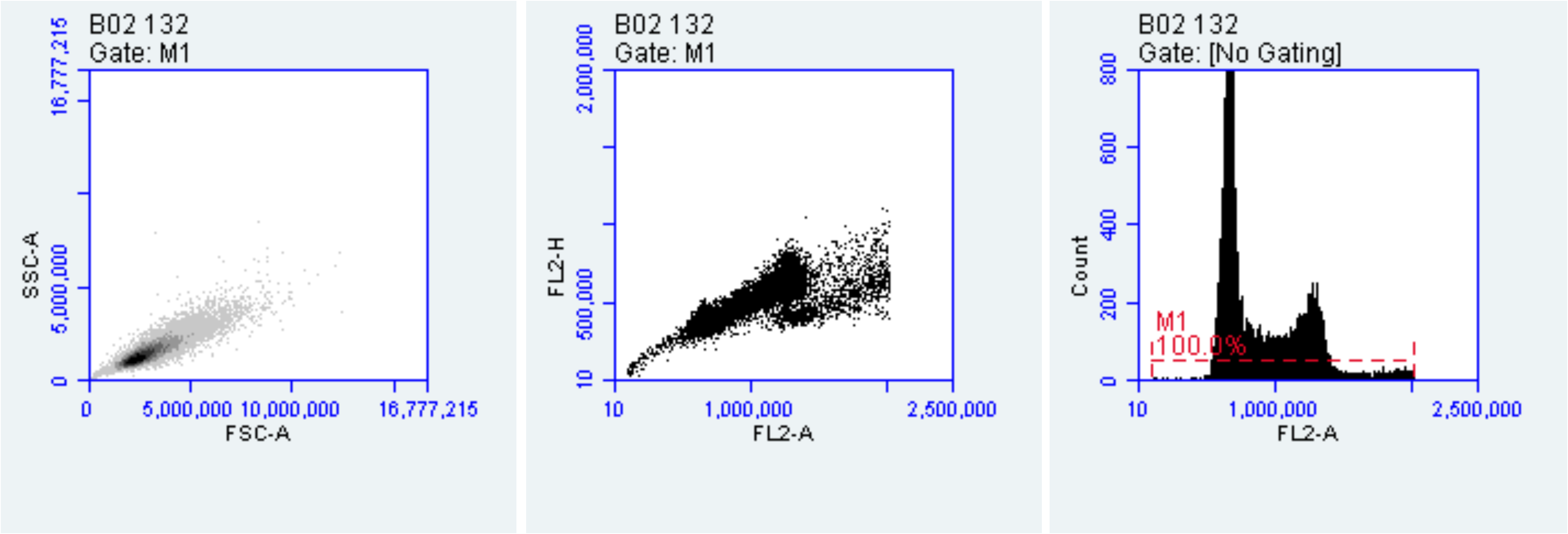
B02 132
Gate: M1
16,777,215
SSC-A
5,000,000
0
0
5,000,000
10,000,000
16,777,215
FSC-A
B02 132
Gate: M1
2,000,000
FL2-H
500,000
10
10
1,000,000
2,500,000
FL2-A
B02 132
Gate: [No Gating]
800
600
400
200
0
Count
M1
100.0%
10
1,000,000
2,500,000
FL2-A

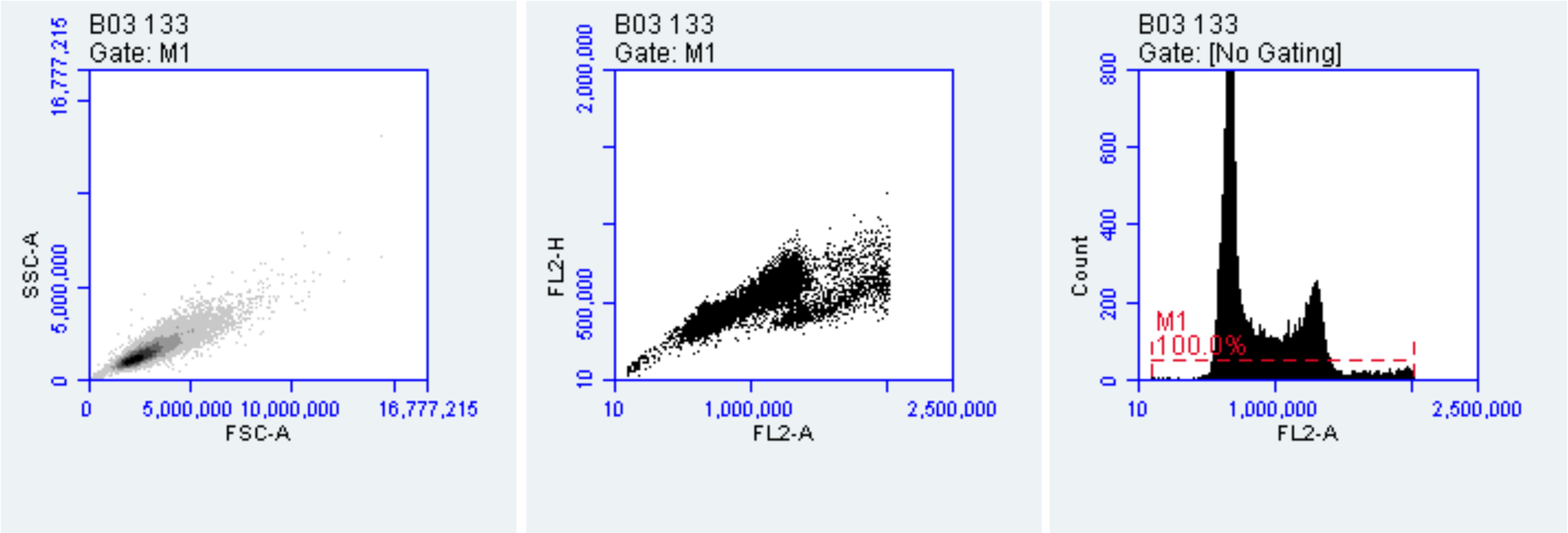
B03 133
Gate: M1
SSC-A
FSC-A
16,777,215
5,000,000
0
5,000,000 10,000,000
B03 133
Gate: M1
FL2-H
FL2-A
2,000,000
500,000
10
1,000,000
2,500,000
B03 133
Gate: [No Gating]
Count
FL2-A
800
600
400
200
0
10
1,000,000
2,500,000
M1
100.0%

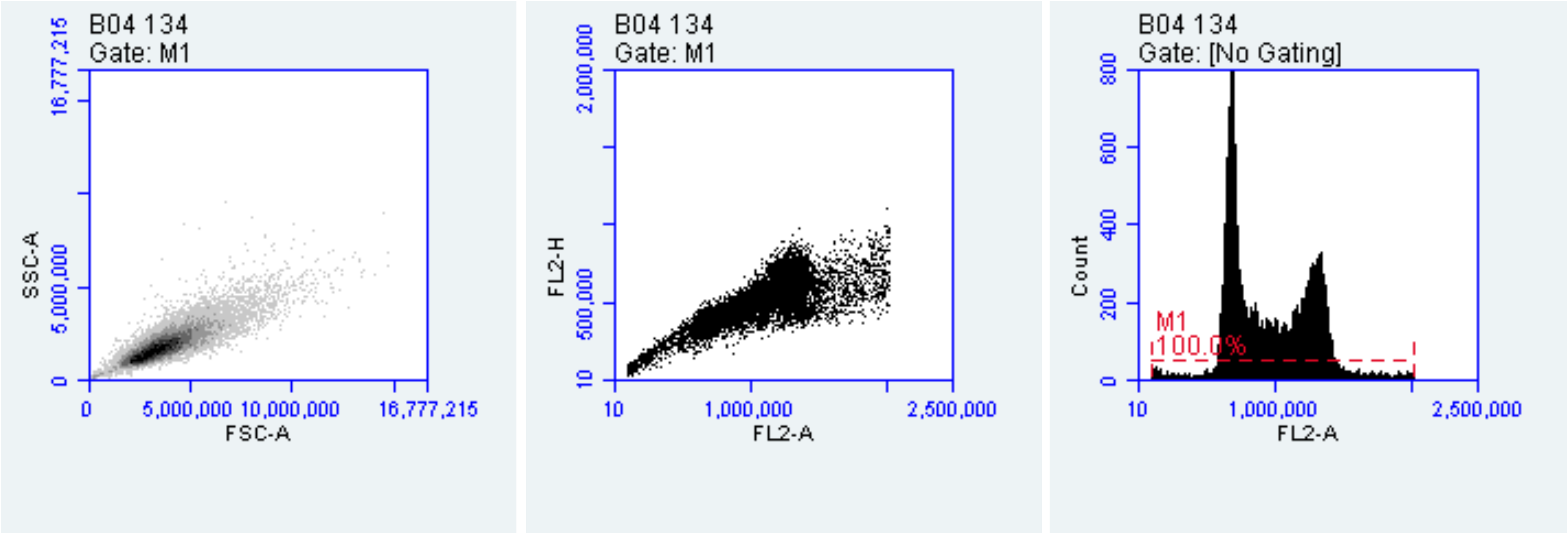
B04 134
Gate: M1
SSC-A
FSC-A
0
5,000,000
10,000,000
16,777,215
B04 134
Gate: M1
FL2-H
FL2-A
10
500,000
2,000,000
1,000,000
2,500,000
B04 134
Gate: [No Gating]
Count
FL2-A
0
200
400
600
800
M1
100.0%

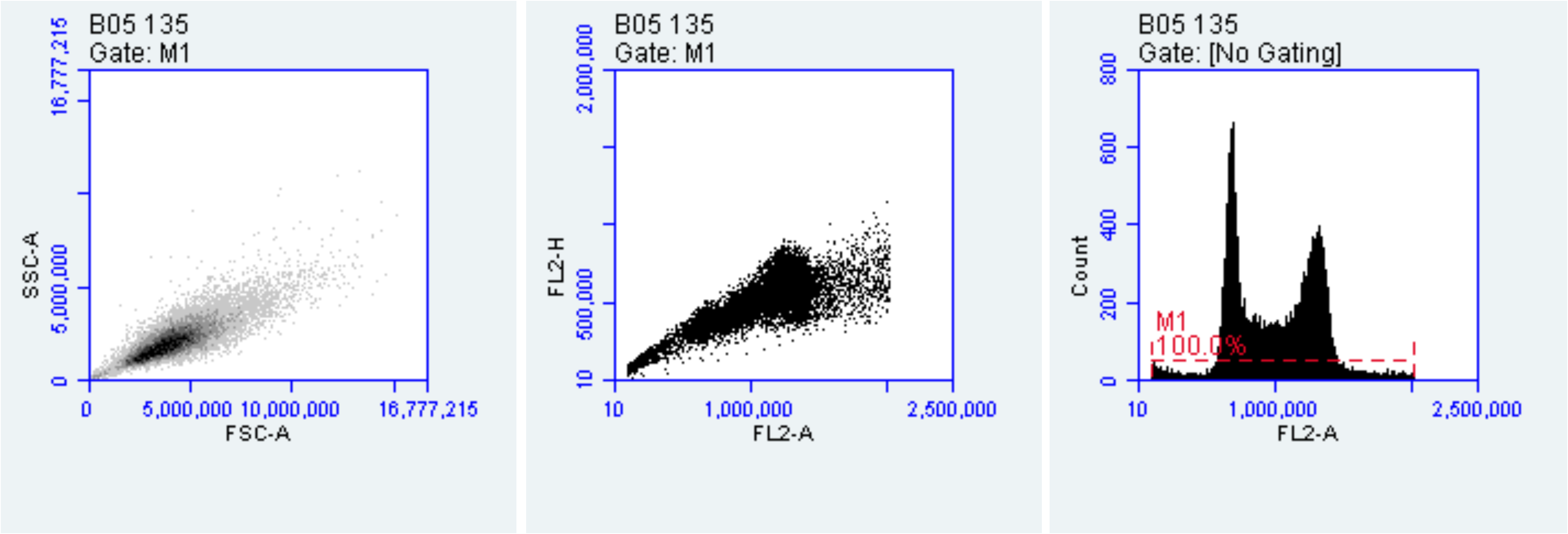

B05 135
Gate: M1
SSC-A
FSC-A
0
5,000,000
10,000,000
16,777,215
B05 135
Gate: M1
FL2-H
FL2-A
10
500,000
2,000,000
1,000,000
2,500,000
B05 135
Gate: [No Gating]
Count
0
200
400
600
800
M1
100.0%
FL2-A
10
1,000,000
2,500,000

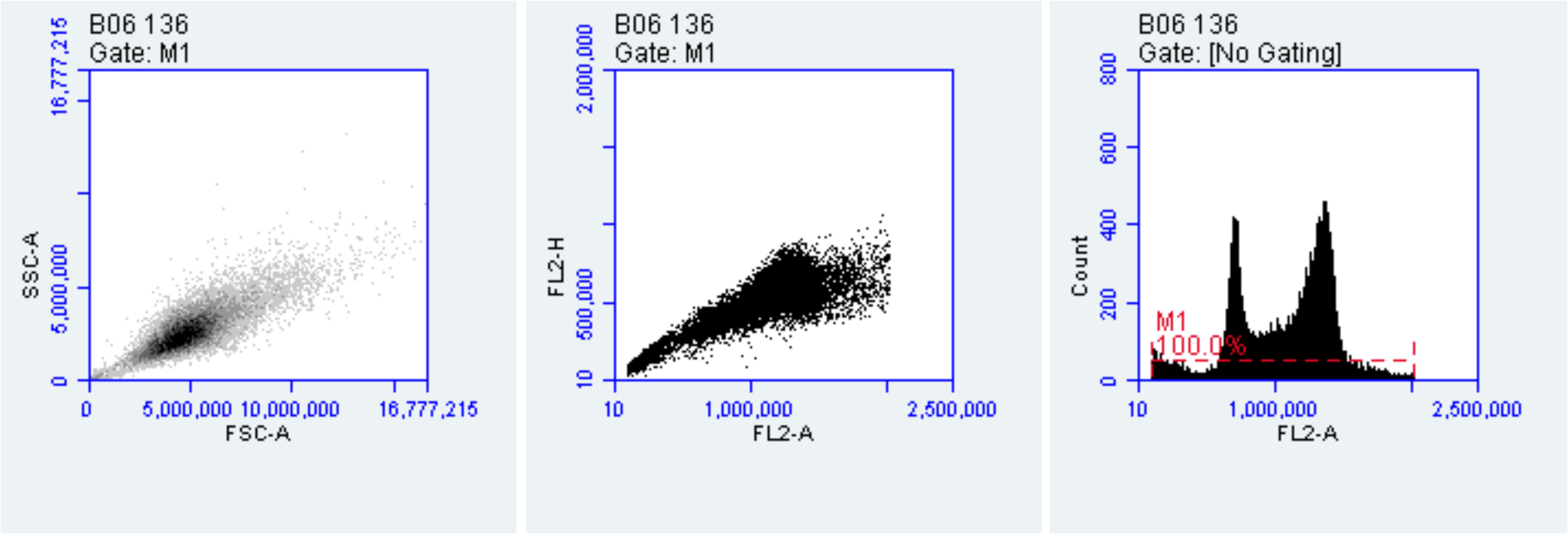
B06 136
Gate: M1
SSC-A
FSC-A
0
5,000,000
10,000,000
16,777,215
B06 136
Gate: M1
FL2-H
FL2-A
10
500,000
2,000,000
1,000,000
2,500,000
B06 136
Gate: [No Gating]
Count
0
200
400
600
800
10
1,000,000
2,500,000
FL2-A
M1
100.0%

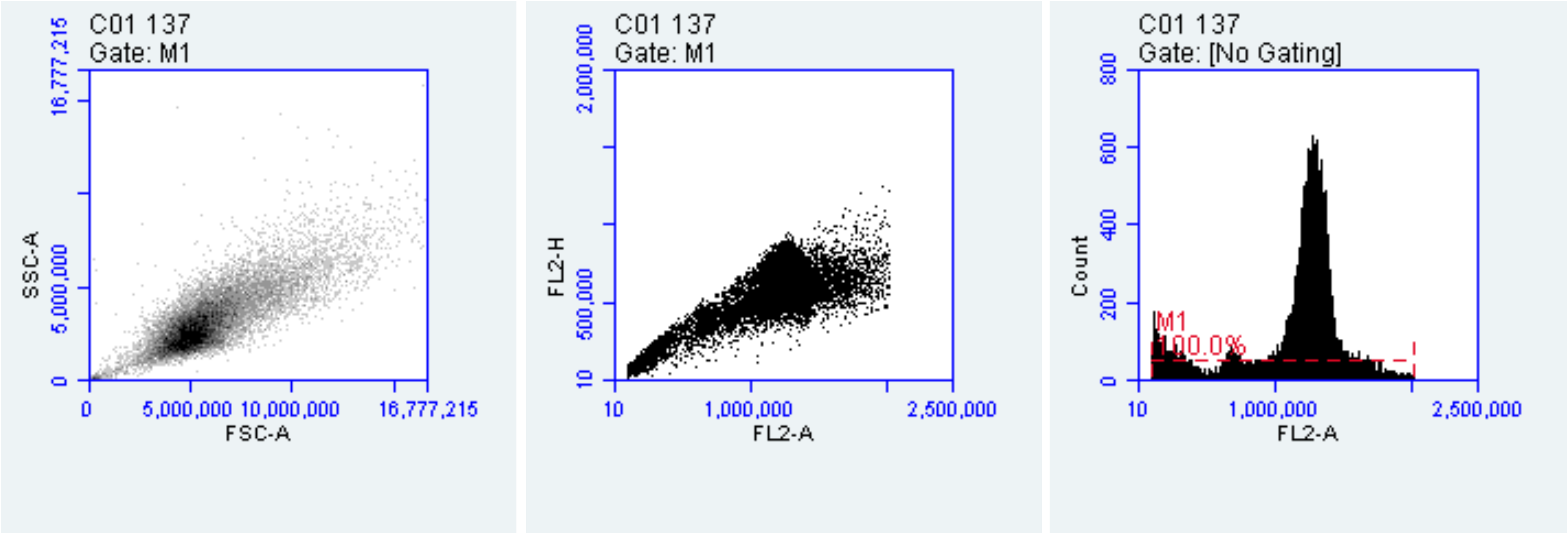
C01 137
Gate: M1
SSC-A
16,777,215
5,000,000
0
FSC-A
0
5,000,000
10,000,000
16,777,215
C01 137
Gate: M1
FL2-H
2,000,000
500,000
10
FL2-A
10
1,000,000
2,500,000
C01 137
Gate: [No Gating]
Count
800
600
400
200
0
M1
100.0%
FL2-A
10
1,000,000
2,500,000

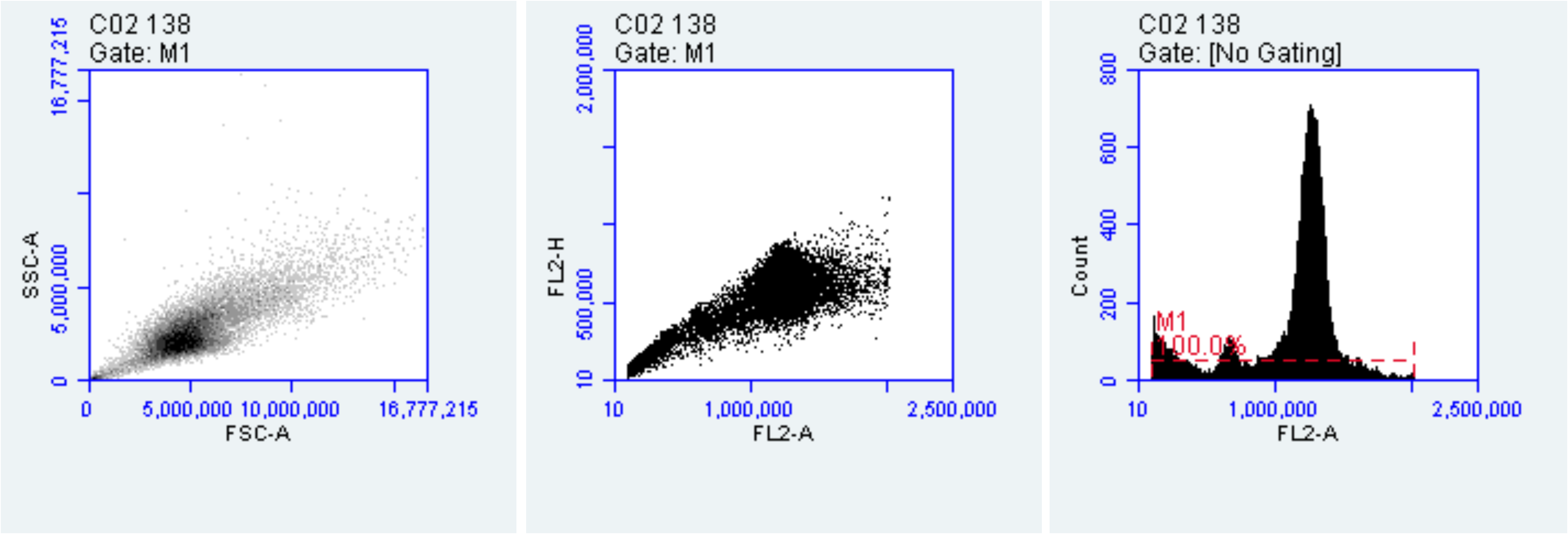
C02 138
Gate: M1
SSC-A
FSC-A
0
5,000,000
16,777,215
0
5,000,000
10,000,000
16,777,215
C02 138
Gate: M1
FL2-H
FL2-A
10
500,000
2,000,000
10
1,000,000
2,500,000
C02 138
Gate: [No Gating]
Count
FL2-A
0
200
400
600
800
10
1,000,000
2,500,000
M1
100.0%

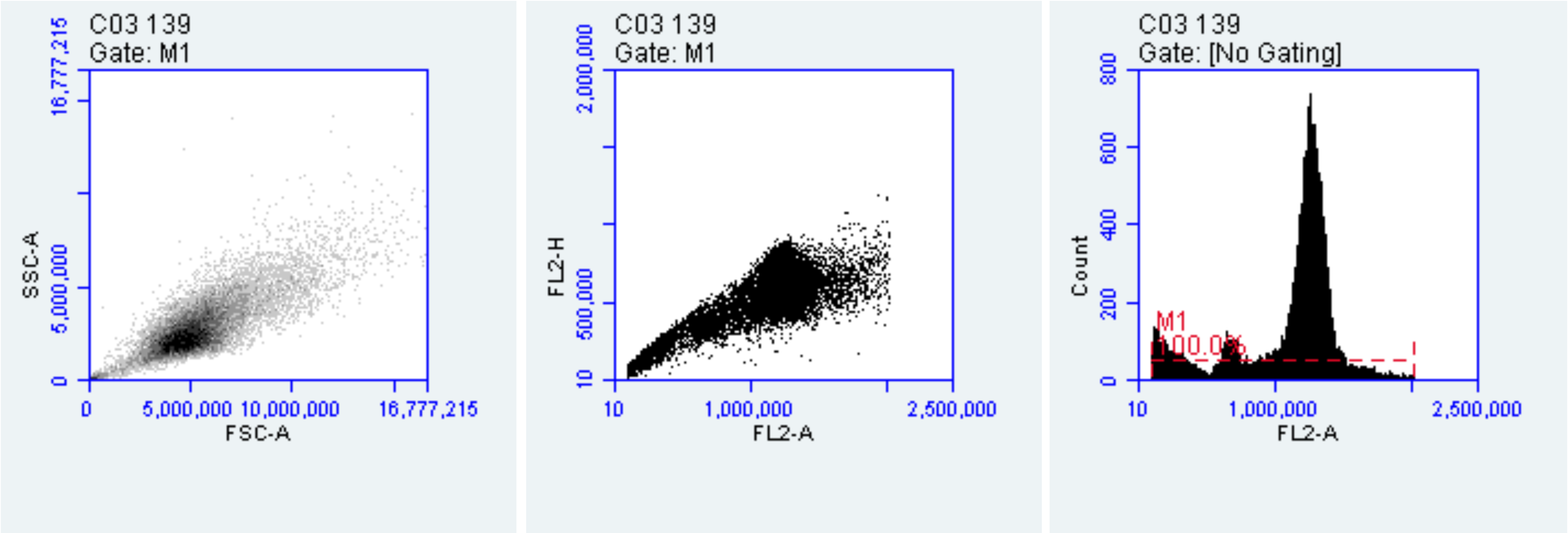
C03 139
Gate: M1
16,777,215
5,000,000
0
SSC-A
0
5,000,000
10,000,000
16,777,215
FSC-A
C03 139
Gate: M1
2,000,000
500,000
10
FL2-H
10
1,000,000
2,500,000
FL2-A
C03 139
Gate: [No Gating]
800
600
400
200
0
Count
M1
100.0%
10
1,000,000
2,500,000
FL2-A

HCC1937 Ambrosin Day 4

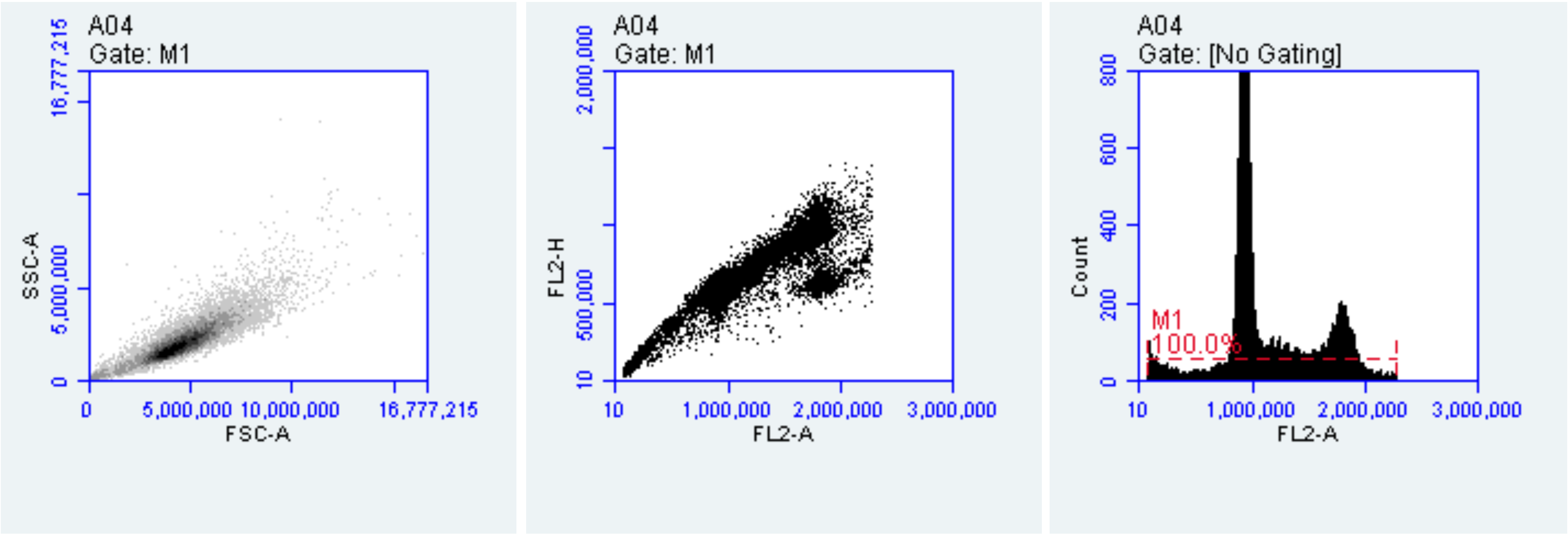

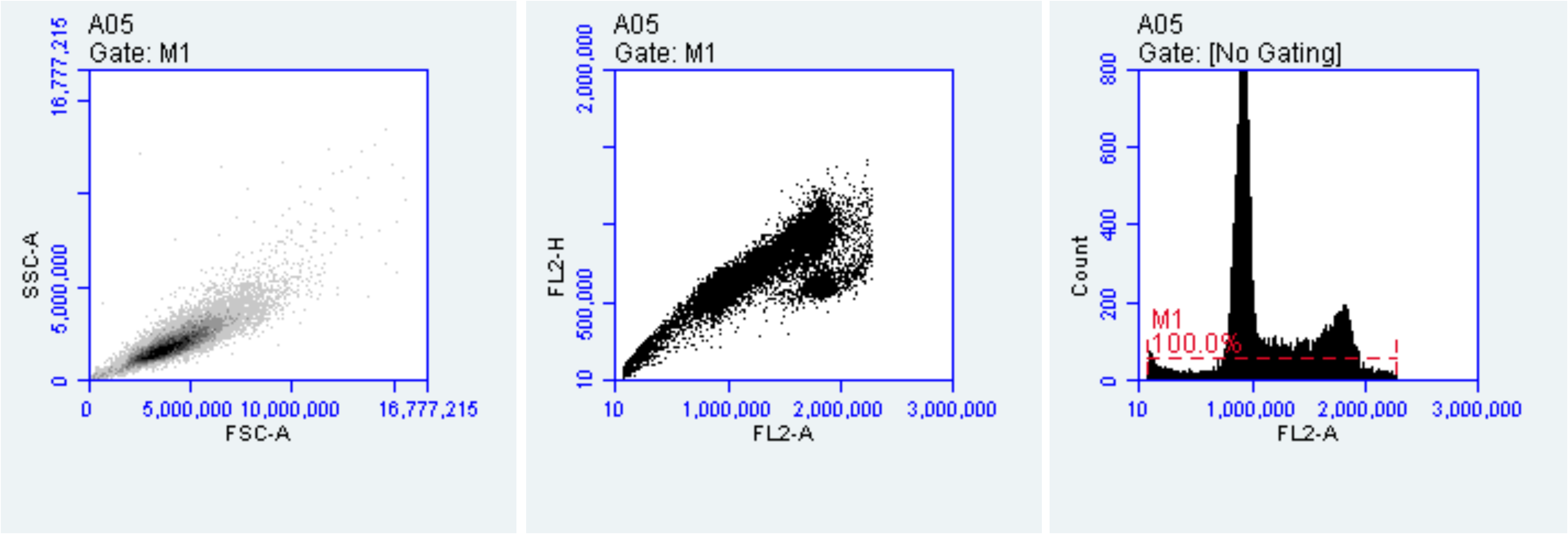

A05
Gate: M1
SSC-A
FSC-A
0
5,000,000
10,000,000
16,777,215
A05
Gate: M1
FL2-H
FL2-A
10
500,000
2,000,000
1,000,000
3,000,000
A05
Gate: [No Gating]
Count
0
200
400
600
800
M1
100.0%
FL2-A
10
1,000,000
2,000,000
3,000,000

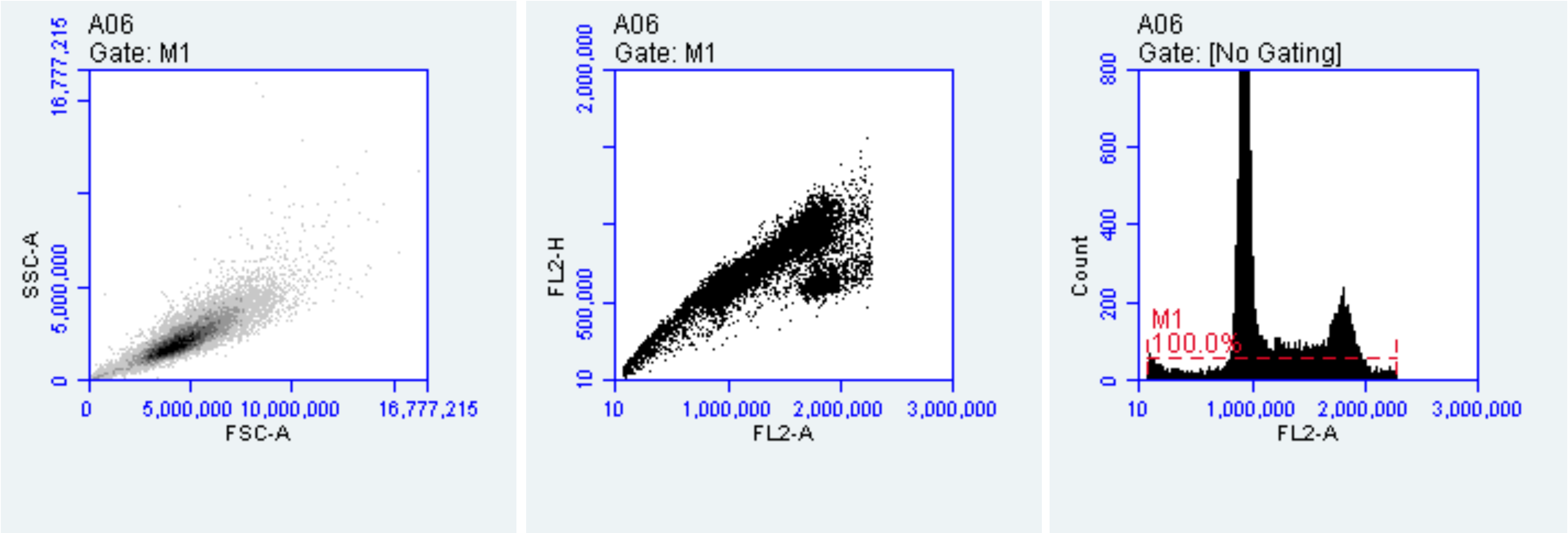
A06
Gate: M1
16,777,215
5,000,000
0
SSC-A
FSC-A
10,000,000
A06
Gate: M1
2,000,000
500,000
10
FL2-H
1,000,000
2,000,000
3,000,000
FL2-A
A06
Gate: [No Gating]
800
600
400
200
Count
M1
100.0%

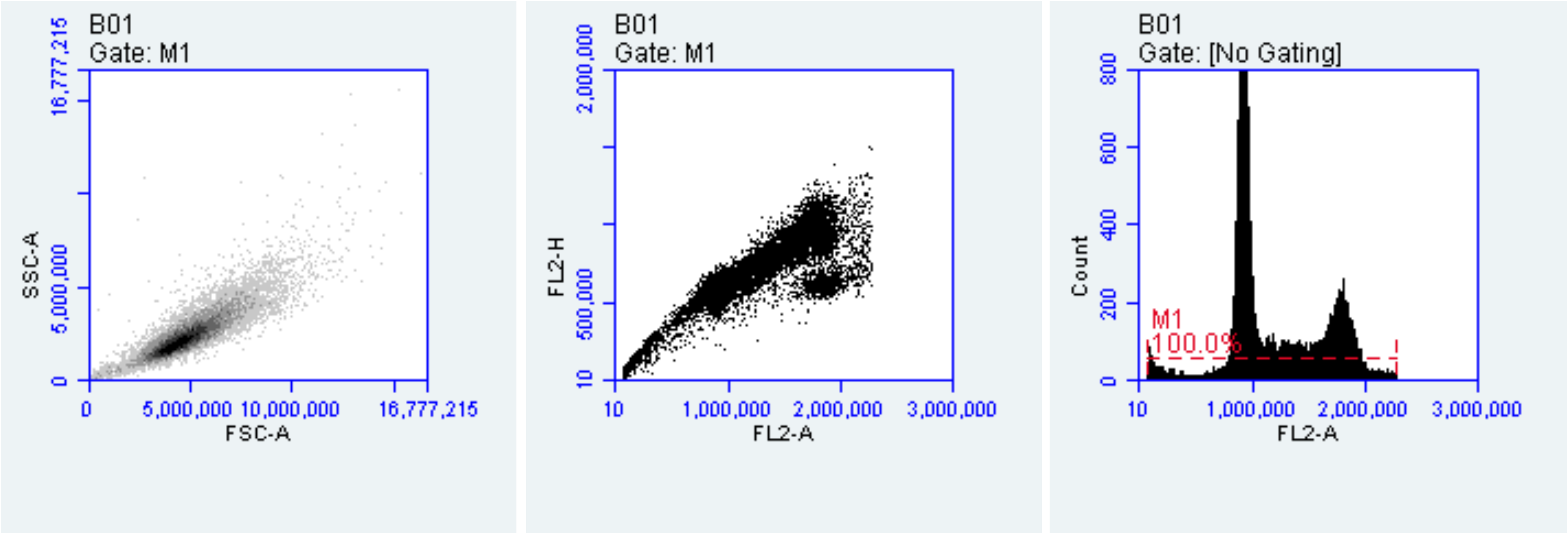
B01
Gate: M1
16,777,215
SSC-A
5,000,000
0
0
5,000,000 10,000,000
16,777,215
FSC-A
B01
Gate: M1
2,000,000
FL2-H
500,000
10
10
1,000,000
2,000,000
3,000,000
FL2-A
B01
Gate: [No Gating]
800
600
400
Count
200
0
M1
100.0%
10
1,000,000
2,000,000
3,000,000
FL2-A

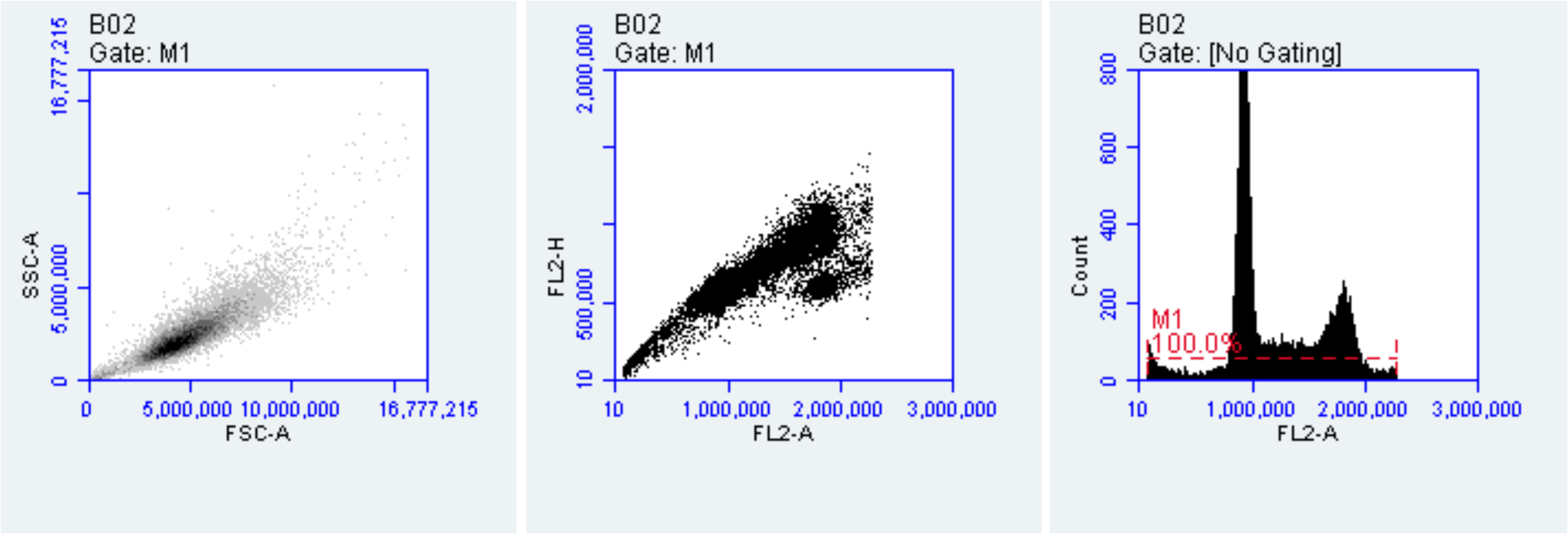
B02
Gate: M1
SSC-A
FSC-A
0
5,000,000
10,000,000
16,777,215
B02
Gate: M1
FL2-H
FL2-A
10
500,000
2,000,000
1,000,000
3,000,000
B02
Gate: [No Gating]
Count
0
200
400
600
800
M1
100.0%

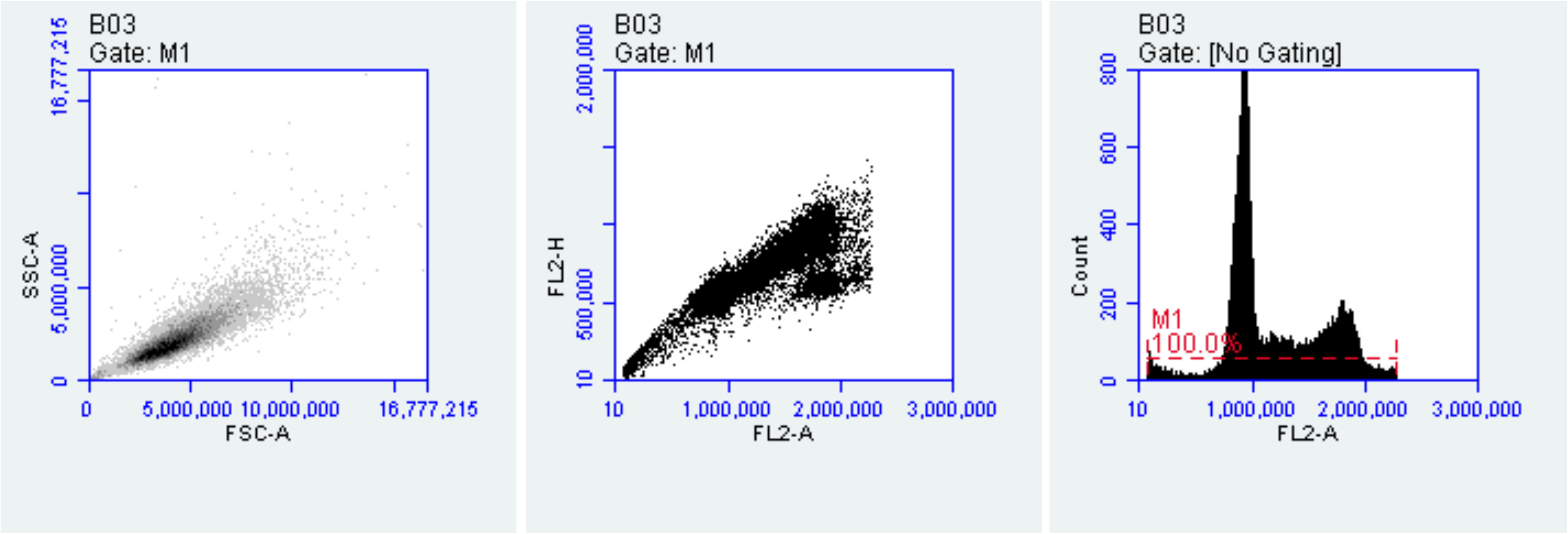
B03
Gate: M1
SSC-A
FSC-A
0
5,000,000
10,000,000
16,777,215
B03
Gate: M1
FL2-H
FL2-A
10
500,000
2,000,000
1,000,000
3,000,000
B03
Gate: [No Gating]
Count
0
200
400
600
800
M1
100.0%
FL2-A
10
1,000,000
2,000,000
3,000,000

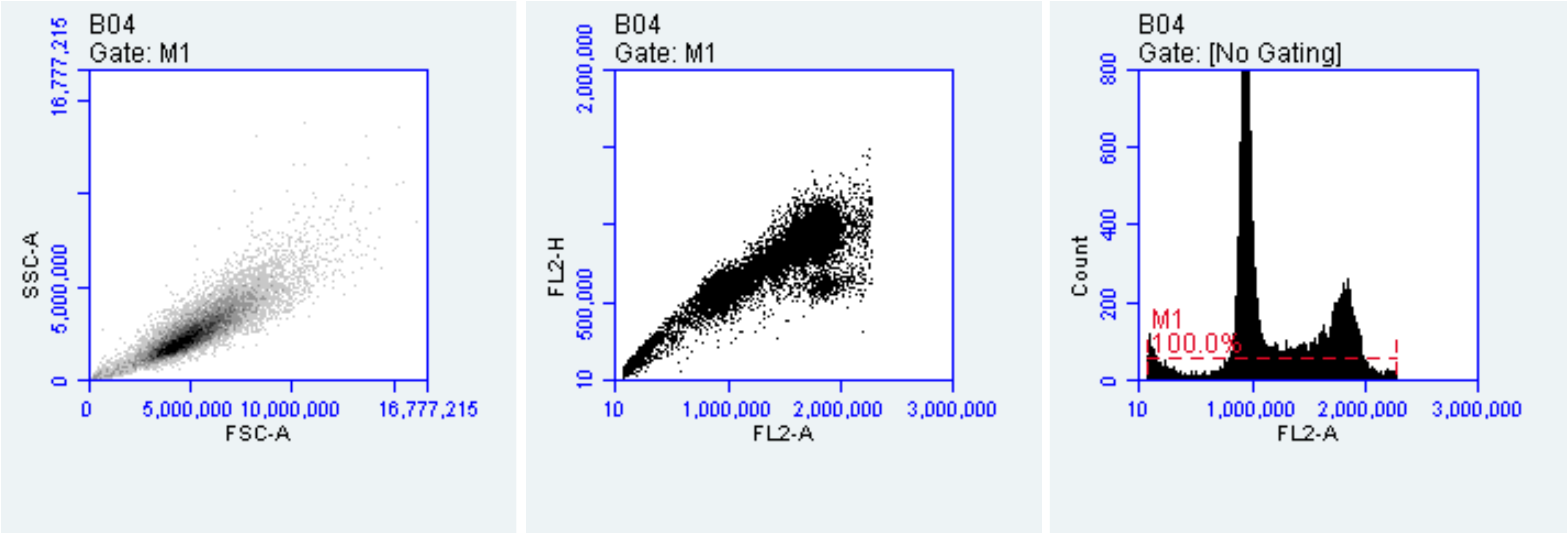
B04
Gate: M1
SSC-A
FSC-A
0
5,000,000
10,000,000
16,777,215
B04
Gate: M1
FL2-H
FL2-A
10
500,000
2,000,000
1,000,000
3,000,000
B04
Gate: [No Gating]
Count
FL2-A
0
200
400
600
800
M1
100.0%

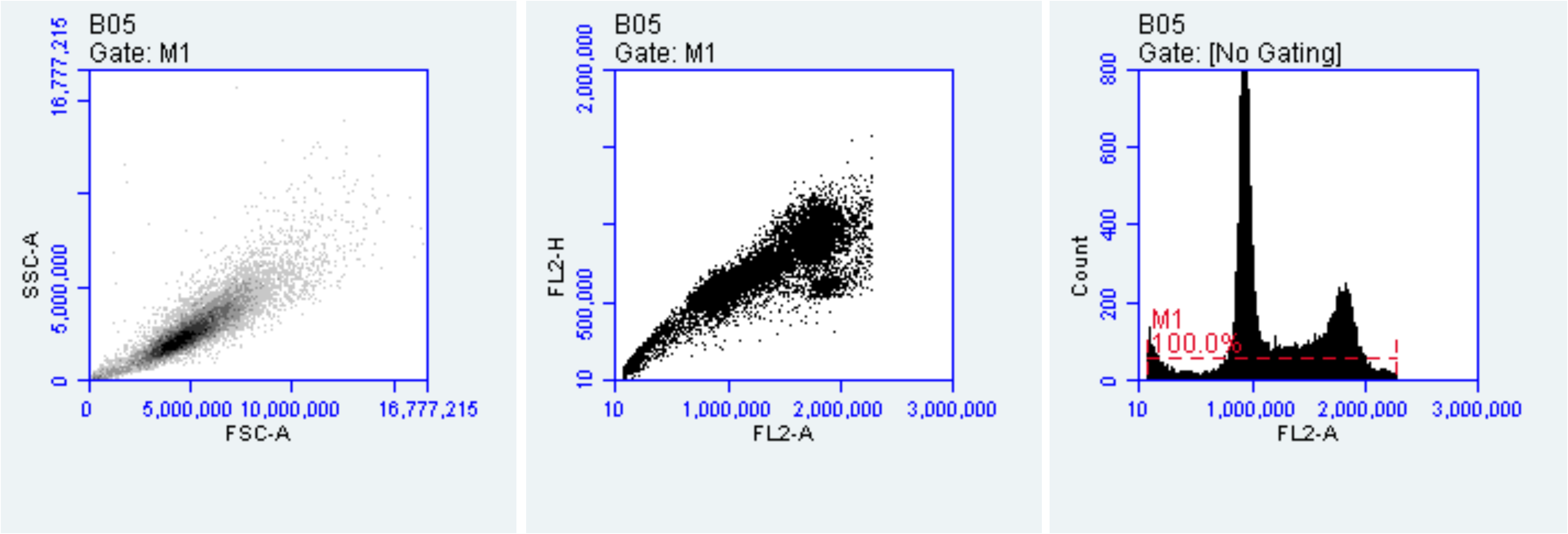

B05
Gate: M1
16,777,215
SSC-A
5,000,000
0
0
5,000,000
10,000,000
16,777,215
FSC-A
B05
Gate: M1
2,000,000
FL2-H
500,000
10
10
1,000,000
2,000,000
3,000,000
FL2-A
B05
Gate: [No Gating]
800
600
400
200
0
Count
M1
100.0%
10
1,000,000
2,000,000
3,000,000
FL2-A

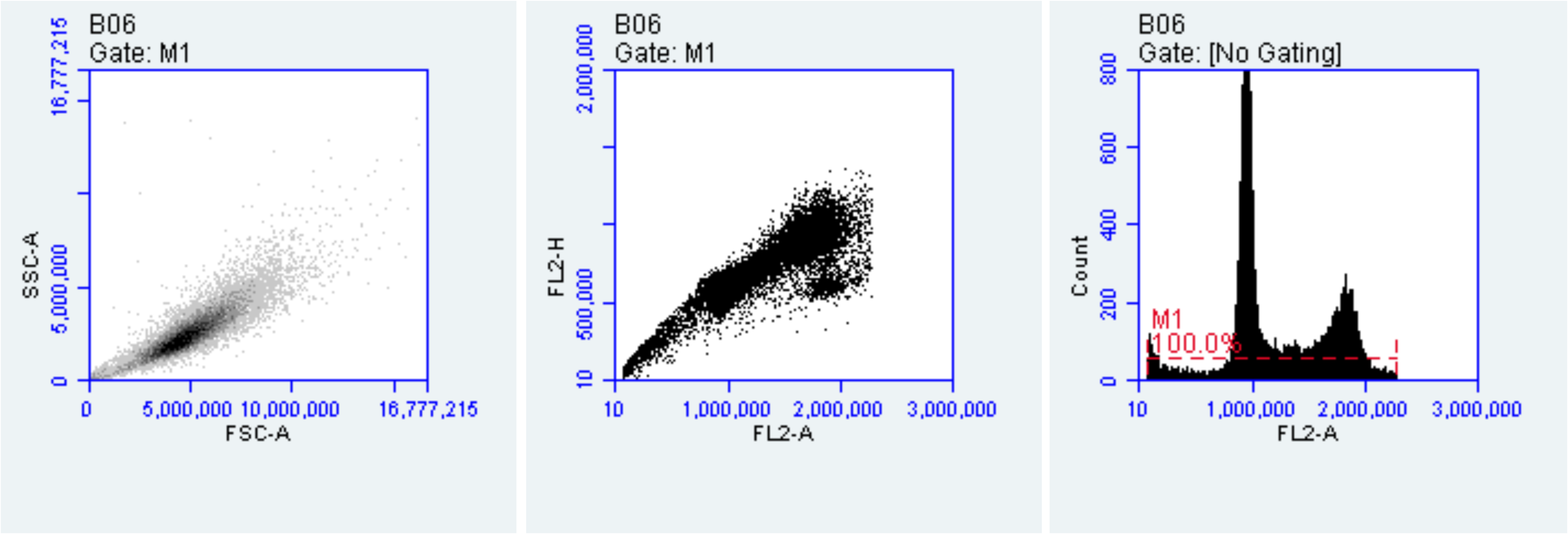
B06
Gate: M1
SSC-A
16,777,215
5,000,000
0
FSC-A
0
5,000,000
10,000,000
16,777,215
B06
Gate: M1
FL2-H
2,000,000
500,000
10
FL2-A
10
1,000,000
2,000,000
3,000,000
B06
Gate: [No Gating]
Count
800
600
400
200
0
M1
100.0%
FL2-A
10
1,000,000
2,000,000
3,000,000

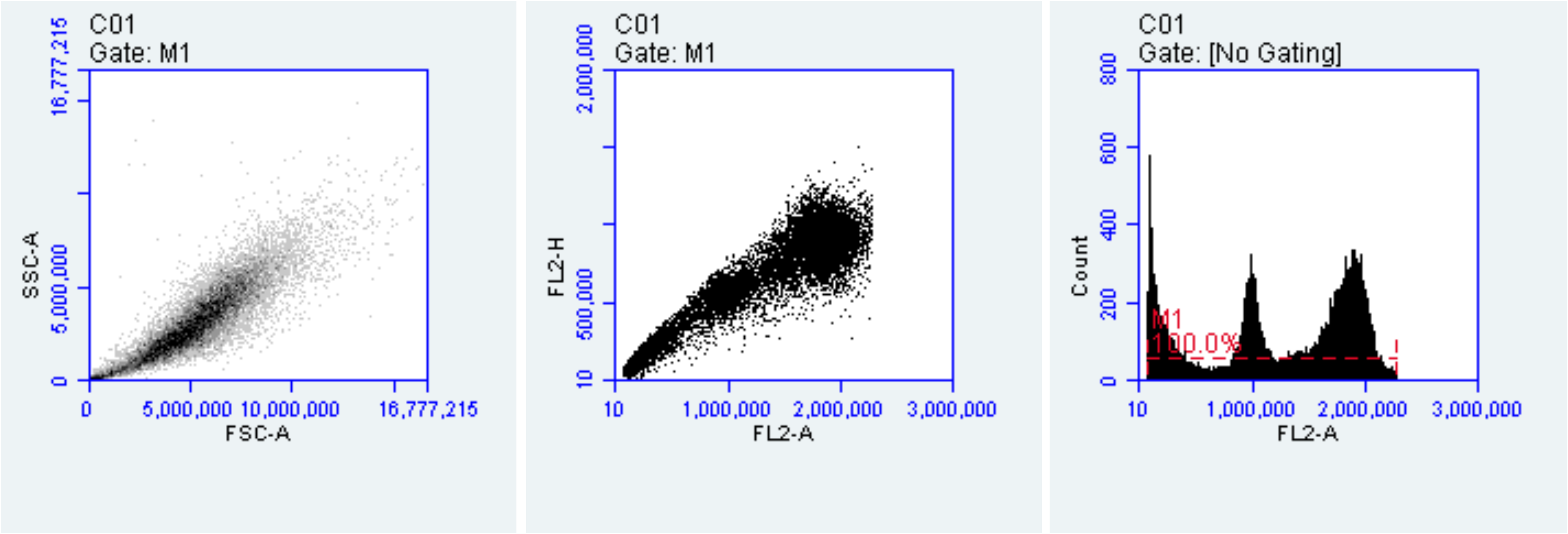
C01
Gate: M1
16,777,215
SSC-A
5,000,000
0
0
5,000,000 10,000,000
16,777,215
FSC-A
C01
Gate: M1
2,000,000
FL2-H
500,000
10
10
1,000,000
2,000,000
3,000,000
FL2-A
C01
Gate: [No Gating]
800
600
400
Count
200
0
M1
100.0%
10
1,000,000
2,000,000
3,000,000
FL2-A

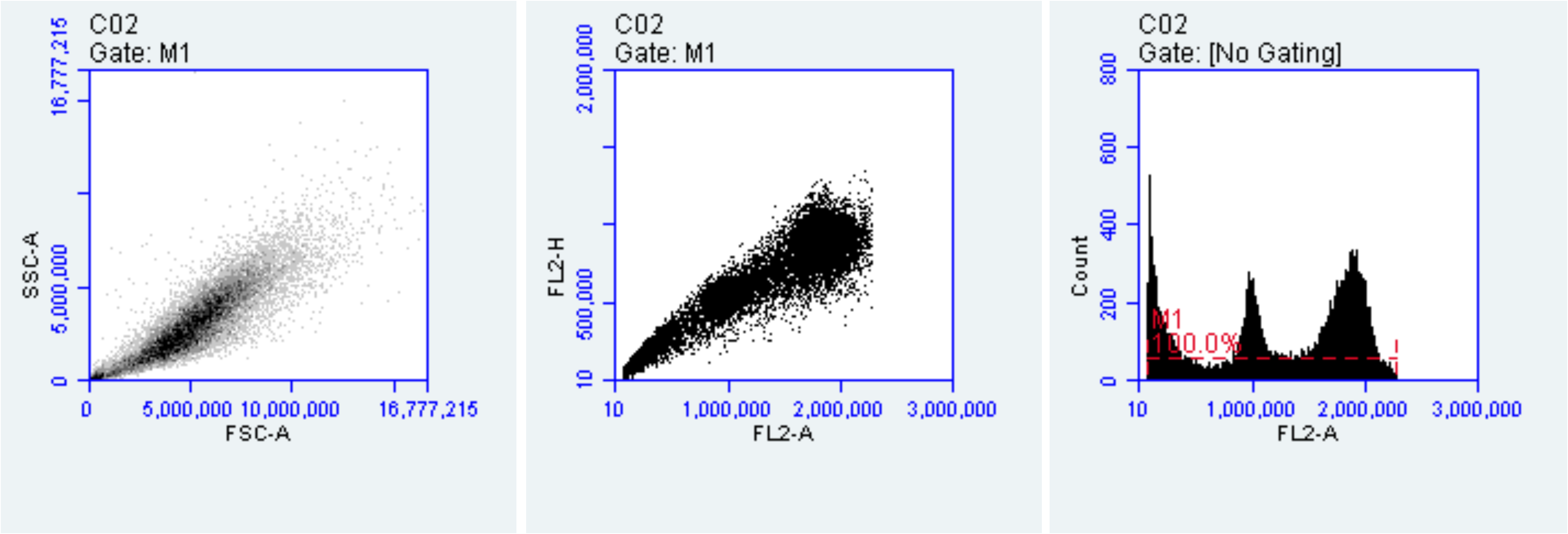
C02
Gate: M1
16,777,215
SSC-A
5,000,000
0
5,000,000
10,000,000
16,777,215
FSC-A
C02
Gate: M1
2,000,000
FL2-H
500,000
10
1,000,000
2,000,000
3,000,000
FL2-A
C02
Gate: [No Gating]
800
600
400
200
0
Count
M1
100.0%
10
1,000,000
2,000,000
3,000,000
FL2-A

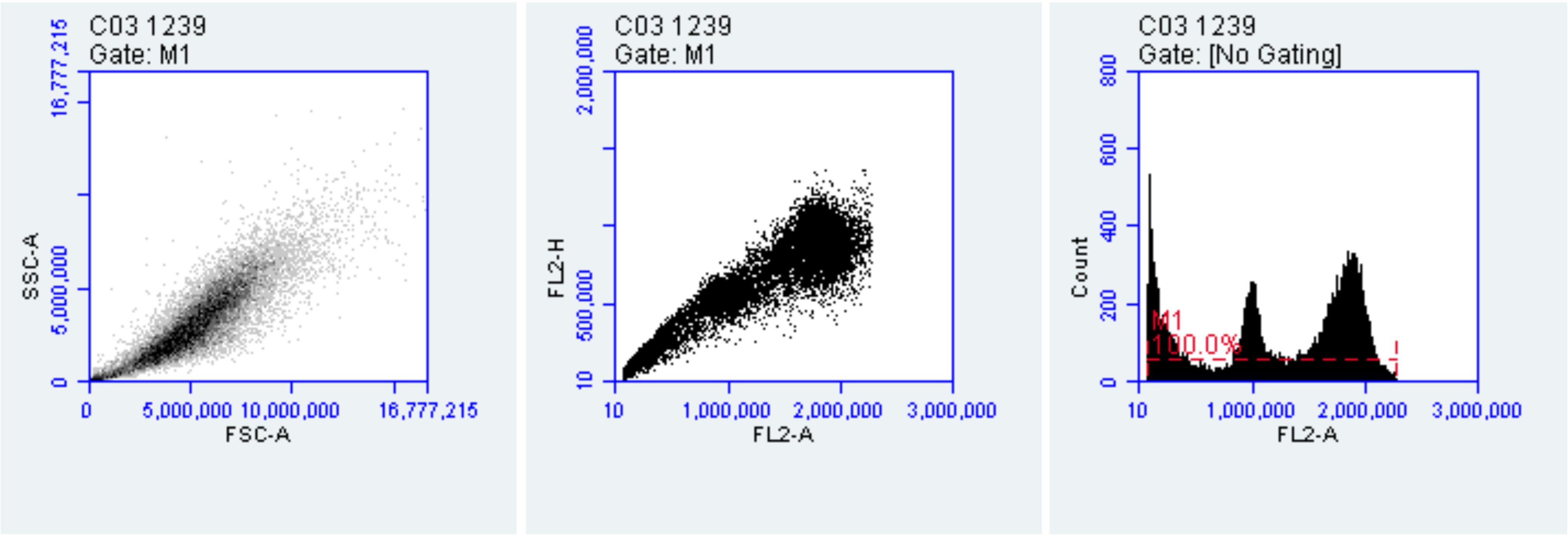
C03 1239
Gate: M1
SSC-A
FSC-A
0
5,000,000
10,000,000
16,777,215
C03 1239
Gate: M1
FL2-H
FL2-A
10
500,000
2,000,000
1,000,000
3,000,000
C03 1239
Gate: [No Gating]
Count
FL2-A
M1
100.0%
0
200
400
600
800
