# Supplementary material for: Anti-cancer stem cell activity of a sesquiterpene lactone isolated from Ambrosia arborescens and of a synthetic derivative
Source: PLoS One. 2017 Sep 1;12(9):e0184304. doi: 10.1371/journal.pone.0184304 (PMC5581169; doi:10.1371/journal.pone.0184304)
Supplement: S4 Fig — (PDF) [file pone.0184304.s004.pdf]

cyclin D JIMT1 150513

1	adjust volumen	X	adjust volumen B-actin	Y	(X/Y)*Control
control	5265,69	100,00	7928,10	100,00	100,00
damsin 1uM	5304,29	100,73	6615,37	83,44	120,72
ambrosin 1uM	3614,25	68,64	6423,33	81,02	84,72
damsin 5uM	1840,34	34,95	7020,41	88,55	39,47
ambrosin 5uM	1553,70	29,51	5331,35	67,25	43,88

cyclin D JIMT1 20150430 2

2	adjust volumen	X	adjust volumen B-actin	Y	(X/Y)*Control
control	14156,52	100,00	18812,52	100,00	100,00
damsin 1uM	12964,93	91,58	14932,98	79,38	115,38
ambrosin 1uM	9290,78	65,63	12597,94	66,97	98,00
damsin 5uM	6536,32	46,17	12974,20	68,97	66,95
ambrosin 5uM	5858,39	41,38	14497,04	77,06	53,70

cyclin D JIMT1 20150430 3

3	adjust volumen	X	adjust volumen B-actin	Y	(X/Y)*Control
control	5316,01	100,00	7928,10	100,00	100,00
damsin 1uM	7413,59	139,46	6615,37	83,44	167,13
ambrosin 1uM	5478,76	103,06	6423,33	81,02	127,21
damsin 5uM	4310,47	81,08	7020,41	88,55	91,57
ambrosin 5uM	4101,61	77,16	5331,35	67,25	114,74

cyclin D JIMT1 20150430 4

4	adjust volumen	X	adjust volumen B-actin	Y	(X/Y)*Control
control	2535,69	100,00	11410,07	100,00	100,00
damsin 1uM	3896,68	153,67	13828,00	121,19	126,80
damsin 5uM	3248,28	128,10	14438,60	126,54	101,23
ambrosin 1uM	4036,54	159,19	14514,63	127,21	125,14
ambrosin 5uM	4767,10	188,00	14341,23	125,69	149,58

Graphic

cyclin D JIMT1	test 1	test 2	test 3	test 4	Mean	SD	SEM
control	100,00	100,00	100,00	100,00	100,00	0,00	0,00
damsin 1uM	120,72	115,38	167,13	126,80	132,51	23,55	11,77
damsin 5uM	148,96	66,95	91,57	101,23	102,18	34,36	17,18
ambrosin 1uM	84,72	98,00	127,21	125,14	108,77	20,83	10,42
ambrosin 5uM	81,46	53,70	114,74	149,58	99,87	41,48	20,74

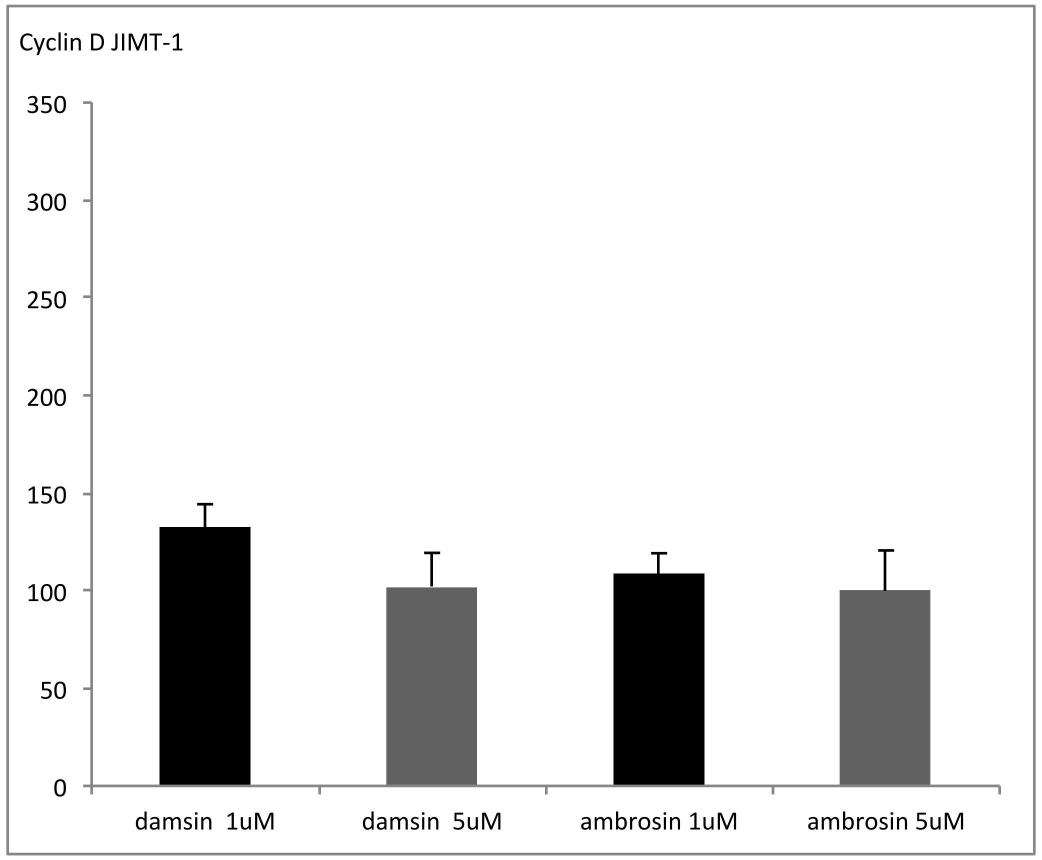

Cyclin D JIMT-1
350
300
250
200
150
100
50
0
damsin 1uM
damsin 5uM
ambrosin 1uM
ambrosin 5uM

CDK2 JIMT-1

1	adjust volumen	X	adjust volumen B-actin	Y	(X/Y)*Control
control	13884,21	100,00	7244,40	100,00	100,00
damsin 1uM	9216,74	66,38	12096,27	166,97	39,76
ambrosin 1uM	8959,87	64,53	13072,27	180,45	35,76
damsin 5uM	11620,27	83,69	11567,13	159,67	52,42
ambrosin 5uM	10168,97	73,24	14324,67	197,73	37,04

CDK2 JIMT-1

2	adjust volumen	X	adjust volumen B-actin	Y	(X/Y)*Control
control	13665,52	100,00	18260,38	100,00	100,00
damsin 1uM	12969,54	94,91	18207,29	99,71	95,18
ambrosin 1uM	9345,50	68,39	17930,92	98,20	69,64
damsin 5uM	7446,14	54,49	11931,45	65,34	83,39
ambrosin 5uM	2965,58	21,70	17717,92	97,03	22,37

CDK2 JIMT-1

3	adjust volumen	X	adjust volumen B-actin	Y	(X/Y)*Control
control	8549,72	100,00	8945,41	100,00	100,00
damsin 1uM	8718,63	101,98	9663,86	108,03	94,39
ambrosin 1uM	9809,44	114,73	12180,77	136,17	84,26
damsin 5uM	7730,21	90,41	11745,45	131,30	68,86
ambrosin 5uM	7290,06	85,27	13741,83	153,62	55,51

Graphic

CDK2 JIMT-1	test 1	test 2	test 3	Mean	SD	SEM
control	100,00	100,00	100,00	100,00	0,00	0,00
damsin 1uM	39,76	95,18	94,39	76,44	31,78	18,35
damsin 5uM	52,42	83,39	68,86	68,22	15,50	8,95
ambrosin 1uM	35,76	69,64	84,26	63,22	24,88	14,36
ambrosin 5uM	37,04	22,37	55,51	38,30	16,61	9,59

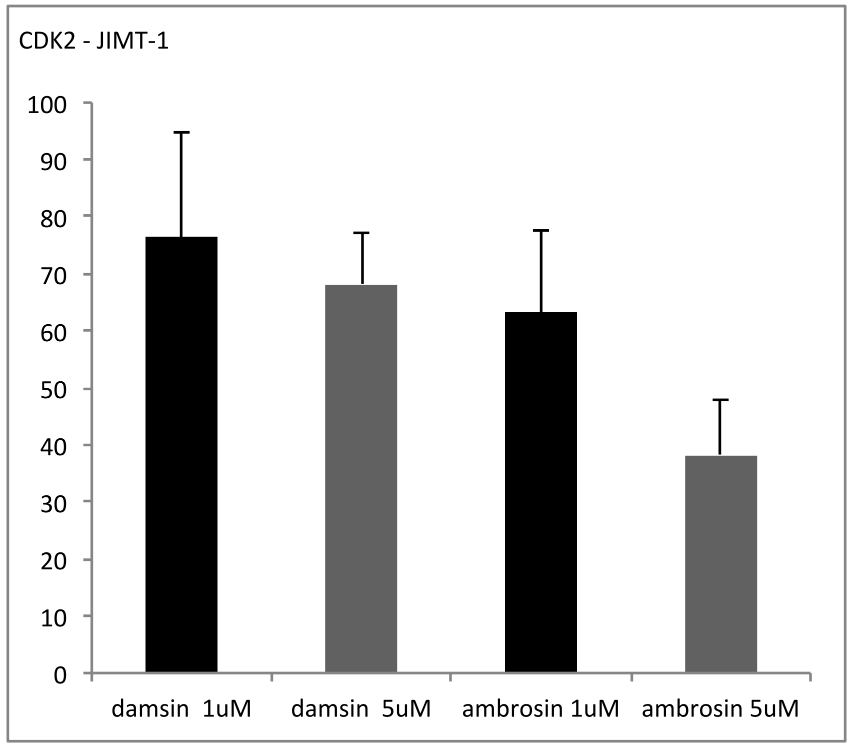
CDK2 - JIMT-1
100
90
80
70
60
50
40
30
20
10
0
damsin 1uM
damsin 5uM
ambrosin 1uM
ambrosin 5uM

P53 MCF-7 1

1	adjust volumen	X	adjust volumen B-actin	Y	(X/Y)*Control
control	10843,78	100,00	11498,21	100,00	100,00
damsin 1uM	10732,74	98,98	7801,92	67,85	145,87
ambrosin 1uM	13930,80	128,47	12394,06	107,79	119,18
damsin 5uM	11103,45	102,39	11464,34	99,71	102,70
ambrosin 5uM	13084,95	120,67	8876,12	77,20	156,31

P53 MCF-7 2

2	adjust volumen	X	adjust volumen B-actin	Y	(X/Y)*Control
control	6231,84	100,00	12117,87	100,00	100,00
damsin 1uM	8526,28	136,82	10302,33	85,02	160,93
ambrosin 1uM	12408,60	199,12	11006,39	90,83	219,22
damsin 5uM	8650,26	138,81	8640,11	71,30	194,68
ambrosin 5uM	13353,71	214,28	7783,69	64,23	333,60

P53 MCF-7 3

3	adjust volumen	X	adjust volumen B-actin	Y	(X/Y)*Control
control	8178,93	100,00	18650,34	100,00	100,00
damsin 1uM	11029,74	134,86	19427,73	104,17	129,46
ambrosin 1uM	9249,76	113,09	13845,59	74,24	152,34
damsin 5uM	13223,53	161,68	15392,34	82,53	195,90
ambrosin 5uM	17037,27	208,31	18181,49	97,49	213,68

P53 MCF-7 4

4	adjust volumen	X	adjust volumen B-actin	Y	(X/Y)*Control
control	7044,99	100,00	19082,72	100,00	100,00
damsin 1uM	5243,26	74,43	17600,21	92,23	80,69
damsin 5uM	10015,90	142,17	20983,65	109,96	129,29
ambrosin 1uM	7667,88	108,84	24782,60	129,87	83,81
ambrosin 5uM	8418,84	119,50	25856,49	135,50	88,19

Graphic

P53 MCF-7	test 1	test 2	test 3	test 4	Mean	SD	SEM
control	100,00	100,00	100,00	100,00	100,00	0,00	0,00
damsin 1uM	145,87	160,93	129,46	80,69	129,24	34,82	17,41
damsin 5uM	102,70	194,68	195,90	129,29	155,64	47,05	23,53
ambrosin 1uM	119,18	219,22	152,34	83,81	143,64	57,64	28,82
ambrosin 5uM	156,31	333,60	213,68	88,19	197,95	103,97	51,98

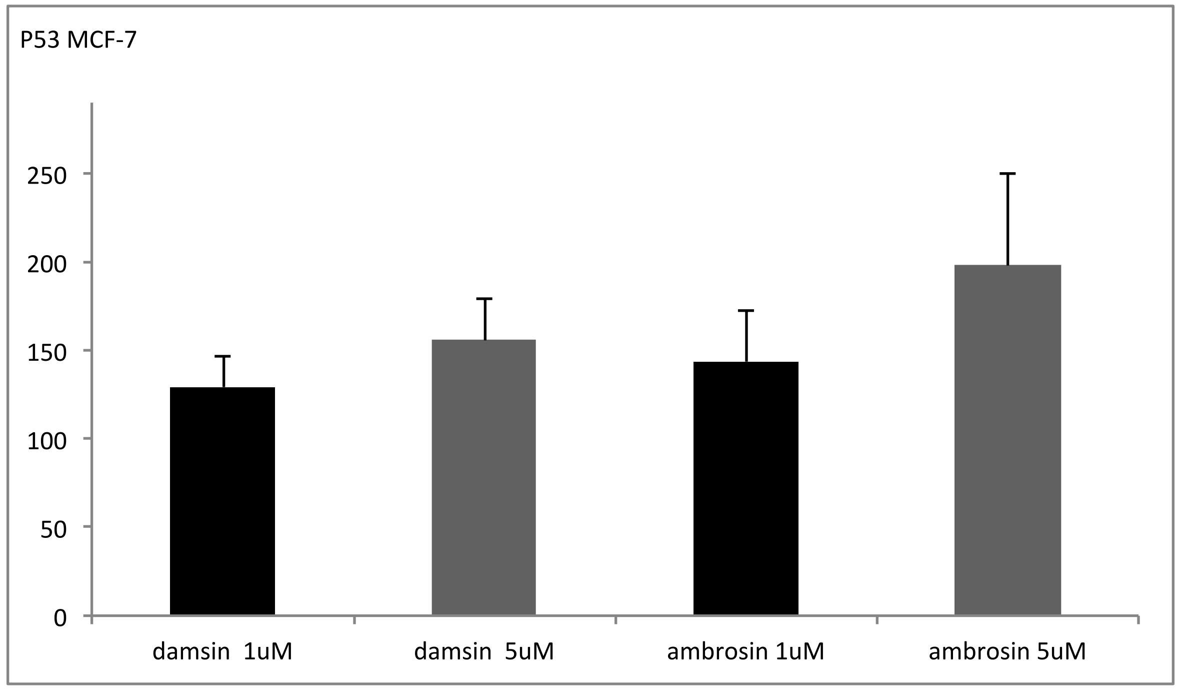
P53 MCF-7
250
200
150
100
50
0
damsin 1uM
damsin 5uM
ambrosin 1uM
ambrosin 5uM

P21 MCF-7 20140701

1	adjust volumen	X	adjust volumen B-actin	Y	(X/Y)*Control
control	2912,98	100,00	11498,21	100,00	100,00
damsin 1uM	2724,31	93,52	7801,92	67,85	137,83
ambrosin 1uM	2557,31	87,79	12394,06	107,79	81,44
damsin 5uM	6411,12	220,09	11464,34	99,71	220,74
ambrosin 5uM	3853,41	132,28	8876,12	77,20	171,36

P21 MCF-7 20140820 - 2

2	adjust volumen	X	adjust volumen B-actin	Y	(X/Y)*Control
control	1389,19	100,00	12117,87	100,00	100,00
damsin 1uM	3075,55	221,39	10302,33	85,02	260,41
ambrosin 1uM	6472,73	465,94	11006,39	90,83	512,99
damsin 5uM	2300,20	165,58	8640,11	71,30	232,23
ambrosin 5uM	2946,11	212,07	7783,69	64,23	330,16

P21 MCF-7 20151103

3	adjust volumen	X	adjust volumen B-actin	Y	(X/Y)*Control
control	3347,19	100,00	6942,98	100,00	100,00
damsin 1uM	4447,88	132,88	6921,54	99,69	133,30
damsin 5uM	5484,82	163,86	9714,81	139,92	117,11
ambrosin 1uM	11281,65	337,05	10196,62	146,86	229,50
ambrosin 5uM	5583,31	166,81	8875,05	127,83	130,49

P21 MCF-7 20151103

4	adjust volumen	X	adjust volumen B-actin	Y	(X/Y)*Control
control	4303,43	100,00	5097,47	100,00	100,00
damsin 1uM	5439,09	126,39	5516,77	108,23	116,78
damsin 5uM	8001,24	185,93	7743,53	151,91	122,39
ambrosin 1uM	8681,54	201,74	10552,84	207,02	97,45
ambrosin 5uM	11351,89	263,79	11621,18	227,98	115,71

P21 MCF-7 20151103

5	adjust volumen	X	adjust volumen B-actin	Y	(X/Y)*Control
control	5379,20	100,00	13437,29	100,00	100,00
damsin 1uM	5650,01	105,03	9459,32	70,40	149,20
ambrosin 1uM	3684,83	68,50	5113,50	38,05	180,01
damsin 5uM	3442,04	63,99	4763,27	35,45	180,51
ambrosin 5uM	10098,22	187,73	7345,40	54,66	343,42

Graphic

P21 MCF-7	test 1	test 2	test 3	test 4	test 5	Mean	SD	SEM
control	100,00	100,00	100,00	100,00	100,00	100,00	0,00	0,00
damsin 1uM	137,83	260,41	133,30	116,78	149,20	159,50	57,60	25,76
damsin 5uM	220,74	232,23	117,11	122,39	101,23	158,74	62,46	27,93
ambrosin 1uM	81,44	180,01	229,50	97,45	180,01	153,68	62,28	27,85
ambrosin 5uM	171,36	330,16	130,49	115,71	80,33	165,61	97,60	43,65

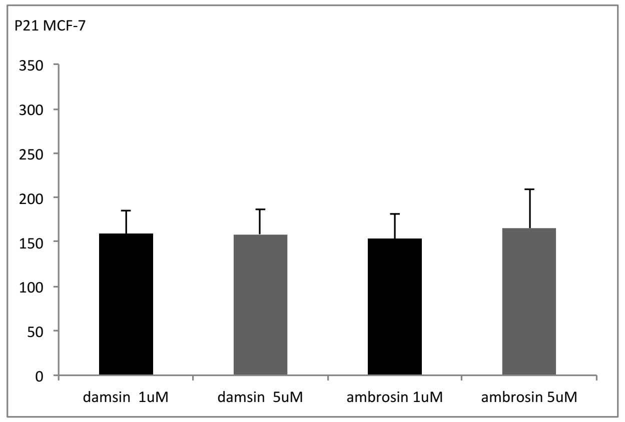

P53 JIMT-1 1

1	adjust volumen	X	adjust volumen B-actin	Y	(X/Y)*Control
control	3880,76	100,00	14181,90	100,00	100,00
damsin 1uM	4423,34	113,98	12222,68	86,19	132,25
ambrosin 1uM	5929,02	152,78	10628,52	74,94	203,86
damsin 5uM	4331,88	111,62	12618,10	88,97	125,46
ambrosin 5uM	3047,51	78,53	9946,54	70,14	111,97

P53 JIMT-1 2

2	adjust volumen	X	adjust volumen B-actin	Y	(X/Y)*Control
control	13015,26	100,00	13453,45	100,00	100,00
damsin 1uM	12173,34	93,53	9811,29	72,93	128,25
ambrosin 1uM	10180,23	78,22	7129,88	53,00	147,59
damsin 5uM	7036,72	54,07	7381,57	54,87	98,54
ambrosin 5uM	7047,62	54,15	8489,30	63,10	85,81

P53 JIMT-1 3

3	adjust volumen	X	adjust volumen B-actin	Y	(X/Y)*Control
control	3307,00	100,00	12885,43	100,00	100,00
damsin 1uM	3597,76	108,79	10020,10	77,76	139,90
ambrosin 1uM	3836,87	116,02	9623,12	74,68	155,36
damsin 5uM	4241,56	128,26	8230,14	63,87	200,81
ambrosin 5uM	3734,84	112,94	9805,22	76,10	148,42

P53 JIMT-1 4

4	adjust volumen	X	adjust volumen B-actin	Y	(X/Y)*Control
control	9512,15	100,00	12753,00	100,00	100,00
damsin 1uM	10557,94	110,99	12481,34	97,87	113,41
damsin 5uM	13037,35	137,06	15497,45	121,52	112,79
ambrosin 1uM	10757,72	113,09	17172,33	134,65	83,99
ambrosin 5uM	10872,87	114,31	17893,27	140,31	81,47

Graphic

	test 1	test 2	test 3	test 4	Mean	SD	SEM
control	100,00	100,00	100,00	100,00	100,00	0,00	0,00
damsin 1uM	132,25	128,25	139,90	113,41	128,45	11,13	5,57
damsin 5uM	125,46	98,54	200,81	112,79	134,40	45,62	22,81
ambrosin 1uM	203,86	147,59	155,36	83,99	147,70	49,23	24,62
ambrosin 5uM	111,97	85,81	148,42	81,47	106,92	30,77	15,39

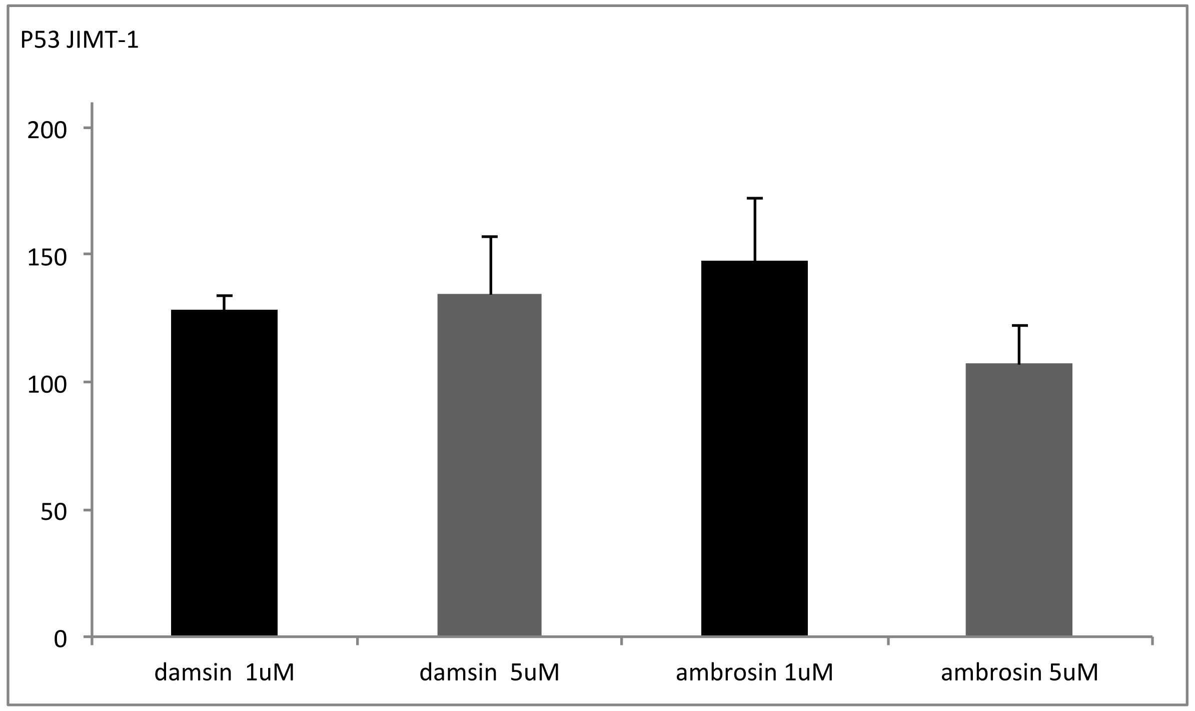
P53 JIMT-1
200
150
100
50
0
damsin 1uM
damsin 5uM
ambrosin 1uM
ambrosin 5uM
