# Supplementary material for: Anti-cancer stem cell activity of a sesquiterpene lactone isolated from Ambrosia arborescens and of a synthetic derivative
Source: PLoS One. 2017 Sep 1;12(9):e0184304. doi: 10.1371/journal.pone.0184304 (PMC5581169; doi:10.1371/journal.pone.0184304)
Supplement: S5 Fig — (PDF) [file pone.0184304.s005.pdf]

Nfκβ P65 MCF-7 20140701

1	adjust volumen	X	adjust volumen B-actin	Y	(X/Y)*Control
control	2808,22	100,00	17369,61	100,00	100,00
damsin 1uM	2333,28	83,09	14318,75	82,44	100,79
ambrosin 1uM	3080,07	109,68	12967,03	74,65	146,92
damsin 5uM	1916,04	68,23	12242,10	70,48	96,81
ambrosin 5uM	2178,96	77,59	11064,32	63,70	121,81

Nfκβ P65 MCF-7 20140821 - 2

2	adjust volumen	X	adjust volumen B-actin	Y	(X/Y)*Control
control	3433,79	100,00	11679,87	100,00	100,00
damsin 1uM	3517,66	102,44	12080,21	103,43	99,05
ambrosin 1uM	2792,11	81,31	14145,99	121,11	67,14
damsin 5uM	2202,60	64,14	8482,03	72,62	88,33
ambrosin 5uM	1550,15	45,14	7530,53	64,47	70,02

Nfκβ P65 MCF-7 20140821 - 3

3	adjust volumen	X	adjust volumen B-actin	Y	(X/Y)*Control
control	1972,08	100,00	12931,42	100,00	100,00
damsin 1uM	1495,98	75,86	4767,25	36,87	205,77
ambrosin 1uM	1848,74	93,75	11698,33	90,46	103,63
damsin 5uM	1168,22	59,24	12303,28	95,14	62,26
ambrosin 5uM	319,03	16,18	15580,89	120,49	13,43

Nfκβ P65 MCF-7

4	adjust volumen	X	adjust volumen B-actin	Y	(X/Y)*Control
control	15511,26	100,00	11977,75	100,00	100,00
damsin 1uM	11626,57	74,96	12695,50	105,99	70,72
damsin 5uM	13269,39	85,55	12397,49	103,50	82,65
ambrosin 1uM	17522,93	112,97	12194,26	101,81	110,96
ambrosin 5uM	14578,12	93,98	11828,81	98,76	95,17

Nfκβ P65 MCF-7

5	adjust volumen	X	adjust volumen B-actin	Y	(X/Y)*Control
control	4802,33	100,00	14019,40	100,00	100,00
damsin 1uM	6721,80	139,97	12275,52	87,56	159,85
damsin 5uM	10662,24	222,02	14924,42	106,46	208,56
ambrosin 1uM	10593,25	220,59	13847,37	98,77	223,33
ambrosin 5uM	7897,92	164,46	18287,55	130,44	126,08

Nfκβ P65 MCF-7

6	adjust volumen	X	adjust volumen B-actin	Y	(X/Y)*Control
control	9923,99	100,00	19304,73	100,00	100,00
damsin 1uM	10446,97	105,27	17929,84	92,88	113,34
damsin 5uM	14600,03	147,12	21334,36	110,51	133,12
ambrosin 1uM	15637,64	157,57	24968,49	129,34	121,83
ambrosin 5uM	12137,53	122,30	25620,31	132,72	92,16

Graphic

Nfκβ P65 MCF-7	test 1	test 2	test 3	test 4	test 5	test 6	Mean	SD	SEM
control	100,00	100,00	100,00	100,00	100,00	100,00	100,00	0,00	0,00
damsin 1uM	100,79	99,05	205,77	70,72	159,85	113,34	124,92	49,15	20,07
damsin 5uM	96,81	88,33	62,26	82,65	208,56	133,12	111,95	52,72	21,52
ambrosin 1uM	146,92	67,14	103,63	110,96	223,33	121,83	128,97	53,04	21,65
ambrosin 5uM	121,81	70,02	13,43	95,17	126,08	92,16	86,44	41,31	16,86

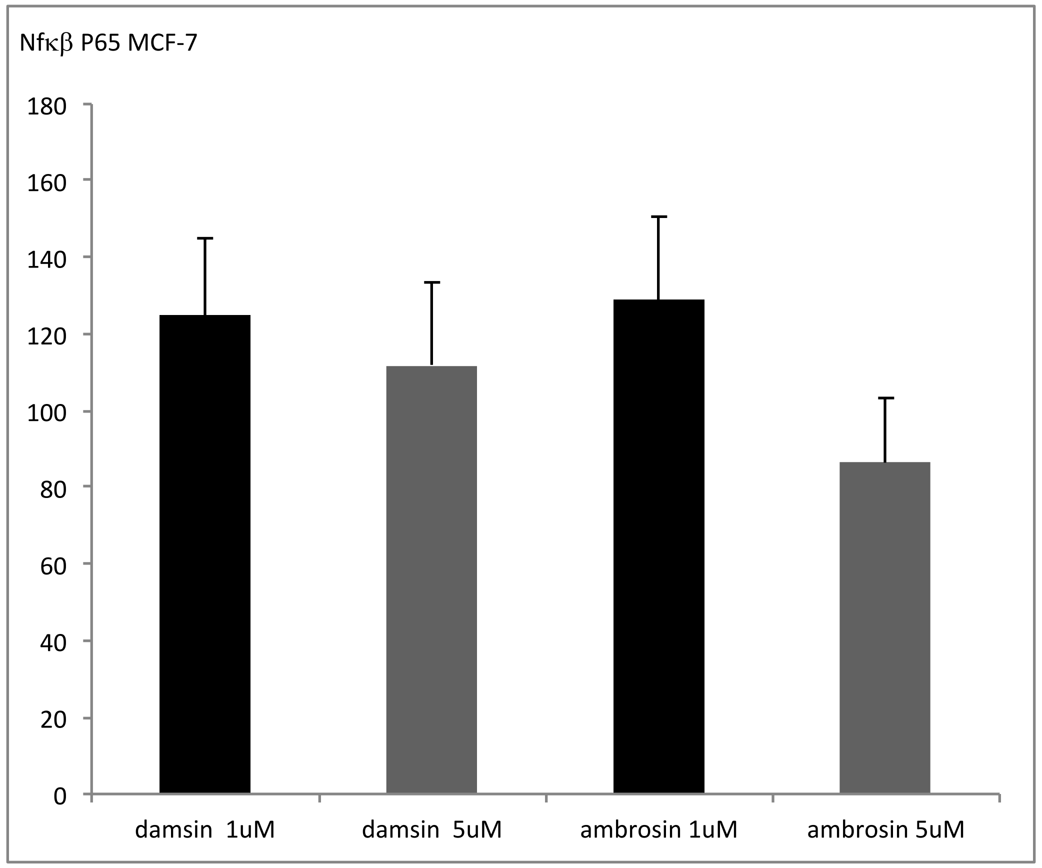

Iκβα MCF-7 20140701

1	adjust volumen	X	adjust volumen B-actin	Y	(X/Y)*Control
control	8793,46	100,00	16617,01	100,00	100,00
damsin 1uM	9478,51	107,79	12977,06	78,10	138,02
ambrosin 1uM	14380,28	163,53	11384,75	68,51	238,69
damsin 5uM	12109,72	137,71	8721,82	52,49	262,37
ambrosin 5uM	10744,11	122,18	9071,64	54,59	223,81

Iκβα MCF-7 20140821 -1

2	adjust volumen	X	adjust volumen B-actin	Y	(X/Y)*Control
control	2714,91	100,00	9856,60	100,00	100,00
damsin 1uM	3807,20	140,23	9073,28	92,05	152,34
ambrosin 1uM	5781,51	212,95	8480,35	86,04	247,51
damsin 5uM	4302,62	158,48	6872,13	69,72	227,31
ambrosin 5uM	3658,58	134,76	9085,61	92,18	146,19

Iκβα MCF-7 20140821 -2

3	adjust volumen	X	adjust volumen B-actin	Y	(X/Y)*Control
control	7904,27	100,00	14436,33	100,00	100,00
damsin 1uM	9415,87	119,12	16018,38	110,96	107,36
ambrosin 1uM	7205,00	91,15	11292,40	78,22	116,53
damsin 5uM	8129,04	102,84	13165,44	91,20	112,77
ambrosin 5uM	8023,98	101,51	14473,72	100,26	101,25

Graphic

Iκβα MCF-7	test 1	test 2	test 3	Mean	SD	SEM
control	100,00	100,00	100,00	100,00	0,00	0,00
damsin 1uM	138,02	152,34	107,36	132,57	22,98	13,27
damsin 5uM	262,37	227,31	112,77	200,82	78,24	45,17
ambrosin 1uM	238,69	247,51	116,53	200,91	73,21	42,27
ambrosin 5uM	223,81	146,19	101,25	157,09	62,00	35,80

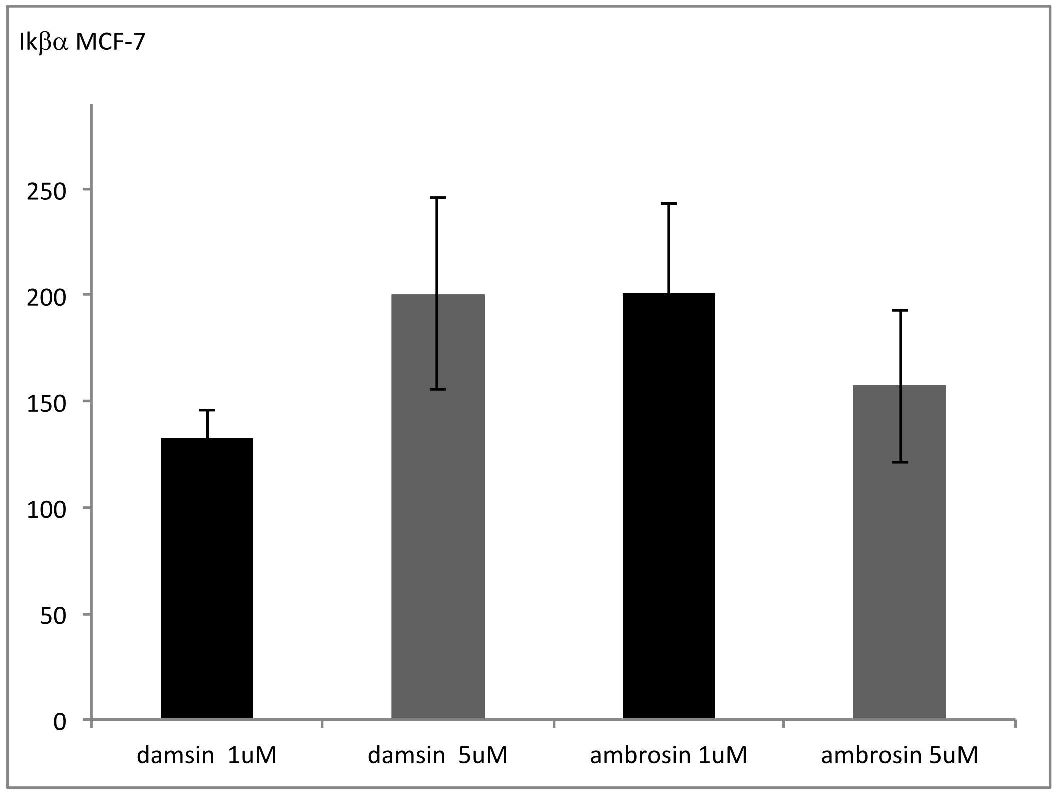
Ikβα MCF-7
250
200
150
100
50
0
damsin 1uM
damsin 5uM
ambrosin 1uM
ambrosin 5uM

pIκβα MCF-7	20140821 -2				
1	adjust volumen	X	adjust volumen B-actin	Y	(X/Y)*Control
control	6410,70	100,00	13083,19	100,00	100,00
damsin 1uM	9768,06	152,37	16472,49	125,91	121,02
ambrosin 1uM	6862,62	107,05	12884,37	98,48	108,70
damsin 5uM	8516,36	132,85	12710,16	97,15	136,74
ambrosin 5uM	2686,82	41,91	11285,86	86,26	48,59

pIκβα MCF-7	20140821 -3				
2	adjust volumen	X	adjust volumen B-actin	Y	(X/Y)*Control
control	9381,75	100,00	6809,75	100,00	100,00
damsin 1uM	8410,85	89,65	7766,23	114,05	78,61
ambrosin 1uM	10839,91	115,54	7968,31	117,01	98,74
damsin 5uM	4131,29	44,04	7590,63	111,47	39,51
ambrosin 5uM	3782,17	40,31	7630,03	112,05	35,98

pIκβα MCF-7	20140822				
3	adjust volumen	X	adjust volumen B-actin	Y	(X/Y)*Control
control	7734,05	100,00	10568,45	100,00	100,00
damsin 1uM	7556,70	97,71	11842,19	112,05	87,20
ambrosin 1uM	9941,66	128,54	9229,09	87,33	147,20
damsin 5uM	6649,57	85,98	10721,73	101,45	84,75
ambrosin 5uM	6043,45	78,14	10912,99	103,26	75,67

Graphic

pIκβα MCF-7	test 1	test 2	test 3	Mean	SD	SEM
control	100,00	100,00	100,00	100,00	0,00	0,00
damsin 1uM	121,02	78,61	87,20	95,61	22,42	12,95
damsin 5uM	136,74	39,51	84,75	87,00	48,66	28,09
ambrosin 1uM	108,70	98,74	147,20	118,21	25,59	14,77
ambrosin 5uM	48,59	35,98	75,67	53,41	20,28	11,71

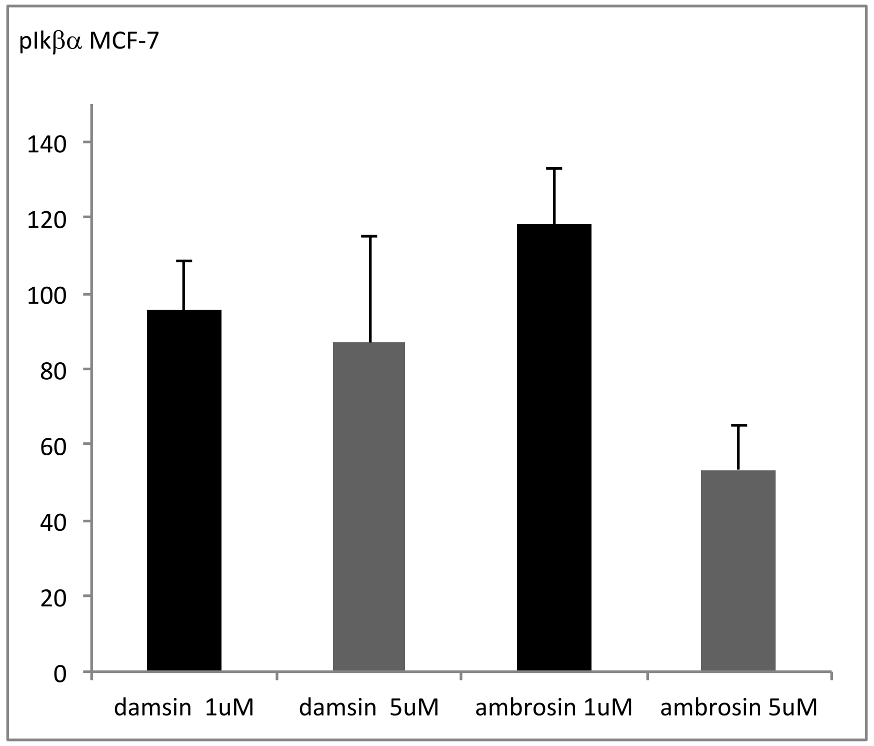

pIkβα MCF-7
140
120
100
80
60
40
20
0
damsin 1uM
damsin 5uM
ambrosin 1uM
ambrosin 5uM

Nfκβ P65 JIMT-1 1

1	adjust volumen	X	adjust volumen B-actin	Y	(X/Y)*Control
control	3410,39	100,00	15368,59	100,00	100,00
damsin 1uM	3590,38	105,28	14513,48	94,44	111,48
ambrosin 1uM	4183,48	122,67	13059,36	84,97	144,36
damsin 5uM	4948,99	145,12	9257,11	60,23	240,92
ambrosin 5uM	5496,63	161,17	12271,46	79,85	201,85

Nfκβ P65 JIMT-1 2

2	adjust volumen	X	adjust volumen B-actin	Y	(X/Y)*Control
control	2228,49	100,00	13208,78	100,00	100,00
damsin 1uM	2556,68	114,73	11737,83	88,86	129,10
ambrosin 1uM	1830,36	82,13	12151,13	91,99	89,28
damsin 5uM	2481,05	111,33	10208,52	77,29	144,05
ambrosin 5uM	2913,64	130,75	11515,62	87,18	149,97

Nfκβ P65 JIMT-1 3

3	adjust volumen	X	adjust volumen B-actin	Y	(X/Y)*Control
control	4148,79	100,00	14335,21	100,00	100,00
damsin 1uM	4305,86	103,79	12287,81	85,72	121,08
ambrosin 1uM	5037,06	121,41	12072,39	84,21	144,17
damsin 5uM	3017,16	72,72	11085,89	77,33	94,04
ambrosin 5uM	1964,30	47,35	9525,89	66,45	71,25

Graphic

Nfκβ P65 JIMT-1	test 1	test 2	test 3	Mean	SD	SEM
control	100,00	100,00	100,00	100,00	0,00	0,00
damsin 1uM	111,48	129,10	121,08	120,55	8,82	5,09
damsin 5uM	240,92	144,05	94,04	159,67	74,67	43,11
ambrosin 1uM	144,36	89,28	144,17	125,94	31,74	18,33
ambrosin 5uM	201,85	149,97	71,25	141,02	65,76	37,97

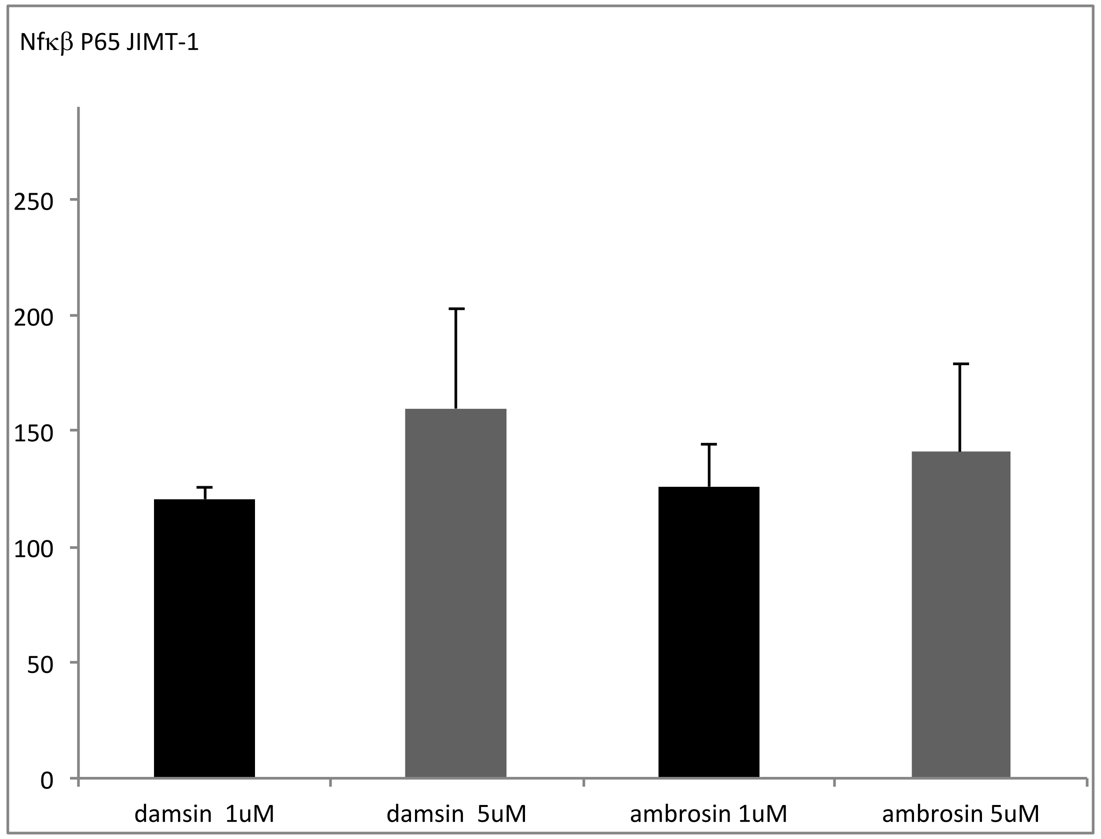
Nfκβ P65 JIMT-1
250
200
150
100
50
0
damsin 1uM
damsin 5uM
ambrosin 1uM
ambrosin 5uM

Iκβα JIMT-1 1

1	adjust volumen	X	adjust volumen B-actin	Y	(X/Y)*Control
control	9065,80	100,00	15919,72	100,00	100,00
damsin 1uM	7329,87	80,85	12326,58	77,43	104,42
ambrosin 1uM	7658,06	84,47	6979,56	43,84	192,67
damsin 5uM	7697,87	84,91	8032,13	50,45	168,29
ambrosin 5uM	8039,38	88,68	8314,56	52,23	169,79

Iκβα JIMT-1 2

2	adjust volumen	X	adjust volumen B-actin	Y	(X/Y)*Control
control	5690,03	100,00	17570,67	100,00	100,00
damsin 1uM	5265,98	92,55	14198,82	80,81	114,53
ambrosin 1uM	6683,66	117,46	12471,65	70,98	165,49
damsin 5uM	7854,62	138,04	12120,28	68,98	200,12
ambrosin 5uM	5368,08	94,34	12230,84	69,61	135,53

Iκβα JIMT-1 3

3	adjust volumen	X	adjust volumen B-actin	Y	(X/Y)*Control
control	9613,41	100,00	11075,48	100,00	100,00
damsin 1uM	9958,34	103,59	12421,67	112,15	92,36
ambrosin 1uM	8869,62	92,26	11271,27	101,77	90,66
damsin 5uM	7558,09	78,62	10928,64	98,67	79,68
ambrosin 5uM	7432,07	77,31	8766,97	79,16	97,67

Iκβα JIMT-1 4

1	adjust volumen	X	adjust volumen B-actin	Y	(X/Y)*Control
control	9629,16	100,00	1388,88	100,00	100,00
damsin 1uM	10210,82	106,04	1479,35	106,51	99,56
damsin 5uM	9539,87	99,07	1672,81	120,44	82,26
ambrosin 1uM	11997,69	124,60	1720,15	123,85	100,60
ambrosin 5uM	9302,48	96,61	1983,80	142,83	67,64

Graphic

Iκβα JIMT-1	test 1	test 2	test 3	test 4	Mean	SD	SEM
control	100,00	100,00	100,00	100,00	100,00	0,00	0,00
damsin 1uM	104,42	114,53	92,36	99,56	102,72	9,30	4,65
damsin 5uM	168,29	200,12	79,68	82,26	132,59	61,01	30,51
ambrosin 1uM	192,67	165,49	90,66	100,60	137,36	49,61	24,80
ambrosin 5uM	169,79	135,53	97,67	67,64	117,66	44,49	22,25

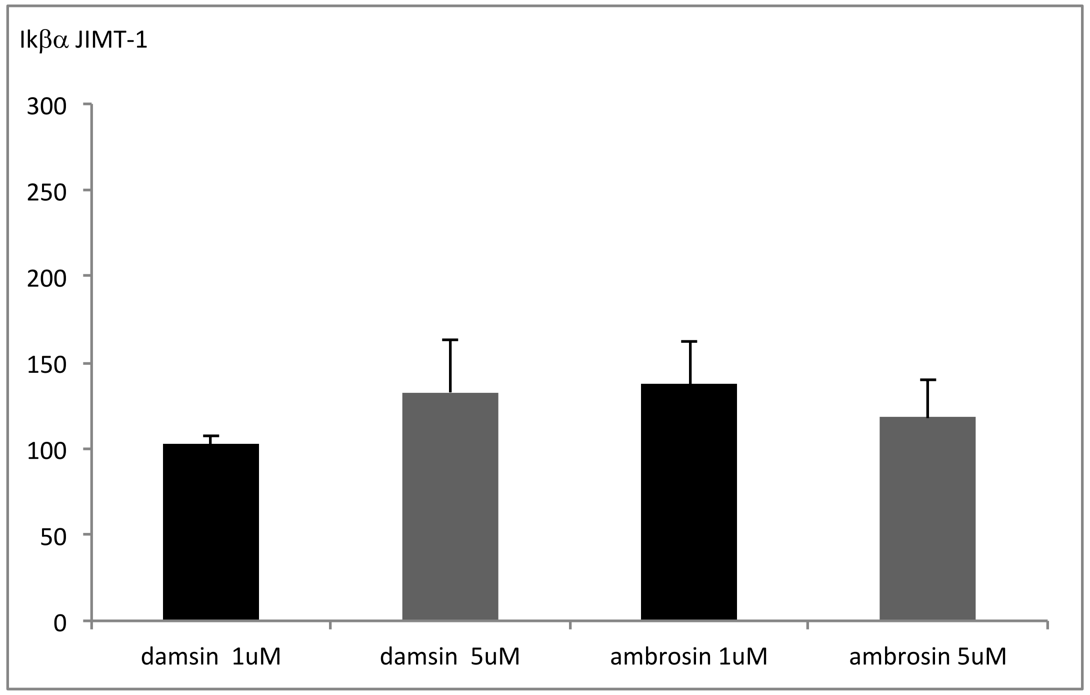

Ikβα JIMT-1
300
250
200
150
100
50
0
damsin 1uM
damsin 5uM
ambrosin 1uM
ambrosin 5uM

pIκβα JIMT-1 1

1	adjust volumen	X	adjust volumen B-actin	Y	(X/Y)*Control
control	8275,45	100,00	15131,89	100,00	100,00
damsin 1uM	7060,38	85,32	11791,09	77,92	109,49
ambrosin 1uM	8675,82	104,84	11994,14	79,26	132,26
damsin 5uM	6563,45	79,31	10186,04	67,32	117,82
ambrosin 5uM	7181,82	86,78	10613,96	70,14	123,73

pIκβα JIMT-1 2

2	adjust volumen	X	adjust volumen B-actin	Y	(X/Y)*Control
control	2779,58	100,00	13039,51	100,00	100,00
damsin 1uM	3091,24	111,21	10589,92	81,21	136,94
ambrosin 1uM	2904,52	104,49	9693,34	74,34	140,57
damsin 5uM	2705,40	97,33	8761,16	67,19	144,86
ambrosin 5uM	1777,92	63,96	8031,04	61,59	103,85

pIκβα JIMT-1 3

3	adjust volumen	X	adjust volumen B-actin	Y	(X/Y)*Control
control	11187,28	100,00	13224,72	100,00	100,00
damsin 1uM	10304,17	92,11	12738,80	96,33	95,62
ambrosin 1uM	11000,81	98,33	14471,06	109,42	89,86
damsin 5uM	10459,39	93,49	13330,69	100,80	92,75
ambrosin 5uM	8426,63	75,32	11134,22	84,19	89,47

pIκβα JIMT-1 4

1	adjust volumen	X	adjust volumen B-actin	Y	(X/Y)*Control
control	8371,14	100,00	15434,78	100,00	100,00
damsin 1uM	11826,25	141,27	16193,73	104,92	134,65
damsin 5uM	17807,24	212,72	17135,13	111,02	191,61
ambrosin 1uM	15252,09	182,20	17237,83	111,68	163,14
ambrosin 5uM	16114,41	192,50	17086,31	110,70	173,89

Graphic

pIκβα JIMT-1	test 1	test 2	test 3	test 4	Mean	SD	SEM
control	100,00	100,00	100,00	100,00	100,00	0,00	0,00
damsin 1uM	109,49	136,94	95,62	134,65	119,18	20,03	10,02
damsin 5uM	117,82	167,49	92,75	191,61	142,42	45,17	22,58
ambrosin 1uM	132,26	144,86	89,86	163,14	132,53	31,14	15,57
ambrosin 5uM	123,73	123,60	89,47	173,89	127,67	34,78	17,39

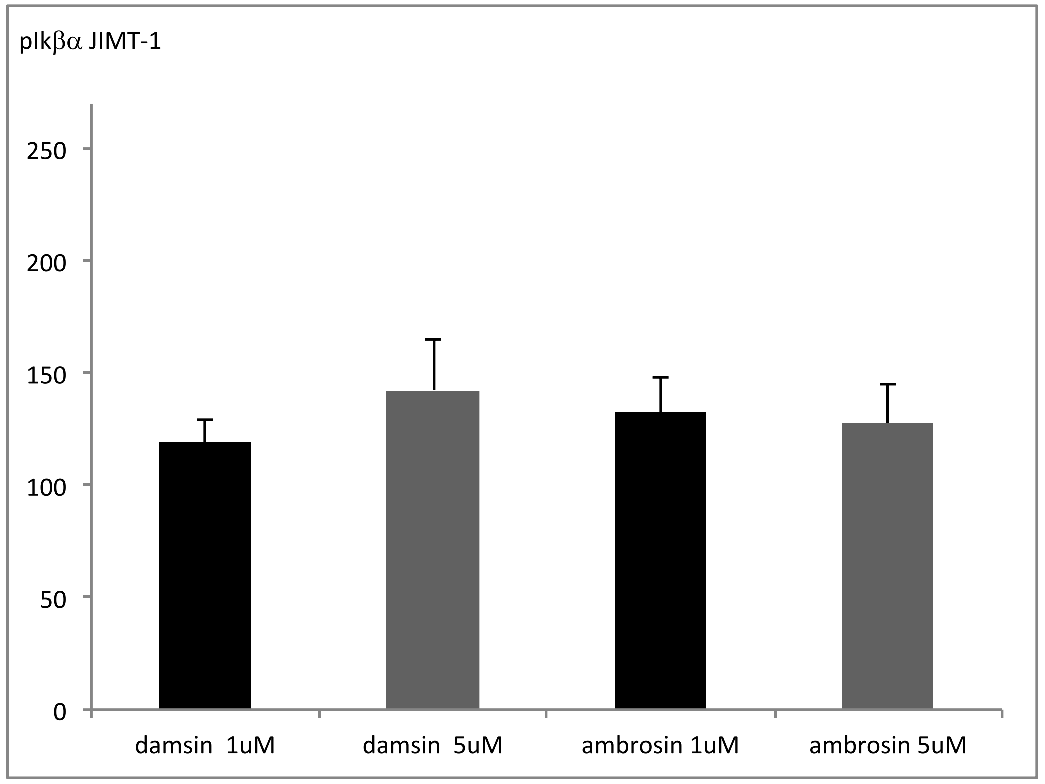
pIkβα JIMT-1
250
200
150
100
50
0
damsin 1uM
damsin 5uM
ambrosin 1uM
ambrosin 5uM
