# Supplementary material for: Anti-cancer stem cell activity of a sesquiterpene lactone isolated from Ambrosia arborescens and of a synthetic derivative
Source: PLoS One. 2017 Sep 1;12(9):e0184304. doi: 10.1371/journal.pone.0184304 (PMC5581169; doi:10.1371/journal.pone.0184304)

sample (MCF-10a 2,5uM)	blue		
Rep 1-2015	total cells	micronuclei	MN%
control 1-40X	87	1	1,15
control 2-40X	29	0	0,00
control 3-40X	46	0	0,00
control 4-40X	34	0	0,00
control 5-40X	74	1	1,35
damsin 1-40X	511	3	0,59
damsin 2-40X	522	3	0,57
damsin 3-40X	482	1	0,21
damsin 4-40X	425	2	0,47
damsin 5-40X	413	0	0,00
ambrosin 1-40X	477	0	0,00
ambrosin 2-40X	379	0	0,00
ambrosin 3-40X	138	0	0,00
ambrosin 4-40X	114	3	2,63
ambrosin 5-40X	223	3	1,35

sample (MCF-10a 5uM)	blue		
Rep 1-2015	total cells	micronuclei	MN%
control 1-40X	87	1	1,15
control 2-40X	29	0	0,00
control 3-40X	46	0	0,00
control 4-40X	34	0	0,00
control 5-40X	74	1	1,35
damsin 1-40X	51	1	1,96
damsin 2-40X	167	1	0,60
damsin 3-40X	103	2	1,94
damsin 4-40X	229	0	0,00
damsin 5-40X	276	0	0,00
ambrosin 1-40X	4	0	0,00
ambrosin 2-40X	3	1	33,33
ambrosin 3-40X	21	2	9,52
ambrosin 4-40X	62	1	1,61
ambrosin 5-40X	43	1	2,33

sample (MCF-10a 2,5uM)	blue		
Rep 2-2015	total cells	micronuclei	MN%
control 1-40X	25	0	0,00
control 2-40X	40	1	2,50
control 3-40X	28	0	0,00
control 4-40X	45	0	0,00
control 5-40X	63	0	0,00
damsin 1-40X	47	1	2,13
damsin 2-40X	77	2	2,60
damsin 3-40X	39	3	7,69
damsin 4-40X	29	2	6,90
damsin 5-40X	43	1	2,33
ambrosin 1-40X	37	1	2,70
ambrosin 2-40X	122	11	9,02
ambrosin 3-40X	47	15	31,91
ambrosin 4-40X	50	5	10,00
ambrosin 5-40X	87	14	16,09

sample (MCF-10a 5uM)	blue		
Rep 2-2015	total cells	micronuclei	MN%
control 1-40X	25	0	0,00
control 2-40X	40	1	2,50
control 3-40X	28	0	0,00
control 4-40X	45	0	0,00
control 5-40X	63	0	0,00
ambrosin 1-40X	3	0	0,00
ambrosin 2-40X	3	0	0,00

sample (MCF-10a 2,5uM)	blue		
Rep 3-2015	total cells	micronuclei	MN%
damsin 1-40X	188	2	1,06
damsin 2-40X	321	1	0,31
damsin 3-40X	107	3	2,80
damsin 4-40X	256	1	0,39
damsin 5-40X	221	4	1,81
ambrosin 1-40X	8	0	0,00
ambrosin 2-40X	10	2	20,00
ambrosin 3-40X	22	2	9,09
ambrosin 4-40X	18	8	44,44
ambrosin 5-40X	13	1	7,69

sample (MCF-10a 5uM)	blue		
Rep 3-2015	total cells	micronuclei	MN%
damsin 1-40X	350	2	0,57
damsin 2-40X	352	3	0,85
damsin 3-40X	94	1	1,06
damsin 4-40X	164	1	0,61
damsin 5-40X	151	1	0,66
ambrosin 1-40X	2	1	50,00
ambrosin 2-40X	4	1	25,00
ambrosin 3-40X	7	1	14,29
ambrosin 4-40X	5	2	40,00
ambrosin 5-40X	2	1	50,00

sample (MCF-10a 2,5uM)	blue		
Rep 4-2015	total cells	micronuclei	MN%
damsin 1-40X	279	1	0,36
damsin 2-40X	64	0	0,00
damsin 3-40X	277	2	0,72
damsin 4-40X	176	5	2,84
damsin 5-40X	216	0	0,00
ambrosin 1-40X	12	2	16,67
ambrosin 2-40X	18	5	27,78
ambrosin 3-40X	14	1	7,14
ambrosin 4-40X	19	4	21,05
ambrosin 5-40X	7	3	42,86

sample (MCF-10a 5uM)	blue		
Rep 4-2015	total cells	micronuclei	MN%
damsin 1-40X	52	0	0,00
damsin 2-40X	63	2	3,17
damsin 3-40X	116	0	0,00
damsin 4-40X	216	2	0,93
damsin 5-40X	131	0	0,00
ambrosin 1-40X	5	1	20,00
ambrosin 2-40X	2	0	0,00
ambrosin 3-40X	2	1	50,00
ambrosin 4-40X	1	0	0,00
ambrosin 5-40X	4	2	50,00

MCF-10a 2,5uM

treatment	rep1	rep2	rep3	rep4	mean	sd		
control 1-40X	1,15	0,00			0,57	0,813		
control 2-40X	0,00	2,50			1,25	1,768		
control 3-40X	0,00	0,00			0,00	0,000		
control 4-40X	0,00	0,00			0,00	0,000	0,74	sd
control 5-40X	1,35	0,00			0,68	0,956	0,50	mean
damsin 1-40X	0,59	2,13	1,06	0,36	1,26	0,789		
damsin 2-40X	0,57	2,60	0,31	0,00	1,16	1,251		
damsin 3-40X	0,21	7,69	2,80	0,72	3,57	3,800		
damsin 4-40X	0,47	6,90	0,39	2,84	2,59	3,733	1,05	sd
damsin 5-40X	0,00	2,33	1,81	0,00	1,38	1,221	1,99	mean
ambrosin 1-40X	0,00	2,70	0,00	16,67	0,90	1,560		
ambrosin 2-40X	0,00	9,02	20,00	27,78	9,67	10,016		
ambrosin 3-40X	0,00	31,91	9,09	7,14	13,67	16,443		
ambrosin 4-40X	2,63	10,00	44,44	21,05	19,03	22,320	6,71	sd
ambrosin 5-40X	1,35	16,09	7,69	42,86	8,38	7,397	10,33	mean

MCF-10a 5uM

treatment	rep1	rep2	rep3	rep4	mean	sd		
control 1-40X	1,15	0,00			0,57	0,813		
control 2-40X	0,00	2,50			1,25	1,768		
control 3-40X	0,00	0,00			0,00	0,000		
control 4-40X	0,00	0,00			0,00	0,000	0,74	sd
control 5-40X	1,35	0,00			0,68	0,956	0,50	mean
damsin 1-40X	1,96		0,57	0,00	0,84	1,008		
damsin 2-40X	0,60		0,85	3,17	1,54	1,420		
damsin 3-40X	1,94		1,06	0,00	1,00	0,972		
damsin 4-40X	0,00		0,61	0,93	0,51	0,471	0,50	sd
damsin 5-40X	0,00		0,66	0,00	0,22	0,382	0,82	mean
ambrosin 1-40X	0,00	0,00	50,00	20,00	17,50	23,629		
ambrosin 2-40X	33,33	0,00	25,00	0,00	14,58	17,180		
ambrosin 3-40X	9,52		14,29	50,00	24,60	0,382		
ambrosin 4-40X	1,61		40,00	0,00	13,87	0,382	8,50	sd
ambrosin 5-40X	2,33		50,00	50,00	34,11	0,382	20,93	mean

	mean	sd	sem
control	0,50	0,74	0,23441
damsin 2.5	1,99	1,05	0,47112
damsin 5	0,82	0,5	0,22458
ambrosin 2,5	10,33	6,71	2,99927
ambrosin 5	20,93	8,5	3,80158

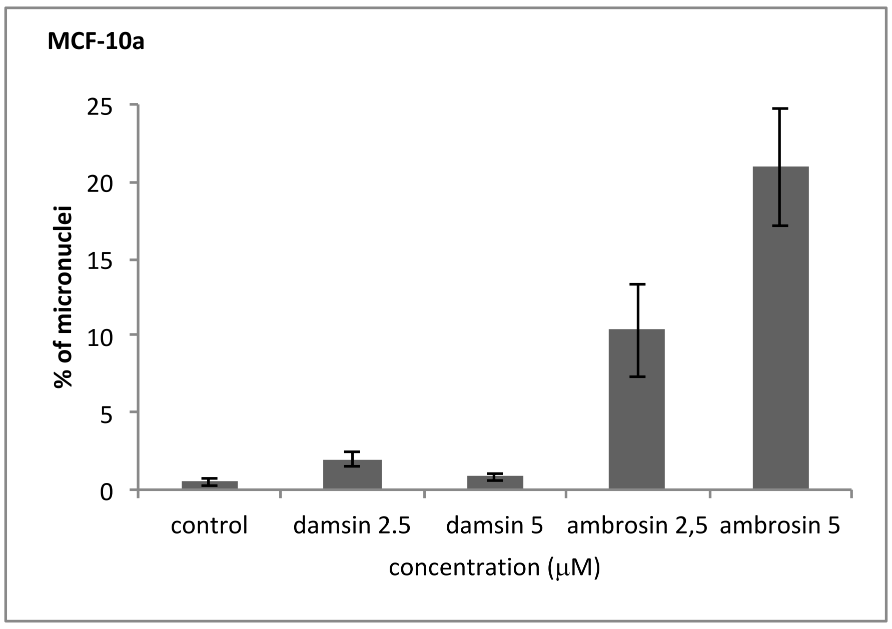

sample (MCF-7 2,5uM)	blue		
Rep 1-2015	total cells	micronuclei	MN%
control 1-40X	29	1	3,45
control 2-40X	58	1	1,72
control 3-40X	63	4	6,35
control 4-40X	79	3	3,80
control 5-40X	69	5	7,25
damsin 1-40X	32	2	6,25
damsin 2-40X	142	4	2,82
damsin 3-40X	128	0	0,00
damsin 4-40X	16	0	0,00
damsin 5-40X	33	3	9,09
ambrosin 1-40X	27	2	7,41
ambrosin 2-40X	21	4	19,05
ambrosin 3-40X	5	2	40,00
ambrosin 4-40X	26	1	3,85
ambrosin 5-40X	34	4	11,76

sample (MCF-7 5uM)	blue		
Rep 1-2015	total cells	micronuclei	MN%
control 1-40X	29	1	3,45
control 2-40X	58	1	1,72
control 3-40X	63	4	6,35
control 4-40X	79	3	3,80
control 5-40X	69	5	7,25
damsin 1-40X	2	1	50,00
damsin 2-40X	3	0	0,00
damsin 3-40X	9	1	11,11
damsin 4-40X	3	0	0,00
damsin 5-40X	2	0	0,00
ambrosin 1-40X	4	0	0,00
ambrosin 2-40X	56	5	8,93
ambrosin 3-40X	5	2	40,00
ambrosin 4-40X	11	2	18,18
ambrosin 5-40X	8	0	0,00

sample (MCF-7 2,5uM)	blue		
Rep 2-2015	total cells	micronuclei	MN%
control 1-40X	171	0	0,00
control 2-40X	99	1	1,01
control 3-40X	59	0	0,00
control 4-40X	71	2	2,82
control 5-40X	77	0	0,00
damsin 1-40X	4	1	25,00
damsin 2-40X	7	1	14,29
damsin 3-40X	16	2	12,50
damsin 4-40X	11	1	9,09
damsin 5-40X	4	0	0,00
ambrosin 1-40X	30	12	40,00
ambrosin 2-40X	41	19	46,34
ambrosin 3-40X	28	13	46,43
ambrosin 4-40X	16	2	12,50
ambrosin 5-40X	36	11	30,56

sample (MCF-7 5uM)	blue		
Rep 2-2015	total cells	micronuclei	MN%
control 1-40X	171	0	0,00
control 2-40X	99	1	1,01
control 3-40X	59	0	0,00
control 4-40X	71	2	2,82
control 5-40X	77	0	0,00
damsin 1-40X	4	2	50,00
damsin 2-40X	25	1	4,00
damsin 3-40X	22	3	13,64
damsin 4-40X	16	2	12,50
damsin 5-40X	10	0	0,00
ambrosin 1-40X	3	2	66,67
ambrosin 2-40X	2	0	0,00
ambrosin 3-40X	3	0	0,00
ambrosin 4-40X	3	2	66,67
ambrosin 5-40X	4	2	50,00

sample (MCF-7 2,5uM)	blue		
Rep 3-2015	total cells	micronuclei	MN%
damsin 1-40X	47	5	10,64
damsin 2-40X	54	4	7,41
damsin 3-40X	80	4	5,00
damsin 4-40X	19	2	10,53
damsin 5-40X	33	0	0,00
ambrosin 1-40X	21	8	38,10
ambrosin 2-40X	30	8	26,67
ambrosin 3-40X	36	19	52,78
ambrosin 4-40X	20	12	60,00
ambrosin 5-40X	39	10	25,64

sample (MCF-7 5uM)	blue		
Rep 3-2015	total cells	micronuclei	MN%
damsin 1-40X	127	3	2,36
damsin 2-40X	19	3	15,79
damsin 3-40X	2	0	0,00
damsin 4-40X	128	8	6,25
damsin 5-40X	8	2	25,00
ambrosin 1-40X	3	0	0,00
ambrosin 2-40X	3	0	0,00
ambrosin 3-40X	3	2	66,67
ambrosin 4-40X	6	4	66,67
ambrosin 5-40X	1	0	0,00

sample (MCF-7 2,5uM)	blue		
Rep 4-2015	total cells	micronuclei	MN%
damsin 1-40X	18	2	11,11
damsin 2-40X	10	3	30,00
damsin 3-40X	23	1	4,35
damsin 4-40X	79	1	1,27
damsin 5-40X	21	0	0,00
ambrosin 1-40X	9	5	55,56
ambrosin 2-40X	16	3	18,75
ambrosin 3-40X	27	2	7,41
ambrosin 4-40X	6	4	66,67
ambrosin 5-40X	13	10	76,92
ambrosin 6-40X	10	2	20,00

sample (MCF-7 5uM)	blue		
Rep 4-2015	total cells	micronuclei	MN%
ambrosin 1-40X	5	2	40,00
ambrosin 2-40X	8	1	12,50
ambrosin 3-40X	3	1	33,33
ambrosin 4-40X	2	0	0,00
ambrosin 5-40X	13	2	15,38

MCF-7 2,5uM

treatment	rep1	rep2	rep3	rep4	mean	sd		
control 1-40X	3,45	0,00			1,72	2,438		
control 2-40X	1,72	1,01			1,37	0,505		
control 3-40X	6,35	0,00			3,17	4,490		
control 4-40X	3,80	2,82			3,31	0,693	2,12	sd
control 5-40X	7,25	0,00			3,62	5,124	2,64	mean
damsin 1-40X	6,25	25,00	10,64	11,11	13,25	8,133		
damsin 2-40X	2,82	14,29	7,41	30,00	13,63	11,889		
damsin 3-40X	0,00	12,50	5,00	4,35	5,46	5,190		
damsin 4-40X	0,00	9,09	10,53	1,27	5,22	5,355	5,15	sd
damsin 5-40X	9,09	0,00	0,00	0,00	2,27	4,545	7,97	mean
ambrosin 1-40X	7,41	40,00	38,10	55,56	35,26	20,151		
ambrosin 2-40X	19,05	46,34	26,67	18,75	27,70	12,956		
ambrosin 3-40X	40,00	46,43	52,78	7,41	36,65	20,183		
ambrosin 4-40X	3,85	12,50	60,00	66,67	35,75	32,158	3,74	sd
ambrosin 5-40X	11,76	30,56	25,64	76,92	36,22	28,277	34,32	mean

MCF-7 5uM

treatment	rep1	rep2	rep3	rep4	mean	sd		
control 1-40X	3,45	0,00			1,72	2,438		
control 2-40X	1,72	1,01			1,37	0,505		
control 3-40X	6,35	0,00			3,17	4,490		
control 4-40X	3,80	2,82			3,31	0,693	2,12	sd
control 5-40X	7,25	0,00			3,62	5,124	2,64	mean
damsin 1-40X	50,00	50,00	2,36		34,12	27,504		
damsin 2-40X	0,00	4,00	15,79		6,60	8,209		
damsin 3-40X	11,11	13,64	0,00		8,25	7,255		
damsin 4-40X	0,00	12,50	6,25		6,25	6,250	12,01	sd
damsin 5-40X	0,00	0,00	25,00		8,33	14,434	12,71	mean
ambrosin 1-40X	0,00	66,67	0,00	40,00	26,67	32,660		
ambrosin 2-40X	8,93	0,00	0,00	12,50	5,36	6,355		
ambrosin 3-40X	40,00	0,00	66,67	33,33	35,00	27,420		
ambrosin 4-40X	18,18	66,67	66,67	0,00	37,88	34,060	13,48	sd
ambrosin 5-40X	0,00	50,00	0,00	15,38	16,35	23,579	24,25	mean

	mean	sd	sem
control	2,64	2,12	0,45588491
damsin 2.5	7,97	5,15	2,30425325
damsin 5	12,71	12,01	5,36926661
ambrosin 2,5	34,32	3,74	1,67048948
ambrosin 5	23,25	13,48	6,02726087

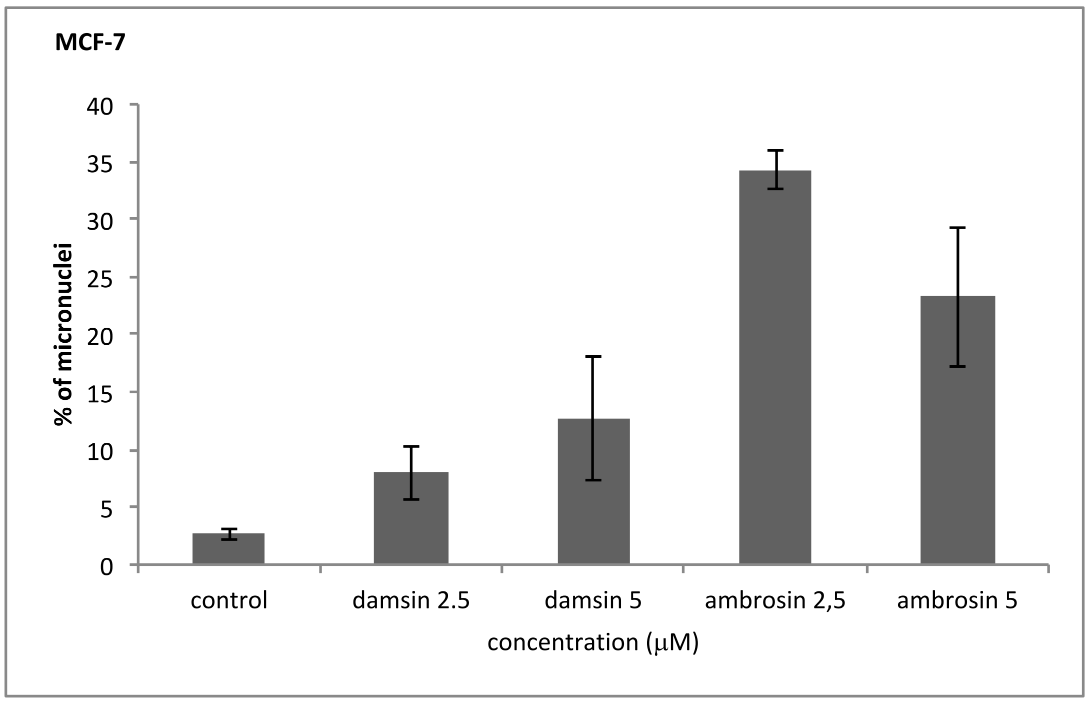

sample (JIMT-1, 5uM)	blue		
Rep 1-2015	total cells	micronuclei	MN%
control 1-40X	103	2	1,94
control 2-40X	153	1	0,65
control 3-40X	482	2	0,41
control 4-40X	68	3	4,41
control 5-40X	90	0	0,00
damsin 1-40X	54	3	5,56
damsin 2-40X	75	5	6,67
damsin 3-40X	71	3	4,23
damsin 4-40X	28	1	3,57
damsin 5-40X	34	1	2,94
ambrosin 1-40X	16	1	6,25
ambrosin 2-40X	7	0	0,00
ambrosin 3-40X	4	1	25,00
ambrosin 4-40X	5	1	20,00
ambrosin 5-40X	11	1	9,09

sample (JIMT-1,	blue		
Rep 1-2015	total cells	micronuclei	MN%
control 1-40X	103	2	1,94
control 2-40X	153	1	0,65
control 3-40X	482	2	0,41
control 4-40X	68	3	4,41
control 5-40X	90	0	0,00
damsin 1-40X	106	3	2,83
damsin 2-40X	105	2	1,90
damsin 3-40X	60	1	1,67
damsin 4-40X	45	2	4,44
damsin 5-40X	64	3	4,69
ambrosin 1-40X	14	2	14,29
ambrosin 2-40X	18	2	11,11
ambrosin 3-40X	10	1	10,00
ambrosin 4-40X	12	2	16,67
ambrosin 5-40X	13	3	23,08

sample (JIMT-1, 5uM)	blue		
Rep 2-2015	total cells	micronuclei	MN%
control 1-40X	255	5	1,96
control 2-40X	129	4	3,10
control 3-40X	514	4	0,78
control 4-40X	113	1	0,88
control 5-40X	146	7	4,79
damsin 1-40X	48	0	0,00
damsin 2-40X	24	3	12,50
damsin 3-40X	35	3	8,57
damsin 4-40X	48	2	4,17
damsin 5-40X	41	4	9,76
ambrosin 1-40X	1	1	100,00
ambrosin 2-40X	1	1	100,00
ambrosin 3-40X	6	1	16,67
ambrosin 4-40X	19	4	21,05
ambrosin 5-40X	23	1	4,35

sample (JIMT-1,	blue		
Rep 2-2015	total cells	micronuclei	MN%
control 1-40X	255	5	1,96
control 2-40X	129	4	3,10
control 3-40X	514	4	0,78
control 4-40X	113	1	0,88
control 5-40X	146	7	4,79
damsin 1-40X	141	6	4,26
damsin 2-40X	115	9	7,83
damsin 3-40X	166	16	9,64
damsin 4-40X	133	13	9,77
damsin 5-40X	93	9	9,68
ambrosin 1-40X	16	2	12,50
ambrosin 2-40X	10	1	10,00
ambrosin 3-40X	5	0	0,00
ambrosin 4-40X	8	4	50,00
ambrosin 5-40X	14	0	0,00

sample (JIMT-1, 5uM)	blue		
Rep 3-2015	total cells	micronuclei	MN%
damsin 1-40X	124	3	2,42
damsin 2-40X	134	3	2,24
damsin 3-40X	58	3	5,17
damsin 4-40X	38	4	10,53
damsin 5-40X	68	4	5,88
ambrosin 1-40X	6	0	0,00
ambrosin 2-40X	7	1	14,29
ambrosin 3-40X	8	1	12,50
ambrosin 4-40X	8	1	12,50
ambrosin 5-40X	13	3	23,08

sample (JIMT-1,	blue		
Rep 3-2015	total cells	micronuclei	MN%
damsin 1-40X	72	1	1,39
damsin 2-40X	150	4	2,67
damsin 3-40X	189	4	2,12
damsin 4-40X	123	4	3,25
damsin 5-40X	77	1	1,30
ambrosin 1-40X	133	5	3,76
ambrosin 2-40X	133	5	3,76
ambrosin 3-40X	181	6	3,31
ambrosin 4-40X	119	15	12,61
ambrosin 5-40X	107	11	10,28

sample (JIMT-1, 5uM)	blue		
Rep 4-2015	total cells	micronuclei	MN%
damsin 1-40X	21	1	4,76
damsin 2-40X	39	2	5,13
damsin 3-40X	31	1	3,23
damsin 4-40X	61	1	1,64
damsin 5-40X	54	2	3,70
ambrosin 1-40X	4	1	25,00
ambrosin 2-40X	9	2	22,22
ambrosin 3-40X	9	0	0,00
ambrosin 4-40X	4	1	25,00
ambrosin 5-40X	8	2	25,00

sample (JIMT-1,	blue		
Rep 4-2015	total cells	micronuclei	MN%
damsin 1-40X	224	17	7,59
damsin 2-40X	173	7	4,05
damsin 3-40X	67	4	5,97
damsin 4-40X	97	6	6,19
damsin 5-40X	166	5	3,01
ambrosin 1-40X	211	3	1,42
ambrosin 2-40X	181	2	1,10
ambrosin 3-40X	216	5	2,31
ambrosin 4-40X	221	9	4,07
ambrosin 5-40X	196	1	0,51

JIMT-1 2,5uM

treatment	rep1	rep2	rep3	rep4	mean	sd	
control 1-40X	1,94	1,96			1,95	0,013	
control 2-40X	0,65	3,10			1,88	1,730	
control 3-40X	0,41	0,78			0,60	0,257	
control 4-40X	4,41	0,88			2,65	2,494	1,44
control 5-40X	0,00	4,79			2,40	3,390	1,89
damsin 1-40X	2,83	4,26	1,39	7,59	4,02	2,654	
damsin 2-40X	1,90	7,83	2,67	4,05	4,11	2,631	
damsin 3-40X	1,67	9,64	2,12	5,97	4,85	3,732	
damsin 4-40X	4,44	9,77	3,25	6,19	5,91	2,842	0,76
damsin 5-40X	4,69	9,68	1,30	3,01	4,67	3,614	4,71
ambrosin 1-40X	14,29	12,50	3,76	1,42	7,99	6,351	
ambrosin 2-40X	11,11	10,00	3,76	1,10	6,49	4,835	
ambrosin 3-40X	10,00	0,00	3,31	2,31	3,91	4,292	
ambrosin 4-40X	16,67	50,00	12,61	4,07	20,84	20,139	6,56
ambrosin 5-40X	23,08	0,00	10,28	0,51	8,47	10,828	9,54

JIMT-1 5uM

treatment	rep1	rep2	rep3	rep4	mean	sd	
control 1-40X	1,94	1,96			1,95	0,013	
control 2-40X	0,65	3,10			1,88	1,730	
control 3-40X	0,41	0,78			0,60	0,257	
control 4-40X	4,41	0,88			2,65	2,494	1,44
control 5-40X	0,00	4,79			2,40	3,390	1,89
damsin 1-40X	5,56	0,00	2,42	4,76	3,18	2,506	
damsin 2-40X	6,67	12,50	2,24	5,13	6,63	4,320	
damsin 3-40X	4,23	8,57	5,17	3,23	5,30	2,322	
damsin 4-40X	3,57	4,17	10,53	1,64	4,98	3,854	1,25
damsin 5-40X	2,94	9,76	5,88	3,70	5,57	3,056	5,13
ambrosin 1-40X	6,25	100,00	0,00	25,00	32,81	46,034	
ambrosin 2-40X	0,00	100,00	14,29	22,22	34,13	44,868	
ambrosin 3-40X	25,00	16,67	12,50	0,00	13,54	10,417	
ambrosin 4-40X	20,00	21,05	12,50	25,00	19,64	5,223	9,73
ambrosin 5-40X	9,09	4,35	23,08	25,00	15,38	10,215	23,10

	mean	sd	sem
control	1,89	1,44	0,3542038
damsin 2.5	4,71	0,76	0,33994326
damsin 5	5,13	1,25	0,56093241
ambrosin 2,5	9,54	6,56	2,93388543
ambrosin 5	23,1	9,73	4,35251813

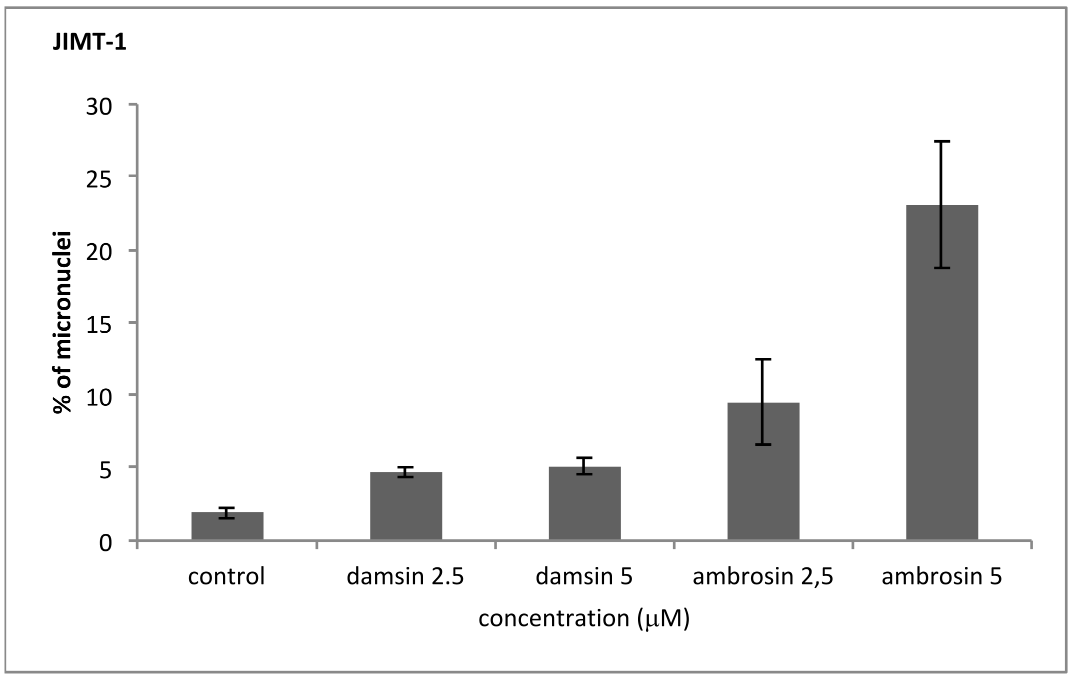

Supplement: S6 Fig — (PDF) [file pone.0184304.s006.pdf]
