# Supplementary material for: Anti-cancer stem cell activity of a sesquiterpene lactone isolated from Ambrosia arborescens and of a synthetic derivative
Source: PLoS One. 2017 Sep 1;12(9):e0184304. doi: 10.1371/journal.pone.0184304 (PMC5581169; doi:10.1371/journal.pone.0184304)

Original flow cytometry data for evaluation of 1 μM damsin on the CD44+/CD24⁻ population

Experimental date 140616

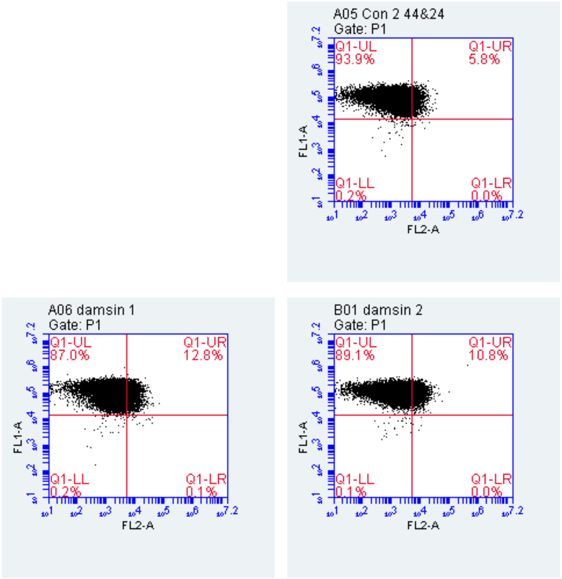

Experimental date 140623

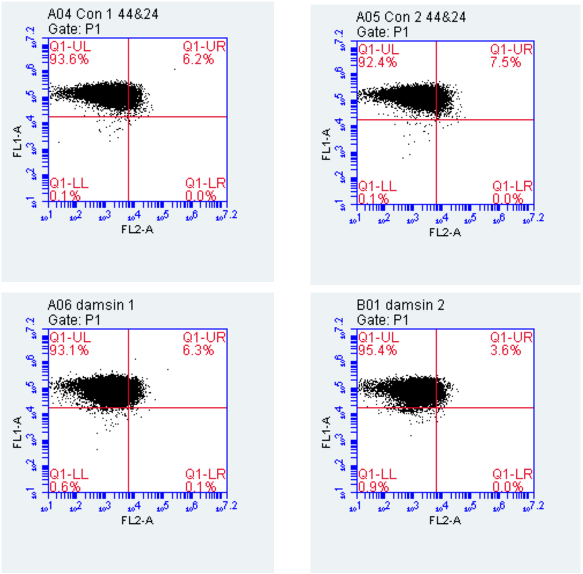

Experimental date 150202

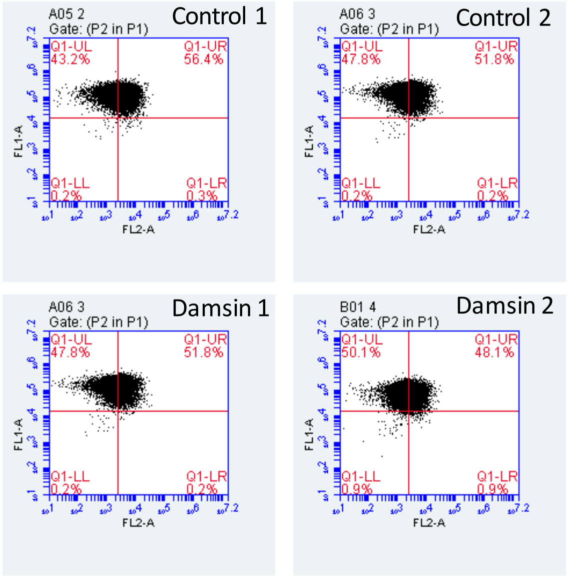

Each figure is from one independently treated cell culture.

Original flow cytometry data for evaluation of 5 μM damsin on the CD44+/CD24[-] population

Experimental date 150828

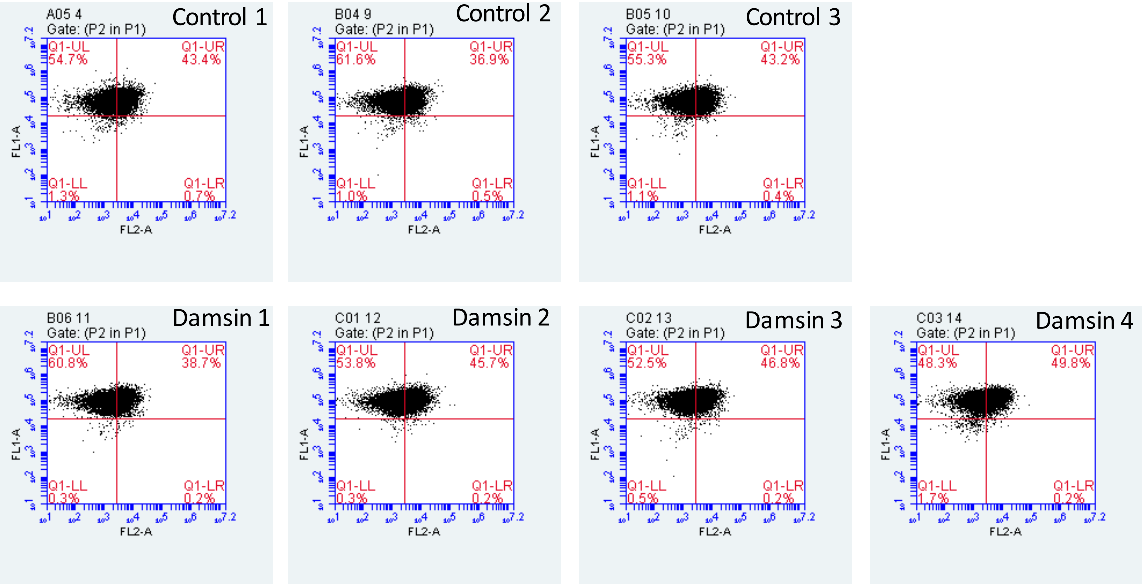

Each figure is from one independently treated cell culture.

Original flow cytometry data for evaluation of 1 μM ambrosin on the CD44+/CD24$^-$ population

Experimental date 140616

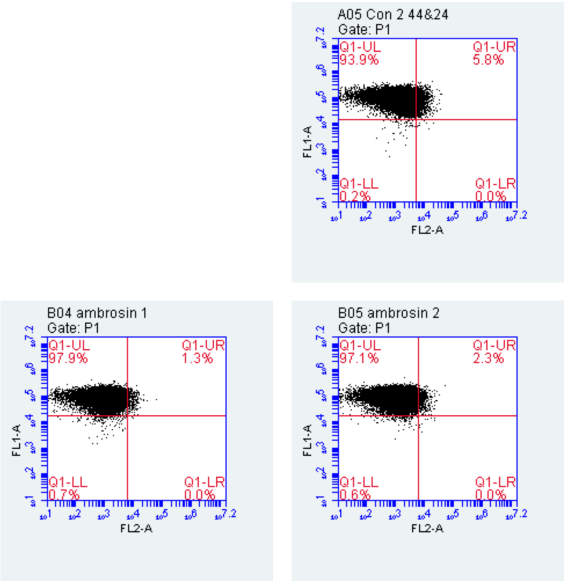

Experimental date 150202

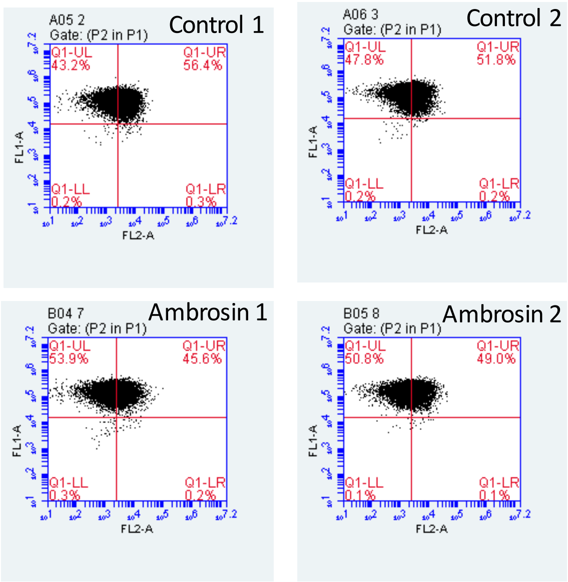

Each figure is from one independently treated cell culture.

Original flow cytometry data for evaluation of 5 μM ambrosin on the CD44+/CD24^{-} population

Experimental date 150828

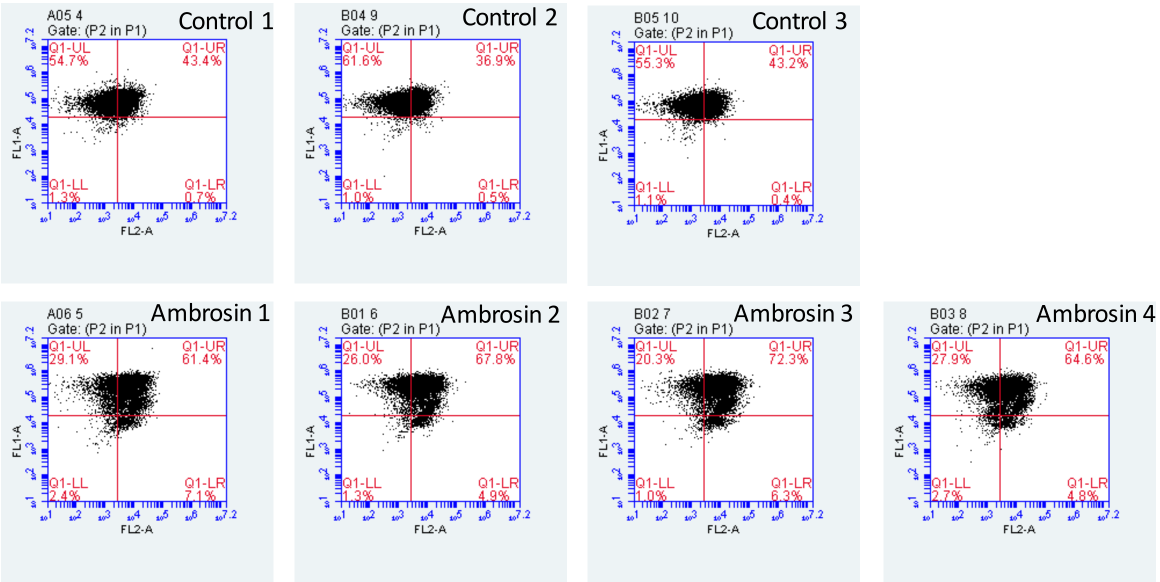

Each figure is from one
independently
treated cell culture.

Original flow cytometry data for evaluation of 1 μM damsin on the ALDH positive population

Experimental date 140704

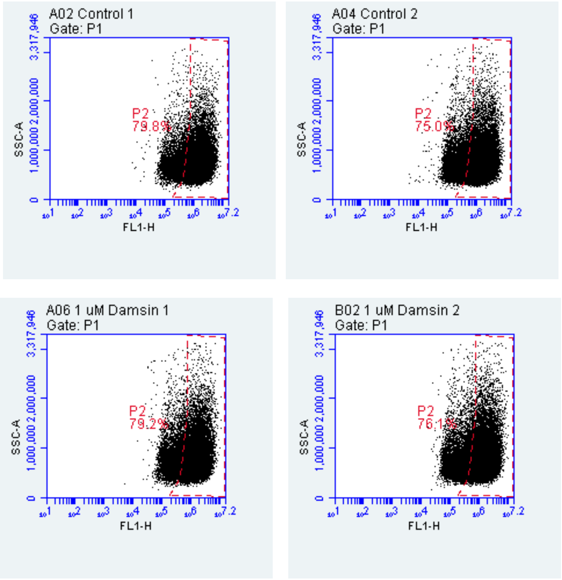

Experimental date 140825

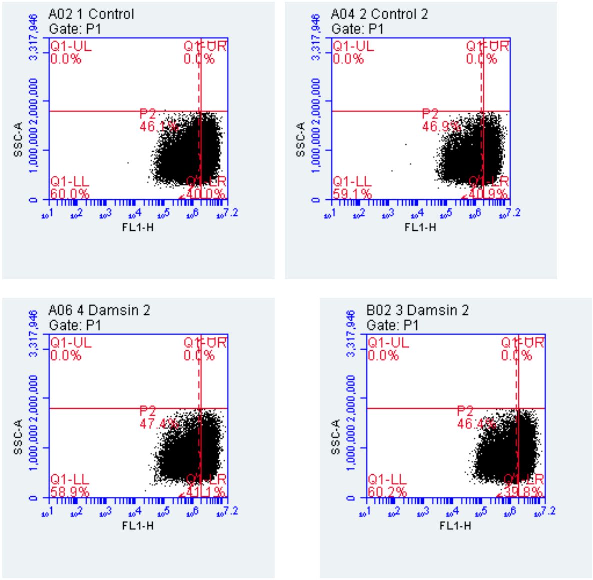

Experimental date 150202

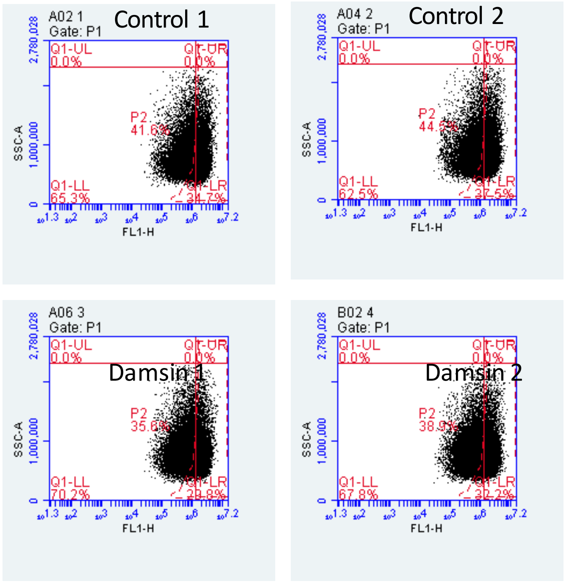

Experimental date 150817

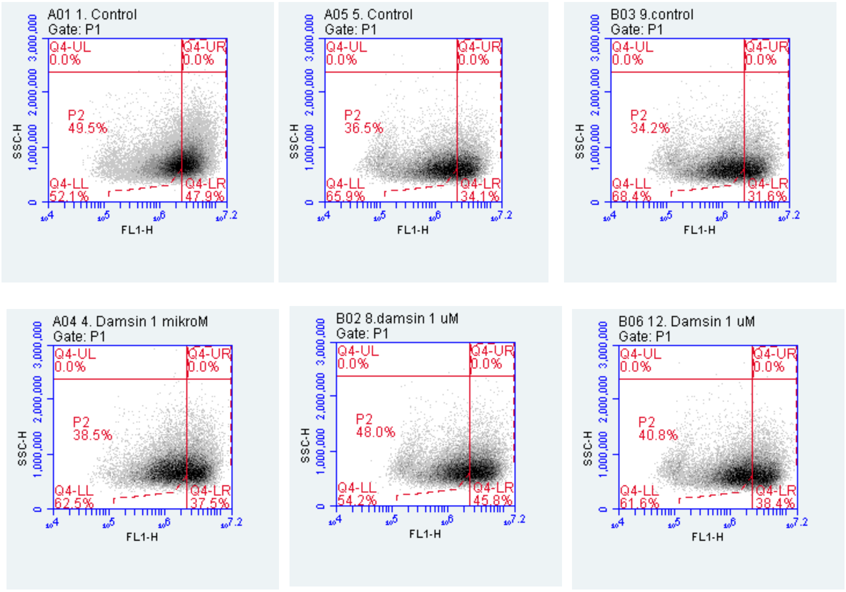

Each figure is from one independently treated cell culture.

Original flow cytometry data for evaluation of 5 μM damsin on the ALDH positive population

Experimental date 150817

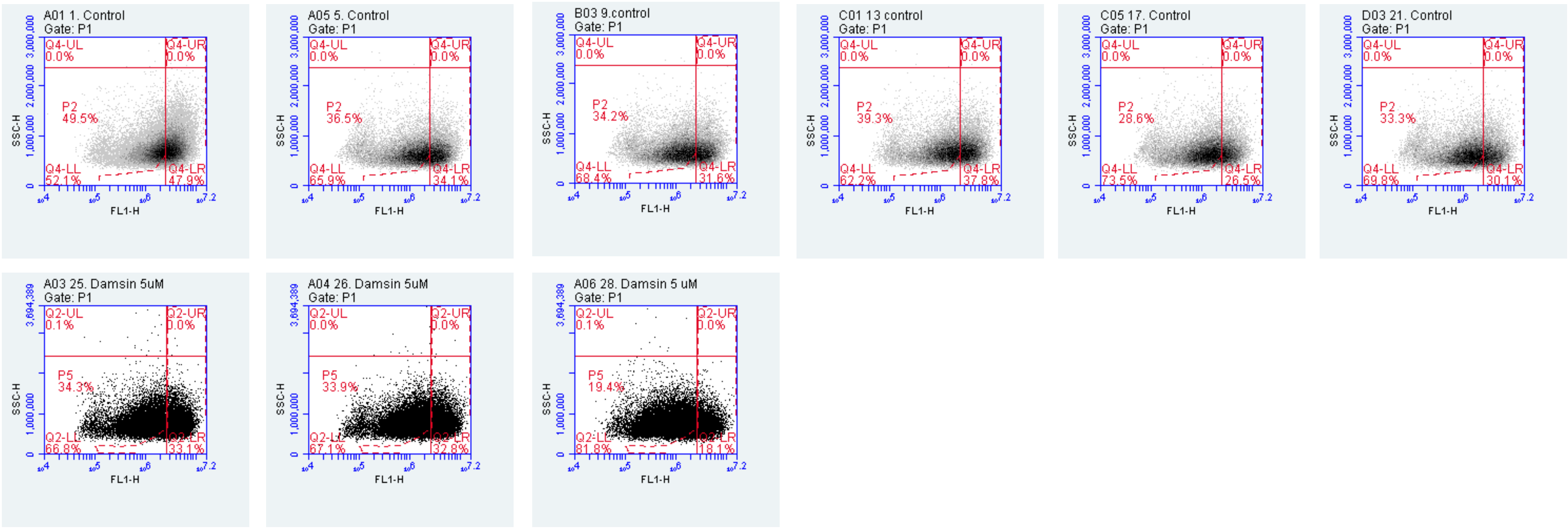

Each figure is from one independently treated cell culture.

Original flow cytometry data for evaluation of 1 μM ambrosin on the ALDH positive population

Experimental date 140704

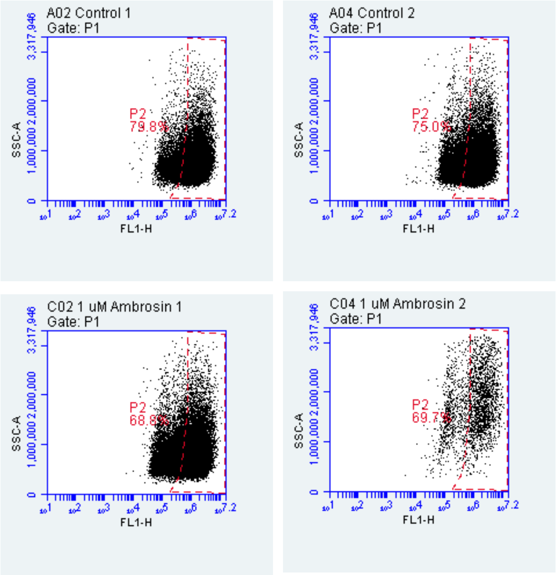

Experimental date 140825

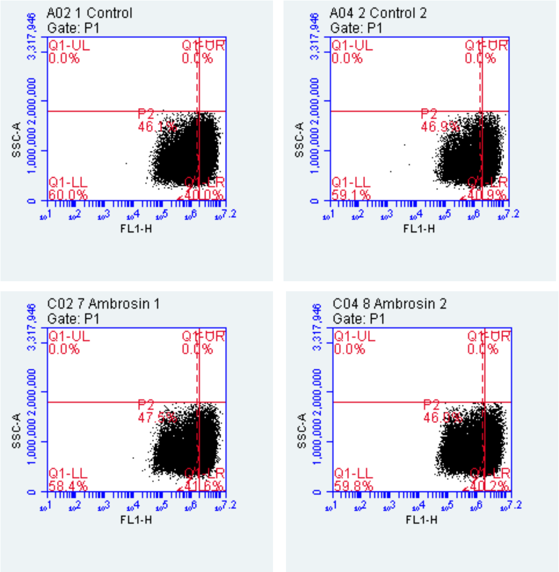

Each figure is from one independently treated cell culture.

Experimental date 150817

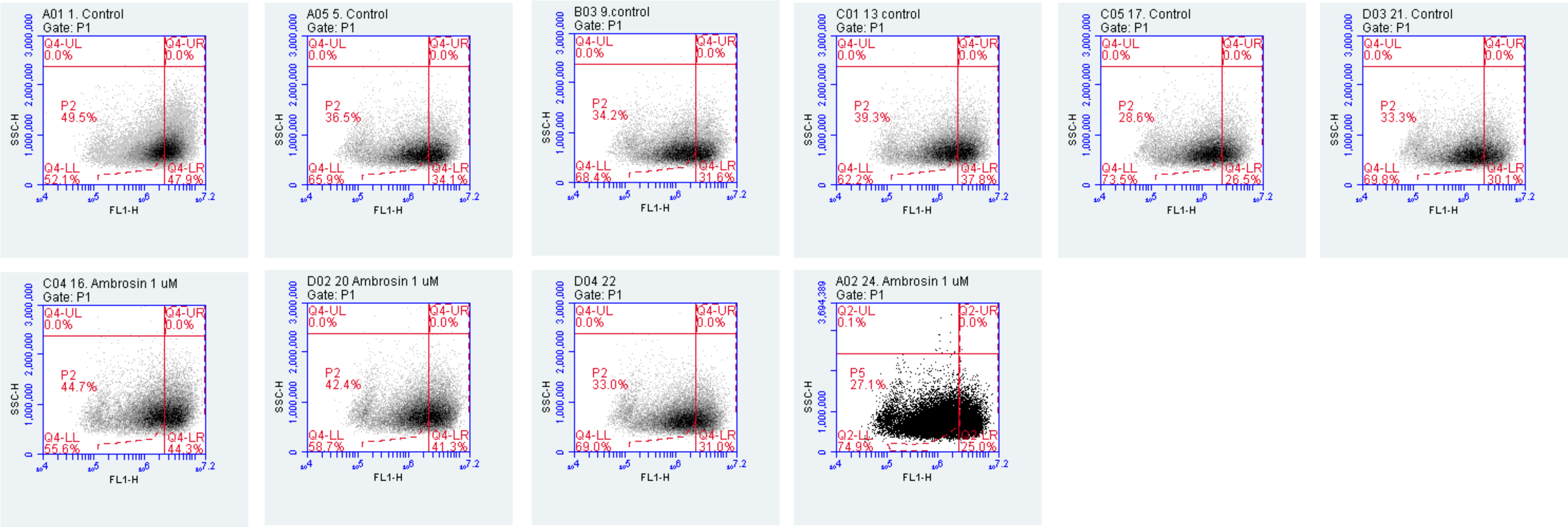

Original flow cytometry data for evaluation of 5 μM ambrosin on the ALDH positive population

Experimental date 140523

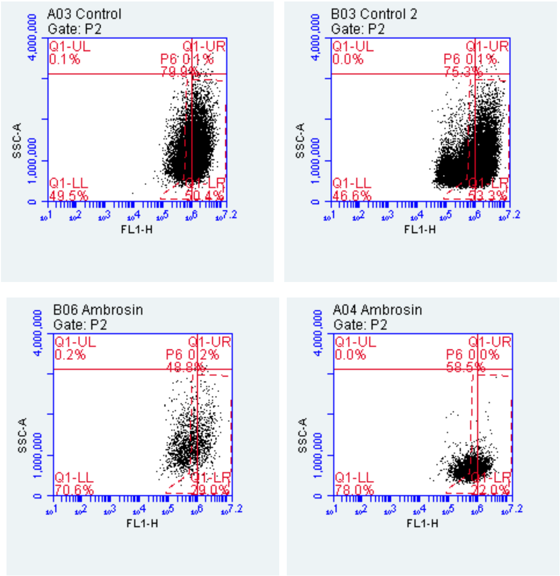

Each figure is from one independently treated cell culture.

Experimental date 150817

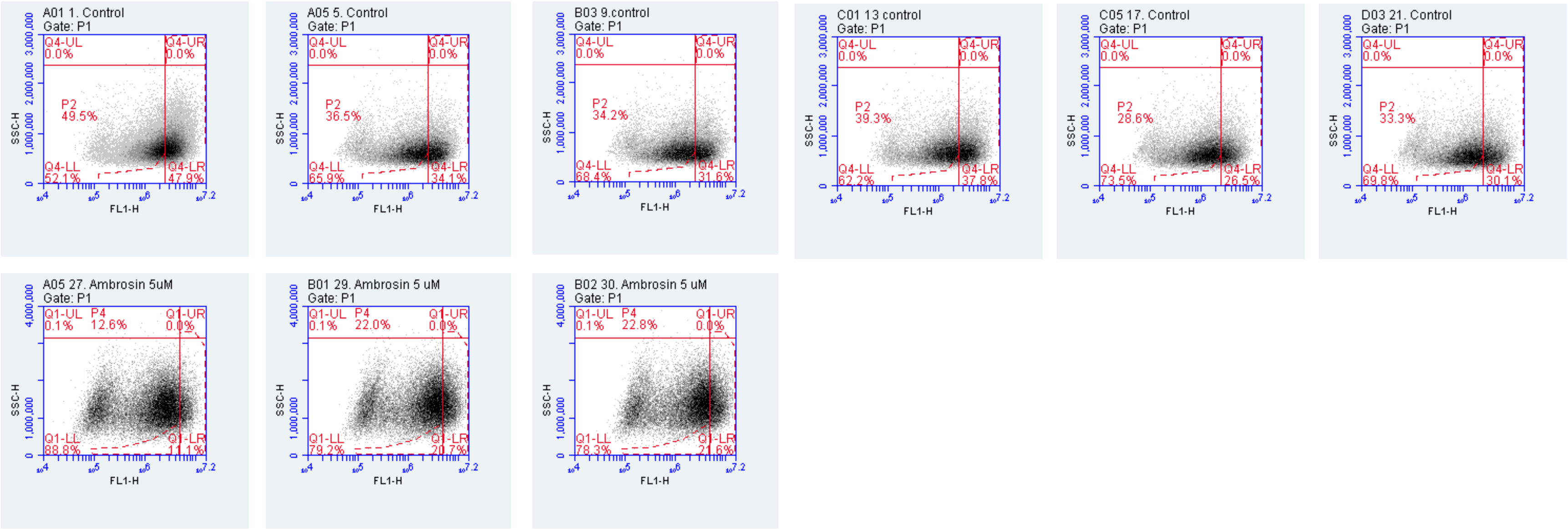

colony formation assay

20140523	5 uM	plate 1	
	control	damsin	ambrosin
	14,25	7,5	3,25
	10,75	9,75	3,25
	12,25	8,25	1
	13	7	0,5
mean	12,5625	8,125	2
in total	398,81	257,94	63,49
in % of seeded	79,76	51,59	12,70
in % of control		64,68	15,92

colony formation assay

20140609	1 uM	plate 1	
	control	damsin	ambrosin
	7,25	7	7,5
	7,25	5,5	7,75
	9	6,75	7
	8,25	7,5	7,5
mean	7,9375	6,6875	7,4375
in total	251,98	212,30	236,11
in % of seeded	50,40	42,46	47,22
in % of control		84,25	93,70

20140523	5 uM	plate 2	
	control	damsin	ambrosin
	14,25	5,75	1,75
	10,75	6	3,75
	12,25	4,25	4
	13	5,5	1,5
mean	12,5625	5,375	2,75
in total	398,81	170,63	87,30
in % of seeded	79,76	34,13	17,46
in % of control		42,79	21,89

	1 uM	plate 1	20150522	
	control	damsin	ambrosin	Damsin
	6,75	10	8,25	10
	9,75	11	9,5	10
	9,75	9,25	8,75	7,25
	10,5	11,25	8,5	5,5
mean	9,1875	10,375	8,75	8,1875
in total	291,67	329,37	277,78	259,92
in % of seeded	58,33	65,87	55,56	51,98
in % of control		112,93	95,24	89,12

20150525	plate 1	5 uM	5 uM	1 mikroM
	control	damsin	ambrosin	ambrosin
	8,25	6	4,75	8,25
	10,5	7	5,25	9,5
	10,25	7,5	4,75	9,25
	7,5	7,25	5,75	8,75
mean	9,125	6,9375	5,125	8,9375
in total	289,68	220,24	162,70	283,73
in % of seeded	57,94	44,05	32,54	56,75
in % of control		76,03	56,16	97,95

	1 uM	plate 1	20150525	
	control	control 2	damsin	ambrosin
	10,75	14,75	13,75	11,75
	10	11	11	12,25
	10,5	12,25	13,5	10,5
	11,25	17,75	11,25	9,25
mean	12,28125		12,375	10,9375
in total	389,88		392,86	347,22
in % of seeded	77,98		78,57	69,44
in % of control			100,76	89,06

5 µM

	damsin	ambrosin
average	61,16	31,33
sd	16,90	21,72

1µM

	damsin	ambrosin
average	96,76	93,99
sd	12,81	3,72

	1 µM	sd	SEM	5 µM	sd	SEM
Damsin	96,76	12,81	7,40	61,16	16,90	9,76
Ambrosin	93,99	3,72	2,15	31,33	21,72	12,54

	MEAN	SEM
D1	96,76	7,4
D5	61,16	9,76
A1	93,98	2,15
A5	31,33	12,54

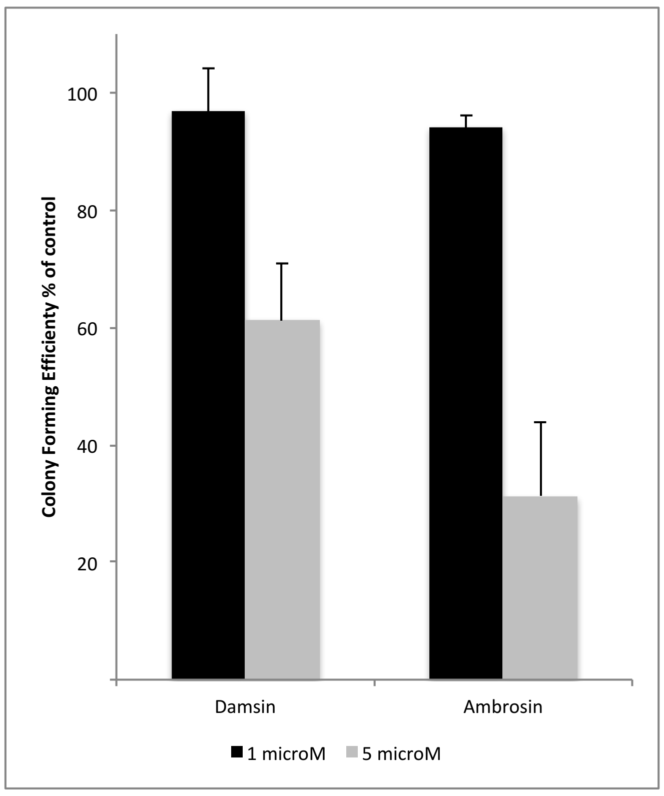

Supplement: S7 Fig — (PDF) [file pone.0184304.s007.pdf]
