# Supplementary material for: Anti-cancer stem cell activity of a sesquiterpene lactone isolated from Ambrosia arborescens and of a synthetic derivative
Source: PLoS One. 2017 Sep 1;12(9):e0184304. doi: 10.1371/journal.pone.0184304 (PMC5581169; doi:10.1371/journal.pone.0184304)
Supplement: S8 Fig — (PDF) [file pone.0184304.s008.pdf]

Wound healing data from 1µM									0	24	48	72
	Assay 1	Assay 1	Assay 3	Assay 3	Assay 4	Assay 5		Mean	SEM			
Damsin												
0 hour	0	0	0	0	0	0		0	0			
24 hour	40,88637	31,19994	40,11618	45,86424	46,43775	35,26298		39,96124	2,426			
48 hour	72,09433	60,20133	69,17694	69,89677	63,13004	40,69626		62,53261	4,734			
72 hour	69,35374	65,55441	88,29959	74,48903	74,08581	61,51453		72,21618	3,805			
Control												
0 hour	0	0	0	0	0	0		0	0,000			
24 hour	46,88675	46,26676	56,59011	42,57118	47,83211	34,028		45,69582	3,005			
48 hour	90,10032	81,09645	86,38797	80,67127	70,85605	48,19848		76,21842	6,201			
72 hour	97,24855	97,98103	96,8393	82,62195	77,35523	89,51643		90,26042	3,546			
Ambrosin												
0 hour	0	0	0	0	0	0		0	0,000			
24 hour	47,64754	44,28358	44,49083	40,14888	43,18545	23,92679		40,61385	3,480			
48 hour	92,30642	80,2168	74,73864	72,41132	66,50082	37,33836		70,58539	7,544			
72 hour	89,80208	85,33031	79,04011	73,02127	76,43019	64,00007		77,93734	3,730			

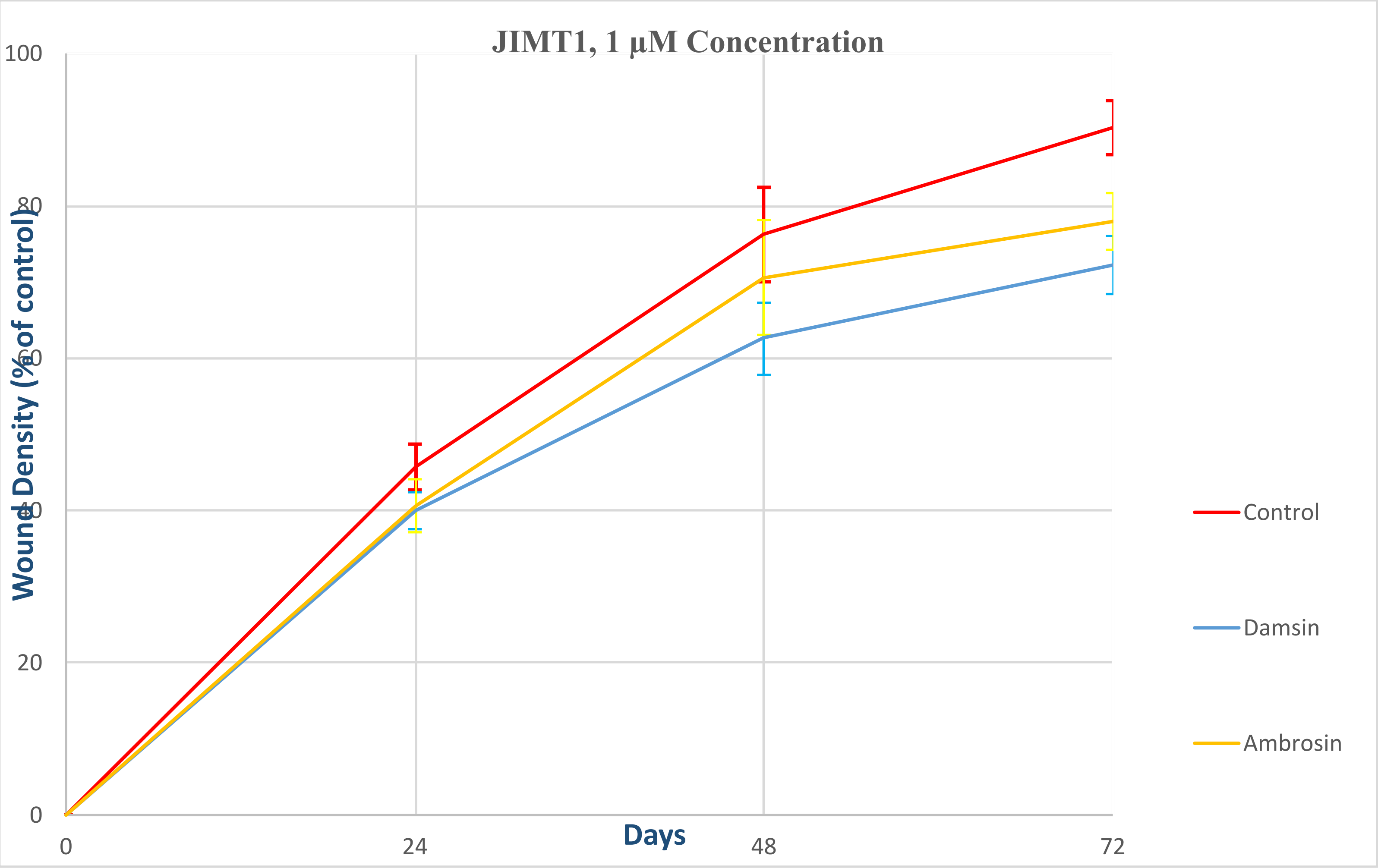

wound healing assay 1 at 1 µM

	Line 1	line 2	line 3	mean	% of mean	
Damsin 1					baz	baste
0 hour	387221	383453	342200	370958	0	0
24 hour	286566	264700	214392	255219,3	68,80006	31,19994
48 hour	169724	181915	91270	147636,3	39,79867	60,20133
72 hour	144907	132495	105934	127778,7	34,44559	65,55441

	Line 1	line 2	line 3	Mean	% of mean	
Damsin 2						
0 hour	390507	401610	361172	384429,7	0	0
24 hour	259994	205497	216260	227250,3	59,11363	40,88637
48 hour	176156	54939	90738	107277,7	27,90567	72,09433
72 hour	171588	100222	81630	117813,3	30,64626	69,35374

	Line 1	line 2	line 3	mean	% of mean	
Control 1						
0 hour	284226	320010	285327	296521	0	0
24 hour	189006	108320	180665	159330,3	53,73324	46,26676
48 hour	99207	11325	57627	56053	18,90355	81,09645
72 hour	17960	0	0	5986,667	2,018969	97,98103

	Line 1	line 2	line 3	mean	% of mean	
Control 2						
0 hour	236726	247441	287100	257089	0	0
24 hour	130960	104913	173772	136548,3	53,11325	46,88675
48 hour	47077	6443	22833	25451	9,899685	90,10032
72 hour	21221	0	0	7073,667	2,751447	97,24855

Ambrosin 1	Line 1	line 2	line 3	mean	% of mean	
0 hour	281247	279829	270564	277213,3	0	0
24 hour	153382	132103	177875	154453,3	55,71642	44,28358
48 hour	88110	50570	25845	54841,67	19,7832	80,2168
72 hour	85333	2980	33686	40666,33	14,66969	85,33031

Ambrosin 2	Line 1	line 2	line 3	mean	% of mean	
0 hour	274292	267049	220138	253826,3	0	0
24 hour	196231	88245	114177	132884,3	52,35246	47,64754
48 hour	52476	2039	4070	19528,33	7,693581	92,30642
72 hour	76415	1000	240	25885	10,19792	89,80208

Wound healing assay 3. 1µM						0	24	48	72
Damsin 1	Line 1	line 2	line 3		mean	% of mean		hours	
0 hour	439646	409520	287639		378935	0	0	0	
24 hour	298140	184245	133033		205139,3	54,13576	45,86424	24	
48 hour	210419	62494	69302		114071,7	30,10323	69,89677	48	
72 hour	208209	41436	40365		96670	25,51097	74,48903	72	

Damsin 2	Line 1	line 2	line 3		mean	% of mean		
0 hour	428840	387559	359568		391989	0	0	0
24 hour	298322	190286	215606		234738	59,88382	40,11618	24
48 hour	213155	43297	106017		120823	30,82306	69,17694	48
72 hour	56924	0	80669		45864,33	11,70041	88,29959	72

Control 1	Line 1	line 2	line 3		mean	% of mean		
0 hour	395012	401028	357973		384671	0	0	0
24 hour	220767	225856	216113		220912	57,42882	42,57118	24
48 hour	132607	7625	82824		74352	19,32873	80,67127	48
72 hour	121119	0	79426		66848,33	17,37805	82,62195	72

Control 2	Line 1	line 2	line 3		mean	% of mean		
0 hour	305457	299117	305320		303298	0	0	0
24 hour	130798	62430	201756		131661,3	43,40989	56,59011	24
48 hour	34561	13842	75452		41285	13,61203	86,38797	48
72 hour	11295	0	17464		9586,333	3,160698	96,8393	72

Ambrosin 1	Line 1	line 2	line 3		mean	% of mean		
0 hour	389507	407816	379975		392432,7	0	0	0
24 hour	264619	220900	219107		234875,3	59,85112	40,14888	24
48 hour	157697	51421	115683		108267	27,58868	72,41132	48
72 hour	157017	57834	102769		105873,3	26,97873	73,02127	72

Ambrosin 2	Line 1	line 2	line 3		mean	% of mean		
0 hour	363499	367987	365185		365557	0	0	0
24 hour	237946	137496	233311		202917,7	55,50917	44,49083	24
48 hour	130234	4619	142181		92344,67	25,26136	74,73864	48
72 hour	137704	5166	86991		76620,33	20,95989	79,04011	72

Wound healing assay 4   1µM

Damsin	Line 1	line 2	line 3		mean	% of mean		hours	
0 hour	400104	328451	406190		378248,3	0	0		0
24 hour	275228	98698	233869		202598,3	53,56225	46,43775		24
48 hour	194031	64514	159835		139460	36,86996	63,13004		48
72 hour	130836	37876	125348		98020	25,91419	74,08581		72

Control	Line 1	line 2	line 3		mean	% of mean		hours	
0 hour	327960	313366	398327		346551	0	0		0
24 hour	170327	146131	225907		180788,3	52,16789	47,83211		24
48 hour	58906	73917	170173		100998,7	29,14395	70,85605		48
72 hour	51652	68485	115290		78475,67	22,64477	77,35523		72

Ambrosin	Line 1	line 2	line 3		mean	% of mean		hours	
0 hour	404931	261515	418688		361711,3	0	0		0
24 hour	274681	28870	312963		205504,7	56,81455	43,18545		24
48 hour	149479	0	214032		121170,3	33,49918	66,50082		48
72 hour	140547	0	115217		85254,67	23,56981	76,43019		72

Wound healing assay 5. 1µM

Damsin	Line 1	line 2	line 3		mean	% of mean		hours
0 hour	493367	462069	417786		457740,7	0	0	0
24 hour	327497	288821	272665		296327,7	64,73702	35,26298	24
48 hour	303447	257345	253580		271457,3	59,30374	40,69626	48
72 hour	178189	185214	165088		176163,7	38,48547	61,51453	72

Control	Line 1	line 2	line 3		mean	% of mean		hours
0 hour	603653	518883	461877		528137,7	0	0	0
24 hour	414418	333460	297391		348423	65,972	34,028	24
48 hour	341770	256166	222814		273583,3	51,80152	48,19848	48
72 hour	89140	0	76963		55367,67	10,48357	89,51643	72

Ambrosin	Line 1	line 2	line 3		mean	% of mean		hours
0 hour	548341	461794	545343		518492,7	0	0	0
24 hour	499764	256353	427185		394434	76,07321	23,92679	24
48 hour	421538	182015	371135		324896	62,66164	37,33836	48
72 hour	242813	72660	244498		186657	35,99993	64,00007	72

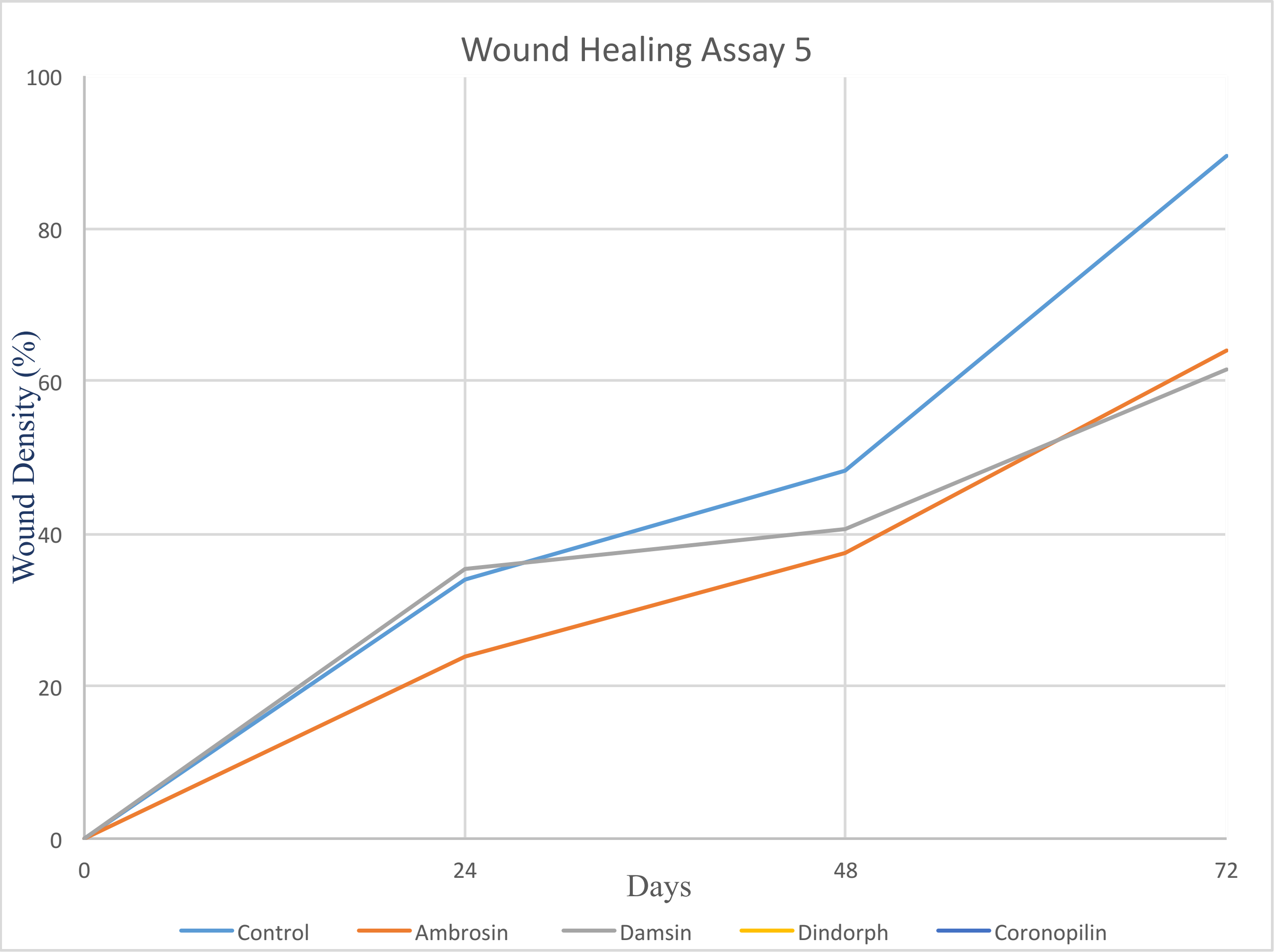

Wound healing 5 µM

				0	24	48	72
Damsin 1	Line 1	line 2	line 3	mean	% of mean		
0 hour	398899	284521	318649	334023	0	0	
24 hour	268354	111789	234431	204858	61,3305072	38,6694928	
48 hour	248395	87222	192672	176096,33	52,7198227	47,2801773	
72 hour	202881	73157	157552	144530	43,2694755	56,7305245	
Damsin 2	Line 1	line 2	line 3	mean	% of mean		
0 hour	367496	330597	418902	372331,67	0	0	
24 hour	220983	181126	358272	253460,33	68,0738052	31,9261948	
48 hour	221934	114936	353622	230164	61,8169285	38,1830715	
72 hour	187126	70350	346633	201369,67	54,0834113	45,9165887	
Control 1	Line 1	line 2	line 3	mean	% of mean		
0 hour	479678	484081	498781	487513,33	0	0	
24 hour	272926	231802	281339	262022,33	53,7467009	46,2532991	
48 hour	205602	150636	220561	192266,33	39,4381692	60,5618308	
72 hour	143070	99531	149877	130826	26,8353686	73,1646314	
Control 2	Line 1	line 2	line 3	mean	% of mean		
0 hour	419873	378224	485336	427811	0	0	
24 hour	236141	233655	353727	274507,67	64,1656401	35,8343599	
48 hour	139463	171268	297892	202874,33	47,421486	52,578514	
72 hour	107834	128289	111924	116015,67	27,1184394	72,8815606	
Ambrosin 1	Line 1	line 2	line 3	mean	% of mean		
0 hour	510552	509504	522853	514303	0	0	
24 hour	350771	241520	374091	322127,33	62,6337652	37,3662348	
48 hour	339350	204562	354687	299533	58,2405702	41,7594298	
72 hour	308488	200381	266970	258613	50,284171	49,715829	
Ambrosin 2	Line 1	line 2	line 3	mean	% of mean		
0 hour	432356	410365	456888	433203	0	0	
24 hour	392475	328608	407603	376228,67	86,8481212	13,1518788	
48 hour	337889	292558	404619	345022	79,6444161	20,3555839	
72 hour	337720	274380	371567	327889	75,6894574	24,3105426	

	Assay 1	Assay 1	Present	Present	Assay 3	Assay 3	Mean	SEM
Control	0	0	0	0	0	0	0	0
	67,7158982	49,3271992	46,2532991	35,8344	64,434759	64,3023325	54,6446414	5,420229
	91,2285709	77,786844	60,5618308	52,5785	90,181955	86,7903637	76,5213464	7,081716
	93,1429322	86,7382329	74,1646314	78,8816	92,543919	93,2214372	86,4487855	3,429154

	Assay 1	Assay 1	Present	Present	Assay 3	Assay 3	Mean	SEM
	0	0	0	0	0	0	0	0
Damsin	32,0827918	53,692083	38,6694928	31,9262	56,83407	62,5324558	45,9561814	4,175449
	48,9944322	81,5420407	47,2801773	38,1831	77,64068	88,8261909	63,744432	7,742204
	60,4772565	88,9354249	52,7305245	45,9166	90,245454	93,6730568	71,9963843	7,718788
Ambrosin	0	0	0	0	0	0	0	0
	36,0423244	41,4223937	37,3662348	13,1519	40,391416	66,1515183	39,0876277	5,213071
	42,603832	53,321072	41,7594298	20,3556	65,778115	68,5566644	48,7291162	5,638504
	50,5974215	53,7244948	49,715829	24,3105	70,988933	72,346148	53,6138948	5,563066

	0	24	48	72
				mean
Control	0	0		0
	46,2532991	35,8343599		41,0438
	60,5618308	52,578514		56,5702
	73,1646314	72,8815606		73,0231
Damsin	0	0		0
	38,6694928	31,9261948		35,2978
	47,2801773	38,1830715		42,7316
	52,7305245	45,9165887		49,3236
Ambrosin	0	0		0
	37,3662348	13,1518788		25,2591
	41,7594298	20,3555839		31,0575
	49,715829	24,3105426		37,0132

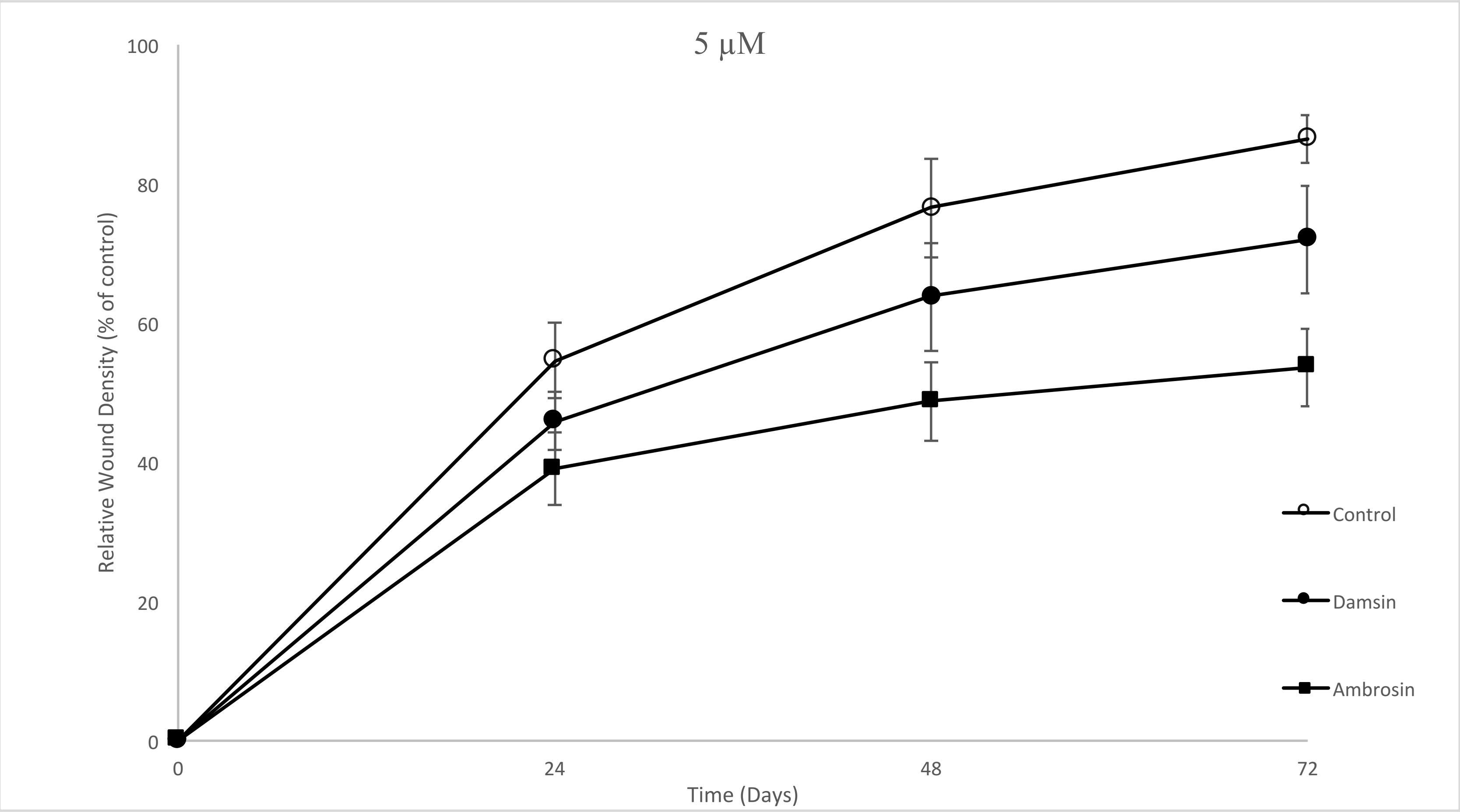

Wound healing assay 1. 5µM

			0	24	48	72		
Damsin 1	Line 1	line 2	line 3		mean	% of mean		
0 hour	380080	388081	503608		423923	0	0	
24 hour	224143	276786	362821		287916,6667	67,9172082	32,08279	
48 hour	128458	234783	285432		216224,3333	51,0055678	48,99443	
72 hour	101644	149232	251762		167546	39,5227435	60,47726	
Damsin 2	Line 1	line 2	line 3		mean	% of mean		
0 hour	368472	430082	524913		441155,6667	0	0	
24 hour	85852	173070	353948		204290	46,307917	53,69208	
48 hour	37774	60347	146164		81428,33333	18,4579593	81,54204	
72 hour	23329	54540	68567		48812	11,0645751	88,93542	
Control 1	Line 1	line 2	line 3		mean	% of mean		
0 hour	404974	425156	364681		398270,3333	0	0	
24 hour	160596	109692	115446		128578	32,2841018	67,7159	
48 hour	43914	30104	30784		34934	8,77142912	91,22857	
72 hour	21609	24398	35922		27309,66667	6,85706777	93,14293	
Control 2	Line 1	line 2	line 3		mean	% of mean		
0 hour	428397	456779	480087		455087,6667	0	0	
24 hour	192324	226250	273243		230605,6667	50,6728008	49,3272	
48 hour	89047	63623	150598		101089,3333	22,213156	77,78684	
72 hour	90216	36151	54691		60352,66667	13,2617671	86,73823	
Ambrosin 1	Line 1	line 2	line 3		mean	% of mean		
0 hour	397283	411705	474528		427838,6667	0	0	
24 hour	304851	274288	325986		273635,6667	63,9576756	36,04232	
48 hour	194957	253623	288109		245563	57,396168	42,60383	
72 hour	134969	212171	286950		211363,3333	49,4025785	50,59742	
Ambrosin 2	Line 1	line 2	line 3		mean	% of mean		
0 hour	397936	467887	550557		472126,6667	0	0	
24 hour	237232	346443	414537		276560,5	58,5776063	41,42239	
48 hour	216071	291396	153684		220383,6667	46,678928	53,32107	
72 hour	210330	291390	153717		218479	46,2755052	53,72449	
						Mean	SD	sem
		Control	0	0		0	0	0
			67,7158982	49,32719923		58,5215487	13,00277	9,194349
			91,2285709	77,786844		84,5077074	9,504736	6,720863
			93,1429322	86,73823285		89,9405825	4,528806	3,20235
		Damsin	0	0		0	0	0
			32,0827918	53,69208299		42,8874374	15,28008	10,80465
			48,9944322	81,54204072		65,2682364	23,01463	16,2738
			60,4772565	88,93542491		74,7063407	20,12296	14,22908
		Ambrosin	0	0		0	0	0
			36,0423244	41,42239371		38,732359	3,804284	2,690035
			42,603832	53,32107203		47,962452	7,578233	5,35862
			50,5974215	53,72449484		52,1609581	2,211175	1,563537

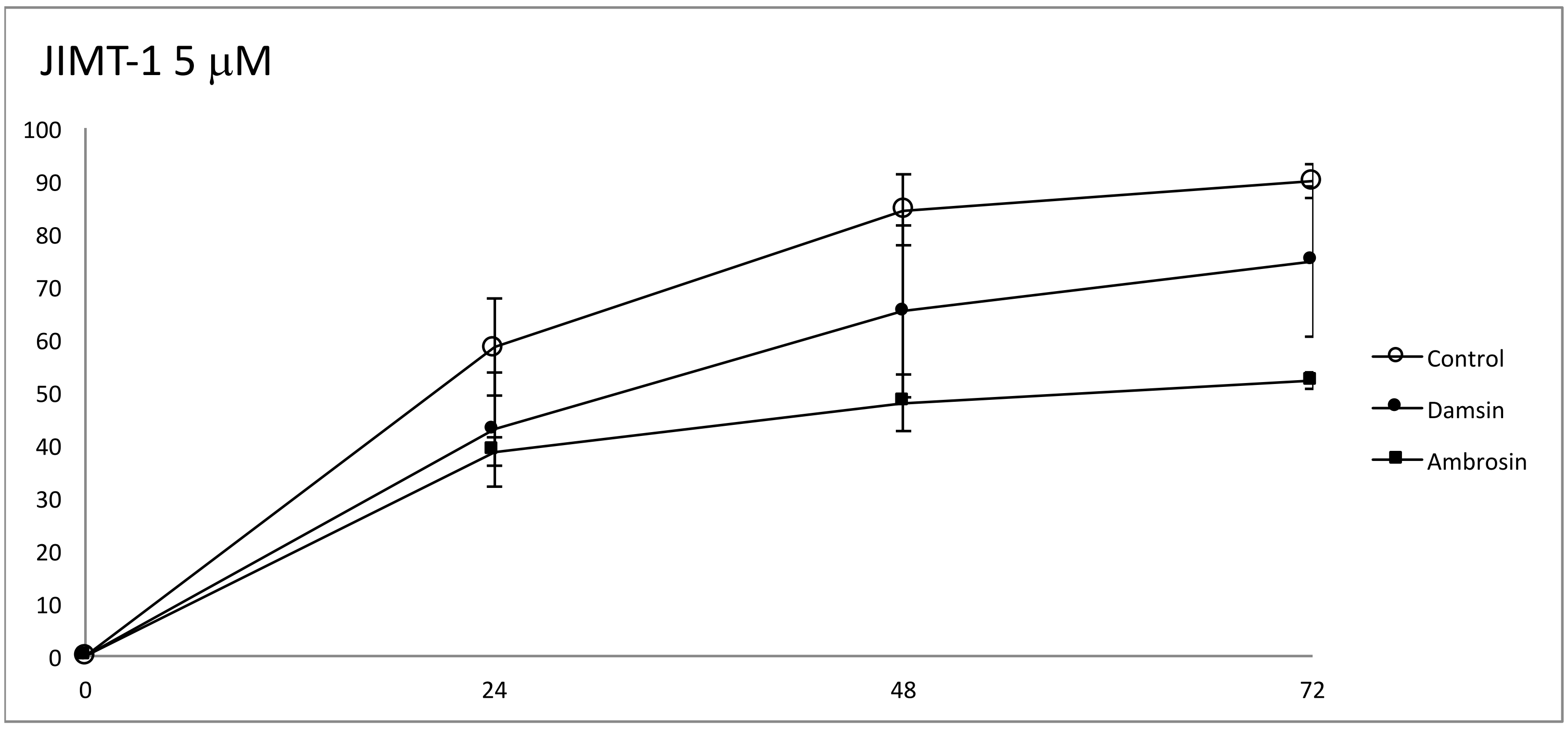
JIMT-1 5 μM
100
90
80
70
60
50
40
30
20
10
0
0
24
48
72
Control
Damsin
Ambrosin

Wound healing assay 3 5µM

Ambrosin 1	Line 1	line 2	line 3	mean	% of mean	
0 hour	402458	443558	419696	421904	0	0
24 hour	313687	227632	213154	251491	59,608584	40,39142
48 hour	274121	213727	175799	144383,5	34,221885	65,77812
72 hour	77701	20308	104645	67551,33	16,011067	83,98893

Ambrosin 2	Line 1	line 2	line 3	mean	% of mean	
0 hour	393069	417089	399914	403357,3	0	0
24 hour	176745	127420	105426	136530,3	33,848482	66,15152
48 hour	163936	113593	102958	126829	31,443336	68,55666
72 hour	91991	14807	34222	47006,67	11,653852	88,34615

Control 1	Line 1	line 2	line 3	mean	% of mean	
0 hour	472613	575946	558049	535536	0	0
24 hour	178861	136646	255887	190464,7	35,565241	64,43476
48 hour	91834	37007	66844	52579,17	9,8180452	90,18195
72 hour	64448	17246	38096	39930	7,4560814	92,54392

Control 2	Line 1	line 2	line 3	mean	% of mean	
0 hour	384383	343118	343611	357037,3	0	0
24 hour	196236	55836	130290	127454	35,697667	64,30233
48 hour	80495	5185	55810	47163,33	13,209636	86,79036
72 hour	58951	3856	9799	24202	6,7785628	93,22144

Damsin 1	Line 1	line 2	line 3	mean	% of mean	
0 hour	514553	491336	362878	456255,7	0	0
24 hour	246828	221519	122494	196947	43,16593	56,83407
48 hour	99650	125033	81364	102015,7	22,35932	77,64068
72 hour	36722	46036	50759	44505,67	9,7545455	90,24545

Damsin 2	Line 1	line 2	line 3	mean	% of mean	
0 hour	487722	424362	410482	440855,3	0	0
24 hour	213732	77095	204706	165177,7	37,467544	62,53246
48 hour	43946	17415	86420	49260,33	11,173809	88,82619
72 hour	30297	17237	36144	27892,67	6,3269432	93,67306
